# Searching for Novel Antiviral Agents as COVID19 Treatments: Guanidino Diaryl Thioureas

**DOI:** 10.1002/cmdc.202501000

**Published:** 2025-11-30

**Authors:** Marco Minneci, Barbara Farkaš, Adeyemi Rahman, Amy Kempf, Inga Nehlmeier, Stefan Pöhlmann, Isabel Rozas

**Affiliations:** ^1^ School of Chemistry Trinity College Dublin The University of Dublin TBSI Dublin D02R590 Ireland; ^2^ Infection Biology Unit German Primate Center 37077 Göttingen Germany; ^3^ Present address: Astex Pharmaceuticals Cambridge CB4 0QA UK

**Keywords:** docking, guanidino diaryl *N,N′*‐thioureas, SARS‐CoV‐2, TMPRSS2, TMPRSS2 inhibitor

## Abstract

The COVID‐19 pandemic highlighted the urgent need for effective antiviral treatments targeting SARS‐CoV‐2. TMPRSS2, a serine protease essential for viral entry into host cells, represents a promising therapeutic target, and this study explores guanidino diaryl thioureas as potential TMPRSS2 inhibitors. Initial screening identified a “hit‐compound” (**1**) with reversible inhibitory activity against TMPRSS2. Computational studies, including docking and molecular dynamics simulations, were conducted to optimize derivatives of compound **1**. Twenty‐five derivatives were synthesized, and their pharmacokinetic properties and cytotoxicity assessments indicated favorable drug‐likeness and minimal toxicity. However, biochemical studies revealed that none of the derivatives improved TMPRSS2 inhibitory activity compared to the original “hit‐compound”. The findings suggest that reversible inhibitors may be suboptimal for TMPRSS2 targeting, as camostat and nafamostat exert their effects through irreversible covalent binding. Future efforts should focus on developing irreversible TMPRSS2 inhibitors to enhance antiviral efficacy against SARS‐CoV‐2.

## Graphical abstract

Structure of the "hit‐compound" found and the proposed interaction with the active site of the TMPRSS2 protease, involving a reversible mechanism of action, are shown.

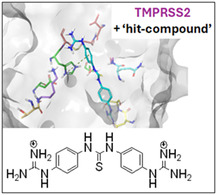

## 1 Introduction

The outbreak of the pathogenic SARS coronavirus 2 (SARS‐CoV‐2) and the ensuing COVID‐19 pandemic claimed almost 18.2 million lives in 2020 and 2021 alone^[^ ^1^ ^]^ and had a major impact on health care systems and economies. To date, only three antiviral agents, targeting the main viral protease or the viral RNA polymerase, are approved and effective for COVID‐19 therapy.^[^ ^2^, ^3^, ^4^ ^]^ However, resistance development and unwanted side effects can limit the clinical usefulness of these agents. Therefore, it is important to explore new options for the treatment of SARS‐CoV‐2 infections.

**Figure 1:**
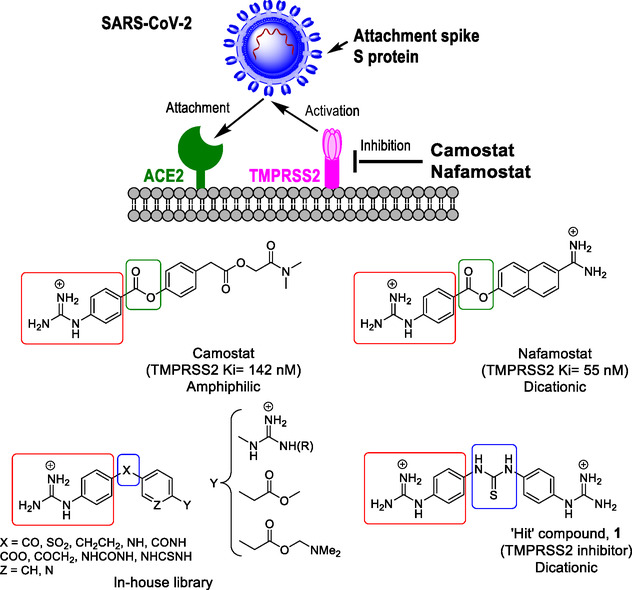
Cell entry of SARS‐CoV‐2 and its inhibition. Upper panel: The viral spike (S) binds to ACE2 on the surface of target cells and is subsequently activated by the protease TMPRSS2. This activation can be inhibited by the clinically approved protease inhibitors camostat or nafamostat. Lower panel: Structure of the known TMPRSS2 inhibitors camostat and nafamostat with the corresponding inhibition constants and general structures of compounds in the in‐house library and that of the “hit” compound (**1**) found in the present study, highlighting shared or related features.

Entry into target cells is the first step of the viral replication cycle and constitutes and attractive target for therapeutic intervention. The SARS‐CoV‐2 spike protein (S) mediates host cell entry and constitutes the key target of the neutralizing antibody response. For cell entry, the receptor binding domain (RBD) located within the surface unit S1 of the viral S protein binds the carboxypeptidase angiotensin‐converting enzyme 2 (ACE2) expressed in the surface of lungs, arteries, heart, kidneys, and intestine cells.^[^ ^5^, ^6^, ^7^, ^8^, ^9^, ^10^ ^]^ This S protein binds to ACE2 with high affinity and the receptor activity of ACE2 is independent of its enzymatic activity.^[^ ^11^ ^]^ Upon ACE2 engagement, the S protein is cleaved and activated by a cellular protease. The activation enables the transmembrane unit S2 of the S protein to fuse the viral and a cellular membrane, resulting in the delivery of the viral genetic information into the host cell cytoplasm (**Figure** **1**). For entry into lung cells, the S protein depends on cleavage by furin at the S1/S2 site in infected cells and on subsequent cleavage by transmembrane protease serine 2 (TMPRSS2) at the S2’ site during cell entry.^[^ ^10^ ^,^ ^12^, ^13^, ^14^, ^15^ ^]^ In cell culture settings, SARS‐CoV‐2 can also use the endosomal cysteine protease cathepsin L for cell entry, and omicron subvariants, which dominate the COVID‐19 pandemic and ensuing endemic since the winter season of 2021, prefer cathepsin L over TMPRSS2 for entry into cell lines.^[^ ^10^ ^,^ ^16^ ^,^ ^17^ ^]^ However, inhibitor studies with primary respiratory epithelial cells and analyses of TMPRSS2 KO cells, organoids, and mice indicate that TMPRSS2 is of central importance to spread and pathogenesis of pre‐omicron and omicron variants.^[^ ^18^, ^19^, ^20^, ^21^, ^22^, ^23^, ^24^, ^25^ ^]^

The serine protease inhibitor camostat has been approved in Japan for use in human pancreatic inflammation^[^ ^26^ ^]^ and is active against TMPRSS2. Camostat inhibits TMPRSS2 with a *K*_i_ = 142 nM, and the related compound nafamostat shows even stronger inhibition with a *K*_i_ = 55 nM. In cell culture, camostat and nafamostat inhibit SARS‐CoV‐2 infection with high efficiency (Figure 1).^[^ ^10^ ^,^ ^22^ ^,^ ^25^ ^,^ ^27^ ^,^ ^28^ ^]^ However, clinical trials mainly revealed little if any benefit to COVID‐19 patients,^[^ ^29^, ^30^, ^31^, ^32^, ^33^, ^34^, ^35^, ^36^, ^37^, ^38^, ^39^ ^]^ likely because oral (camostat) and intravenous (nafamostat) application did not allow for inhibitor concentrations in the respiratory tract sufficient for suppression of SARS‐CoV‐2 infection. In this context, it is noteworthy that a TMPRSS2 inhibitor unable to interfere with SARS‐CoV‐2 infection upon systemic application in a rodent model was able to inhibit SARS‐CoV‐2 with high efficiency upon topical application.^[^ ^40^ ^]^ Further, several TMPRSS2 inhibitors were found to efficiently block infection upon topical application in rodent models.^[^ ^40^, ^41^, ^42^, ^43^ ^]^ Finally, aerosolized aprotinin, another protease inhibitor active against TMPRSS2, was shown to be well tolerated and of clinical benefit in COVID‐19 patients.^[^ ^44^ ^]^

Considering that TMPRSS2, which is dispensable for development and homeostasis in mice,^[^ ^45^ ^]^ constitutes an attractive target for antiviral intervention and that some of the structural features of the TMPRSS2 inhibitors discussed above are present in diphenyl guanidiniums from our in‐house library (Figure 1), we tested several of these in‐house library compounds for their inhibition of TMPRSS2. We identified a bis‐guanidino diaryl thiourea as a “hit” compound (**1**, Figure 1), and now we report on the rational design and synthesis of novel thiourea based TMPRSS2 inhibitors.

## 2 Results and Discussion

### 2.1 Structural Design

The crystal structure of TMPRSS2 had not been resolved when the in‐house library was screened for potential inhibitors, and hence the actual mechanism of inhibition of nafamostat and camostat was thought to go through competitive reversible binding. Thus, virtual screening of some of the guanidine‐based derivatives from the in‐house library (Figure 1, and Table S1, Supporting Information) was conducted in the TMPRSS2 homology model developed by Singh et al.^[^ ^46^ ^]^ using the UniProt sequence (ID O15393) and templating it in SWISS MODEL server.^[^ ^47^ ^]^ Molecules were geometrically optimized by means of density functional theory (DFT) calculations with the Gaussian16 software^[^ ^48^ ^]^ using M06‐2X functional and 6‐31+G(d,p) basis set in implicit water SMD model and afterwards docked with Autodock Vina docking suite, version 1.2.1.^[^ ^49^ ^]^ We found that some of these compounds could fit not only near the catalytic triad but also in different hydrophobic pockets around the catalytic site several pockets within the TMPRSS2 structure; thus, these derivatives were submitted for testing as TMPRSS2 inhibitors, and “hit‐compound” **1** was identified.

In 2021, the structure of the complex between nafamostat and TMPRSS2 was resolved (PDBID 7MEQ)^[^ ^50^ ^]^ evidencing its irreversible mechanism of inhibition forming a covalent bond with the target. Direct comparison of the homology model and X‐ray structure has shown no noticeable differences in protein folding and residue side chain positioning. With that information in hand, several families of new derivatives were proposed to optimally interact with the binding site of the enzyme (near the catalytic triad and in different hydrophobic pockets around the catalytic site) potentially inhibiting its protease activity. The compounds considered in the present study, together with the original “hit‐compound” from our in‐house library, include asymmetric diaryl thiourea derivatives with potential as noncovalent competitive reversible inhibitors.

**Figure 2:**
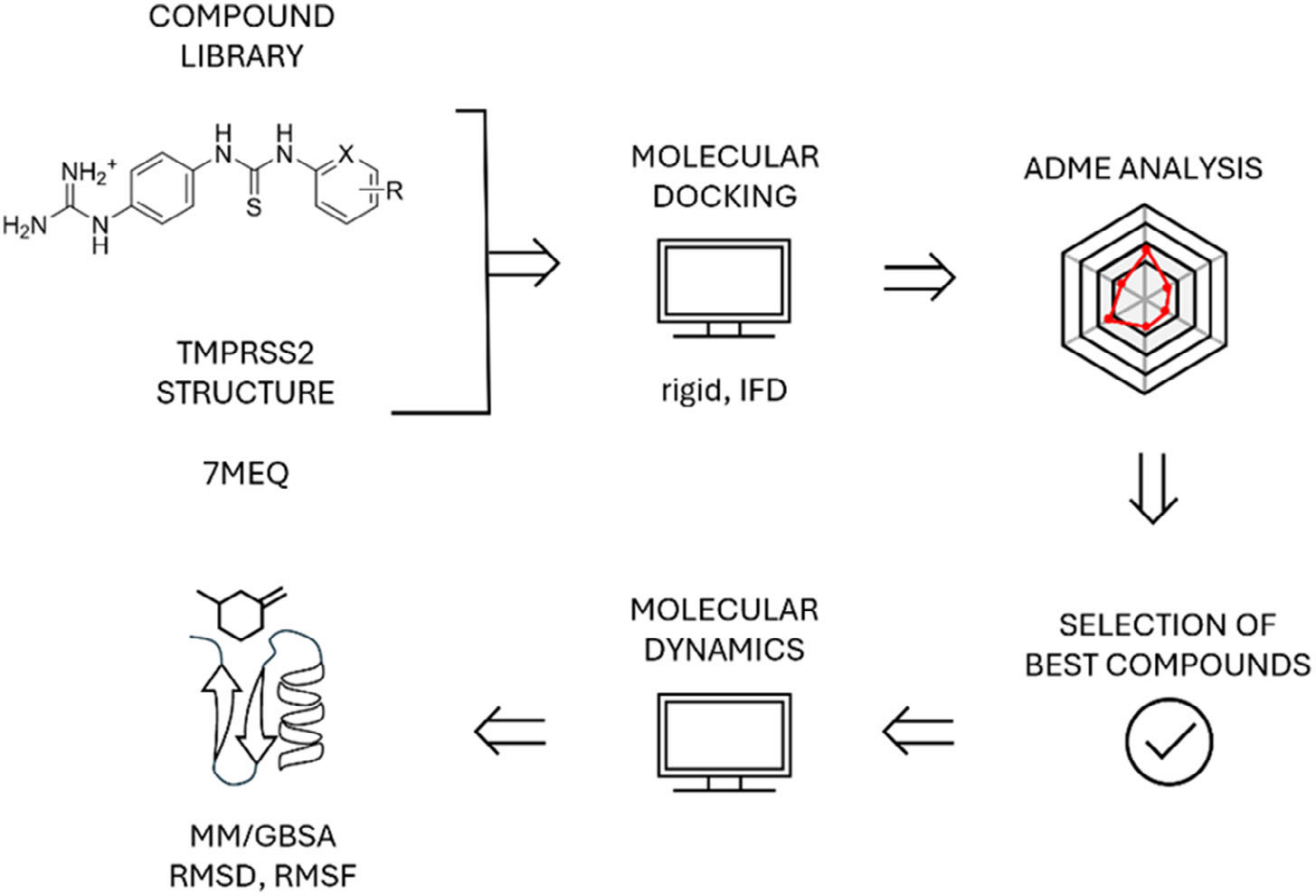
Schematic representation of the methodology: a library of compound analogs to the hit compound were docked into TMPRSS2 crystal structure (PDB: 7MEQ) using Glide XP and IFD protocols, protein‐ligand interactions and compound ADME properties were analyzed, and the best follow up hits were taken into molecular dynamics simulations with Desmond for MM/GBSA, RMSD, and RMSF analysis.

Modeling protocol to establish the interactions, drug‐likeness, and rank the proposed compounds according to their likelihood of TMPRSS2 inhibition with the crystal structure at hand combined molecular docking and pharmacokinetic properties analysis (i.e., ADME: absorption, distribution, metabolism, and excretion), after which the best compounds were further followed up in molecular dynamics (MD) simulations to determine protein–ligand complex stability. The schematic representation of computational workflow is shown in **Figure** **2**.

### 2.2 Docking Studies

As mentioned, the initial virtual screen of compounds within the in‐house library was performed on a homology model using the Autodock Vina software, version 1.2.1.^[^ ^49^ ^]^ The binding site is visualized in Figure S1A, Supporting Information, and a number of compounds were identified by Autodock Vina to have an improved binding potential to TMPRSS2 homology model compared to camostat (Table S1, Supporting Information). Representative binding modes for nafamostat and an in‐house guanidine derivative hit are shown in Figure S1B,C, Supporting Information, whereas the positioning of the charged guanidinium and amidinium *functional* *ities* and the placement of aromatic groups coincides between most of the in‐house analogs and literature inhibitors camostat and nafamostat, adding confidence in the potential of the in‐house compounds to effectively inhibit the target.

**Figure 3:**
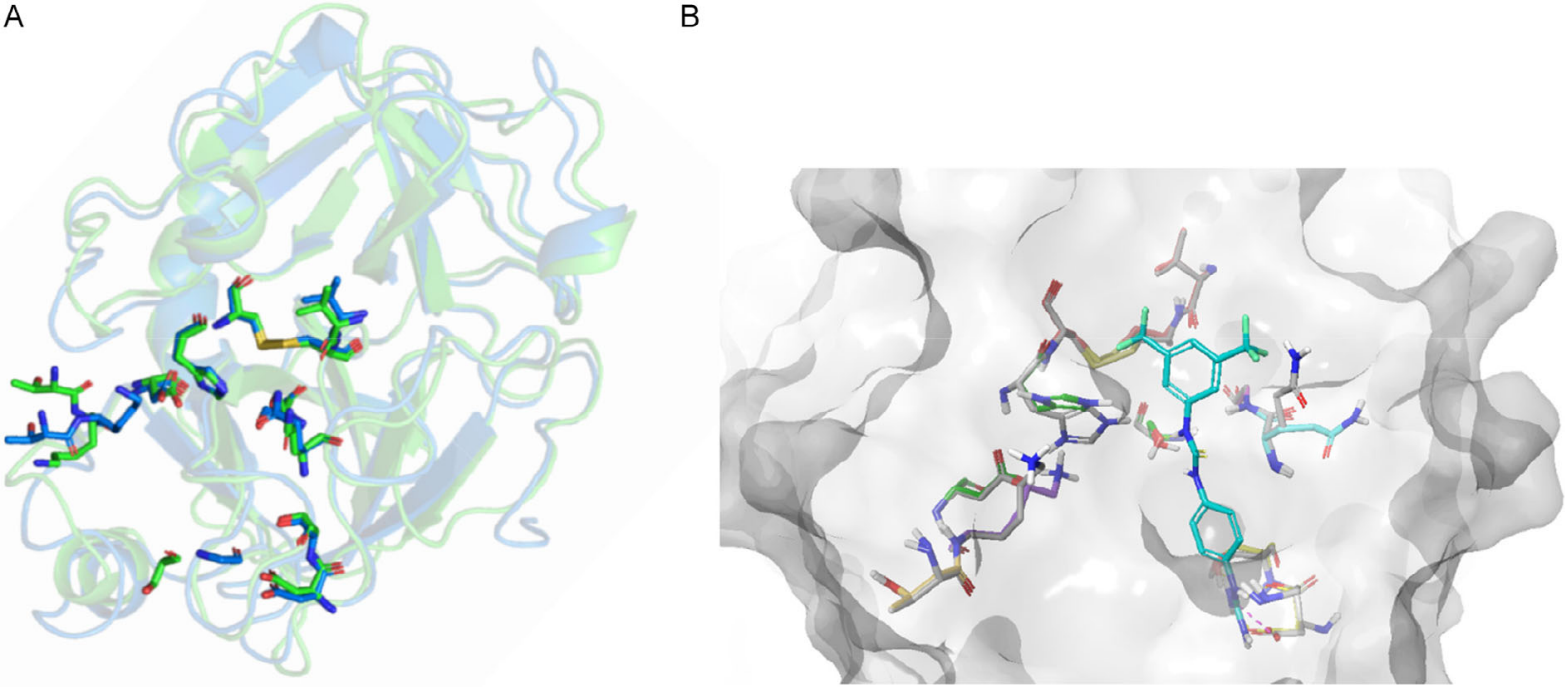
A) Comparison between the homology model (Uniprot ID O15393, green) and crystal structure (PDBID 7MEQ, blue) of TMPRSS2. Key binding site residues are shown in atomic code colors. B) Example of induced fit movement observed in the protein for docking of compound **18** (apo TMPRSS2 binding site residues in gray, IFD TMPRSS2 binding site residues with docked compound **18** in colors corresponding to atom code).

Based on the structure of the “hit‐compound” **1** and using as template the actual crystal structure of TMPRSS2 complexed with nafamostat (PDBID: 7MEQ), several derivatives of the “hit‐compound” (compounds **2**–**25**, see structures in Table S2, Supporting Information) were explored to optimally interact with the binding site of the enzyme (catalytic triad and nearby hydrophobic pockets) in a noncovalent, reversible manner to potentially inhibit the protease activity. Since the homology model and crystal structure of TMPRSS2 are very similar at the binding site (**Figure** **3**A), it is not surprising that the binding modes have been conserved when docking (using Autodock Vina 1.2.1 and confirmed using Glide XP and IFD protocols) was repeated in the newly resolved crystal structure (Table S2, Supporting Information). Induced fit uncovered small side chain perturbations around the binding site which allow for better volumetric accommodation and tighter binding of proposed compounds (see example on compound **18** in Figure 3B).

Most common flips occurred on residues Q438, H296, and S441. Only seven of the 24 compounds maintained the binding mode of the “hit‐compound” when subjected to the IFD calculations (compound hit‐like pose either the best scoring pose or within 0.3 kcal mol^−1^ of the best scoring pose), with lipophilic tails of majority of the remaining analogs preferred to shift into the adjacent pocket A. None of the docking scores exceeded that of the initial “hit‐compound”. A list of interactions included in Table S2, Supporting Information, identified a consistent set of hydrogen bonding network arising from a high number of NH and NH_2_ donors present in the hit analogs but also highlighted an importance of *π–* *π* stacking with H296 and formation of the salt bridge with, in majority of cases, acidic side chain of D435. Examples of interaction diagrams post IFD are shown in Figure S2, Supporting Information, for nafamostat and “hit‐compound”.

### 2.3 Assessment of Pharmacokinetic (ADME) Properties

Drug‐likeness of the compound library has been assessed using the SwissADME online tool^[^ ^51^ ^]^ and based on the physicochemical properties [including molecular weight (MW), number of hydrogen bond donors and acceptors (HBD and HBA), calculated lipophilicity (cLogP), topological polar surface area (TPSA), and water solubility] and the presence of any pan assay interference structures (PAINS)^[^ ^52^ ^]^ which indicate disruptive functional groups with high nonspecificity to numerous biological targets. Results have been summarized in **Table** **1**. Only three of the selected compounds have increased HBD count to what is defined favorable by the Lipinski's rule of five, and, with the exception of compound **8** which contains an aniline functional group included in the list of known PAINS, the whole library is demonstrating good drug‐ and lead‐likeness properties.

**Table 1:** Drug‐likeness metrics for the compound library calculated by the SwissADME tool.

	MW	HBD	HBA	cLogP	TPSA	Water solubility	Lipinski violations	PAINS
**1 (hit)**	344.44	8	0	0.21	183	Moderate	1, HBD > 5	–
**2**	286.38	5	0	1.34	120	Moderate	–	–
**3**	304.37	5	1	1.83	120	Moderate	–	–
**4**	365.27	5	0	2.15	120	Moderate	–	–
**5**	300.40	5	0	1.88	120	Moderate	–	–
**6**	342.48	5	0	2.80	120	Poor	–	–
**7**	331.37	5	2	0.79	166	Moderate	–	–
**8**	301.39	6	0	1.00	146	Moderate	1, HBD > 5	Yes
**9**	314.43	5	0	2.25	120	Moderate	–	–
**10**	376.50	5	0	3.13	120	Poor	–	–
**11**	378.47	5	1	2.98	129	Poor	–	–
**12**	310.40	5	0	1.88	120	Moderate	–	–
**13**	316.40	5	1	1.60	129	Moderate	–	–
**14**	314.43	5	0	2.24	120	Moderate	–	–
**15**	379.30	5	0	2.65	120	Poor	–	–
**16**	388.82	5	3	3.16	120	Poor	–	–
**17**	346.43	5	2	1.55	138	Moderate	–	–
**18**	422.37	5	6	3.56	120	Poor	–	–
**19**	340.47	5	0	2.52	120	Poor	–	–
**20**	287.36	5	1	0.91	133	Moderate	–	–
**21**	301.39	5	1	1.27	133	Moderate	–	–
**22**	301.39	5	1	1.23	133	Moderate	–	–
**23**	301.39	5	1	1.24	133	Moderate	–	–
**24**	321.81	5	1	1.41	133	Moderate	–	–
**25**	326.40	6	1	1.32	148	Moderate	1, HBD > 5	–

### 2.4 Molecular Dynamics (MD) Study

**Figure 4:**
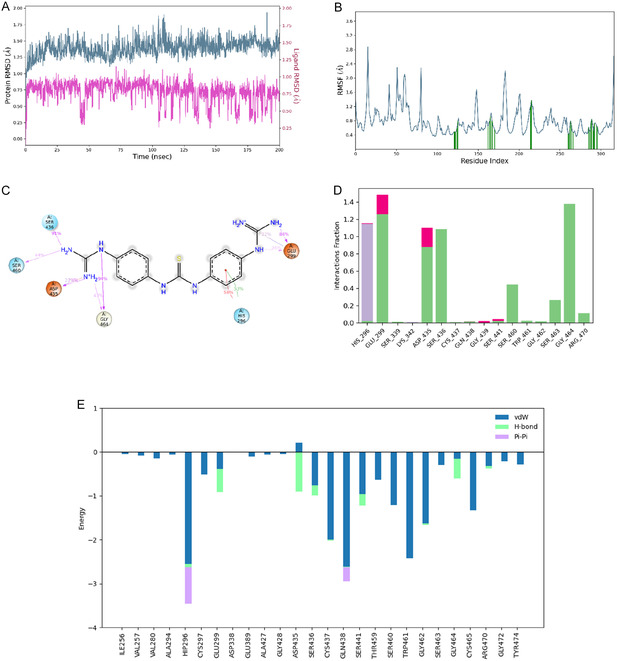
Statistics of 200 ns MD simulation of the hit compound **1** bound to TMPRSS2: A) protein and ligand RMSDs, B) protein RMSF, C) 2D representation of ligand‐protein contact frequencies, D) histogram of ligand–protein interaction fractions, and E) MM/GBSA decomposition of interactions between ligand and binding site residues.

Docking studies indicated that the initial “hit‐compound” remained the best binder amongst the analogs, and, hence, it was subsequently simulated for 200 ns in an MD calculation to evaluate the stability of the binding mode and established ligand‐protein interactions. Statistics across the full length of the simulation are shown in **Figure** **4**. The binding mode is stable throughout the simulation with ligand RMSD below 1 Å (Figure 4A), and there are no major shifts in the protein either as demonstrated by continuous RMSD between 1.25 and 1.5 Å and minimal spikes in the per residue RMSF values (Figure 4A,B). Main protein‐ligand contacts captured by docking are maintained as the most frequent interactions (Figure 4C,D), confirming the key role of the two terminal guanidinium moieties.

To confirm the strength of the binding, thermal MM/GBSA energy has been calculated over the second half of the trajectory (from 100–200 ns), with intervals of 100 ps, amounting to 1000 frames and summing up to a binding energy of −61.5 kcal mol^−1^. Decomposition of MM/GBSA interaction energies per residue averaged over the second half of the trajectory is shown in Figure 4E. In this plot, we can observe how van der Waals forces dominate most of the attractive interactions of the ligand (“hit” compound **1**) and the residues of the target TMPRSS2; these are followed by HBs with Hip296, Glu299, Asp432, Ser436, Ser441, Gly464, and Arg470, being the stronger contributions to HBs the interactions with the acidic residues (i.e., Asp and Glu) probably through the guanidinium moiety. Finally, pi–pi interactions contribute the less to the MM/GBSA interaction energy.

### 2.5 Synthesis

The structure of our “hit‐compound” as well as that of nafamostat and camostat present similar characteristics: (i) two aromatic systems, (ii) a carbonyl linker connecting those, and (iii) at least one cationic system (guanidinium or amidinium). Considering the positive results from the computational studies, the thiourea motif in the "hit compound", and our experience in this type of derivatives,^[^ ^53^ ^,^ ^54^ ^]^ we have prepared a series of *N,N′*‐(4‐guanidinophenyl)(aryl) thiourea derivatives to be tested as TMPRSS2 inhibitors.

**Scheme 1:**
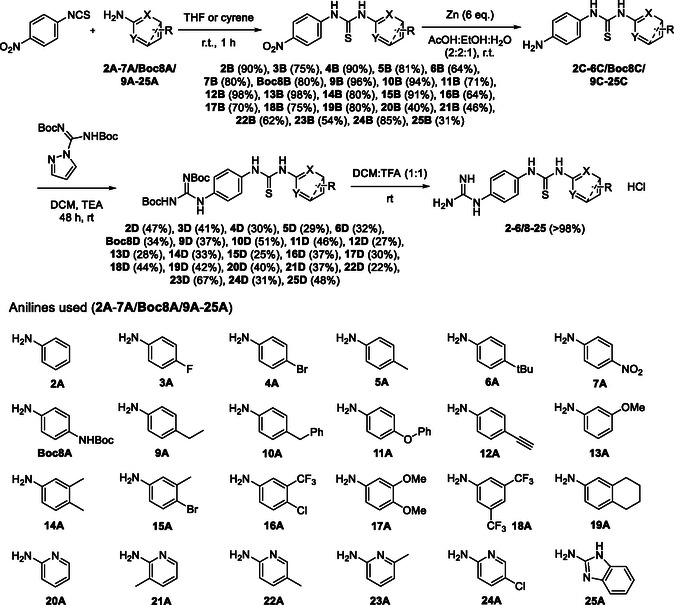
General route for the preparation of guanidino *N,N′*‐diaryl thioureas, indicating the anilines used.

The synthesis of all compounds **2**‐**25** requires first the preparation of the corresponding ‘Boc’ protected derivatives **2D**‐**6D/Boc8D/9D**‐**25D** by installing the Boc‐protected guanidinyl group using *N,N′*‐bis‐Boc protected pyrazole‐1‐carboxamidine as guanidylating agent on the anilino‐thioureas **2C**‐**6C/Boc8C/9C**‐**25C**. The latter are obtained upon Zn reduction of nitrophenyl thioureas **2B**‐**6B/Boc8B/9B**‐**25B** which are easily prepared by reacting the commercially available 4‐nitrophenyl isothiocyanate and the desired anilines **2A**‐**6A/Boc8A/9A**‐**25A** (**Scheme** **1**). Such synthetic work and its optimization have been extensively discussed in a previous publication by us with anilines **2A‐6A**, **Boc8A**‐**15A**, **18A‐21A**, and **24A‐25A**.^[^ ^54^ ^]^ In the present article, four additional anilines (**16A**, **17A**, **22A**, and **23A**) were utilized to complete the series of *N,N′*‐[(4‐guanidinophenyl)(aryl)]thiourea derivatives for testing (Scheme 1).

**Scheme 2:**
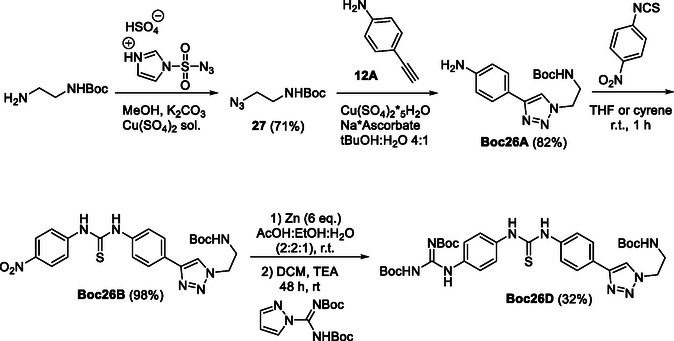
Preparation of starting aniline **Boc26A** and Boc‐protected *N,N′*‐(guanidinophenyl)(triazolophenyl)‐thiourea **Boc26D**.

The aniline bearing a terminal alkyne (**12A**) was further modified by means of “click” chemistry (**Scheme** **2**). To do so, mono Boc‐protected ethyldiamine was reacted with the diazo‐transfer reagent 1‐(azidosulfonyl)‐1*H*‐imidazol‐3‐ium hydrogen sulfate.^[^ ^55^ ^]^ Then, the azido‐compound thus obtained (**27**) was reacted with aniline **12A** to obtain the corresponding triazole system in the presence of Cu(II). Two different sources of Cu(II) were screened, i.e., Cu(OAc)_2_·H_2_O and Cu(SO_4_)_2_·5H_2_O, but only the sulfate salt appeared to be effective. Different solvent mixtures were also tested finding that the MeOH/H_2_O system did not work unlike the ^t^BuOH/H_2_O mixture which yielded the desired product in quantitative fashion. Next, the obtained aniline **26A** was reacted with *p*‐nitrophenyl isothiocyanate to yield the corresponding nitrophenyl thiourea **26B** that was used to achieve the corresponding *N,N′*‐[4‐(di‐Boc‐guanidino)phenyl](aryl) thiourea **26D** as described in Scheme 2.

Finally, Boc‐protected *N,N′*‐[(4‐guanidinophenyl)(aryl)]thioureas (**2D**‐**6D**/**Boc8D**/**9D**‐**25D**/**Boc26D**) were deprotected upon 4 h treatment with a DCM:TFA (1:1) solution (Scheme 1). Completion of the reaction was assessed by either ^1^H‐NMR (ca. 50 µL aliquot diluted in 600 µL of CD_2_Cl_2_) or C18 RP TLC (H_2_O:MeOH, 1:1). The solvent mixture was next removed at low pressure, and the excess TFA was eliminated by triturating with Et_2_O. Such procedure afforded all the final compounds in quantitative yields.

### 2.6 Preliminary Toxicity Assessment

The aim of this work is to find selective TMPRSS2 inhibitors; therefore, interactions with other biological systems in the body can drive to unwanted toxicities. For that reason, and since many bis‐guanidinium structures, such as the “hit‐compound”, are known to be DNA minor groove binders, the ability of these novel compounds to bind to DNA was also tested to assess their potential cytotoxicity. UV‐Thermal DNA denaturation experiments provide a very good information on the affinity of compounds towards DNA (i.e., the thermal melting temperature, Δ*T*_m_, which is the difference in melting between that of isolated DNA and that of the DNA–ligand complex). We have previously reported that the thermal melting temperature of the “hit‐compound” with a DNA oligo rich in AT pairs (poly(dA•dT)_2_) was Δ*T*_m_ = 23.0 °C indicating a strong binding to this oligo what would not be a desired feature for an antiviral drug.^[^ ^53^ ^]^ However, when DNA thermal denaturation experiments were performed with salmon testes DNA and sample compounds **2** and **20**, it was found very small to null Δ*T*_m_ values (0.65 °C and −0.25 °C, respectively, Figure S3, Supporting Information) indicating that these particular molecules are not prone to interact with DNA. This result is in agreement with previous reports from our group indicating the need of at least two guanidinium cations for binding to DNA.^[^ ^56^ ^,^ ^57^ ^]^

Additionally, the cytotoxicity of the new *N,N′*‐(4‐guanidinophenyl)(aryl) thioureas prepared was tested to assess whether they can be harmful to healthy human cells or not. The assay implemented is the well‐established cell viability AlamarBlue in vitro assay, and the breast MCF‐10A cells were chosen as a model of noncancerous cells. As a sample compound, derivative **2** was tested using concentrations up to 100 µM, and even at such high concentrations, no significant cell‐death was detected (Figure S4, Supporting Information). An IC_50_ value for this compound could not be found, suggesting that these molecules present not a significant risk to the cytoplasm environment.

### 2.7 Biochemical Study

Guanidinium salts **1**‐**26** were then evaluated for their ability to prevent SARS‐CoV‐2 from entering cells. For this, we used pseudotyped particles bearing the SARS‐CoV‐2 B.1 S protein (amino acid sequence identical to that of Wuhan‐Hu‐1 S protein but includes mutation D614G). These particles are safe and mimic key aspects of SARS‐CoV‐2 cell entry and its inhibition.^[^ ^58^ ^]^ Particles pseudotyped with the glycoprotein of vesicular stomatitis virus (VSV‐G) were used as controls since VSV‐G‐driven entry is independent of ACE2 and TMPRSS2. As target cells, we used Calu‐3 human lung cells, which express endogenous TMPRSS2 and allow for TMPRSS2‐dependent cell entry, and Vero76 African green monkey kidney cells, which do not express TMPRSS2 but allow for cathepsin L‐dependent entry.^[^ ^59^ ^]^ Finally, we used the serine protease inhibitor camostat to test for TMPRSS2‐dependence of viral entry and chloroquine, which elevates endosomal pH, to test for cathepsin L‐dependent entry, since low pH is required for cathepsin L activity. In addition, chloroquine is known to block entry of VSV and other viruses that require low pH for triggering of their glycoproteins.

Entry into Vero76 cells driven by the SARS‐CoV‐2 S protein and VSV‐G was not inhibited by camostat but was reduced by the highest concentration of chloroquine tested (Figure S5, Supporting Information), in agreement with expectations. Further, SARS‐CoV‐2 S protein‐mediated entry into Calu‐3 cells was inhibited by camostat but not chloroquine in a dose dependent manner, while the reverse observation was made for VSV‐G‐driven entry (Figure S6, Supporting Information), in keeping with expectations. In contrast, none of the compounds tested reduced SARS‐CoV‐2 S protein or VSV‐G‐driven entry into Vero76 (TMPRSS2‐independent SARS‐CoV‐2 S protein‐driven entry) or Calu‐3 cells (TMPRSS2‐dependent SARS‐CoV‐2 S protein‐driven entry), suggesting that they failed to inhibit TMPRSS2 activity or were not able to reduce TMPRSS2 activity to a degree that resulted in antiviral activity (Figure S5–S16, Supporting Information).

## 3 Conclusion

Considering structural similarities found between the known TMPRSS2 inhibitor camostat and compounds in our in‐house library, a “hit‐compound” (**1**) was found to interfere with the entrance of SARS‐CoV‐2 into host cells through TMPRSS2 interaction. Considering the limited knowledge of the TMPRSS2 structure at the time, it was assumed that the inhibition of TMPRSS2 by compound **1** was effected through a reversible interaction. Accordingly, in a following up optimization step, a thorough computational study was carried out to understand the interaction between TMPRSS2 and **1** and to proposed optimized derivatives. Thus, flexible docking studies were performed with 24 derivatives of compound **1** (compounds **2–25**) and TMPRSS2, followed by the assessment of their ADME properties. Finally, MD simulations between **1** and TMPRSS2 were carried out to better understand the interactions established by this type of compounds and the protease. These studies showed that all the compounds proposed could be good inhibitors of TMPRSS2, and therefore, they were prepared following standard synthetic pathways followed in Rozas’ group. Additionally, a new derivative was prepared following “click” chemistry procedures (**26**). All compounds were then tested for their ability to block SARS‐CoV‐2 from entering host cells, but unfortunately none of them showed improved TMPRSS2 inhibitory activity compared to that of the “hit” compound **1**. Several reasons can explain this disappointing results. Nowadays, it is known that camostat as well as its analog nafamostat exert their TMPRSS2 inhibition through an irreversible mechanism of action by covalently binding the phenylguanidinium moiety to the S332 residue; therefore, a reversible type of inhibitors will not be optimal blockers of TMPRSS2. However, compound **1** showed a certain degree of inhibitory activity that only could be exerted through reversible interaction to the target; therefore, the difference between compound **1** and the rest of the derivatives should reside on the interactions established with TMPRSS2, and, in fact, the HB and salt bridge formed between a guanidinium group of **1** and the E299 residue of the protease is not observed in any of the other compounds tested (see Figure 4 and Table S2, Supporting Information); therefore, these interactions seem to be key for the activity observed in the “hit‐compound”. In any case, future efforts in developing TMPRSS2 inhibitors should focus on compounds with an irreversible mechanism of action.

## 4 Experimental Section

4.1

4.1.1

##### Computational Studies

Details of the initial docking of the “in‐house” library screening can be found in the Supporting Information. In the following modeling step, compound library was prepared using LigPrep with Epik^[^ ^60^ ^]^ at physiological conditions (pH = 7.0 ± 0.4) to determine the favored tautomerization and protonation states, while TMPRSS2 was preprocessed via Protein Preparation Wizard^[^ ^61^ ^]^ as provided in the Schrodinger Suite (version 2020–1) and covalently modified serine residue recovered into its natural state. Molecular docking was performed using both Autodock Vina 1.2.1 docking tool to compare with the initial homology model screen and Glide^[^ ^62^ ^,^ ^63^ ^]^ with the extra precision (XP) scoring function and induced fit docking (IFD)^[^ ^64^ ^]^ to account for any protein movement in the near vicinity of the bound ligand.

The drug‐likeness was probed in the form of ADME profiles using the online SwissADME tool,^[^ ^51^ ^]^ focusing on the number of hydrogen bond (HB) donors and acceptors, polar surface area (TPSA), and ligand lipophilicity (logP). Established protein‐ligand interactions, stability, and affinity were thoroughly investigated by performing MD simulations and analysis of the root mean square deviation (RMSD) of both ligand and protein, root mean square fluctuation (RMSF) on per residue level, protein‐ligand contact analysis, and molecular mechanics/generalized Born surface area (MM/GBSA) calculations, all performed via the Desmond MD software.^[^ ^65^ ^]^ Standard MD protocol was used, including the initial minimization using the conjugate gradient algorithm, short 10 ns NVT equilibration including the gradual increase in system temperature from 10 to 300 K with restraints on heavy atoms to allow solvent relaxation, a 5 ns NPT equilibration with heavy atom restrains followed by another 5 ns with released restraints, and finally a 200 ns NPT production stage with trajectory output saved at 100 ps intervals, where the binding free energies were assessed over the final 100 ns using the thermal MM/GBSA approach over the final 100 ns with intervals of 100 ps, selecting 1000 frames. For NVT and NPT stages, Nose–Hoover chain thermostat and isotropic Martyna–Tobias–Klein barostat were used, with relaxation times of 1.0 and 2.0 ps, respectively.

##### General Procedures for the Boc‐Guanidylation of N,N′‐[(4‐Aminophenyl)(Aryl)]Thioureas and for their Boc‐Deprotection

The corresponding *N,N′‐*[(4‐aminophenyl)(aryl)]thioureas (1 eq.) were dissolved in DCM [0.05 M] in the presence of excess of triethylamine (10 eq.), and *N,N′*‐bis‐Boc‐protected pyrazole‐1‐carboxamidine (1.2 eq.), which was used as guanidylating agent, was then added. The reaction was allowed to proceed for 48 h at room temperature under N_2_ atm. The crude product was concentrated in vacuo and purified via flash chromatography (eluent: hexane with a gradient of EtOAc from 0% to 50%). The corresponding Boc‐protected guanidines (1 eq.) were dissolved in a 1:1 TFA:DCM solution [0.05 M]. The reaction was allowed to proceed for 4 h at room temperature under N_2_ atm. The crude product was concentrated in vacuo. The excess of TFA was removed with Et_2_O. Deprotected salts were obtained in quantitative yields.

##### Biochemical Experiments: Cell Culture

All cell cultures were maintained at 37 °C in a humidified incubator with 5% CO_2_. 293T cells (human, female, kidney; ACC‐635, DSMZ; RRID: CVCL_0063) and Vero76 cells (African green monkey, female, kidney; CRL‐1586, ATCC; RRID: CVCL_0574; kindly provided by Andrea Maisner) were cultured in Dulbecco's Modified Eagle Medium (DMEM; PAN‐Biotech) supplemented with 10% fetal bovine serum (FBS; Biochrom) and 1% penicillin–streptomycin (PAN‐Biotech). Calu‐3 cells (human, male, lung; HTB‐55, ATCC; RRID: CVCL_0609; kindly provided by Stephan Ludwig) were maintained in DMEM/F‐12 with Nutrient Mix (Thermo Fisher Scientific), supplemented with 10% FBS, 1% penicillin–streptomycin, 1% nonessential amino acids, and 1 mM sodium pyruvate. Cell line authentication was performed via short tandem repeat (STR) profiling or PCR amplification and sequencing of a cytochrome c oxidase gene fragment, along with assessment of cell morphology and growth behavior. All cell lines were routinely tested for mycoplasma contamination. 293T cells were transfected using a calcium phosphate precipitation method.

##### Biochemical Experiments: Production of Pseudotypes and Inhibition of Viral Entry

Pseudotyped virus particles were produced as previously described.^[^ ^10^ ^]^ In brief, 293T cells were transfected with plasmids encoding S protein or VSV‐G. After 24 h, medium was removed and the cells inoculated with VSV‐G‐trans‐complemented VSV*ΔG (FLuc) (kindly provided by Gert Zimmer^[^ ^66^ ^]^). Following a 1 h incubation at 37 °C, cells were washed, and SARS‐CoV‐2 S protein‐expressing cells were cultured in medium containing anti‐VSV‐G antibody (I1‐hybridoma supernatant; ATCC CRL‐2700). After 16–18 h, supernatants were harvested, clarified by centrifugation (4,000 × g, 10 min), aliquoted, and stored at –80 °C. For infection assays, target cells were seeded in 96‐well plates, incubated with compounds for 120 min, and infected with equal volumes of pseudotyped particles. Following 16–18 h incubation in the presence of compounds, the medium was removed, and cells were lysed in PBS with 0.5% Tergitol 15‐S‐9 (Carl Roth) for 30 min at room temperature. Lysates were transferred to white 96‐well plates, mixed with luciferase substrate (Beetle‐Juice, PJK), incubated for 1 min, and luminescence was measured using a Hidex Sense plate reader (Hidex).

## Supporting Information

Synthetic methods’ details and characterization data for all compounds prepared; UV‐thermal DNA denaturation details; cell viability assays (i.e., AlamarBlue) with MCF10 cell line experimental conditions; biochemical experiments protocols and results; ^1^H NMR and ^13^C NMR spectra; and SwissADME results for compounds **1**–**25**.

## Conflict of Interest

The authors declare no conflict of interest.

## Supporting information

Supplementary Material

## Data Availability

The data that support the findings of this study are included in the Supplemental Information document and available from the corresponding author upon reasonable request.
