# Supplementary material for: Searching for Novel Antiviral Agents as COVID19 Treatments: Guanidino Diaryl Thioureas
Source: ChemMedChem. 2025 Nov 30;21(1):e202501000. doi: 10.1002/cmdc.202501000 (PMC12812011; doi:10.1002/cmdc.202501000)
Supplement: Supplementary file 1 — Supplementary Material [file CMDC-21-e202501000-s001.pdf]

Supplementary Data

Searching for novel antiviral agents as COVID19 treatments: guanidino diaryl thioureas

Marco Minecci,[a] Barbara Farkaš,[a],# Adeyemi Rahman,[a] Amy Kempf,[b] Inga Nehlmeier,[b] Stefan Pöhlmann[b] and Isabel Rozas[a],*

[a]*School of Chemistry, TBSI, Trinity College Dublin, The University of Dublin, 152-160 Pearse Street, Dublin D02 R590, Ireland.* [b]*Infection Biology Unit, German Primate Center, Kellnerweg 4, 37077 Göttingen, Germany*

Table of contents

1. Computational details S2

2. Chemical synthesis S6

   2.1. Materials and Methods S6

   2.2. Synthesis of aniline Boc26A S8

   2.3 Synthesis of *N,N′*-[(4-nitrophenyl)(aryl)]thioureas S10

   2.4. Synthesis of *N,N′*-(bis-Boc-Guanidino)(aryl) thioureas S21

   2.5.Synthesis of trifluoroacetate salts S34

3. UV thermal denaturation experiments S44

4. Cell viability assays S45

5. Biochemical experiments S46

6. ^{1}H NMR and ^{13}C NMR spectra of relevant compounds S61

7. SwissADME results for all compounds modelled S79

1. Computational details

1.1. Docking of the structures in the in-house library (initial screening)

Virtual screening of the guanidine-based derivatives from the in-house library (structures in Table S1) were conducted in the TMPRSS2 homology model developed by Singh et al.[1] using the UniProt sequence (ID O15393) and templating it in SWISS MODEL server.[2]

Ligand preparation: Molecules were geometrically optimised by means of density functional theory (DFT) calculations with the Gaussian16 software[3] using M06-2X functional and 6-31+G(d,p) basis set in implicit water SMD model, and afterwards docked with Autodock Vina 1.2.1 docking suite.[4] Each optimised structure was imported into GaussView where it was converted to pdb format. The pdb structure was imported to Autodock Vina where its torsions were set. The ligand structure was then concerted to pdbqt format which was used for the docking experiment.

Docking: After receptor and ligand preparation, the configuration file was prepared in txt format containing the correct coordinates. The docking experiment was then run through the command terminal using the Autodock Vina 1.2.1 software. Docking was performed allowing flexibility to the ligands and using a rigid target. The parameters used regarding the size of the box were: size_x = 18; size_y = 10; size_z = 20. Upon completion of the calculation, output files were generated containing binding poses which are ranked based on their theoretical binding affinity in the form of Gscores (kcal/mol).

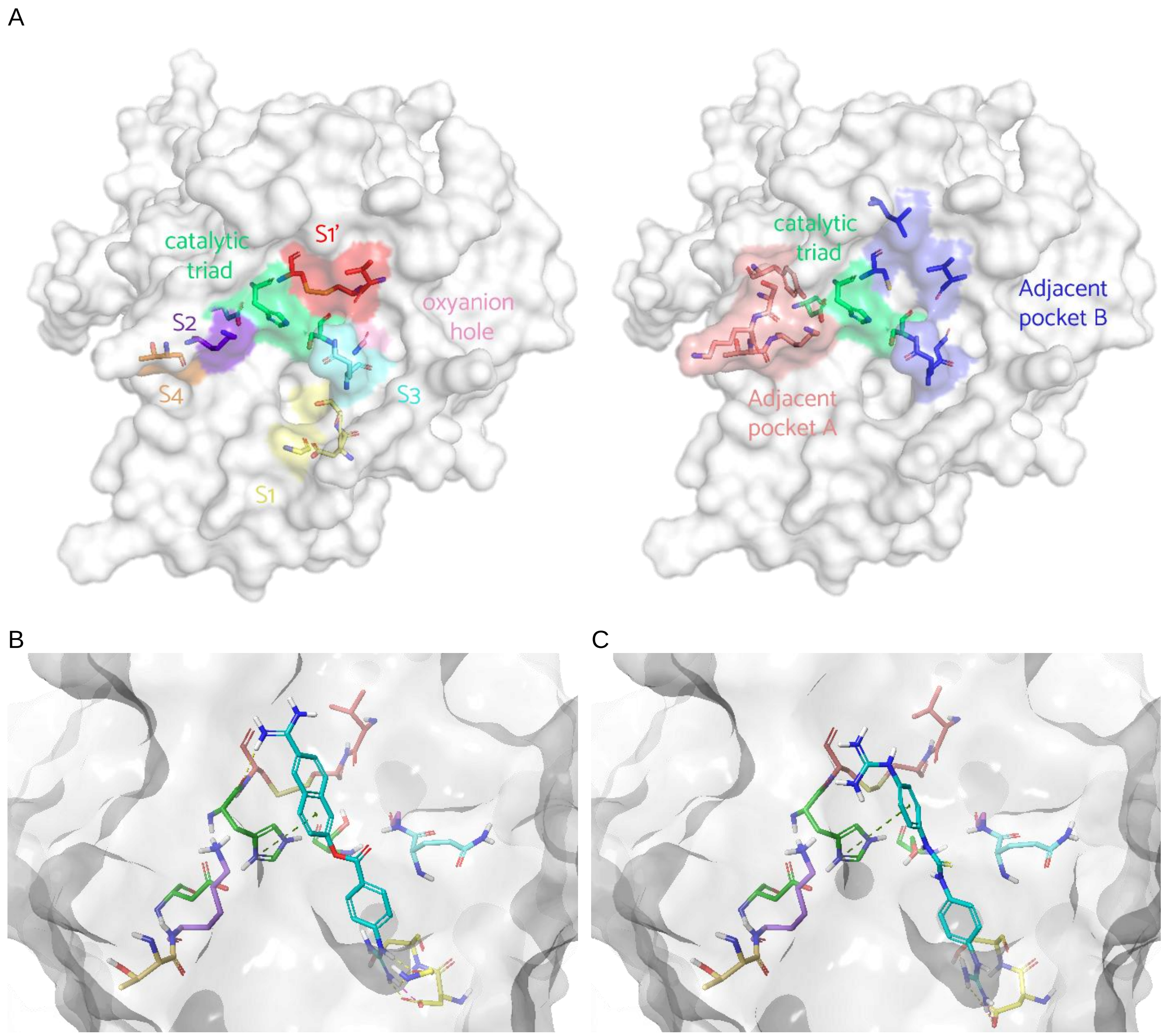

Figure S1. (A) Binding site of TMPRSS2 with highlighted key residues (catalytic triad H296, D345, S441, S1 D435, S436, G464, S2 K342, S3 Q438, S4 T341, S1’ V280, C281, C297, oxyanion hole G439) and sub-pocket areas; (B) Binding pose of nafamostat docked into TMPRSS2. (C) Binding pose of the ‘hit-compound’ from the in-house library docked into TMPRSS2 (hydrogen bonds in dashed yellow, π-π stacking in dashed green, salt bridges in dashed pink).

Table S1. Docking results obtained by Vina-Autodock (with flexible side chains of binding site residues) of some compounds in the in-house library into a homology model of the structure of TMPRSS2.

	Optimised structure	GScore (kcal/mol)		Optimised structure	GScore (kcal/mol)
**Camostat**	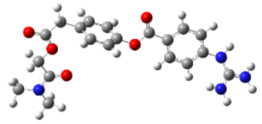	-7.6	**Lib7**	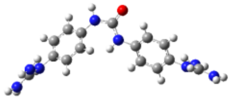	-7.9
**Nafamostat**	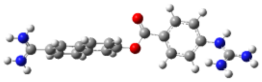	-8.9	**Lib8 (hit-comp)**	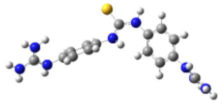	-8.2
**Lib1**	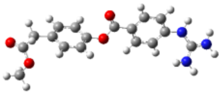	-7.5	**Lib9**	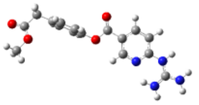	-7.1
**Lib2**	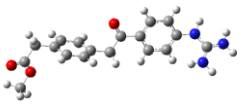	-7.5	**Lib10**	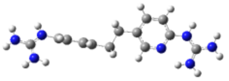	-7.6
**Lib3**	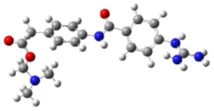	-7.1	**Lib11**	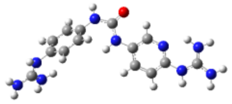	-7.7
**Lib4**	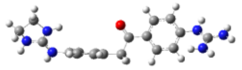	-7.5	**Lib12**	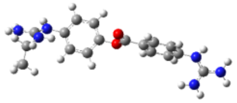	-8.1
**Lib5**	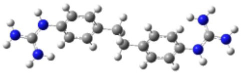	-7.3	**Lib13**	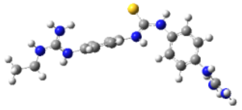	-7.0
**Lib6**	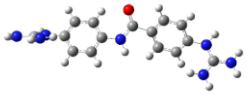	-7.4			

1.2. Docking of the structures of the proposed new analogues of the 'hit-compound' (1)

Compound library was prepared using LigPrep with Epik[5] at physiological conditions (pH 7.0±0.4) to determine the favoured tautomerization and protonation states, while TMPRSS2 was pre-processed via ProteinPreparationWizard[6] as provided in the Schrodinger Suite (version 2020-1), and covalently modified serine residue recovered into its natural state. Molecular docking was performed using both Autodock Vina 1.2.1 docking tool to compare with the initial homology model screen, and Glide[7,8] with the extra precision (XP) scoring function and induced fit docking (IFD)[9] to account for any protein movement in the near vicinity of the bound ligand.

Table S2. Docking results of the hit-compound analogues obtained by Glide XP and IFD in kcal/mol. IFD scores in brackets indicate the best scoring pose for cases where these differed from the hit compound binding pose. Listed interactions correspond to the best scoring IFD binding poses.

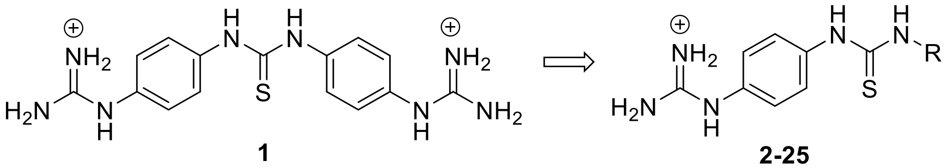

	R	Glide XP	Glide IFD	HBD --- RES	HBA --- RES	π---π	Salt bridge
**Camostat**		-6.1	-8.3	G464, D435, S436	K342	H296	D435
**Nafamostat**		-6.8	-10.1	S436, G464, D435		H296	D435
**1 (Hit-compound)**		-6.5	-10.2	S441, D435, G464, S436, R470, E299		H296	E299, D435
**2**	Ph	-5.9	-8.7	G464, D435, S436, R470, S441			D435
**3**	4(F)Ph	-6.1	(-8.5)	D435, S436, C437, R470			D435, K467
**4**	4(Br)Ph	-5.9	-7.5 (-8.4)	G464, D435, S436, Q438		H296	D435
**5**	4(Me)Ph	-7.4	(-8.8)	G464, D435, S436, S441			D435
**6**	4(^{t}Bu)Ph	-6.2	-8.3 (-9.4)	G464, S436, D435, Q438, G462			D435
**7**	4(NO_2)Ph	-6.8	(-8.2)	G464, D435, S436, S441			D435
**8**	4(NH_2)Ph	-6.4	-8.8 (-9.6)	G464, D435, S436, H296, S460		H296	D435
**9**	4(Et)Ph	-6.0	-7.6 (-9.1)	G464, D435, S436, Q438		H296	D435

**10**	4(CH_2Ph)Ph	-7.1	-8.4 (-9.4)	G462, S436, D435, G464, Q438		W461	D435
**11**	4(OPh)Ph	-6.9	-9.6 (-9.9)	D435, S436, Q438, G462, G464		W461	D435
**12**	4(C≡CH)Ph	-6.4	-8.3 (-8.4)	G464, D435, S436, Q438		H296	D435
**13**	3(OMe)Ph	-6.4	-8.5 (-8.6)	G464, D435, S436, Q438		H296	D435
**14**	4(Me)-3(Me)Ph	-5.8	(-8.9)	D435, S436, G464, S441		H296	D435
**15**	4(Br)-3(Me)Ph	-6.5	-7.4 (-9.0)	G464, D435, S436, S460		H296	D435
**16**	4(Cl)-3(CF_3)Ph	-6.9	-7.0 (-8.4)	G464, D435, S436, Q438, G462			D435
**17**	4(OMe)-3(OMe)Ph	-7.0	-7.9 (-9.0)	S436, D435, G464, G462, Q438			D435
**18**	3(CF_3)-3'(CF_3)Ph	-7.1	-9.5	G464, D435, S436, S441			D435
**19**	Benzo-cyclohexyl	-6.8	-7.3 (-9.7)	G464, D435, S436, G462			D435
**20**	Pyr	-7.7	-7.4 (-8.5)	G464, D435, S436	G439, S441	H296	D435
**21**	2(Me)-(2-Pyr)	-7.0	-7.3 (-8.7)	G464, S436, D435, Q438		H296	D435
**22**	4(Me)-(2-Pyr)	-8.8	-7.3 (-8.1)	G464, D435, S436, G462			D435
**23**	3(Me)-(2-Pyr)	-6.9	-8.5 (-9.4)	S460, G464, S436, D435	S441	H296	D435
**24**	4(Cl)-(2-Pyr)	-7.6	-8.5 (-8.6)	G464, D435, S436	G439	H296	D435
**25**	Benzimid-azole	-6.7	-9.1	D435, S436, G464, S441		H296	D435

Nafamostat **Hit-compound (1)**

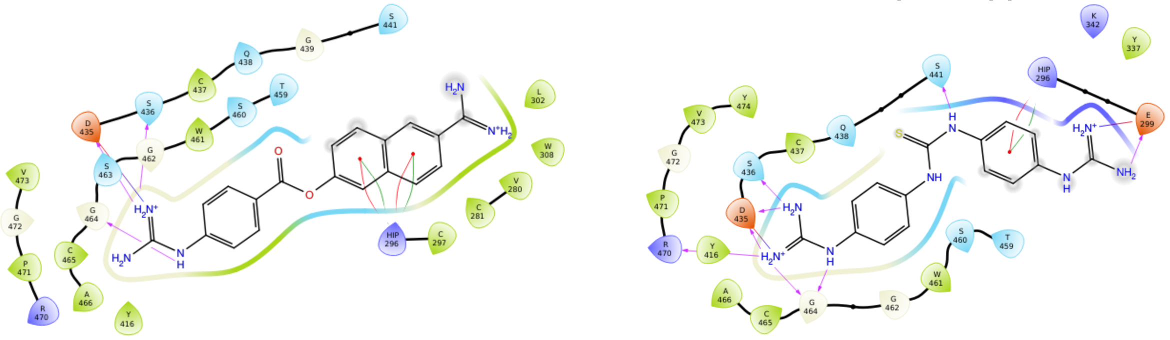

Figure S2. Interaction diagrams for the IFD docking of nafamostat and the 'hit-compound' (**1**) in TMPRSS2 crystal structure (PDBID: 7MEQ).

1.3. Molecular Dynamics protocol

Established protein-ligand interactions, stability, and affinity were thoroughly investigated by performing MD simulations and analysis of the root mean square deviation (RMSD) of both ligand and protein, root mean square fluctuation (RMSF) on per residue level, protein-ligand contact analysis, and molecular mechanics/generalized Born surface area (MM/GBSA) calculations were performed via Desmond MD software.[65] Standard MD protocol was employed, including the initial minimization using the conjugate gradient algorithm, short 10 ns NVT equilibration including the gradual increase in system temperature from 10K to 300K with restraints on heavy atoms to allow solvent relaxation, a 5 ns NPT equilibration with heavy atom restrains followed by another 5 ns with released restraints, and finally a 200 ns NPT production stage with trajectory output saved at 100 ps intervals, where the binding free energies were assessed over the final 100 ns (1000 frames) using the thermal MM/GBSA approach. For NVT and NPT stages, Nose-Hoover chain thermostat and isotropic Martyna-Tobias-Klein barostat were used, with relaxation times of 1.0 and 2.0 ps, respectively.

2. Chemical Synthesis

2.1. Materials and Methods

All commercial chemicals were obtained from either Sigma-Aldrich, Fischer Scientific or Fluorochem and used without further purification. Deuterated solvents for NMR analysis were purchased from Fischer Scientific. Column chromatography was performed using Sigma-Aldrich silica gel 100-200 mesh. Solvents for synthesis purposes were used at HPLC grade. Analytical TLC was performed using Merck Kieselgel 60 F-254 silica gel plates. Visualisation was completed using a UV light (254 nm). NMR spectra were recorded on Bruker DPX-400 Advance and Bruker AV-600 spectrometers, operating a either 400.13 and 600.1 MHz for ^{1}H NMR; 100.6 and 150.9 MHz for ^{13}C NMR. Shifts are reported as referenced to the internal solvent signal. NMR data was processed using TopSpin, Bruker's NMR data analysis software. HRMS spectra were measured on a Micromass LCT electrospray TOF instrument with a WATERS 2690 autosampler and methanol/acetonitrile as carrier solvent.

Method A – Synthesis of *N,N'*-[(p-Nitrophenyl)(Aryl)]Thioureas

p-Nitrophenyl isothiocyanate (1 eq.) was reacted with the corresponding aniline **2A-7A/Boc8A/9A-25A/Boc26A** (1 eq.) in either cyrene or dry THF [0.5 M] under an N_2 atm. The reaction was allowed to proceed for 1-2 hours at room temperature, monitored by TLC (Hex:EtOAc, 7:3). Upon completion, the reaction was concentrated *in vacuo* affording the corresponding *N,N'*-(p-nitro)(aryl) thioureas in quantitative fashion.

Method B – Reduction of *N,N'*-[(4-Nitrophenyl)(Aryl)]Thioureas to *N,N'*-[(p-Aminophenyl)(Aryl)] Thioureas

N,N'-(p-Nitro)(aryl) thioureas **2B-7B/Boc8B/9B-25B/Boc26B** (1 eq.) were reacted with zinc powder (6 eq.) in an 2:2:1 EtOH:AcOH:H_2O [0.1] M solution under an N_2 environment. The reaction was allowed to proceed for 1-2 hours at room temperature, monitored by TLC (Hex:EtOAc, 1:1). Zinc dust in excess was filtered via syringe using a PTFE syringe filter (0.45 µm) and the solvent mixture was removed at low pressure. Traces of water and acetic acid were removed *in vacuo*. The obtained amines were directly used in the next step without further purification.

Method C – Guanidylation of *N,N′*-[(4-Aminophenyl)(Aryl)]Thioureas

The corresponding *N,N′*-(p-amino)(aryl) thioureas **2C-6C/Boc8C/9C-25C/Boc26C** (1 eq.) were dissolved in DCM [0.05] M in the presence of excess of triethylamine (10 eq.) and the *N,N′*-bis-Boc protected pyrazole-1-carboxamidine (1.2 eq.), which was used as guanidylating agent, was then added. The reaction was allowed to proceed for 48 h at room temperature under N_2 atm. The crude product was concentrated in vacuo and purified *via* flash chromatography (Eluent: Hexane with a gradient of EtOAc from 0% to 50%).

Method D – Deprotection of *N,N′*-[(4-Boc-Guanidinophenyl)(Aryl)]Thioureas

Compounds **2D-6D/Boc-8D/9D-25D/Boc26D** (1 eq.) were dissolved in a 1:1 TFA:DCM solution [0.05] M. The reaction was allowed to proceed for 4 h at room temperature under N_2 atm. The crude product was concentrated in vacuo. The excess of TFA was removed with Et_2O. Deprotected salts were obtained in quantitative yields (>98%).

2.2. Synthesis of aniline Boc26A

***tert*-Butyl-(2-azidoethyl)carbamate, 27.**[10]

Commercially available *N*-Boc-ethylenediamine (800 mg, 5 mmol, 1eq.) was dissolved in 50 mL of MeOH (0.1 M) and cooled at 0 °C. A K_2CO_3 aqueous solution 0.5 M (50 mL, 25 mmol, 5 eq.) was added. After 15 minutes, diazo transfer compound 1-(azidosulfonyl)-1H-imidazol-3-ium hydrogen sulfate (1.6 g, 6 mmol, 1.2 eq.), $CuSO_4{\cdot}5H_2O$ solution 0.1 M (500 µL, 0.05 mmol, 0.01 eq.). The reaction was stirred at room temperature overnight. Upon completion, most of the methanol was evaporated at reduced pressure. The resulting crude was dissolved in 200 mL of CH_2Cl_2 and washed with HCl 1 M (2 x 100 mL). The organic phase was then dried over Na_2SO_4 and concentrated under vacuum. Product **27** (colourless liquid) was obtained in a 71% yield and directly used in the next step.[1]

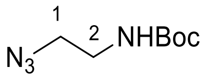

^{1}H NMR (400 MHz, DMSO-d$_6$) δ 7.02 (s, 1H, N<u>H</u>Boc), 3.27 (t, 2H, H_1, J_{1-2} = 5.7 Hz), 3.09 (q, 2H, H_2, J_{1-2} = J_{2-NH} = 5.7 Hz), 70.2 (s, 9H, C(C<u>H</u>$_3$)$_3$).

HRMS (m/z ESI$^+$): m/z found 187.1194 $[M+1]^+$; $C_7H_{15}N_4O_2$ requires 187.1190

***tert*-Butyl (2-(4-(4-aminophenyl)-1H-1,2,3-triazol-1-yl)ethyl)carbamate, Boc26A**

Aniline **12A** (117 mg, 1 mmol, 1 eq) and *tert*-butyl (2-azidoethyl)carbamate (**27**) (186 mg, 1 mmol, 1 eq) were dissolved in 4 mL of degassed *t*BuOH. 500 µL of a 0.08 M solution of copper (II) sulphate (0.2 mmol, 0.2 eq) and 500 µL of a 0.16 M solution of sodium ascorbate (0.4 mmol, 0.4 eq) were added. The reaction was stirred for 24 h at room temperature. Upon completion, the reaction was diluted with 50 mL of EtOAc and washed with a saturated NH_4Cl sol. (1 x 30 mL), water (2 x 50 mL) and brine (1 x 30 mL). The organic phase was then dried over Na_2SO_4 and concentrated under vacuum. Product **Boc26A** was then purified via flash chromatography (Eluent: Hexane with a gradient of EtOAc from 0% to 50%). The compound was obtained in 82% yield.

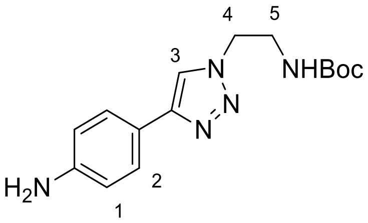

^{1}H NMR (400 MHz, DMSO-d$_6$) δ 8.15 (s, 1H, H_3), 7.44 (d, 2H, H_2, $J_{1\text{-}2}$ = 8.4 Hz), 6.98 (s, 2H, N$\underline{H}$Boc), 6.57 (d, 2H, H_1, $J_{1\text{-}2}$ = 8.4 Hz), 5.17 (s, 2H, N$\underline{H}_2$), 4.34 (d, 2H, H_4, $J_{4\text{-}5}$ = 6.0 Hz), 3.36 (q, 2H, H_5, $J_{4\text{-}5}$ = 6.0 Hz), 1.32 (s, 9H, H_{16}).

HRMS (m/z ESI$^+$): m/z found 304.1771 $[M+1]^+$; $C_{15}H_{22}N_5O_2$ requires 304.1768

2.3. Synthesis and characterisation of *N,N'*-[(4-nitrophenyl)(aryl)]thioureas

All of them were prepared according to **Method A** utilising the corresponding anilines **2A-7A/Boc8A/9A-Boc26A**. Compounds **2B-6B**, **Boc8B-15B**, **18B-21B**, **24B-25B** have all been reported already as described in reference 11.

1-(4-Nitrophenyl)-3-phenylthiourea, 2B.[11]

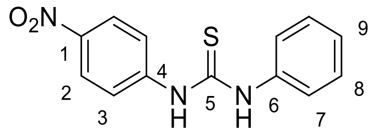

Yield: 90%

^{1}H NMR (600 MHz, DMSO-d$_6$) δ 10.37 (s, 1H, NO_2PhN<u>H</u>), 10.28 (s, 1H, PhN<u>H</u>), 8.21 (d, 2H, H_2, J_{2-3} = 9.1 Hz), 7.97 (d, 2H, H_3, J_{2-3} = 9.1 Hz), 7.52 (d, 2H, H_7, J_{7-8} = 8.7 Hz), 7.37 (t, 1H, H_9, J_{9-8} = 7.4 Hz), 7.18 (t, 2H, H_8, J_{7-8} = 8.7 Hz, J_{9-8} = 7.4 Hz).

^{13}C NMR (150 MHz, DMSO-d$_6$) δ 179.8 (C_5), 146.7 (C_4), 142.8 (C_1), 139.6 (C_9), 125.6 (C_9), 124.7 (C_2), 124.2 (C_8), 123.1 (C_7), 122.2 (C_3).

HRMS (m/z ESI$^+$): m/z found 274.0602 $[M+1]^+$; $C_{13}H_{12}N_3O_2S$ requires 274.0606

1-(4-Nitrophenyl)-3-(4-fluorophenyl) thiourea, 3B.[11]

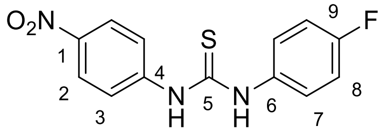

Yield: 75%

^{1}H NMR (600 MHz, DMSO-d$_6$) δ 10.38 (s, 1H, NO_2PhN<u>H</u>), 10.22 (s, 1H, F-PhN<u>H</u>), 8.21 (d, 2H, H_2, J_{2-3} = 9.6 Hz), 7.84 (d, 2H, H_3, J_{2-3} = 9.6 Hz), 7.50 (dd, 2H, H_7, J_{7-8} = 8.7 Hz, J_{7-F} = 4.8 Hz), 7.22 (t, 2H, H_8, J_{8-F} = 8.7 Hz).

^{13}C NMR (150 MHz, DMSO-d$_6$) δ 180.2 (C_5), 159.4 (d, C_9, J_{9-F} = 242 Hz), 146.8 (C_4), 142.7 (C_1), 137.7 (d, C_8, J_{8-F} = 2.7 Hz), 126.7 (d, C_7, J_{7-F} = 5.8 Hz), 124.9 (C_2), 124.7, (C_7), 122.2 (C_3).

HRMS (m/z ESI$^+$): m/z found 292.0060 $[M+1]^+$; $C_{13}H_{11}FN_3O_2S$ requires 292.0056

1-(4-Nitrophenyl)-3-(4-bromophenyl)thiourea, 4B.[11]

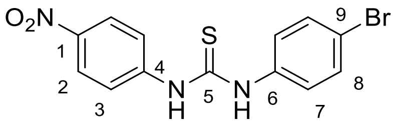

Yield: 90%

^{1}H NMR (600 MHz, DMSO-d$_6$) δ 10.45 (s, 1H, $NO_2PhN\underline{H}$), 10.33 (s, 1H, $BrPhN\underline{H}$), 8.22 (d, 2H, H_2, $J_{2\text{-}3}$ = 9.2 Hz), 7.84 (d, 2H, H_3, $J_{2\text{-}3}$ = 9.2 Hz), 7.56 (d, 2H, H_7, $J_{7\text{-}8}$ = 8.7 Hz), 7.49 (d, 2H, H_8, $J_{7\text{-}8}$ = 8.7 Hz).

^{13}C NMR (150 MHz, DMSO-d$_6$) δ 179.8 (C_5), 146.5 (C_4), 142.9 (C_1), 138.9 (C_6), 131.9 (C_8), 126.1 (C_7), 124.9 (C_2), 122.2 (C_1), 116.3 (C_9).

HRMS (m/z ESI$^+$): m/z found 351.9748 $[M+1]^+$; $C_{13}H_{11}BrN_3O_2S$ requires 351.9755

1-(4-Nitrophenyl)-3-(4-methylphenyl)thiourea 5B.[11]

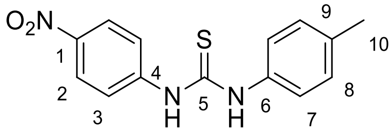

Yield: 81%

^{1}H NMR (600 MHz, DMSO-d$_6$) δ 10.31 (s, 1H, $NO_2PhN\underline{H}$), 10.21 (s, 1H, $MePhN\underline{H}$), 8.20 (d, 2H, H_2, $J_{2\text{-}3}$ = 8.9 Hz), 7.85 (d, 2H, H_3), 7.37 (d, 2H, H_7, $J_{7\text{-}8}$ = 8.1 Hz), 7.17 (d, 2H, H_8), 2.30 (s, 3H, H_{10}).

^{13}C NMR (150 MHz, DMSO-d$_6$) δ 179.8 (C_5), 146.9 (C_4), 142.6 (C_1), 136.8 (C_9), 134.8 (C_6), 129.6 (C_8), 124.8 (C_3), 124.3 (C_7), 121.9 (C_2), 21.0 (C_{10}).

HRMS (m/z ESI$^+$): m/z found 287.0807 $[M+1]^+$; $C_{14}H_{14}N_3O_2S$ requires 287.0806

1-(4-Nitrophenyl)-3-[4-(*tert*-butyl)phenyl]thiourea, 6B.[11]

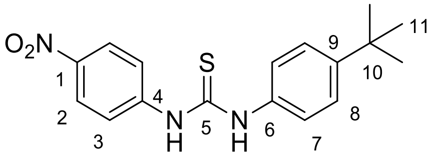

Yield: 64%

^{1}H NMR (600 MHz, DMSO-d$_6$) δ 10.32 (s, 1H, $NO_2PhN\underline{H}$), 10.20 (s, 1H, $tBuN\underline{H}$), 8.20 (d, 2H, H_2, $J_{2\text{-}3}$ = 9.6 Hz), 7.84 (d, 2H, H_3, $J_{2\text{-}3}$ = 9.6 Hz), 7.41 - 7.39 (m, 4H, H_7, H_8), 1.29 (s, 9H, $ArC(C\underline{H}_3)_3$).

^{13}C NMR (150 MHz, DMSO-d$_6$) δ 179.7 (C_5), 147.9 (C_4), 146.9 (C_1), 142.7 (C_6), 136.8 (C_9), 127.7 (C_7), 125.8 (C_2), 123.9 (C_8), 121.9 (C_3), 34.7 (Ar$\underline{C}$(CH_3)$_3$), 31.6 (ArC($\underline{C}H_3$)$_3$).

HRMS (m/z ESI$^+$): m/z found 330.1281 [M+1]$^+$; $C_{17}H_{20}N_3O_2S$ requires 330.1276

1,3-bis(4-nitrophenyl)thiourea, 7B.[12]

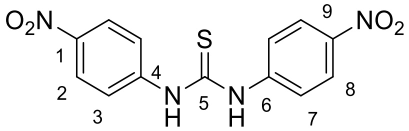

Yield: 81%

^{1}H NMR (600 MHz, DMSO-d$_6$) δ 10.79 (s, 2H, NO_2PhN$\underline{H}$), 8.25 (d, 4H, H_2, J_{2-3} = 9.1 Hz), 7.86 (d, 4H, H_3, J_{2-3} = 9.1 Hz).

^{13}C NMR (150 MHz, DMSO-d$_6$) δ 179.7 (C_5), 146.1 (C_4), 143.2 (C_1), 124.9 (C_2), 122.6 (C_3).

HRMS (m/z ESI$^+$): m/z found 319.0502 [M+1]$^+$; $C_{13}H_{11}N_4O_4S$ requires 319.0496

1-(4-Nitrophenyl)-3-[4-(*tert*-butoxycarbonyl)aminophenyl]thiourea, Boc8B.[11]

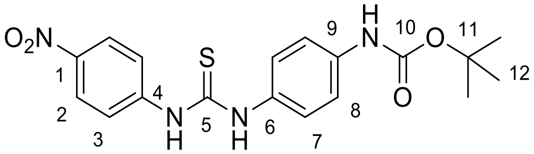

Yield: 80%

^{1}H NMR (600 MHz, DMSO-d$_6$) δ 10.23 (s, 1H, NO_2PhN$\underline{H}$), 10.12 (s, 1H, BocNHPhN$\underline{H}$), 9.37 (s, 1H, BocN$\underline{H}$PhNH), 8.21 (d, 2H, H_2, J_{2-3} = 9.2 Hz), 7.83 (d, 2H, H_3, J_{2-3} = 9.2 Hz), 7.43 (d, 2H, H_7, J_{7-8} = 9.0 Hz), 7.33 (d, 2H, H_8, J_{7-8} = 9.0 Hz), 1.48 (s, 3H, H_{12}).

^{13}C NMR (150 MHz, DMSO-d$_6$) δ 179.8 (C_5), 152.5 (C_{10}), 146.3 (C_4), 141.8 (C_1), 140.9 (C_6), 135.8 (C_9), 126.8 (C_2), 124.3 (C_8), 123.8 (C_3), 122.3 (C_7), 79.4 (C_{11}), 28.4 (C_{12}).

HRMS (m/z ESI$^+$): m/z found 398.1277 [M+1]$^+$; $C_{18}H_{21}N_4O_4S$ requires 389.1283

1-(4-Nitrophenyl)-3-(4-ethylphenyl)thiourea, 9B.[11]

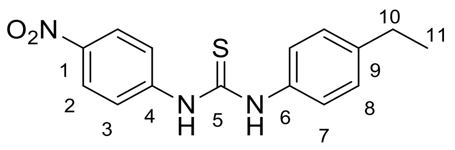

Yield: 96%

^{1}H NMR (400 MHz, DMSO-d_6) δ 10.30 (s, 1H, $NO_2PhN\underline{H}$), 10.20 (s, 1H, $EtPhN\underline{H}$), 8.20 (d, 2H, H_2, J_{2-3} = 9.3 Hz), 7.85 (d, 2H, H_3, J_{2-3} = 9.3 Hz), 7.39 (d, 2H, H_8, J_{8-7} = 8.3 Hz), 7.21 (d, 2H, H_7), 2.60 (q, 2H, H_{10}, J_{10-11} = 7.6 Hz), 1.18 (t, 3H, H_{11}, J_{10-11} = 7.6 Hz).

^{13}C NMR (100 MHz, DMSO-d_6) δ 179.6 (C_5), 149.8 (C_4), 146.7 (C_1), 142.8 (C_9), 140.5 (C_6), 129.9 (C_8), 124.9 (C_3), 122.1 (C_2), 120.3 (C_8), 118.7 (C_7), 28.5 (C_{10}), 14.5 (C_{11}).

HRMS (m/z ESI$^+$): m/z found 304.0721 $[M+1]^+$; $C_{14}H_{14}N_3O_3S$ requires 304.0711

1-(4-Nitrophenyl)-3-(4-benzylphenyl)thiourea, 10B.[11]

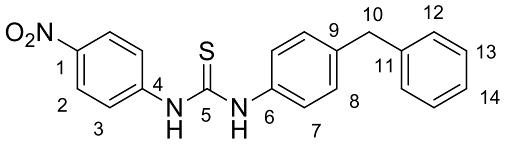

Yield: 94%

^{1}H NMR (600 MHz, DMSO-d_6) δ 10.32 (s, 1H, $NO_2PhN\underline{H}$), 10.20 (s, 1H, $BnPhN\underline{H}$), 8.19 (d, 2H, H_2, J_{2-3} = 9.2 Hz), 7.82 (d, 2H, H_3, J_{2-3} = 9.2 Hz), 7.38 (d, 2H, H_7, J_{7-8} = 8.4 Hz), 7.29 (t, 2H, H_{13}, J_{13-12} = J_{13-14} = 7.8 Hz), 7.27 - 7.17 (m, 5H, H_8, H_{12}, H_{14}), 3.93 (s, 3H, H_{10}).

^{13}C NMR (150 MHz, DMSO-d_6) δ 179.9 (C_5), 146.8 (C_4), 142.7 (C_1), 141.7 (C_{11}), 138.6 (C_6), 137.3 (C_9), 129.3 (C_{13}), 129.2 (C_8), 128.9 (C_{12}), 126.4 (C_{14}), 124.8 (C_2), 124.3 (C_7), 121.9 (C_3), 41.2 (C_{10}).

HRMS (m/z ESI$^+$): m/z found 364.1101 $[M+1]^+$; $C_{20}H_{17}N_3O_2S$ requires 364.1119

1-(4-Nitrophenyl)-3-(4-phenoxyphenyl) thiourea, 11B.[11]

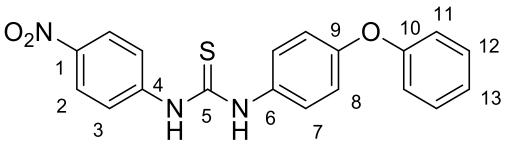

Yield: 71%

^{1}H NMR (600 MHz, DMSO-d$_6$) δ 10.36 (s, 1H, NO$_2$PhN<u>H</u>), 10.26 (s, 1H, PhOPhN<u>H</u>), 8.21 (d, 2H, H$_2$, J$_{2-3}$ = 9.2 Hz), 7.83 (d, 2H, H$_3$, J$_{2-3}$ = 9.2 Hz), 7.44 (d, 2H, H$_7$, J$_{7-8}$ = 8.8 Hz), 7.40 (dd, 2H, H$_{12}$, J$_{12-11}$ = 8.5 Hz, J$_{12-13}$ = 7.4 Hz), 7.15 (tt, 1H, H$_{13}$, J$_{12-13}$ = 7.4 Hz, J$_{13-11}$ = 0.9 Hz), 7.05 - 6.99 (m, 4H, H$_8$, H$_{11}$).

^{13}C NMR (150 MHz, DMSO-d$_6$) δ 179.9 (C$_5$), 157.2 (C$_{10}$), 154.2 (C$_9$), 146.9 (C$_4$), 142.6 (C$_1$), 134.9 (C$_6$), 130.5 (C$_{12}$), 126.3 (C$_7$), 124.9 (C$_2$), 123.1 (C$_{13}$), 122.1 (C$_3$), 119.3 (C$_8$), 118.9 (C$_{11}$).

HRMS (m/z ESI$^+$): m/z found 366.0640 [M+1]$^+$; $C_{19}H_{16}N_3O_3S$ requires 366.0834

1-(4-Nitrophenyl)-3-(4-ethynylphenyl)thiourea, 12B.[11]

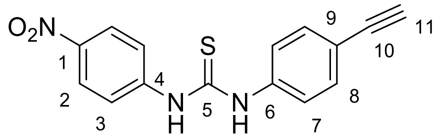

Yield: 98%

^{1}H NMR (600 MHz, DMSO-d$_6$) δ 10.48 (s, 1H, NO$_2$PhN<u>H</u>), 10.40 (s, 1H, HC≡CPhN<u>H</u>), 8.23 (d, 2H, H$_2$, J$_{2-3}$ = 9.2 Hz), 7.85 (d, 2H, H$_3$, J$_{2-3}$ = 9.2 Hz), 7.37 (d, 2H, H$_7$, J$_{7-8}$ = 8.8 Hz), 7.17 (2, 1H, H$_8$, J$_{7-8}$ = 8.8 Hz), 4.15 (s, 3H, H$_{11}$).

^{13}C NMR (150 MHz, DMSO-d$_6$) δ 179.5 (C$_5$), 146.5 (C$_4$), 143.0 (C$_1$), 140.0 (C$_6$), 132.6 (C$_8$), 132.5 (C$_2$), 126.9 (C$_1$), 124.9 (C$_7$), 123.6 (C$_3$), 123.4 (C$_9$), 83.2 (C$_{10}$), 81.0 (C$_{11}$).

HRMS (m/z ESI$^+$): m/z found 298.0657 [M+1]$^+$; $C_{15}H_{12}N_3O_2S$ requires 298.0650

1-(4-Nitrophenyl)-3-(3-methoxyphenyl) thiourea, 13B.[11]

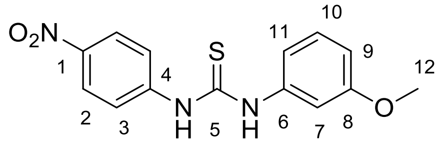

Yield: 98%

^{1}H NMR (400 MHz, DMSO-d$_6$) δ 10.39 (s, 1H, NO$_2$PhN<u>H</u>), 10.28 (s, 1H, mOMePhN<u>H</u>), 8.21 (d, 2H, H$_2$, J$_{2-3}$ = 9.2 Hz), 7.83 (d, 2H, H$_3$, J$_{2-3}$ = 9.2 Hz), 7.28 (t, 1H, H$_{10}$, J$_{10-11}$ = J$_{10-9}$ = 8.3 Hz), 7.17 (s, 1H, H$_7$), 7.05 (d, 1H, H$_{11}$, J$_{10-11}$ = 8.3 Hz,), 6.76 (d, 1H, H$_9$, J$_{10-9}$ = 8.3 Hz,), 3.75 (s, 3H, H$_{12}$).

^{13}C NMR (100 MHz, DMSO-d$_6$) δ 179.6 (C$_5$), 159.8 (C$_8$), 146.7 (C$_4$), 142.8 (C$_1$), 140.5 (C$_6$), 129.9 (C$_{10}$), 124.9 (C$_2$), 122.1 (C$_3$), 116.1 (C$_{11}$), 110.8 (C$_9$), 109.8 (C$_7$), 55.6 (C$_{12}$).

HRMS (m/z ESI$^+$): m/z found 304.0721 [M+1]$^+$; $C_{14}H_{14}N_3O_3S$ requires 304.0711

1-(4-Nitrophenyl)-3-(3,4-dimethylphenyl)thiourea 14B.[11]

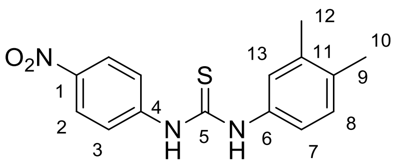

Yield: 80%

^{1}H NMR (600 MHz, DMSO-d$_6$) δ 10.26 (s, 1H, NO_2PhN<u>H</u>), 10.15 (s, 1H, XyN<u>H</u>), 8.20 (d, 2H, H_2, $J_{2\text{-}3}$ = 9.3 Hz), 7.83 (d, 2H, H_3, $J_{2\text{-}3}$ = 9.3 Hz), 7.22 (d, 1H, H_{13}, $J_{13\text{-}7}$ = 1.7 Hz), 7.18 (dd, 1H, H_7, $J_{7\text{-}8}$ = 8.1 Hz, $J_{13\text{-}7}$ = 1.7 Hz), 7.12 (d, 1H, H_8, $J_{7\text{-}8}$ = 8.1 Hz), 2.21 (s, 3H, H_{12}), 2.20 (s, 3H, H_{10}).

^{13}C NMR (150 MHz, DMSO-d$_6$) δ 179.7 (C_5), 146.9 (C_4), 143.3 (C_1), 136.9 (C_9), 136.8 (C_{11}), 133.7 (C_6), 130.0 (C_8), 125.5 (C_{13}), 124.8 (C_2), 122.0 (C_3), 20.0 (C_{10}), 19.4 (C_{12}).

HRMS (m/z ESI$^+$): m/z found 302.0967 $[M+1]^+$; $C_{15}H_{16}N_3O_2S$ requires 302.0963

1-(4-Nitrophenyl)-3-(4-bromo-3-methylphenyl)thiourea, 15B.[11]

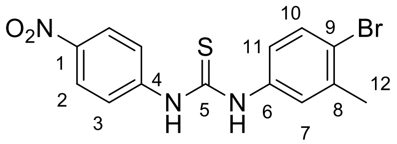

Yield: 91%

^{1}H NMR (600 MHz, DMSO-d$_6$) δ 10.42 (s, 1H, NO_2PhN<u>H</u>), 10.28 (s, 1H, ArN<u>H</u>), 8.21 (d, 2H, H_2, $J_{2\text{-}3}$ = 9.4 Hz), 7.83 (d, 2H, H_3, $J_{2\text{-}3}$ = 9.4 Hz), 7.56 (d, 1H, H_{10}, $J_{10\text{-}11}$ = 8.8 Hz), 7.47 (d, 1H, H_7, $J_{7\text{-}11}$ = 2.0 Hz), 7.29 (dd, 1H, H_{11}, $J_{10\text{-}11}$ = 8.8 Hz, $J_{7\text{-}11}$ = 2.0 Hz), 2.34 (s, 3H, H_{12}).

^{13}C NMR (150 MHz, DMSO-d$_6$) δ 179.8 (C_5), 146.6 (C_4), 142.9 (C_1), 138.9 (C_8), 137.9 (C_6), 132.6 (C_{10}), 126.6 (C_7), 124.9 (C_2), 123.7 (C_{11}), 122.2 (C_3), 120.2 (C_9), 23.0 (C_{12}).

HRMS (m/z ESI$^+$): m/z found 366.9908 $[M+1]^+$; $C_{14}H_{13}BrN_3O_2S$ requires 366.9912

1-(4-Nitrophenyl)-3-(3-trifluoromethyl-4-chloropheny) thiourea, 16B

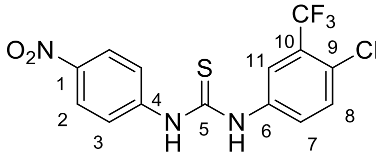

Yield: 64%

^{1}H NMR (600 MHz, DMSO-d$_6$) δ 10.63 (s, 1H, NO$_2$PhN$\underline{H}$), 10.48 (s, 1H, ArN$\underline{H}$), 8.23 (d, 2H, H$_2$, J$_{2-3}$ = 9.2 Hz), 8.08 (d, 1H, H$_{11}$, J$_{11-7}$ = 2.5 Hz), 7.83-7.79 (m, 3H, H$_3$, H$_7$).

^{13}C NMR (150 MHz, DMSO-d$_6$) δ 180.1 (C$_5$), 146.1 (C$_4$), 143.2 (C$_1$), 139.1 (C$_6$), 132.3 (C$_8$), 129.1 (C$_7$), 126.9 (C$_{10}$), 125.0 (C$_2$), 123.1 (q, $\underline{C}$F3, J$_{C-F}$ = 272.0 Hz), 123.0 (q, C$_{11}$, J$_{11-F}$ = 5.5 Hz), 122.6 (C$_3$), 112.8 (C$_9$).

HRMS (m/z ESI$^+$): m/z found 376.0112 [M+1]$^+$; $C_{14}H_{10}ClF_3N_3O_2S$ requires 376.0100

1-(4-Nitrophenyl)-3-[3,4-dimethoxyphenyl]thiourea, 17B

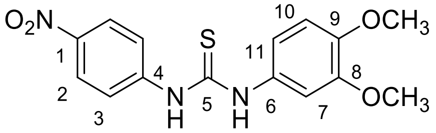

Yield: 70%

^{1}H NMR (600 MHz, DMSO-d$_6$) δ 10.21 (s, 1H, NO$_2$PhN$\underline{H}$), 10.12 (s, 1H, ArN$\underline{H}$), 8.20 (d, 2H, H$_2$, J$_{2-3}$ = 9.3 Hz), 7.84 (d, 2H, H$_3$, J$_{2-3}$ = 9.3 Hz), 7.13 (s, 1H, H$_7$), 6.96 (s, 2H, H$_{10}$, H$_{11}$), 3.76 (s, 3H, O$\underline{C}$H$_3$), 3.74 (s, 3H, O$\underline{C}$H$_3$).

^{13}C NMR (150 MHz, DMSO-d$_6$) δ 179.7 (C$_5$), 148.9 (C$_9$), 147.0 (C$_4$), 146.9 (C$_1$), 142.7 (C$_8$), 132.3 (C$_6$), 124.8 (C$_2$), 122.1 (C$_3$), 116.9 (C$_{11}$), 112.2 (C$_{10}$), 109.6 (7$_1$), 56.2 (O$\underline{C}$H$_3$), 56.0 (O$\underline{C}$H$_3$).

HRMS (m/z ESI$^+$): m/z found 334.0857 [M+1]$^+$; $C_{15}H_{16}N_3O_4S$ requires 334.0857

1-(4-Nitrophenyl)-3-(3,5-bis(trifluoromethyl)phenyl)thiourea, 18B.[11]

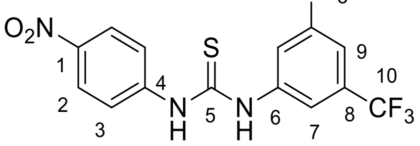

Yield: 75%

^{1}H NMR (600 MHz, DMSO-d$_6$) δ 10.81 (s, 1H, NO$_2$PhN$\underline{H}$), 10.65 (s, 1H, ArN$\underline{H}$), 8.26 (s, 2H, H$_7$), 8.25 (d, 2H, H$_2$, J$_{2-3}$ = 9.2 Hz), 7.87 (s, 1H, H$_9$), 7.82 (d, 2H, H$_3$, J$_{2-3}$ = 9.2 Hz).

^{13}C NMR (150 MHz, DMSO-d$_6$) δ 180.4 (C$_5$), 145.9 (C$_4$), 143.5 (C$_1$), 141.8 (C$_{11}$), 130.4 (C$_6$), 126.9 (C$_{10}$), 125.0 (C$_2$), 124.2 (C$_7$), 122.9 (C$_3$), 118.1 (C9).

HRMS (m/z ESI⁺): m/z found 410.0355 $[M+1]^+$; $C_{15}H_{10}F_6N_3O_2S$ requires 410.0353

1-(4-Nitrophenyl)-3-(5,6,7,8-tetrahydronaphthalen-2-yl)thiourea, 19B.[11]

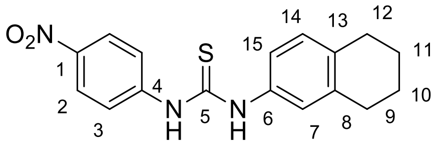

Yield: 80%

^{1}H NMR (600 MHz, DMSO-d$_6$) δ 10.25 (s, 1H, $NO_2PhN\underline{H}$), 10.13 (s, 1H, $ArN\underline{H}$), 8.19 (d, 2H, H_2, $J_{2\text{-}3}$ = 9.1 Hz), 7.83 (d, 2H, H_3, $J_{2\text{-}3}$ = 9.1 Hz), 7.17 (dd, 1H, H_{15}, $J_{15\text{-}14}$ = 8.3 Hz, $J_{15\text{-}7}$ = 2.0 Hz), 7.14 (m, 1H, H_4), 7.04 (d, 1H, H_{14}, $J_{15\text{-}14}$ = 8.3 Hz), 2.69 (m, 4H, H_9, H_{12}), 2.69 (m, 4H, H_{10}, H_{11}).

^{13}C NMR (150 MHz, DMSO-d$_6$) δ 179.7 (C_5), 146.9 (C_4), 142.6 (C_1), 137.3 (C_8), 136.6 (C_6), 134.2 (C_{13}), 129.5 (C_{14}), 124.7 (C_3), 124.6 (C_2), 122.0 (C_7), 121.9 (C_{15}), 29.3 (C_9), 28.8 (C_{12}), 23.2 (C_{10}), 23.1 (C_{11}).

HRMS (m/z ESI⁺): m/z found 328.1125 $[M+1]^+$; $C_{13}H_{12}N_3O_2S$ requires 328.1119

1-(4-Nitrophenyl)-3-(pyridin-2-yl) thiourea 20B.[11]

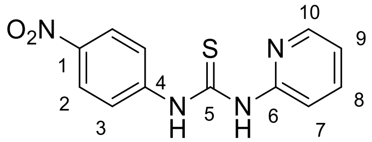

Yield: 40%

^{1}H NMR (600 MHz, DMSO-d$_6$) δ 14.47 (s, 1H, $PyN\underline{H}$), 11.21 (s, 1H, $NO_2PhN\underline{H}$), 8.39 (dd, 1H, H_{10}, $J_{10\text{-}9}$ = 5.1 Hz, $J_{10\text{-}8}$ = 1.8 Hz), 8.28 (d, 2H, H_2, $J_{2\text{-}3}$ = 7.0 Hz), 8.18 (d, 2H, H_3, $J_{2\text{-}3}$ = 7.0 Hz), 7.90 - 7.86 (m, 1H, H_8), 7.30 (d, 1H, H_7, $J_{7\text{-}8}$ = 8.4 Hz), 7.20 - 7.16 (m, 1H, H_9).

^{13}C NMR (150 MHz, DMSO-d$_6$) δ 178.0 (C_5), 153.2 (C_6), 145.6 (C_{10}), 145.0 (C_4), 143.5 (C_1), 139.7 (C_8), 124.3 (C_2), 124.3 (C_2), 123.0 (C_3), 118.7 (C_9), 113.2 (C_7).

HRMS (m/z ESI⁺): m/z found 275.0600 $[M+1]^+$; $C_{12}H_{11}N_4O_2S$ requires 275.0602

1-(4-Nitrophenyl)-3-(3-methylpyridin-2-yl)thiourea, 21B.[11]

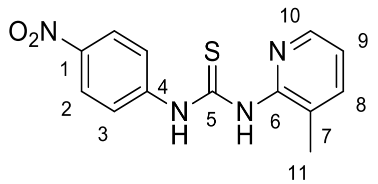

Yield: 46%

^{1}H NMR (600 MHz, DMSO-d$_6$) δ 13.84 (s, 1H, PyN<u>H</u>), 9.59 (s, 1H, NO_2PhN<u>H</u>), 8.32 - 8.23 (m, 3H, H_2, H_{10}), 8.12 (d, 2H, H_3, $J_{2\text{-}3}$ = 9.1 Hz), 7.80 (br, 2H, H_8), 7.18 (br, 1H, H_9), 2.38 (s, 3H, H_{11}).

^{13}C NMR (150 MHz, DMSO-d$_6$) δ 178.7 (C_5), 153.3 (C_7), 151.9 (C_{10}), 143.8.0 (C_4), 143.1 (C_1), 142.5 (C_6), 140.8 (C_8), 139.7 124.3 (C_2), 123.0 (C_3), 118.7 (C_9), 17.0 (C_{11}).

HRMS (m/z ESI$^+$): m/z found 289.0762 $[M+1]^+$; $C_{13}H_{13}N_4O_2S$ requires 289.0759

1-(4-Nitrophenyl)-3-(4-methylpyridin-2-yl)thiourea, 22B

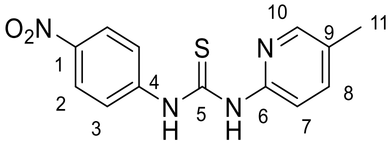

Yield: 62%

^{1}H NMR (400 MHz, DMSO-d$_6$) δ 13.39 (s, 1H, PyN<u>H</u>), 11.11 (s, 1H, NO_2PhN<u>H</u>), 8.24 (d, 1H, H_2, $J_{2\text{-}3}$ = 9.0 Hz), 8.20 (s, 1H, H_{10}), 8.15 (d, 1H, H_3, $J_{2\text{-}3}$ = 9.0 Hz), 7.71 (d, 1H, H_8, $J_{2\text{-}3}$ = 8.5 Hz), 7.20 (d, 1H, H_7, $J_{2\text{-}3}$ = 8.5 Hz), 2.25 (s, 3H, H_{11}).

^{13}C NMR (100 MHz, DMSO-d$_6$) δ 178.2 (C_5), 151.7 (C_6), 145.5 (C_1), 145.4 (C_{10}), 143.9 (C_4), 140.8 (C_8), 128.4 (C_9), 124.7 (C_2), 123.3 (C_3), 113.2 (C_7), 17.7 (C_{11}).

HRMS (m/z ESI$^+$): m/z found 289.0753 $[M+1]^+$; $C_{13}H_{13}N_4O_2S$ requires 289.0759

1-(4-Nitrophenyl)-3-(6-methylpyridin-2-yl)thiourea, 23B

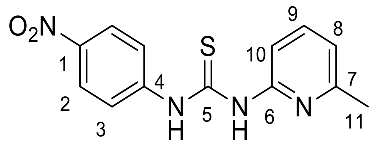

Yield: 54%

^{1}H NMR (400 MHz, DMSO-d$_6$) δ 14.08 (s, 1H, PyN<u>H</u>), 11.13 (s, 1H, NO_2PhN<u>H</u>), 8.26 (d, 2H, H_2, $J_{2\text{-}3}$ = 9.0 Hz), 8.17 (d, 2H, H_3, $J_{2\text{-}3}$ = 9.0 Hz), 7.75 (t, 1H, H_9, $J_{9\text{-}8}$ = $J_{9\text{-}10}$ = 7.9 Hz), 7.08 (d, 1H, H_8 $J_{8\text{-}9}$ = 8.3 Hz), 7.01 (d, 1H, H_{10}, $J_{10\text{-}9}$ = 7.3 Hz), 2.51 (s, 3H, H_{11}).

^{13}C NMR (100 MHz, DMSO-d$_6$) δ 178.2 (C_5), 155.0 (C_6), 153.2 (C_{10}), 145.5 (C_4), 143.9 (C_1), 140.4 (C_8), 124.9 (C_2), 122.8 (C_3), 118.4 (C_9), 110.5 (C_7), 17.0 (C_{11}).

HRMS (m/z ESI$^+$): m/z found 289.0765 $[M+1]^+$; $C_{13}H_{13}N_4O_2S$ requires 289.0759

1-(4-Nitrophenyl)-3-(5-chloropyridin-2-yl)thiourea, 24B.[11]

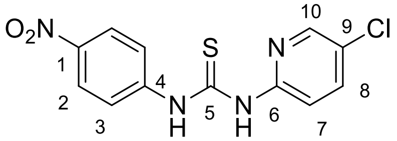

Yield: 85%

^{1}H NMR (400MHz, DMSO-d$_6$) δ 13.81 (s, 1H, mClPyN<u>H</u>), 11.33 (s, 1H, NO_2PhN<u>H</u>), 8.46 (d, 1H, H_{10}, $J_{10\text{-}8}$ = 2.7 Hz), 8.28 (d, 2H, H_2, $J_{2\text{-}3}$ = 9.3 Hz), 8.15 (d, 2H, H_3, $J_{2\text{-}3}$ = 9.3 Hz), 8.01 (dd, 1H, H_8, $J_{8\text{-}7}$ = 9.0 Hz, $J_{10\text{-}8}$ = 2.7 Hz), 7.35 (d, 1H, H_7, J = 8.8 Hz).

^{13}C NMR (100 MHz, DMSO-d$_6$) δ 178.2 (C_5), 152.1 (C_{10}), 145.3 (C_4), 144.6 (C_6), 144.2 (C_1), 139.9 (C_8), 125.1 (C_9), 124.7 (C_8), 123.7 (C_3), 115.2 (C_9).

HRMS (m/z ESI$^+$): m/z found 310.0112 $[M+1]^+$; $C_{12}H_{10}N4_3O_2SCl$ requires 310.0105

1-(1H-Benzo[d]imidazol-2-yl)-3-(4-nitrophenyl) thiourea, 25B.[11]

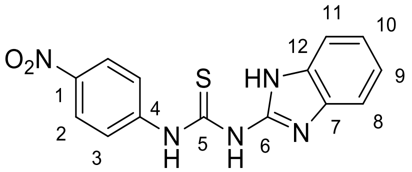

Yield: 31%

^{1}H NMR (600 MHz, DMSO-d$_6$) δ 12.94 (s, 2H, ArN<u>H</u>, N<u>H</u>), 10.33 (s, 1H, NO_2PhN<u>H</u>), 8.13 (br, 4H, H_2, H_3), 7.55 - 7. 51 (m, 2H, H_9, H_{10}), 7.27 - 7.23 (m, 2H, H_8, H_{11}).

^{13}C NMR (150 MHz, DMSO-d$_6$) δ 182.6 (C_5), 155.1 (C_6), 146.6 (C_4), 140.7 (C_1), 143.5 (C_1), 129.2 (C_7, C_{12}), 124.4 (C_2), 122.9 (C_3), 119.8 (C_8), 118.7 (C_9), 113.2 (C_{10}), 118.7 (C_8), 113.2 (C_{12}).

HRMS (m/z ESI$^+$): m/z found 314.0703 $[M+1]^+$; $C_{14}H_{12}N_5O_2S$ requires 314.0711

***tert*-butyl (2-(4-(4-(3-(4-nitrophenyl)thioureido)phenyl)-1H-1,2,3-triazol-1-yl)ethyl)carbamate, Boc26B**

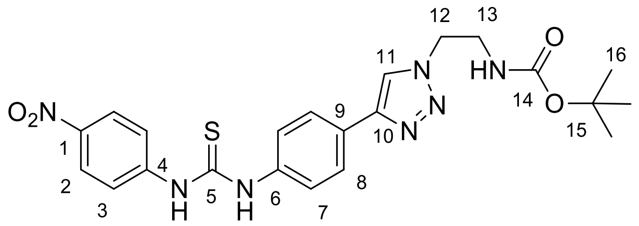

Yield: 98%

^{1}H NMR (400 MHz, DMSO-d$_6$) δ 10.39 (s, 1H, NO$_2$PhN<u>H</u>), 10.31 (s, 1H, ArN<u>H</u>), 8.45 (s, 1H, H_{11}), 8.19 (d, 2H, H_2, $J_{2\text{-}3}$ = 9.2 Hz), 7.82 (d, 2H, H_3, $J_{2\text{-}3}$ = 9.2 Hz), 7.79 (d, 2H, H_8, $J_{7\text{-}8}$ = 8.6 Hz), 7.57 (d, 1H, H_8, $J_{7\text{-}8}$ = 8.4 Hz), 7.01 (t, 1H, N<u>H</u>Boc, $J_{N\underline{H}\text{-}13}$ = 6.0 Hz), 4.41 (t, 2H, H_{12}, $J_{12\text{-}13}$ = 6.0 Hz), 3.40 (q, 2H, H_{13}, $J_{12\text{-}13}$ = $J_{13\text{-}N\underline{H}}$ = 6.0 Hz), 1.32 (s, 9H, H_{16}).

^{13}C NMR (100 MHz, DMSO-d$_6$) δ 179.6 (C_5), 156.1 (C_{14}), 146.4 (C_4), 142.8 (C_2), 139.0 (C_6), 127.9 (C_9), 125.8 (C_8), 124.8 (C_2), 124.3 (C_7), 122.1 (C_3), 121.8 (C_{11}), 78.4 (C_{15}), 49.7 (C_{12}), 40.5 (C_{13}), 28.6 (C_{16}).

HRMS (m/z ESI$^+$): m/z found 484.1771 [M+1]$^+$; $C_{22}H_{26}N_7O_4S$ requires 484.1762

2.3. Synthesis and Characterisation of *N,N'*-(bis-Boc-Guanidino)(aryl) thioureas

All were prepared according to **Method C** starting directly from the corresponding aminophenyl thioureas **2C-6C/Boc8C/9C-Boc26C** which were prepared by reduction of the nitrophenyl thioureas **2B-6B/Boc8B/9B-Boc26B** according to **Method B** and used without purification. Compound **diBoc1** has already been reported by us in reference 13; compounds **2D-6D, Boc8D-15D**, **18D-21D**, **24D-25D** have already been reported by us in reference 2.

1-{4-[1,3-di(*tert*-butoxycarbonyl)guanidino]phenyl}thiourea, diBoc1.[12]

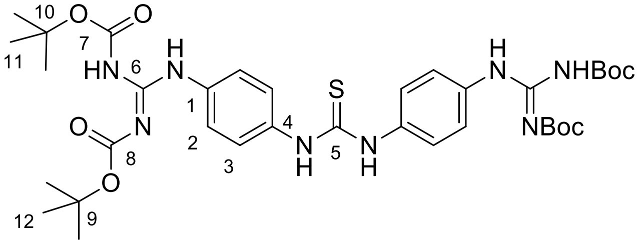

Yield: 32%

^{1}H NMR (600 MHz, DMSO-d$_6$) δ 11.43 (s, 2H, PhNH), 9.97 (s, 2H, NHPhNHC=S), 9.79 (s, 2H, NHPhNHC=S), 7.53 – 7.40 (m, 8H, H_2, H_3), 1.52 (s, 9H, H_{11}), 1.41 (s, 9H, H_{12}).

^{13}C NMR (150 MHz, DMSO-d$_6$) δ 180.0 (C_5), 163.2 (C_6), 153.3 (C_7), 152.6 (C_8), 136.7 (C_4), 133.5 (C_1), 124.4 (C_3), 123.4 (C_2), 83.4 (C_{10}), 79.2 (C_9), 28.4 (C_{11}), 28.1 (C_{12}).

HRMS (m/z ESI$^+$): m/z found 743.3557 $[M+1]^+$; $C_{35}H_{51}N_8O_8S$ requires 743.3546

1-{4-[1,3-di(*tert*-butoxycarbonyl)guanidino]phenyl}-3-phenylthiourea, 2D.[11]

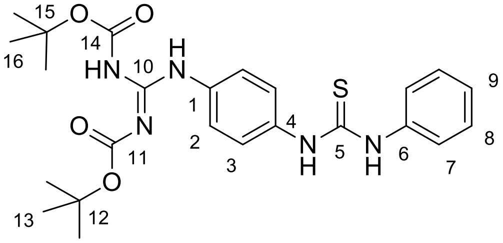

Yield: 47%

^{1}H NMR (600 MHz, DMSO-d$_6$) δ 11.44 (s, 1H, PhNH), 9.98 (s, 1H, NHPhNHC=S), 9.80 (s, 1H, NHPhNHC=S), 9.78 (s, 1H, BocNH), 7.53 - 7.43 (m, 6H, H_2, H_3, H_7), 7.34 (t, 2H, H_8, J_{7-8} = J_{9-8} = 7.5 Hz), 7.13 (t, 1H, H_9, J_{7-8} = 7.5 Hz), 1.53 (s, 9H, H_{16}), 1.41 (s, 9H, H_{13}).

^{13}C NMR (150 MHz, DMSO-d$_6$) δ 180.1 (C_5), 163.2 (C_{10}), 153.3 (C_{14}), 152.6 (C_{11}), 140.0 (C_6), 136.8 (C_4), 133.6 (C_1), 128.9 (C_8), 124.9 (C_9), 124.5 (C_3), 124.5 (C_7), 123.5 (C_2), 83.8 (C_{15}), 79.3 (C_{12}), 28.4 (C_{13}), 28.3 (C_{16}).

HRMS (m/z ESI$^+$): m/z found 486.2176 $[M+1]^+$; $C_{13}H_{12}N_3O_2S$ requires 486.2175

1-{4-[1,3-di(*tert*-butoxycarbonyl)guanidino]phenyl}-3-(4-fluorophenyl)thiourea, 3D.[11]

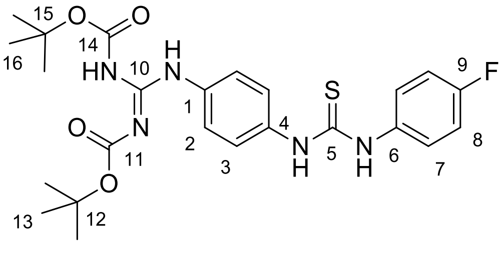

Yield: 41%

^{1}H NMR (600 MHz, DMSO-d$_6$) δ 11.42 (s, 1H, FPhNH), 9.97 (s, 1H, NHPhNHC=S), 9.78 (s, 1H, NHPhNHC=S), 9.75 (s, 1H, BocNH), 7.53 - 7.42 (m, 6H, H_2, H_3, H_7), 7.34 (d, 2H, H_7, $J_{8\text{-}F}$ = $J_{8\text{-}F}$ = 8.7 Hz), 1.52 (s, 9H, H_{16}), 1.41 (s, 9H, H_{13}).

^{13}C NMR (150 MHz, DMSO-d$_6$) δ 180.4 (C_5), 163.2 (C_{10}), 153.3 (C_{11}), 152.6 (C_{14}), 136.6 (C_4), 136.2 (C_1), 133.4 (C_6), 126.6 (d, C_7, $J_{7\text{-}F}$ = 8.2 Hz), 124.5 (C_2) 123.4 (C_3), 115.5 (d, C_8, $J8_{\text{-}F}$ = 22.8 Hz), 83.8 (C_{15}), 79.9 (C_{12}), 28.4 (C_{16}), 28.1 (C_{13}).

HRMS (m/z ESI$^+$): m/z found 504.2085 $[M+1]^+$; $C_{24}H_{131}FN_5O_4S$ requires 504.2081

1-{4-[1,3-di(*tert*-butoxycarbonyl)guanidino]phenyl}-3-(4-bromophenyl)thiourea, 4D.[11]

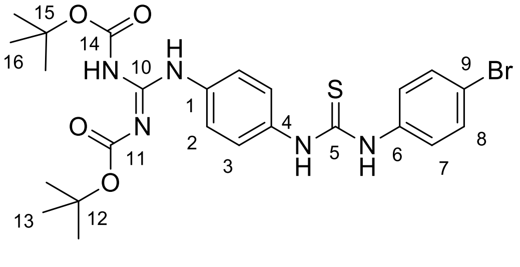

Yield: 30%

^{1}H NMR (600 MHz, DMSO-d$_6$) δ 11.43 (s, 1H, BrPhNH), 9.96 (s, 1H, NHPhNHC=S), 9.70 (s, 1H, NHPhNHC=S), 9.68 (s, 1H, BocNH), 7.49 (d, 2H, H_2, $J_{2\text{-}3}$ = 9.1 Hz), 7.45 (d, 2H, H_3, $J_{2\text{-}3}$ = 9.1 Hz), 7.34 (d, 2H, H_7, $J_{7\text{-}8}$ = 8.4 Hz), 7.14 (d, 2H, H_8, $J_{7\text{-}8}$ = 8.4 Hz), 1.52 (s, 9H, H_{14}), 1.41 (s, 9H, H_{17}).

^{13}C NMR (150 MHz, DMSO-d$_6$) δ 180.0 (C_5), 163.3 (C_{10}), 153.5 (C_{14}), 152.8 (C_{11}), 137.8 (C_4), 136.5 (C_1), 134.2 (C_6), 129.4 (C_8), 124.4 (C_7) 124.3 (C_3), 123.4 (C_2), 117.2 (C_9), 83.9 (C_{15}), 79.2 (C_{12}), 28.2 (C_{16}), 21.0 (C_{13}).

HRMS (m/z ESI$^+$): m/z found 565.5021 $[M+1]^+$; $C_{24}H_{31}BrN_5O_4S$ requires 565.5029

1-{4-[1,3-di(*tert*-butoxycarbonyl)guanidino]phenyl}-3-(4-methylphenyl)thiourea, 5D.[11]

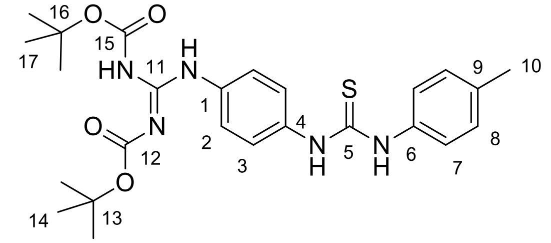

Yield: 29%

^{1}H NMR (600 MHz, DMSO-d$_6$) δ 11.44 (s, 1H, ArNH), 9.97 (s, 1H, NHPhNHC=S), 9.70 (s, 1H, NHPhNHC=S), 9.67 (s, 1H, BocNH), 7.49 (d, 2H, H_2, J_{2-3} = 9.0 Hz), 7.45 (d, 2H, H_3, J_{2-3} = 9.0 Hz), 7.34 (d, 2H, H_7, J_{7-8} = 8.4 Hz), 7.14 (d, 2H, H_8, J_{7-8} = 8.4 Hz), 2.29 (s, 3H, H_{10}), 1.53 (s, 9H, H_{14}), 1.43 (s, 9H, H_{17}).

^{13}C NMR (150 MHz, DMSO-d$_6$) δ 180.0 (C_5), 163.2 (C_{11}), 153.4 (C_{15}), 152.6 (C_{12}), 137.2 (C_4), 136.8 (C_1), 134.2 (C_6), 133.4 (C_9), 129.4 (C_8), 124.4 (C_7) 124.3 (C_3), 123.4 (C_2), 83.9 (C_{16}), 79.2 (C_{13}), 28.4 (C_{14}), 28.2 (C_{17}), 21.0 (C_{10}).

HRMS (m/z ESI$^+$): m/z found 500.2324 $[M+1]^+$; $C_{25}H_{34}N_5O_3S$ requires 500.2331

1-{4-[1,3-di(*tert*-butoxycarbonyl)guanidino]phenyl}-3-(4-*tert*-butylphenyl)thiourea, 6D.[11]

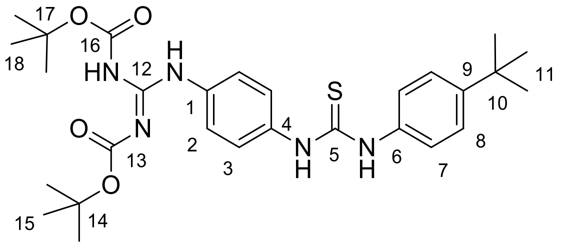

Yield: 32%

^{1}H NMR (600 MHz, DMSO-d$_6$) δ 11.44 (s, 1H, tBuPhNH), 9.97 (s, 1H, NHPhNHC=S), 9.72 (br, 2H, NHPhNHC=S, BocNH), 7.49 (d, 2H, H_2, J_{2-3} = 9.5 Hz), 7.45 (d, 2H, H_3, J_{2-3} = 9.5 Hz), 7.39 (d, 2H, H_7, J_{7-8} = 8.6 Hz), 7.35 (d, 2H, H_8, J_{7-8} = 8.6 Hz), 1.52 (s, 9H, H_{15}), 1.41 (s, 9H, H_{18}), 1.28 (s, 9H, H_{11}).

^{13}C NMR (150 MHz, DMSO-d$_6$) δ 180.0 (C_5), 163.2 (C_{12}), 153.4 (C_{16}), 152.6 (C_{13}), 137.2 (C_4), 136.8 (C_1), 134.2 (C_6), 133.4 (C_9), 125.4 (C_8), 124.4 (C_7) 123.8 (C_3), 123.4 (C_2), 83.9 (C_{16}), 79.2 (C_{13}), 31.7 (C_{11}), 28.4 (C_{18}), 28.1 (C_{15}).

HRMS (m/z ESI$^+$): m/z found 542.2804 $[M+1]^+$; $C_{28}H_{40}N_5O_4S$ requires 542.2801

1-{4-[1,3-di(*tert*-butoxycarbonyl)guanidino]phenyl}-3-{4-[(*tert*-butoxycarbonyl)amino]phenyl} thiourea, Boc8D.[11]

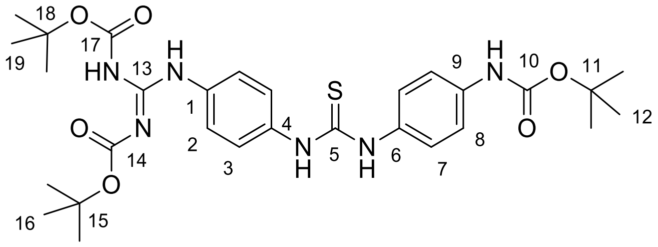

Yield: 34%

^{1}H NMR (600 MHz, DMSO-d$_6$) δ 11.42 (s, 1H, ArNH), 9.96 (s, 1H, BocNH), 9.64 (s, 1H, NHPhNHC=S), 9.61 (s, 1H, NHPhNHC=S), 9.32 (s, 1H, BocNHPh), 7.48 (d, 2H, H_2, $J_{2\text{-}3}$ = 8.9 Hz), 7.43 (d, 2H, H_3, $J_{2\text{-}3}$ = 8.9 Hz), 7.40 (d, 2H, H_7, $J_{7\text{-}8}$ = 7.9 Hz), 7.30 (d, 2H, H_8, $J_{7\text{-}8}$ = 7.9 Hz), 1.52 (s, 9H, H_{16}), 1.41 (s, 9H, H_{19}), 1.39 (s, 3H, H_{12}).

^{13}C NMR (150 MHz, DMSO-d$_6$) δ 180.1 (C_5), 163.2 (C_{13}), 153.4 (C_{10}), 153.3 (C_{17}), 152.6 (C_{14}), 136.9 (C_4), 136.8 (C_1), 133.9 (C_6), 133.4 (C_9), 125.3 (C_7), 124.5 (C_3), 123.4 (C_8), 118.7 (C_8), 83.8 (C_{18}), 79.4 (C_{15}), 79.3 (C_{11}), 28.6 (C_{12}), 28.4 (C_{16}), 28.1 (C_{19}).

HRMS (m/z ESI$^+$): m/z found 601.2818 $[M+1]^+$; $C_{29}H_{41}N_6O_6S$ requires 601.2808

1-{4-[1,3-di(*tert*-butoxycarbonyl)guanidino]phenyl}-3-(4-ethylphenyl)thiourea, 9D.[11]

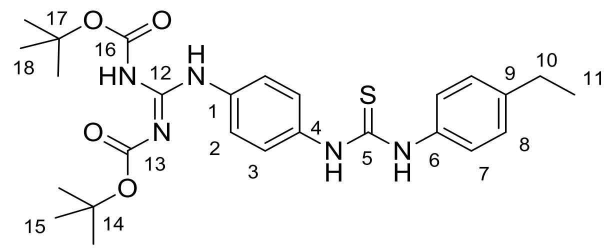

Yield: 37%

^{1}H NMR (400 MHz, DMSO-d$_6$) δ 11.43 (s, 1H, PhNH), 9.96 (s, 1H, NHPhNHC=S), 9.71 (s, 1H, NHPhNHC=S), 9.70 (s, 1H, BocNH), 7.49 (d, 2H, H_2, $J_{2\text{-}3}$ = 8.9 Hz), 7.46 (d, 2H, H_3, $J_{2\text{-}3}$ = 8.9 Hz), 7.37

(d, 2H, H_8, J_{8-7} = 8.3 Hz), 7.18 (d, 2H, H_7, J_{8-7} = 8.3 Hz), 2.59 (q, 2H, H_{10}, J_{10-11} = 7.60 Hz), 1.53 (s, 9H, H_{16}), 1.41 (s, 9H, H_{13}), 1.18 (t, 3H, H_{11}, J_{10-11} = 7.60 Hz).

^{13}C NMR (100 MHz, DMSO-d$_6$) δ 180 (C_5), 163.1 (C_{12}), 153.3 (C_{16}), 152.6 (C_{13}), 140.6 (C_6), 137.5 (C_9), 136.9 (C_4), 133.4 (C_1), 128.2 (C_7), 125.4 (C_8), 124.4 (C_3), 123.4 (C_2), 83.9 (C_{17}), 79.3 (C_{14}), 28.4 (C_{15}), 28.3 (C_{18}), 28.2 (C_{10}), 16.1 (C_{11})

HRMS (m/z ESI$^+$): m/z found 513.2484 [M+1]$^+$; $C_{26}H_{36}N_5O_4S$ requires 513.2488

1-{4-[1,3-di(*tert*-butoxycarbonyl)guanidino]phenyl}-3-(4-benzylphenyl)thiourea, 10D.[11]

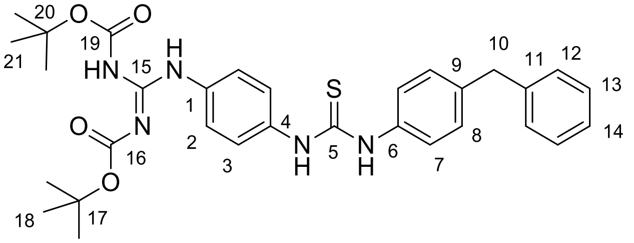

Yield: 51%

^{1}H NMR (600 MHz, DMSO-d$_6$) δ 11.43 (s, 1H, BnPhN<u>H</u>), 9.96 (s, 1H, NHPhN<u>H</u>C=S), 9.73 (br, 2H, N<u>H</u>PhNHC=S, BocN<u>H</u>), 7.53 - 7.15 (m, 13H, H_2, H_3, H_7, H_8, H_{12}, H_{13}, H_{14}), 3.91 (s, 3H, H_{10}), 1.52 (s, 9H, H_{21}), 1.41 (s, 9H, H_{18}).

^{13}C NMR (150 MHz, DMSO-d$_6$) δ 180.0 (C_5), 158.3 (C_{15}), 157.1 (C_{16}), 153.0 (C_{19}), 143.8 (C_4), 136.7 (C_1), 141.9 (C_{11}), 138.5 (C_6), 137.4 (C_9), 129.3 (C_{13}), 129.3 (C_8), 128.8 (C_{12}), 126.4 (C_{14}), 130.8 (C_2), 124.5 (C_7), 124.2 (C_3), 83.8 (C_{21}), 79.3 (C_{20}), 41.1 (C_{10}), 28.4 (C_{19}), 28.1 (C_{20}).

HRMS (m/z ESI$^+$): m/z found 576.2650 [M+1]$^+$; $C_{31}H_{38}N_5O_4S$ requires 576.2644

1-{4-[1,3-di(*tert*-butoxycarbonyl)guanidino]phenyl}-3-(4-phenoxyphenyl)thiourea, 11D.[11]

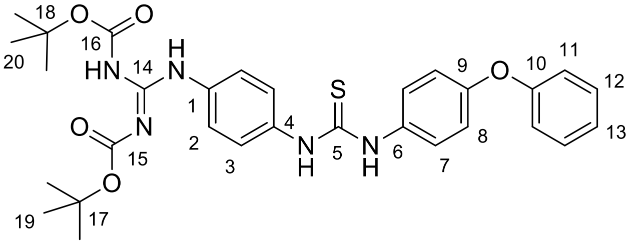

Yield: 46%

^{1}H NMR (600 MHz, DMSO-d$_6$) δ 11.43 (s, 1H, PhOPhNH), 9.97 (s, 1H, NHPhNHC=S), 9.78 (s, 1H, NHPhNHC=S), 9.75 (s, 1H, BocNH), 7.53 - 7.35 (m, 8H, H_2, H_3, H_7, H_{12}), 7.14 (t, 1H, H_{13}, J_{12-13} = 7.1 Hz), 7.03 - 6.97 (m, 4H, H_8, H_{11}), 1.52 (s, 9H, H_{19}), 1.41 (s, 9H, H_{20}).

^{13}C NMR (150 MHz, DMSO-d$_6$) δ 179.9 (C_5), 160.1 (C_{10}), 158.9 (C_9), 157.6 (C_{14}), 153.2 (C_{16}), 152.7 (C_{15}), 143.9 (C_4), 136.4 (C_1), 133.7 (C_6), 130.2 (C_2), 126.3 (C_{11}), 124.5 (C_7), 123.7 (C_1), 123.4 (C_3), 119.3 (C_6), 118.8 (C_{12}), 83.8 (C_{18}), 79.3 (C_{17}), 28.4 (C_{19}), 28.1 (C_{20}).

HRMS (m/z ESI$^+$): m/z found 578.2441 $[M+1]^+$; $C_{30}H_{36}N_5O_5S$ requires 578.2437

1-{4-[1,3-di(*tert*-butoxycarbonyl)guanidino]phenyl}-3-(4-ethynylphenyl)thiourea, 12D.[11]

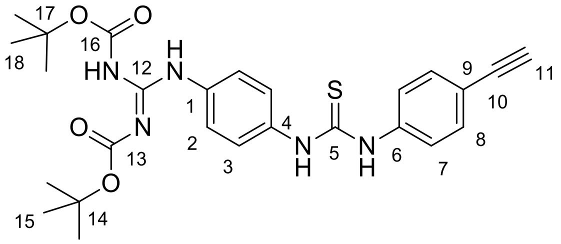

Yield: 27%

^{1}H NMR (600 MHz, CDCl$_3$) δ 11.59 (s, 1H, ArNH), 10.31 (br, 1H, BocNH), 8.47 (s, 1H, NHPhNHC=S), 8.24 (s, 1H, NHPhNHC=S), 7.55 (d, 2H, H_8, J_{8-7} = 8.7 Hz), 7.45 (d, 2H, H_2, J_{2-3} = 8.7 Hz), 7.41 (d, 2H, H_3, J_{2-3} = 8.7 Hz), 7.30 (d, 1H, H_7, J_{8-7} = 8.7 Hz), 3.01 (s, 3H, H_{11}), 1.52 (s, 9H, H_{15}), 1.41 (s, 9H, H_{18}).

^{13}C NMR (150 MHz, CDCl$_3$) δ 179.3 (C_5), 163.2 (C_{12}), 154.0 (C_{16}), 153.2 (C_{13}), 138.5 (C_4), 134.7 (C_1), 134.0 (C_6), 132.8 (C_3), 125.3 (C_7), 123.9 (C_8, C_2), 119.4 (C_9), 84.1 (C_{10}), 83.2 (C_{17}), 80.2 (C_{14}), 77.4 (C_{11}), 28.1 (C_{15}, C_{18}).

HRMS (m/z ESI$^+$): m/z found 510.2175 $[M+1]^+$; $C_{26}H_{32}N_5O_4S$ requires 510.2175

1-{4-[1,3-di(*tert*-butoxycarbonyl)guanidino]phenyl}-3-(3-methoxyphenyl)thiourea, 13D.[11]

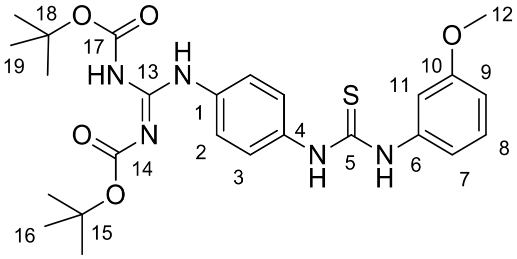

Yield: 28%

^{1}H NMR (400 MHz, DMSO-d$_6$) δ 11.43 (s, 1H, MeOPhNH), 9.97 (s, 1H, NHPhNHC=S), 9.79 (s, 1H, NHPhNHC=S), 9.77 (s, 1H, BocNH), 7.50 (d, 2H, H_2, $J_{2\text{-}3}$ = 8.8 Hz), 7.45 (d, 2H, H_3, $J_{2\text{-}3}$ = 8.8 Hz), 7.24 (t, 1H, H_8, $J_{7\text{-}8}$ = $J_{8\text{-}9}$ = 8.1 Hz), 7.03 (s, 1H, H_{11}), 7.05 (d, 1H, H_9, $J_{8\text{-}9}$ = 8.1 Hz), 6.76 (d, 1H, H_7, $J_{7\text{-}8}$ = 8.1 Hz), 3.76 (s, 3H, H_{12}), 1.52 (s, 9H, H_{16}), 1.41 (s, 9H, H_{19}).

^{13}C NMR (100 MHz, DMSO-d$_6$) δ 179.9 (C_5), 160.1 (C_{13}), 159.4 (C_{10}), 153.7 (C_{17}), 152.2 (C_{14}), 140.5 (C_6), 134.7 (C_1), 129.9 (C_8), 124.9 (C_4), 124.2 (C_3), 122.1 (C_2), 116.1 (C_7), 110.4 (C_9), 109.8 (C_{11}), 84.3 (C_{18}), 79.1 (C_{15}), 55.3 (C_{12}), 28.4 (C_{16}), 28.2 (C_{19}).

HRMS (m/z ESI$^+$): m/z found 516.2283 $[M+1]^+$; $C_{25}H_{34}N_5O_5S$ requires 516.2280

1-{4-[1,3-di(*tert*-butoxycarbonyl)guanidino]phenyl}-3-(3,4-dimethylphenyl)thiourea, 14D.[11]

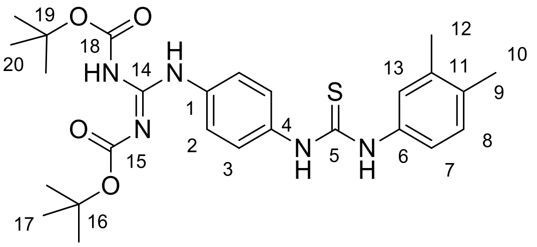

Yield: 33%

^{1}H NMR (600 MHz, DMSO-d$_6$) δ 11.44 (s, 1H, ArNH), 9.96 (s, 1H, NHPhNHC=S), 9.64 (s, 1H, NHPhNHC=S), 9.62 (s, 1H, BocNH), 7.48 (d, 2H, H_2, $J_{2\text{-}3}$ = 8.7 Hz), 7.44 (d, 2H, H_3, $J_{2\text{-}3}$ = 8.7 Hz), 7.20 (d, 1H, H_{13}, $J_{13\text{-}7}$ = 1.7 Hz), 7.17 (dd, 1H, H_7, $J_{7\text{-}8}$ = 8.1 Hz, $J_{13\text{-}7}$ = 1.7 Hz), 7.09 (d, 1H, H_8, $J_{7\text{-}8}$ = 8.1 Hz), 2.21 (s, 3H, H_{12}), 2.20 (s, 3H, H_{10}), 1.53 (s, 9H, H_{20}), 1.43 (s, 9H, H_{17}).

^{13}C NMR (150 MHz, DMSO-d$_6$) δ 180.0 (C_5), 163.2 (C_{14}), 153.4 (C_{18}), 152.6 (C_{15}), 137.4 (C_4), 136.9 (C_9), 136.7 (C_1), 133.4 (C_{11}), 133.1 (C_6), 129.9 (C_8), 125.6 (C_{13}), 124.5 (C_3), 123.3 (C_2), 121.9 (C_7), 83.8 (C_{19}), 79.3 (C_{16}), 28.4 (C_{17}), 28.1 (C_{20}), 20.0 (C_{10}), 19.3 (C_{12}).

HRMS (m/z ESI$^+$): m/z found 514.2492 $[M+1]^+$; $C_{26}H_{36}N_5O_4S$ requires 514.2488

1-{4-[1,3-di(*tert*-butoxycarbonyl)guanidino]phenyl}-3-(3-methyl-4-bromophenyl)thiourea, 15D.[11]

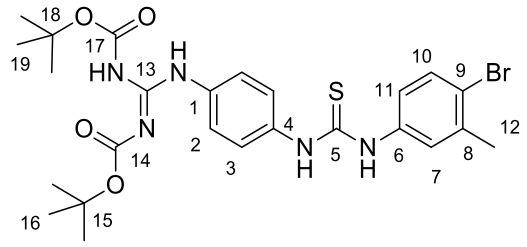

Yield: 25%

^{1}H NMR (600 MHz, DMSO-d$_6$) δ 11.44 (s, 1H, ArNH), 9.96 (s, 1H, NHPhNHC=S), 9.64 (s, 1H, NHPhNHC=S), 9.62 (s, 1H, BocNH), 7.51 (d, 2H, H_2, J_{2-3} = 9.2 Hz), 7.49 (d, 2H, H_3, J_{2-3} = 9.2 Hz), 7.56 (d, 1H, H_{10}, J_{10-11} = 8.8 Hz), 7.45 (d, 1H, H_7, J_{7-11} = 2.0 Hz), 7.42 (dd, 1H, H_{11}, J_{10-11} = 8.8 Hz, J_{7-11} = 2.0 Hz), 2.33 (s, 3H, H_{12}), 1.53 (s, 9H, H_{20}), 1.43 (s, 9H, H_{17}).

^{13}C NMR (150 MHz, DMSO-d$_6$) δ 180.1 (C_5), 158.3 (C_{13}), 156.2 (C_{14}), 153.3 (C_{17}), 137.2 (C_4), 136.9 (C_1), 136.8 (C_3), 133.9 (C_6), 133.4 (C_2), 127.3 (C_8), 124.5 (C_7), 123.4 (C_{10}), 122.1 (C_{11}), 118.7 (C_9), 83.8 (C_{18}), 79.4 (C_{15}), 28.4 (C_{19}), 28.1 (C_{16}), 23.1 (C_{12}).

HRMS (m/z ESI$^+$): m/z found 579.1431 $[M+1]^+$; $C_{25}H_{33}BrN_5O_4S$ requires 579.1437

1-{4-[1,3-di(*tert*-butoxycarbonyl)guanidino]phenyl}-3-(4-Chloro-3-trifluromethylphenyl) thiourea, 16D

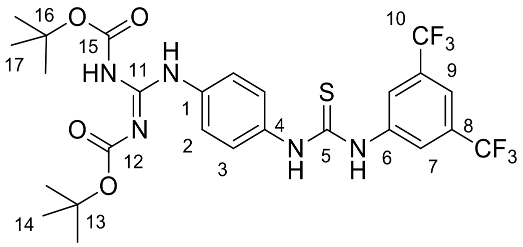

Yield: 37%

^{1}H NMR (600 MHz, DMSO-d$_6$) δ 11.42 (s, 1H, ArPhNH), 10.10 (s, 1H, NHPhNHC=S), 10.06 (s, 1H, NHPhNHC=S), 9.98 (s, 1H, BocNH), 8.09 (d, 1H, H_{11}, J_{11-7} = 2.5 Hz), 7.80 (dd, 1H, H_7, J_{8-7} = 8.6 Hz, J_{11-7} = 2.5 Hz), 7.67 (d, 1H, H_8, J_{8-7} = 8.6 Hz), 7.51 (d, 2H, H_2, J_{2-3} = 8.6 Hz), 7.42 (d, 2H, H_3, J_{2-3} = 8.6 Hz), 1.52 (s, 9H, H_{14}), 1.41 (s, 9H, H_{17}).

^{13}C NMR (150 MHz, DMSO-d$_6$) δ 179.7 (C_5), 162.6 (C_{12}), 152.7 (C_{14}), 152.1 (C_{13}), 139.2 (C_4), 135.6 (C_1), 133.5 (C_6), 131.5 (C_8), 128.45 (C_{11}), 124.1 (C_3), 123.1 (C_2), 123.0 (C_7), 123.0 (q, CF_3, JC-F = 271.2 Hz), 122.4 (C_9), 83.3 (C_{16}), 78.8 (C_{15}), 27.9 (C_{18}), 27.6 (C_{17}).

HRMS (m/z ESI$^+$): m/z found 558.1647 $[M+1]^+$; $C_{25}H_{30}ClF_3N_5O_4S$ requires 558.1654

1-{4-[1,3-di(*tert*-butoxycarbonyl)guanidino]phenyl}-3-(3,4-dimethoxylphenyl)thiourea, 17D

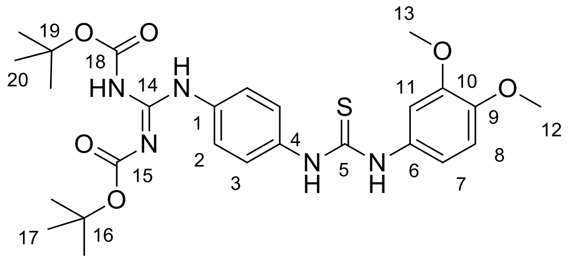

Yield: 30%

^{1}H NMR (600 MHz, DMSO-d$_6$) δ 11.43 (s, 1H, ArN<u>H</u>), 9.98 (s, 1H, NHPhN<u>H</u>C=S), 9.64 (s, 1H, N<u>H</u>PhNHC=S), 9.59 (s, 1H, BocN<u>H</u>), 7.49 (d, 2H, H_2, $J_{2\text{-}3}$ = 8.6 Hz), 7.44 (d, 2H, H_3, $J_{2\text{-}3}$ = 8.6 Hz), 7.12 (s, 1H, H_6), 6.92 (s, 2H, H_8, H_{11}), 2.21 (s, 3H, H_{12}), 3.75 (s, 3H, H_{12}), 3.73 (s, 3H, H_{13}), 1.52 (s, 9H, H_{20}), 1.41 (s, 9H, H_{17}).

^{13}C NMR (150 MHz, DMSO-d$_6$) δ 179.6 (C_5), 162.7 (C_{14}), 152.8 (C_{18}), 152.1 (C_{15}), 148.3 (C_9), 146.2 (C_{10}), 136.3 (C_1), 132.9 (C_4), 132.2 (C_6), 124.1 (C_8), 122.8 (C_3), 116.4 (C_2), 111.6 (C_7), 109.3 (C_8), 83.8 (C_{19}), 78.8 (C_{16}), 55.7 (C_{13}), 55.5 (C_{12}), 27.9 (C_{17}), 27.6 (C_{20}).

HRMS (m/z ESI$^+$): m/z found 546.2399 $[M+1]^+$; $C_{26}H_{36}N_5O_6S$ requires 546.2381

1-{4-[1,3-di(*tert*-butoxycarbonyl)guanidino]phenyl}-3-[(3,5-di-trifluoromethyl)phenyl] thiourea, 18D.[11]

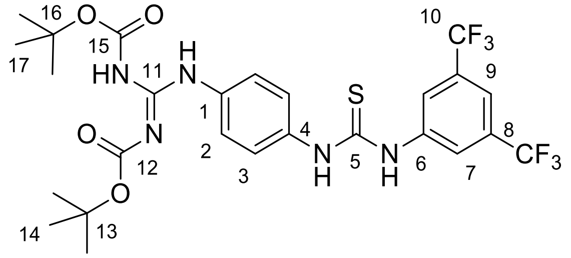

Yield: 44%

^{1}H NMR (600 MHz, DMSO-d$_6$) δ 11.41 (s, 1H, ArN<u>H</u>), 10.28 (s, 1H, NHPhN<u>H</u>C=S), 10.21 (s, 1H, N<u>H</u>PhNHC=S), 9.98 (s, 1H, BocN<u>H</u>), 8.24 (s, 2H, H_7), 7.79 (s, 1H, H_9) 7.54 (d, 2H, H_2, $J_{2\text{-}3}$ = 8.8 Hz), 7.42 (d, 2H, H_3, $J_{2\text{-}3}$ = 8.8 Hz), 1.52 (s, 9H, H_{14}), 1.41 (s, 9H, H_{17}).

^{13}C NMR (150 MHz, DMSO-d$_6$) δ 180.0 (C_5), 158.2 (C_{11}), 153.4 (C_{15}), 152.6 (C_{12}), 137.2 (C_4), 136.8 (C_1), 134.2 (C_6), 133.4 (C_7), 129.4 (C_3), 124.3 (C_2), 118.7 (C_9), 83.9 (C_{16}), 79.2 (C_{13}), 28.4 (C_{14}), 28.2 (C_{17}).

HRMS (m/z ESI$^+$): m/z found 622.1928 $[M+1]^+$; $C_{26}H_{30}F_6N_5O_4S$ requires 622.1922

1-{4-[1,3-di(*tert*-butoxycarbonyl)guanidino]phenyl}-3-(5,6,7,8-tetrahydronaphthalen-2-yl)thiourea, 19D.[11]

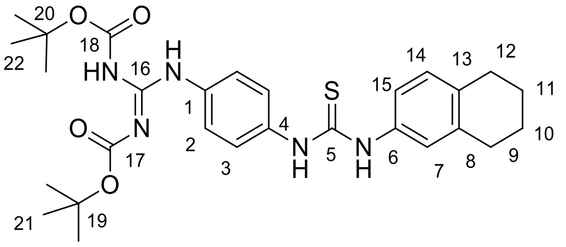

Yield: 42%

^{1}H NMR (600 MHz, DMSO-d$_6$) δ 11.44 (s, 1H, ArN<u>H</u>), 9.97 (s, 1H, NHPhN<u>H</u>C=S), 9.65 (s, 1H, N<u>H</u>PhNHC=S), 9.63 (s, 1H, BocN<u>H</u>), 7.45 (d, 2H, H_2, J_{2-3} = 8.9 Hz), 7.45 (d, 2H, H_3, J_{2-3} = 8.9 Hz), 7.16 (dd, 1H, H_{15}, J_{14-15} = 8.3 Hz, J_{7-15} = 2.0 Hz), 7.12 (d, 1H, H_7, J_{7-15} = 2.0 Hz), 7.01 (d, 1H, H_{14}, J_{14-15} = 8.3 Hz), 2.79 - 2.66 (m, 4H, H_9, H_{12}), 1.76 - 1.70 (m, 4H, H_{10}, H_{11}).

^{13}C NMR (150 MHz, DMSO-d$_6$) δ 178.0 (C_5), 163.2 (C_{16}), 153.3 (C_{17}), 152.6 (C_{18}), 152.6 (C_8), 137.1 (C_6), 137.0 (C_{13}), 136.9 (C_1), 133.5 (C_4), 133.4 (C_{14}), 129.3 (C_7), 124.6 (C_2), 123.3 (C_3), 122.0 (C_{15}), 83.8 (C_{20}), 79.3 (C_{19}), 29.3 (C_9), 28.8 (C_{12}), 28.3 (C_{21}), 28.1 (C_{22}), 23.2 (C_{11}), 23.1 (C_{10}).

HRMS (m/z ESI$^+$): m/z found 540.2651 $[M+1]^+$; $C_{28}H_{38}N_5O_4S$ requires 540.2644

1-{4-[1,3-di(*tert*-butoxycarbonyl)guanidino]phenyl}-3-(2-pyridyl)thiourea, 20D.[11]

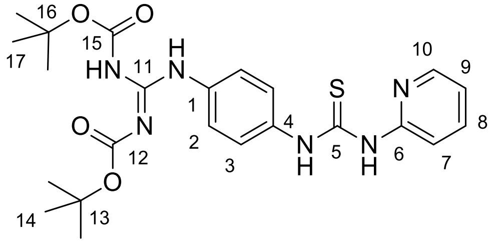

Yield: 40%

^{1}H NMR (600 MHz, DMSO-d$_6$) δ 13.84 (s, 1H, PyN<u>H</u>), 11.43 (s, 1H, NHPhN<u>H</u>C=S), 10.88 (s, 1H, N<u>H</u>PhNHC=S), 10.02 (s, 1H, BocN<u>H</u>), 8.33 (dd, 1H, H_{10}, J_{10-9} = 5.1 Hz, J_{10-8} = 1.3 Hz), 7.88 - 7.83 (m, 1H, H_8), 7.69 (d, 2H, H_2, J_{2-3} = 9.0 Hz), 7.57 (d, 2H, H_3, J_{2-3} = 9.0 Hz), 7.21 (d, 1H, H_7, J_{7-8} = 8.8 Hz), 7.15 - 7.09 (m, 1H, H_9), 1.53 (s, 9H, H_{14}), 1.42 (s, 9H, H_{17}).

^{13}C NMR (150 MHz, DMSO-d$_6$) δ 178.7 (C_5), 163.2 (C_{11}), 154.0 (C_{15}), 153.4 (C_{12}), 152.6 (C_6), 146.1 (C_{10}), 139.9 (C_8), 136.0 (C_4), 134.4 (C_1), 124.8 (C_2), 123.4 (C_3), 118.7 (C_9), 113.4 (C_7), 83.9 (C_{16}), 79.3 (C_{13}), 28.4 (C_{14}), 28.2 (C_{13}).

HRMS (m/z ESI+): m/z found 488.2129 [M+1]+; $C_{23}H_{31}N_6O_4S$ requires 488.2127

1-{4-[1,3-di(*tert*-butoxycarbonyl)guanidino]phenyl}-3-(3-methylpyrid-2-yl)thiourea, 21D.[11]

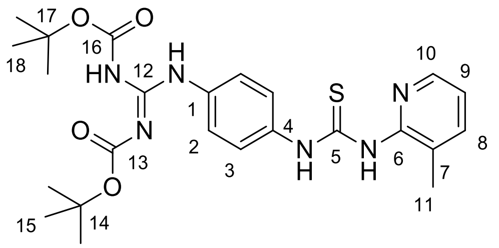

Yield: 37%

^{1}H NMR (600 MHz, DMSO-d$_6$) δ 13.84 (s, 1H, PyNH), 11.43 (s, 1H, NHPhNHC=S), 10.88 (s, 1H, NHPhNHC=S), 10.02 (s, 1H, BocNH), 8.23 (d, 1H, H_{10}, J_{10-9} = 4.3 Hz), 7.74 (d, 1H, H_8, J_{8-9} = 7.1 Hz), 7.68 (d, 2H, H_3, J_{2-3} = 8.9 Hz), 7.56 (d, 2H, H_2, J_{2-3} = 8.9 Hz), 7.12 (dd, 1H, H_9, J_{8-9} = 7.1 Hz, J_{10-9} = 4.3 Hz), 2.37 (s, 3H, H_{11}), 1.53 (s, 9H, H_{14}), 1.42 (s, 9H, H_{17}).

^{13}C NMR (150 MHz, DMSO-d$_6$) δ 178.7 (C_5), 163.1 (C_{12}), 153.3 (C_7), 152.6 (C_{16}), 151.9 (C_3), 143.8 (C_{10}), 140.8 (C_8), 136.0 (C_4), 134.4 (C_1), 124.8 (C_2), 123.4 (C_3), 122.4 (C_9), 119.5 (C_7), 83.9 (C_{17}), 79.3 (C_{14}), 28.4 (C_{15}), 28.2 (C_{18}), 17.0 (C_{11}).

HRMS (m/z ESI+): m/z found 501.2290 [M+1]+; $C_{24}H_{33}N_6O_4S$ requires 501.2284

1-{4-[1,3-di(*tert*-butoxycarbonyl)guanidino]phenyl}-3-(5-methylpyrid-2-yl)thiourea, 22D

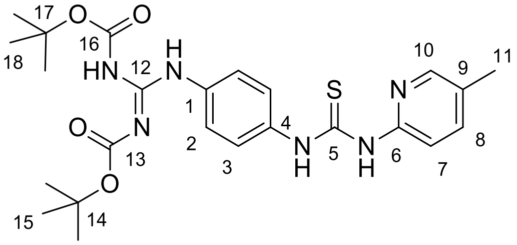

Yield: 22%

^{1}H NMR (600 MHz, DMSO-d$_6$) δ 13.77 (s, 1H, PyNH), 11.43 (s, 1H, NHPhNHC=S), 10.82 (s, 1H, NHPhNHC=S), 10.01 (s, 1H, BocNH), 8.16 (s, 1H, H_{10}), 7.62 – 7.64 (m, 3H, H_8, H_3), 7.56 (d, 2H, H_2, J_{2-3} = 8.7 Hz), 7.18 (d, 2H, H_7, J_{2-3} = 8.5 Hz), 2.25 (s, 3H, H_{11}), 1.52 (s, 9H, H_{14}), 1.42 (s, 9H, H_{17}).

^{13}C NMR (150 MHz, DMSO-d$_6$) δ 177.9 (C_5), 162.6 (C_{12}), 152.8 (C_{13}), 152.1 (C_{16}), 151.6 (C_6), 144.8 (C_{10}), 140.1 (C_8), 135.6 (C_1), 133.8 (C_4), 127.2 (C_9), 124.3 (C_3), 122.9 (C_2), 112.5 (C_7), 83.4 (C_{16}), 78.8 (C_{17}), 27.9 (C_{18}), 27.6 (C_{15}), 17.2 (C_{11}).

HRMS (m/z ESI$^+$): m/z found 501.2279 $[M+1]^+$; $C_{24}H_{33}N_6O_4S$ requires 501.2284

1-{4-[1,3-di(*tert*-butoxycarbonyl)guanidino]phenyl}-3-(6-methylpyrid-2-yl)thiourea, 23D

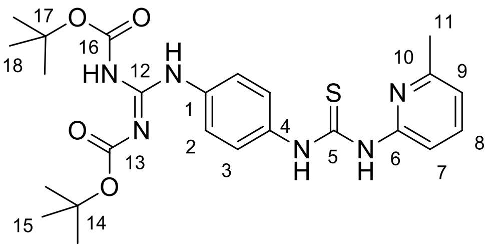

Yield: 67%

^{1}H NMR (600 MHz, DMSO-d$_6$) δ 14.18 (s, 1H, PyNH), 11.43 (s, 1H, NHPhNHC=S), 10.83 (s, 1H, NHPhNHC=S), 10.00 (s, 1H, BocNH), 7.78 (m, 3H, H_3, H_8), 7.57 (d, 2H, H_2, $J_{2\text{-}3}$ = 7.5 Hz), 7.06 (d, 1H, H_7, $J_{7\text{-}8}$ = 8.4 Hz), 6.97 (d, 1H, H_9, $J_{9\text{-}8}$ = 7.5 Hz), 2.49 (s, 3H, H_{11}), 1.52 (s, 9H, H_{14}), 1.42 (s, 9H, H_{17}).

^{13}C NMR (150 MHz, DMSO-d$_6$) δ 178.2 (C_5), 163.3 (C_{12}), 154.9 (C_{10}), 153.6 (C_6), 153.4 (C_{16}), 152.6 (C_{13}), 140.1 (C_8), 136.1 (C_1), 134.2 (C_4), 124.0 (C_3), 123.4 (C_2), 117.8 (C_9), 110.2 (C_7), 83.8 (C_{17}), 79.3 (C_{14}), 28.3 (C_{15}), 28.1 (C_{18}), 23.9 (C_{11}).

HRMS (m/z ESI$^+$): m/z found 501.2277 $[M+1]^+$; $C_{24}H_{33}N_6O_4S$ requires 501.2284

1-{4-[1,3-di(*tert*-butoxycarbonyl)guanidino]phenyl}-3-(5-chloropyrid-2-yl)thiourea, 24D.[11]

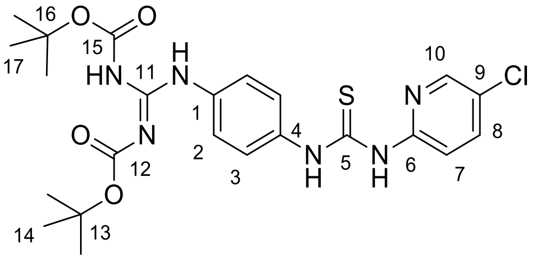

Yield: 31%

^{1}H NMR (400MHz, DMSO-d$_6$) δ 13.27 (s, 1H, PyNH), 11.43 (s, 1H, NHPhNHC=S), 11.04 (s, 1H, NHPhNHC=S), 10.02 (s, 1H, BocNH), 8.40 (d, 1H, H_{10}, $J_{10\text{-}8}$ = 2.7 Hz), 7.97 (dd, 1H, H_8, $J_{8\text{-}7}$ = 9.1 Hz, $J_{10\text{-}8}$ = 2.7 Hz), 7.67 (d, 2H, H_3, $J_{2\text{-}3}$ = 8.8 Hz), 7.57 (d, 2H, H_2, $J_{2\text{-}3}$ = 8.8 Hz), 7.32 (d, 1H, H_7, $J_{8\text{-}7}$ = 9.1 Hz), 1.53 (s, 9H, H_{14}), 1.42 (s, 9H, H_{17}).

^{13}C NMR (100 MHz, DMSO-d$_6$) δ 180.1 (C_5), 163.1 (C_{11}), 153.5 (C_{15}), 152.4 (C_{12}), 151.9 (C_{10}), 145.7 (C_6), 140.1 (C_8), 138.1 (C_1), 125.9 (C_3), 123.4 (C_9), 120.0 (C_2), 115.7 (C_7), 84.6 (C_{16}), 80.0 (C_{13}), 28.4 (C_{14}), 28.1 (C_{17}).

HRMS (m/z ESI⁺): m/z found 521.1747 $[M+1]^+$; $C_{23}H_{30}ClN_6O_4S$ requires 521.1738

1-{4-[1,3-di(*tert*-butoxycarbonyl)guanidino]phenyl}-3-(1H-benzo[d]imidazol-2-yl)thiourea, 25D.[11]

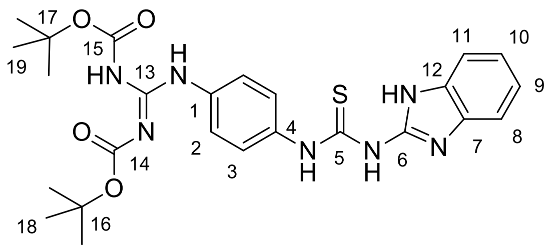

Yield: 48%

^{1}H NMR (600 MHz, DMSO-d$_6$) δ 12.38 (s, 1H, ArNH), 11.47 (s, 1H, NHPhNHC=S), 9.97 (s, 1H, NHPhNHC=S), 9.96 (s, 1H, BocNH), 7.91 (d, 2H, H_3, $J_{2\text{-}3}$ = 8.5 Hz), 7.51 (d, 2H, H_2, $J_{2\text{-}3}$ = 8.5 Hz), 7.32 (d, 1H, H_8, $J_{8\text{-}9}$ = 7.7 Hz), 7.19 (d, 1H, H_{11}, $J_{10\text{-}11}$ = 8.1 Hz), 7.07 (m, 2H, H_9), 6.98 (m, 1H, H_{10}), 5.57 (s, 1H, NH(N=)C-NHAr), 1.53 (s, 9H, H_{18}), 1.43 (s, 9H, H_{19}).

^{13}C NMR (150 MHz, DMSO-d$_6$) δ 166.7 (C_5), 163.2 (C_6), 155.9 (C_{13}), 153.4 (C_{14}), 152.6 (C_{15}), 138.7 (C_{12}), 138.0 (C_7), 132.1 (C_4), 131.3 (C_4), 123.5 (C_2), 122.6 (C_9), 122.2 (C_{10}), 121.8 (C_3), 114.7 (C_{11}), 110.8 (C_8), 82.8 (C_{16}), 79.2 (C_{17}), 28.4 (C_{18}), 28.2 (C_{19}).

HRMS (m/z ESI⁺): m/z found 526.2235 $[M+1]^+$; $C_{25}H_{32}N_7O_4S$ requires 526.2236

1-{4-[1,3-di(*tert*-butoxycarbonyl)guanidino]phenyl}-3-{4-[1-(*tert*-butoxycarbonylethylamino) triazolo]phenyl}thiourea, Boc26D

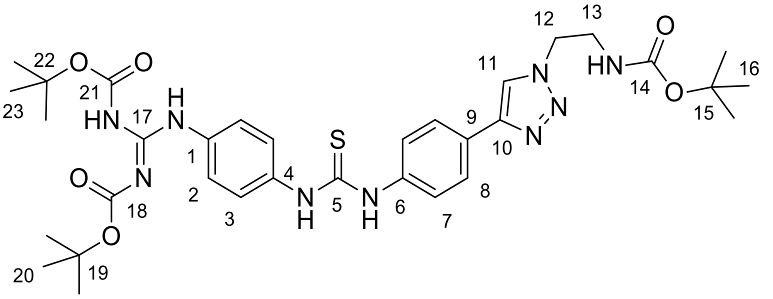

Yield: 32%

^{1}H NMR (600 MHz, CD$_3$OD) δ 11.44 (s, 1H, ArNH), 9.98 (s, 1H, BocNH), 9.89 (s, 1H, NHPhNHC=S), 9.84 (s, 1H, NHPhNHC=S), 8.74 (s, 1H, H_{11}), 7.79 (d, 2H, H_2, $J_{2\text{-}3}$ = 8.7 Hz), 7.58 (d, 2H, H_2, $J_{2\text{-}3}$ = 8.7 Hz), 7.51 (d, 2H, H_7, $J_{7\text{-}8}$ = 9.0 Hz), 7.47 (d, 1H, H_7, $J_{8\text{-}7}$ = 9.0 Hz), 7.05 (t, 1H, CH_2NHBoc, $J_{NH\text{-}13}$ = 5.6

Hz), 4.43 (t, 2H, H_{12}, $J_{12\text{-}13}$ = 6.0 Hz), 3.43 (q, 2H, H_{13}, $J_{12\text{-}13}$ = 5.9 Hz, $J_{NH\text{-}13}$ = 5.9 Hz), 1.53 (s, 9H, H_{23}), 1.41 (s, 9H, H_{20}), 1.35 (s, 9H, H_{16}).

^{13}C NMR (150 MHz, CD_3OD) δ 179.4 (C_5), 162.7 (C_{17}), 155.6 (C_{21}), 152.8 (C_{18}), 152.1 (C_{14}), 145.9 (C_1), 138.9 (C_4), 136.2 (C_6), 133.0 (C_9), 126.9 (C_{10}), 125.2 (C_2), 123.9 (C_3), 123.7 (C_7), 122.9 (C_8), 122.9 (C_8), 121.2 (C_{11}), 83.3 (C_{22}), 78.8 (C_{19}), 78.0 (C_{15}), 49.2 (C_{12}), 40.1 (C_{12}), 28.1 (C_{16}), 27.8 (C_{20}), 27.6 (C_{23}).

HRMS (m/z ESI$^+$): m/z found 696.3277 $[M+1]^+$; $C_{33}H_{46}N_9O_6S$ requires 696.3287

2.4. Synthesis and Characterisation of trifluoroacetate salts of *N,N'*-[(4-Guanidinophenyl)(aryl)] thioureas

Deprotection of the *N,N'*-(di-Boc-guanidinophenyl)(aryl) thioureas (**2D-6D/Boc8D/9D-25D/ Boc26D**) was performed according to **Method D**. Compound **1** has already been reported by us in reference 4. Compounds **2** and **20** have already been reported by us in reference 2.

1,3-bis(4-guanidinophenyl)thiourea bistrifluoroacetate salt, 1.[12]

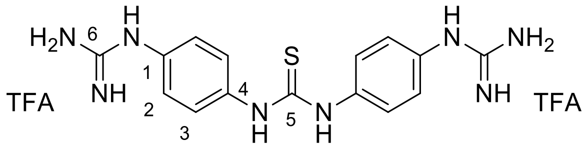

Yield: 92%

^{1}H NMR (400 MHz, CD_3OD) δ 7.56 (d, 2H, H_2, $J_{2\text{-}3}$ = 8.8 Hz), 7.28 (d, 2H, H_3, $J_{2\text{-}3}$ = 8.8 Hz).

^{13}C NMR (100 MHz, CD_3OD) δ 181.5 (C_5), 157.0 (C_6), 138.6 (C_1), 131.9 (C_4), 126.3 (C_2), 126.1 (C_3).

HRMS (m/z ESI$^+$): m/z found 343.1446 $[M+1]^+$; $C_{15}H_{19}N_8S$ requires 343.1448

1-(4-Guanidinophenyl)-3-phenylthiourea trifluoroacetate salt, 2.[11]

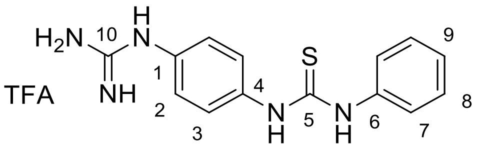

Yield: >98%

^{1}H NMR (400 MHz, D_2O) δ 7.43 (t, 2H, H_8, $J_{8\text{-}7}$ = $J_{8\text{-}9}$ = 7.8 Hz) 7.39 - 7.27 (m, 7H, H_2, H_3, H_7, H_9).

^{13}C NMR (150 MHz, D_2O) δ 180.0 (C_5), 137.0 (C_4), 134.1 (C_1), 133.0 (C_6), 129.7 (C_2), 128.24 (C_3), 127.8 (C_9), 127.0 (C_8), 126.4 (C_7).

HRMS (m/z ESI$^+$): m/z found 286.1131 $[M+1]^+$; $C_{14}H_{16}N_5S$ requires 286.1126

1-(4-fluorophenyl)-3-(4-guanidinophenyl)thiourea trifluoroacetate salt, 3

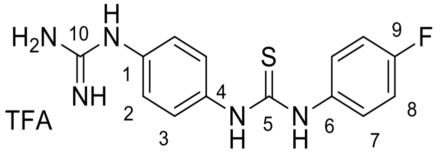

Yield: >98%

^{1}H NMR (400 MHz, CD$_3$OD) δ 7.55 (d, 2H, H$_2$, J_{2-3} = 8.8 Hz), 7.45 (dd, 2H, H$_7$, J_{7-F} = 4.9, J_{8-7} = 9.1 Hz), 7.30 (d, 2H, H$_3$, J_{2-3} = 8.8 Hz), 7.14 (t, 2H, H$_8$, J_{8-F} = J_{8-7} = 8.8 Hz).

^{13}C NMR (100 MHz, CD$_3$OD) δ 181.6 (C$_5$), 160.8 (d C$_9$, J_{9-F} = 244.0 Hz), 157.1 (C$_{10}$), 138.9 (C$_4$), 134.7 (d, C$_6$, J_{6-F} = 2.8 Hz), 131.9 (C$_4$), 127.0 (d, C$_7$, J_{7-F} = 8.3 Hz), 126.6 (C$_2$), 126.2 (C$_3$), 115.2 (d, C$_8$, J_{8-F} = 22.6 Hz).

HRMS (m/z ESI$^+$): m/z found 304.1033 [M+1]$^+$; $C_{14}H_{15}FN_5S$ requires 304.1027

1-(4-bromophenyl)-3-(4-guanidinophenyl)thiourea trifluoroacetate salt, 4

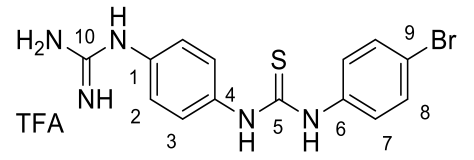

Yield: >98%

^{1}H NMR (400 MHz, CD$_3$OD) δ 7.55 (d, 2H, H$_8$, J_{7-8} = 8.7 Hz), 7.50 (d, 2H, H$_2$, J_{2-3} = 8.9 Hz), 7.41 (d, 2H, H$_3$, J_{2-3} = 8.9 Hz), 7.27 (d, 2H, H$_7$, J_{7-8} = 8.7 Hz).

^{13}C NMR (100 MHz, CD$_3$OD) δ 182.5 (C$_5$), 158.4 (C$_{10}$), 140.2 (C$_9$), 139.5 (C$_1$), 133.3 (C$_6$), 132.9 (C$_2$), 127.8 (C$_8$), 127.6 (C$_3$), 119.5 (C$_4$).

HRMS (m/z ESI$^+$): m/z found 464.0212 [M+1]$^+$; $C_{14}H_{15}BrN_5S$ requires 464.0227

1-(4-guanidinophenyl)-3-(p-tolyl)thiourea trifluoroacetate salt, 5

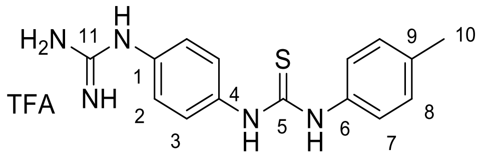

Yield: >98%

^{1}H NMR (400 MHz, CD$_3$OD) δ 7.53 (d, 2H, H$_2$, J_{2-3} = 8.8 Hz), 7.31 (d, 2H, H$_8$, J_{7-8} = 8.2 Hz), 7.28 (d, 2H, H$_3$, J_{2-3} = 8.8 Hz), 7.22 (d, 2H, H$_7$, J_{7-8} = 8.2 Hz), 2.36 (s, 3H, H$_{10}$).

^{13}C NMR (100 MHz, CD$_3$OD) δ 181.1 (C$_5$), 157.1 (C$_{11}$), 139.0 (C$_9$), 135.8 (C$_4$), 135.7 (C$_1$), 131.9 (C$_6$), 129.3 (C$_7$), 126.7 (C$_2$), 126.2 (C$_3$), 124.6 (C$_8$), 19.6 (C$_{10}$).

HRMS (m/z ESI$^+$): m/z found 300.1269 [M+1]$^+$; $C_{15}H_{18}N_5S$ requires 300.1269

1-(4-(tert-butyl)phenyl)-3-(4-guanidinophenyl)thiourea trifluoroacetate salt, 6

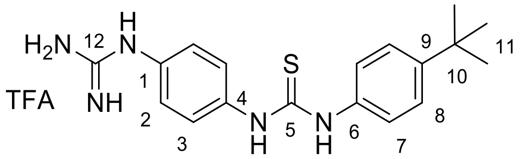

Yield: >98%

^{1}H NMR (400 MHz, CD_3OD) δ 7.55 (d, 2H, H_2, J_{2-3} = 8.9 Hz), 7.46 (d, 2H, H_8, J_{7-8} = 8.6 Hz), 7.36 (d, 2H, H_3, J_{2-3} = 8.9 Hz), 7.28 (d, 2H, H_8, J_{7-8} = 8.6 Hz), 1.35 (s, 9H, H_{11}).

^{13}C NMR (100 MHz, CD_3OD δ 181.0 (C_5), 157.2 (C_{12}), 139.1 (C_9), 135.7 (C_1), 133.3 (C_6), 131.9 (C_4), 126.8 (C_2), 126.2 (C_3), 125.7 (C_7), 124.2 (C_8), 34.0 (C_{10}), 30.3 (C_{11}).

HRMS (m/z ESI$^+$): m/z found 342.1751 $[M+1]^+$; $C_{18}H_{24}N_5S$ requires 342.1747

1-(4-aminophenyl)-3-(4-guanidinophenyl)thiourea trifluoroacetate salt, 8

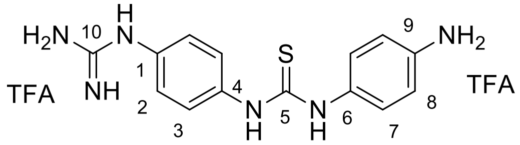

Yield: >98%

^{1}H NMR (400 MHz, CD_3OD) δ 7.69 (d, 2H, H_7, J_{7-8} = 8.9 Hz), 7.60 (d, 2H, H_3, J_{2-3} = 8.7 Hz), 7.37 (d, 2H, H_8, J_{7-8} = 8.9 Hz), 7.30 (d, 2H, H_2, J_{2-3} = 8.7 Hz).

^{13}C NMR (100 MHz, CD_3OD) δ 181.5 (C_5), 157.0 (C_{10}), 138.8 (C_1), 131.9 (C_4), 127.0 (C_2), 126.4 (C_7), 124.1 (C_3), 125.7 (C_8), 121.3 (C_9), 119.3 (C_6).

HRMS (m/z ESI$^+$): m/z found 301.1241 $[M+1]^+$; $C_{14}H_{17}N_6S$ requires 301.1230

1-(4-ethylphenyl)-3-(4-guanidinophenyl)thiourea trifluoroacetate salt, 9

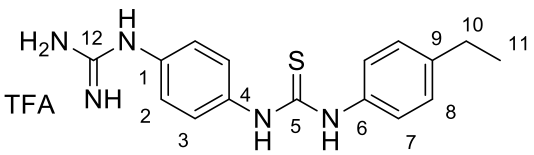

Yield: >98%

^{1}H NMR (400 MHz, CD_3OD) δ 7.54 (d, 2H, H_2, $J_{2\text{-}3}$ = 8.8 Hz), 7.34 (d, 2H, H_8, $J_{8\text{-}7}$ = 8.4 Hz), 7.28 (d, 2H, H_3, $J_{2\text{-}3}$ = 8.8 Hz), 7.26 (d, 2H, H_7, $J_{8\text{-}7}$ = 8.4 Hz), 2.68 (q, 2H, H_{10}, $J_{10\text{-}11}$ = 7.8 Hz), 1.26 (t, 3H, H_{11}, $J_{10\text{-}11}$ = 7.8 Hz).

^{13}C NMR (100 MHz, CD_3OD) δ 181.1 (C_5), 157.1 (C_{12}), 142.3 (C_9), 139.1 (C_4), 135.9 (C_1), 131.9 (C_6), 128.2 (C_7), 126.8 (C_2), 126.2 (C_3), 124.6 (C_8), 28.0 (C_{10}), 14.7 (C_{11}).

HRMS (m/z ESI$^+$): m/z found 314.1451 $[M+1]^+$; $C_{16}H_{20}N_5S$ requires 314.1434

1-(4-benzylphenyl)-3-(4-guanidinophenyl)thiourea trifluoroacetate salt, 10

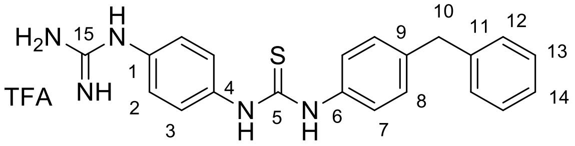

Yield: >98%

^{1}H NMR (400 MHz, CD_3OD) δ 7.53 (d, 2H, H_2, $J_{2\text{-}3}$ = 8.5 Hz), 7.37 - 7.15 (m, 11H, H_3, H_7, H_8, H_{12}, H_{13}, H_{14}), 3.99 (s, 3H, H_{10}).

^{13}C NMR (100 MHz, CD_3OD) δ 181.1 (C_5), 157.1 (C_{15}), 141.1 (C_9), 139.5 (C_{11}), 139.1 (C_4), 136.3 (C_1), 131.9 (C_6), 129.2 (C_8), 128.5 (C_{12}), 128.1 (C_{13}), 126.8 (C_2), 126.2 (C_3), 125.7 (C_{14}), 124.6 (C_7), 40.9 (C_{10}).

HRMS (m/z ESI$^+$): m/z found 376.1599 $[M+1]^+$; $C_{21}H_{22}N_5S$ requires 376.1591.

1-(4-guanidinomphenyl)-3-(4-phenoxyphenyl)thiourea trifluroacetate salt, 11

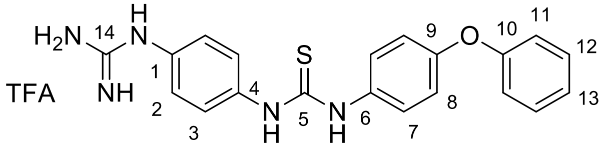

Yield: >98%

^{1}H NMR (600 MHz, CD_3OD) δ 7.55 (d, 2H, H_2, $H_{2\text{-}3}$ = 8.7 Hz), 7.43 – 7.34 (m, 4H, H_7, H_{12}), 7.29 (d, 2H, H_3, $H_{2\text{-}3}$ = 8.7 Hz), 7.14 (dt, 1H, H_{13}, $J_{12\text{-}13}$ = 7.4 Hz, $J_{11\text{-}13}$ = 1.2 Hz), 7.06 – 7.01 (m, 4H, H_8, H_{11}).

^{13}C NMR (150 MHz, CD_3OD) δ 179.9 (C_5), 157.2 (C_{14}), 157.0 (C_{10}), 155.5 (C_9), 139.0 (C_1), 133.6 (C_6), 131.9 (C_4), 129.5 (C_7), 126.7 (C_2), 126.6 (C_{12}), 126.2 (C_3), 123.2 (C_{13}), 118.8 (C_{11}), 118.5 (C_8).

HRMS (m/z ESI$^+$): m/z found 378.1391 $[M+1]^+$; $C_{20}H_{20}N_5OS$ requires 378.1384

1-(4-ethynylphenyl)-3-(4-guanidinophenyl)thiourea trifluroacetate salt, 12

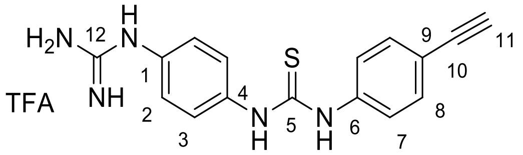

Yield: >98%

^{1}H NMR (400 MHz, CD_3OD) δ 746 – 7.35 (m, 4H, H_2, H_8), 7.06 (d, 2H, H_3, $J_{2\text{-}3}$ = 8.5 Hz), 6.71 (d, 2H, H_7, $J_{7\text{-}8}$ = 7.5 Hz), 3.42 (s, 3H, H_{11}).

^{13}C NMR (100 MHz, CD_3OD) δ 180.3 (C_5), 157.3 (C_{12}), 139.6 (C_1), 132.8 (C_6), 131.9 (C_7), 126.5 (C_2, C_3), 124.0 (C_4), 118.9 (C_9), 115.2 (C_8), 82.7 (C_{10}), 77.0 (C_{11}),

HRMS (m/z ESI$^+$): m/z found 310.1112 $[M+1]^+$; $C_{16}H_{16}N_5S$ requires 310.1121.

1-(4-guanidinophenyl)-3-(3-methoxyphenyl)thiourea trifluoroacetate salt, 13

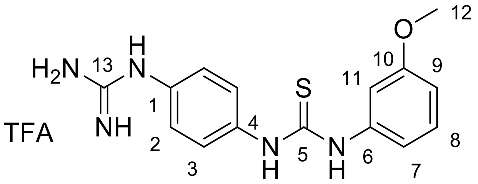

Yield: >98%

^{1}H NMR (400 MHz, CD_3OD) δ 7.55 (d, 2H, H_2, $J_{2\text{-}3}$ = 8.7 Hz), 7.30 (t, 1H, H_8, $J_{7\text{-}8}$ = 8.0 Hz, $J_{8\text{-}9}$ = 8.0 Hz), 7.29 (d, 1H, H, $J_{2\text{-}3}$ = 8.0 Hz), 7.11 (t, 1H, H_{11}, $J_{11\text{-}7}$ = 2.1 Hz, $J_{11\text{-}9}$ = 8.0 Hz), 7.00 (dd, 1H, H_7, $J_{7\text{-}8}$ = 8.0 Hz, $J_{7\text{-}11}$ = 2.4 Hz), 6.82 (dd, 1H, H_9, $J_{9\text{-}8}$ = 8.0 Hz, $J_{9\text{-}11}$ = 2.4 Hz), 3.82 (s, 3H, H_{12}).

^{13}C NMR (100 MHz, CD_3OD) δ 180.9 (C_5), 160.4 (C_{10}), 157.1 (C_{13}), 139.1 (C_1), 133.4 (C_6), 131.9 (C_4), 129.5 (C_8), 126.7 (C_2), 126.2 (C_3), 116.2 (C_7), 111.1 (C_9), 109.9 (C_{11}), 54.4 (C_{12}).

HRMS (m/z ESI$^+$): m/z found 316.1219 $[M+1]^+$; $C_{15}H_{18}N_5OS$ requires 316.1227

1-(3,4-dimethylphenyl)-3-(4-guanidinophenyl)thiourea trifluoroacetate salt, 14

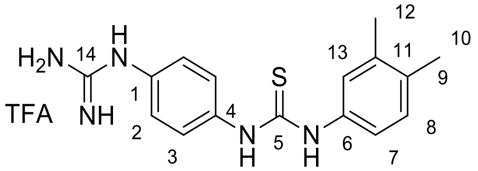

Yield: >98%

^{1}H NMR (400 MHz, CD_3OD) δ 7.53 (d, 2H, H_2, J_{2-3} = 8.7 Hz), 7.27 (d, 2H, H_3, J_{2-3} = 8.7 Hz), 7.20 - 7.10 (m, 3H, H_7, H_8, H_{13}), 2.29 (s, 3H, H_{12}), 2.28 (s, 3H, H_{10}).

^{13}C NMR (100 MHz, CD_3OD) δ 180.9 (C_5), 157.2 (C_{14}), 139.1 (C_1), 137.3 (C_9), 135.8 (C_4), 134.5 (C_{11}), 131.9 (C_6), 129.8 (C_8), 126.9 (C_2), 126.2 (C_3), 125.8 (C_8), 122.1 (C_7), 18.5 (C_{10}), 17.9 (C_{12}).

HRMS (m/z ESI$^+$): m/z found 314.1447 $[M+1]^+$; $C_{16}H_{20}N_5S$ requires 314.1434

1-(4-bromo-3-methylphenyl)-3-(4-guanidinophenyl)thiourea trifluoroacetate salt, 15

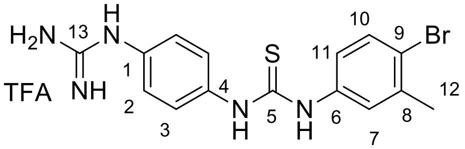

Yield: >98%

^{1}H NMR (400 MHz, CD_3OD) δ 7.55 (d, 2H, H_2, J_{2-3} = 8.7 Hz), 7.53 (d, 2H, H_{10}, J_{10-11} = 8.2 Hz), 7.41 (d, 1H, H_7, J_{7-11} = 2.7 Hz), 7.29 (d, 1H, H_3, J_{2-3} = 8.7 Hz), 7.24 (dd, 1H, H_{11}, J_{10-11} = 8.7 Hz, J_{7-11} = 2.7 Hz), 2.41 (s, 3H, H_{12}).

^{13}C NMR (100 MHz, CD_3OD) δ 181.1 (C_5), 157.2 (C_{13}), 138.9 (C_1), 138.2 (C_9), 138.1 (C_6), 133.5 (C_4), 132.2 (C_{10}), 126.5 (C_2, C_7), 126.2 (C_3), 123.4 (C_{11}), 120.7 (C_8), 21.6 (C_{12}).

HRMS (m/z ESI$^+$): m/z found 378.0375 $[M+1]^+$; $C_{15}H_{17}BrN_5S$ requires 378.0383

1-(4-chloro-3-(trifluoromethyl)phenyl)-3-(4-guanidinophenyl)thiourea trifluoroacetate salt, 16

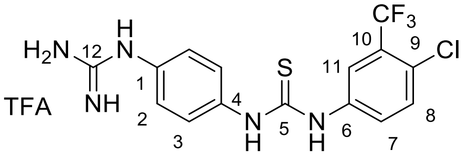

Yield: >98%

^{1}H NMR (400 MHz, CD_3OD) δ 8.02 (d, 1H, H_{11}, J_{11-7} = 1.9 Hz), 7.72 (dd, 1H, H_7, J_{8-7} = 8.8 Hz, J_{11-7} = 1.9 Hz), 7.60 – 7.53 (m, 3H, H_2, H_8), 7.28 (d, 2H, H_2, J_{2-3} = 8.6 Hz).

^{13}C NMR (100 MHz, CD_3OD) δ 181.4 (C_5), 157.0 (C_{12}), 138.6 (C_1), 133.3 (C_6), 131.9 (C_4), 131.4 (C_8), 128.3 (C_7), 126.8 (C_9), 126.1 (C_2, C_3), 124.0 (q, <u>C</u>F_3, J_{C-F} = 274.0 Hz), 122.9 (C_4).

HRMS (m/z ESI$^+$): m/z found 388.0611 $[M+1]^+$; $C_{15}H_{14}ClF_3N_5S$ requires 388.0606

1-(3,4-dimethoxyphenyl)-3-(4-guanidinophenyl)thiourea trifluoroacetate salt, 17

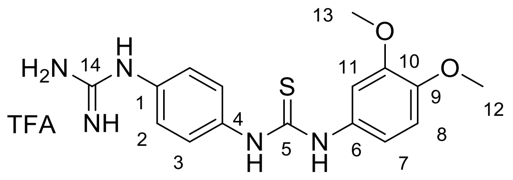

Yield: >98%

^{1}H NMR (400 MHz, CD_3OD) δ 7.53 (d, 2H, H_2, $J_{2\text{-}3}$ = 8.7 Hz), 7.28 (d, 2H, H_3, $J_{2\text{-}3}$ = 8.7 Hz), 7.10 (d, 1H, H_{11}, $J_{11\text{-}7}$ = 2.3 Hz), 6.98 (d, 1H, H_8, $J_{7\text{-}8}$ = 8.6 Hz), 6.93 (dd, 1H, H_7, $J_{7\text{-}8}$ = 8.6 Hz, $J_{7\text{-}11}$ = 2.3 Hz), 3.85 (s, 6H, H_{12}, H_{13}).

^{13}C NMR (100 MHz, CD_3OD) δ 181.1 (C_5), 157.1 (C_{14}), 149.3 (C_9), 147.7 (C_{10}), 139.0 (C_1), 132.0 (C_4), 131.3 (C_6), 126.9 (C_2), 126.2 (C_3), 117.4 (C_8), 111.7 (C_7), 109.8 (C_{11}), 55.2 (C_{13}), 55.1 (C_{12}).

HRMS (m/z ESI$^+$): m/z found 346.1335 $[M+1]^+$; $C_{16}H_{20}N_5O_2S$ requires 346.1333

1-(3,5-bis(trifluoromethyl)phenyl)-3-(4-guanidinophenyl)thiourea trifluoroacetate salt, 18

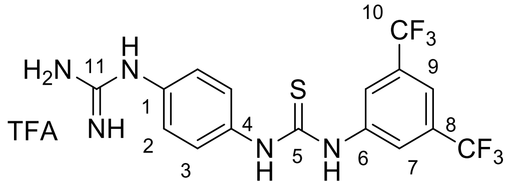

Yield: >98%

^{1}H NMR (400 MHz, CD_3OD) δ 8.24 (s, 2H, H_7), 7.72 (s, 1H, H_9), 7.62 (d, 2H, H_2, $J_{2\text{-}3}$ = 8.6 Hz), 7.32 (d, 2H, H_3, $J_{2\text{-}3}$ = 8.6 Hz).

^{13}C NMR (100 MHz, CD_3OD) δ 181.3 (C_5), 157.0 (C_{11}), 141.5 (C_6), 138.4 (C_1), 132.0 (C_4), 131.3 (q, C_8, $J_{8\text{-}F}$ = 34.5 Hz), 126.1 (C_2, C_3), 123.3 (C_7), 123.0 (q, C_{10}, $J_{10\text{-}F}$ = 224.0 Hz), 117.2 (C_9).

HRMS (m/z ESI$^+$): m/z found 422.0881 $[M+1]^+$; $C_{16}H_{14}F_6N_5S$ requires 422.0869

1-(4-guanidinophenyl)-3-(5,6,7,8-tetrahydronaphthalen-2-yl)thiourea trifluoroacetate salt, 19

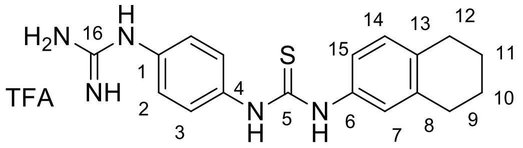

Yield: >98%

^{1}H NMR (400 MHz, CD_3OD) δ 7.52 (d, 2H, H_2, $J_{2\text{-}3}$ = 8.9 Hz), 7.28 (d, 2H, H_3, $J_{2\text{-}3}$ = 8.9 Hz), 7.12 – 7.08 (m, 3H, H_7, H_{14}, H_{15}), 2.79 - 2.66 (m, 4H, H_9, H_{12}), 1.84 - 1.75 (m, 4H, H_{10}, H_{11}).

^{13}C NMR (140 MHz, CD_3OD) δ 180.9 (C_5), 157.1 (C_{16}), 139.1 (C_1), 137.8 (C_8), 135.4 (C_6), 135.1 (C_{13}), 131.9 (C_4), 129.4 (C_7), 126.8 (C_2), 126.2 (C_3), 125.1 (C_{14}), 122.0 (C_{15}), 29.0 (C_9), 28.6 (C_{12}), 22.9 (C_{11}), 22.8 (C_{10}).

HRMS (m/z ESI$^+$): m/z found 340.1578 $[M+1]^+$; $C_{18}H_{22}N_5S$ requires 340.1591

1-(4-Guanidinophenyl)-3-(2-pyridinyl)thiourea trifluoroacetate salt, 20.[11]

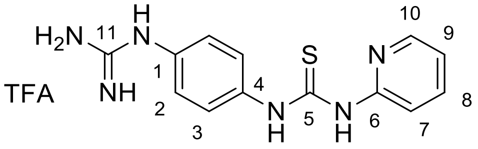

Yield: >98%

^{1}H NMR (400MHz, CD_2Cl_2) δ 13.65 (s, 1H, PyN<u>H</u>), 12.12 (s, 1H, NHPhN<u>H</u>C=S), 9.45 (s, 1H, N<u>H</u>PhNHC=S), 8.25 (t, 1H, H_8, $J_{8\text{-}9}$ = $J_{7\text{-}8}$ = 7.8 Hz), 8.13 (d, 1H, H_{10}, $J_{10\text{-}9}$ = 7.8 Hz), 7.74 (d, 2H, H_7, $J_{7\text{-}8}$ = 7.8 Hz), 7.66 (d, 2H, H_2, $J_{2\text{-}3}$ = 8.4 Hz), 7.44 (t, 1H, H_9, $J_{8\text{-}9}$ = $J_{10\text{-}9}$ = 7.8 Hz), 7.30 (d, 2H, H_3, $J_{2\text{-}3}$ = 8.4 Hz), 6.80 (br, 4H, $^+$N<u>H</u>$_2$=C-N<u>H</u>$_2$).

^{13}C NMR (100Mhz, CD_2Cl_2) δ 180.0 (C_5), 157.2 (C_{11}), 153.8 (C_6), 148.2 (C_{10}), 138.3 (C_8), 134.5 (C_1), 128.4 (C_4), 127.3 (C_3), 117.9 (C_9), 166.0 (C_2), 113.0 (C_7).

HRMS (m/z ESI$^+$): m/z found 287.1082 $[M+1]^+$; $C_{13}H_{15}N_5S$ requires 287.1079

1-(4-guanidinophenyl)-3-(3-methylpyridin-2-yl)thiourea trifluoroacetate salt, 21

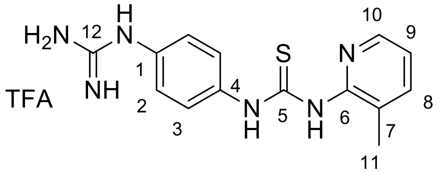

Yield: >98%

^{1}H NMR (400 MHz, CD_3OD) δ 8.21 (d, 1H, H_{10}, $J_{10\text{-}9}$ = 5.0 Hz), 7.80 – 7.73 (m, 3H, H_3, H_8), 7.3 (d, 2H, H_2, $J_{2\text{-}3}$ = 8.7 Hz), 7.14 (dd, 1H, H_9, $J_{8\text{-}9}$ = 7.5 Hz, $J_{10\text{-}9}$ = 5.1 Hz), 2.40 (s, 3H, H_{11}).

^{13}C NMR (100 MHz, CD_3OD) δ 180.9 (C_5), 158.3 (C_{12}), 152.6 (C_6), 143.7 (C_{10}), 142.1 (C_8), 139.6 (C_1), 133.9 (C_4), 127.5 (C_2), 127.4 (C_3), 123.3 (C_7), 120.4 (C_9), 16.7 (C_{11}).

HRMS (m/z ESI$^+$): m/z found 301.1228 $[M+1]^+$; $C_{14}H_{17}N_6S$ requires 301.1230

1-(4-guanidinophenyl)-3-(5-methylpyridin-2-yl)thiourea trifluoroacetate salt, 22

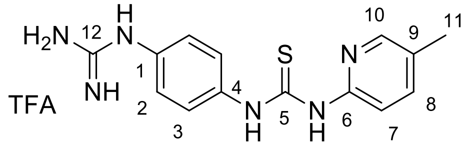

Yield: >98%

^{1}H NMR (400 MHz, CD_3OD) δ 8.11 (s, 1H, H_{10}), 7.76 – 7.67 (m, 3H, H_3), 7.30 (d, 2H, H_2, $J_{2\text{-}3}$ = 8.9 Hz), 7.07 (d, 1H, H_7, $J_{7\text{-}8}$ = 8.6 Hz), 2.30 (s, 3H, H_{11}).

^{13}C NMR (100 MHz, CD_3OD) δ 179.1 (C_5), 157.0 (C_{12}), 150.9 (C_6), 143.0 (C_{10}), 141.0 (C_8), 138.1 (C_1), 132.3 (C_4), 128.5 (C_9), 126.2 (C_2), 125.9 (C_3), 112.9 (C_7), 16.2 (C_{11}).

HRMS (m/z ESI$^+$): m/z found 301.1224 $[M+1]^+$; $C_{14}H_{17}N_6S$ requires 301.1230

1-(4-guanidinophenyl)-3-(6-methylpyridin-2-yl)thiourea trifluoroacetate salt, 23

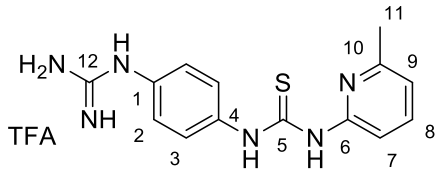

Yield: >98%

^{1}H NMR (400 MHz, CD_3OD) δ 7.84 (d, 2H, H_3, $J_{2\text{-}3}$ = 8.8 Hz), 7.71 (d, 1H, H_8, $J_{7\text{-}8}$ = $J_{8\text{-}9}$ = 8.4 Hz), 7.34 (d, 1H, H_2, $J_{2\text{-}3}$ = 7.8 Hz), 6.99 (d, 1H, H_7, $J_{7\text{-}8}$ = 8.4 Hz), 6.90 (d, 1H, H_9, $J_{8\text{-}9}$ = 8.4 Hz), 2.53 (s, 3H, H_{11}).

^{13}C NMR (100 MHz, CD_3OD) δ 179.3 (C_5), 157.0 (C_{12}), 155.0 (C_{10}), 153.1 (C_6), 139.4 (C_8), 138.6 (C_1), 132.0 (C_4), 126.1 (C_3), 125.6 (C_2), 117.4 (C_9), 109.3 (C_7), 22.5 (C_{11}).

HRMS (m/z ESI$^+$): m/z found 301.1233 $[M+1]^+$; $C_{14}H_{17}N_6S$ requires 301.1230.

1-(5-chloropyridin-2-yl)-3-(4-guanidinophenyl)thiourea trifluoroacetate salt, 24

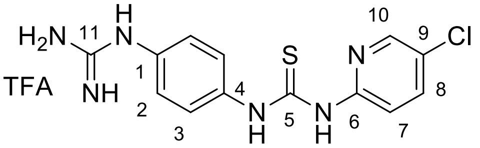

Yield: >98%

^{1}H NMR (400MHz, CD$_3$OD) δ 8.34 (d, 1H, H_{10}, $J_{10\text{-}8}$ = 2.6 Hz), 7.85 (dd, 1H, H_8, $J_{8\text{-}7}$ = 8.9 Hz, $J_{10\text{-}8}$ = 2.6 Hz), 7.77 (d, 2H, H_3, $J_{2\text{-}3}$ = 8.8 Hz), 7.34 (d, 2H, H_2, $J_{2\text{-}3}$ = 8.8 Hz), 7.13 (d, 1H, H_7, $J_{8\text{-}7}$ = 8.9 Hz).

^{13}C NMR (100 MHz, CD$_3$OD) δ 179.5 (C_5), 157.0 (C_{11}), 151.9 (C_9), 144.0 (C_{10}), 138.9 (C_8), 138.4 (C_1), 132.4 (C_4), 126.4 (C_3), 126.0 (C_2), 125.3 (C_6), 113.7 (C_7).

HRMS (m/z ESI$^+$): m/z found 321.0688 $[M+1]^+$; $C_{13}H_{14}ClN_6S$ requires 321.0684

1-(1H-benzo[d]imidazol-2-yl)-3-(4-guanidinophenyl)thioureatrifluoroacetate salt, 25

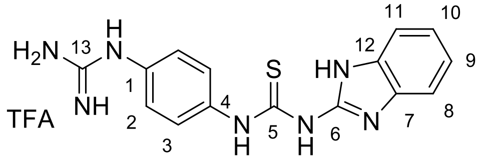

Yield: >98%

^{1}H NMR (400 MHz, CD$_3$OD) δ 7.80 (brd, 2H, H_3), 7.47 (brd, 2H, H_2), 7.35 – 7.16 (m, 4H, H_8, H_9, H_{10}, H_{11}).

^{13}C NMR (140 MHz, CD$_3$OD) δ 125.8 (C_2, C_3)

HRMS (m/z ESI$^+$): m/z found 326.1191 $[M+1]^+$; $C_{15}H_{16}N_7S$ requires 326.1183

1-(4-(1-(2-aminoethyl)-1H-1,2,3-triazol-4-yl)phenyl)-3-(4-guanidinophenyl)thiourea trifluoroacetate salt, 26

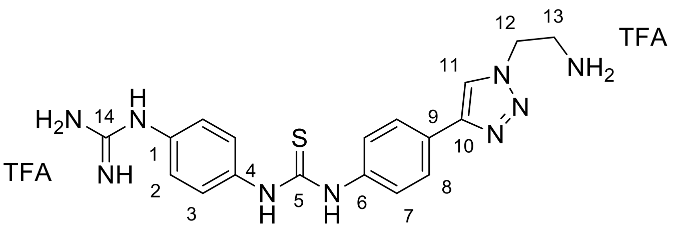

Yield: >98%

^{1}H NMR (400 MHz, CD_3OD) δ 8.43 (s, 1H, H_{11}), 7.85 (d, 2H, H_2, J_{2-3} = 8.5 Hz), 7.64 – 7.53 (m, 4H, H_7, H_8), 7.28 (d, 2H, H_3, J_{2-3} = 8.5 Hz), 4.80 (t, 2H, H_{12}, J_{12-13} = 5.6 Hz), 3.58 (t, 2H, H_{13}, J_{12-13} = 5.6 Hz).

^{13}C NMR (100 MHz, CD_3OD) δ 180.2 (C_5), 158.9 (C_{14}), 145.2 (C_9), 143.7 (C_1), 141.1 (C_4), 131.5 (C_6), 131.2 (C_{10}), 124.6 (C_2), 124.0 (C_3), 123.2 (C_8), 122.1 (C_7), 117.4 (C_{11}), 41.0 (C_{12}), 27.0 (C_{13}).

HRMS (m/z ESI$^+$): m/z found 396.1718 $[M+1]^+$; $C_{18}H_{22}N_9S$ requires 396.1714.

3. Biophysical experiments

3.1. UV thermal denaturation experiments

Thermal melting experiments were conducted with a Varian Cary 300 Bio spectrophotometer equipped with a 6×6 multicell temperature-controlled block. Temperatures were monitored with a thermistor inserted into a 1 cm quartz cuvette containing the same volume of water as in the sample cells. Absorbance changes at 260 nm were monitored from a range of 30 °C to 90 °C with a heating rate of 1 °C min^{-1} and a data collection rate of five points per °C. The salmon sperm DNA was purchased from Sigma Aldrich (extinction coefficient ε_{260}= 6600 M^{-1} cm^{-1}). A quartz cell with a 1 cm path length was filled with a 1 mL solution of DNA or DNA-compound complex. The DNA (150 µM base) and the compound solution (15 µM) were prepared in a phosphate buffer ([10 mM K_2HPO_4/KH_2PO_4], adjusted to pH 7) so that a compound to DNA base ratio of 0.1 was obtained. The thermal melting temperatures of the duplex or duplex-compound complex obtained from the first derivative of the melting curves are reported.

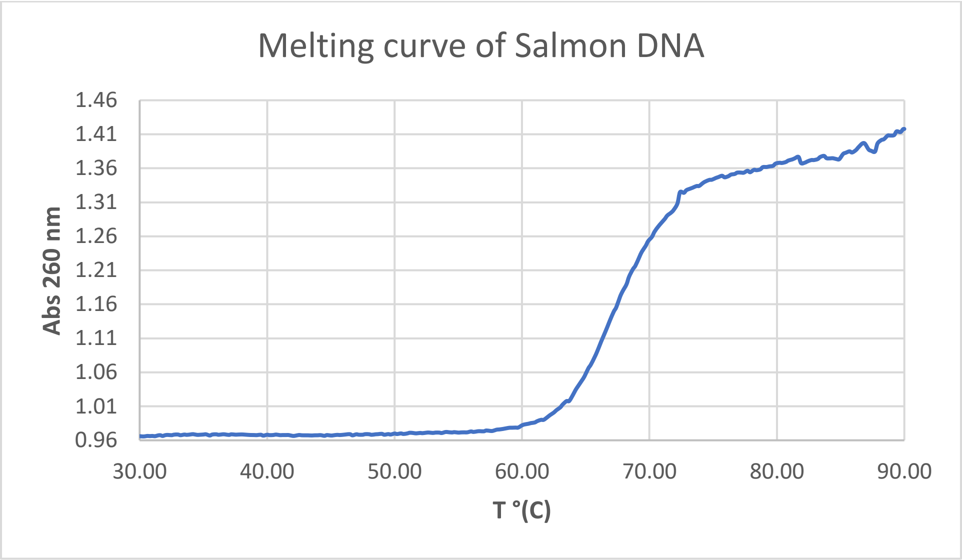

(a)

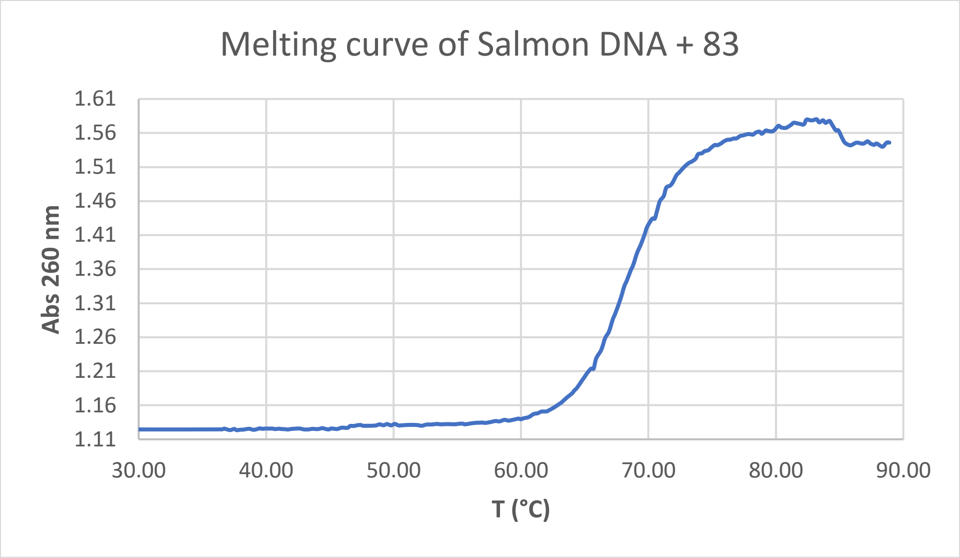

(b)

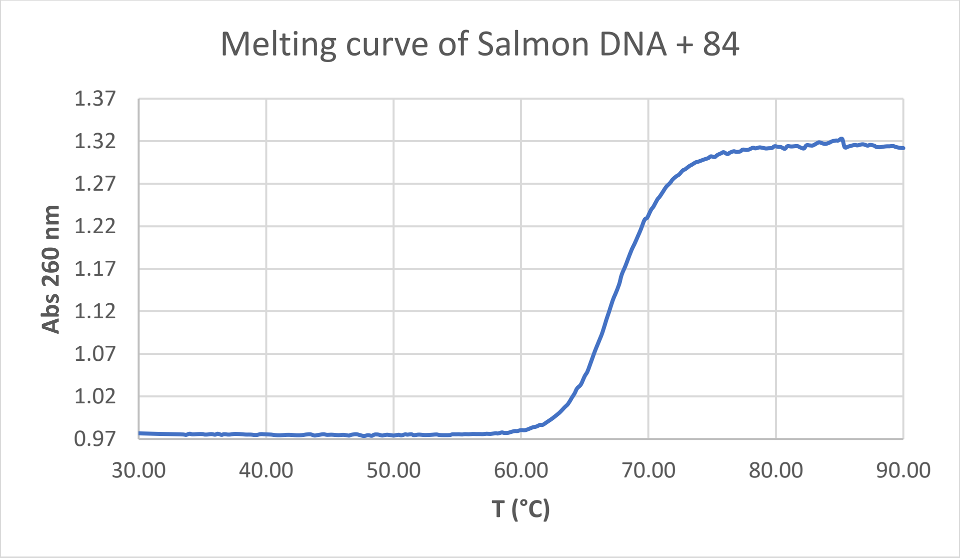

(c)

Figure S3. Salmon testes DNA melting curves obtained for (a) st-DNA alone, (b) st-DNA with compound 2 (83 in graph) and (c) st-DNA with compound 20 (84 in graph).

4. Cell viability assays

MCF-10 cells were seeded at $2.5x10^4$cells/well. The following day, cells were treated with either compound compounds **2** (0.1% (v/v) DMSO) at 0.1 µM, 1 µM, 5 µM, 10 µM, 25 µM, 50 µM and 100 µM in triplicate. After 72 hours 10% AlamarBlue (i.e. 20 µL) was added to each well. Plates were incubated in the dark for 6 hours at 37 °C. Fluorescence was measured using a SpectraMax Gemini plate reader (excitation wavelength 544 nm - emission wavelength 590 nm). The procedure was repeated three times. It appeared that no significant killing was achieved, indicating no cytotoxicity is exerted by the compound **2** on the MCF-10 cell line.

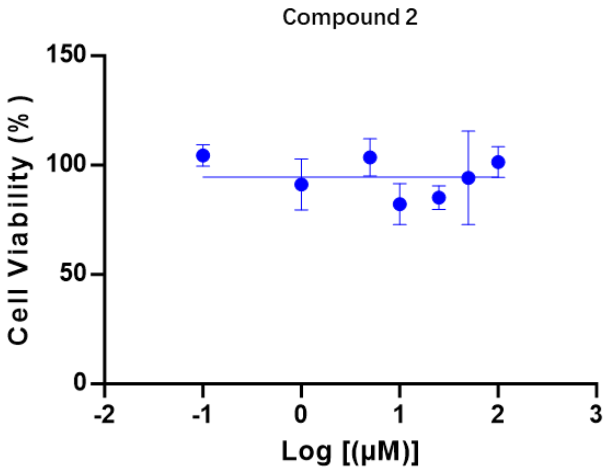

Figure S4. Dose-dependent cell viability curve obtained for compound 2 in the AlamarBlue assay.

5. Biochemical experiments

Virus: Vesicular stomatitis virus derived vector pseudotyped with SARS-CoV-2 spike or VSV glycoprotein (VSV-G)

Target cells: Vero (kidney, African green monkey, allow cathepsin L dependent entry), Calu-3 (lung, human, allow TMPRSS2 dependent entry)

Control inhibitors: Camostat (blocks TMPRSS2-dependent entry), chloroquine (blocks Cathepsin L dependent entry)

As a general guide, any compound that: (1) inhibits SARS-CoV-2 spike driven entry into Calu-3 cells, (2) does not inhibit SARS-CoV-2 spike driven entry into Vero cells, and (3) is inactive against VSV-G driven entry, is per definition a TMPRSS2 inhibitor

Structures of the compounds synthesized and fully characterized, including HPLC purity >90%. Codes as assigned by the German team and appearing in the biochemical results graphs.

**Codes in biochemical graphs**	**Number in manuscript**	**Structure**
**Hit compound A9 or MM300**	**1**	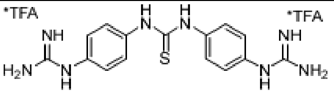
**MM223**	**2**	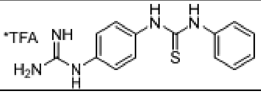
**MM286**	**3**	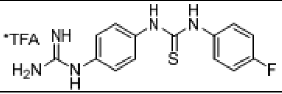
**MM295**	**4**	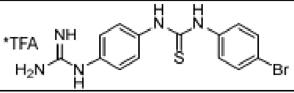
**MM296**	**5**	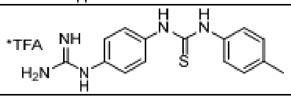
**MM298**	**6**	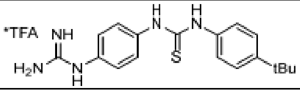
**MM535**	**8**	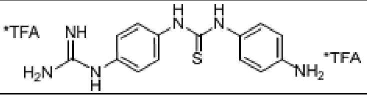
**MM533**	**9**	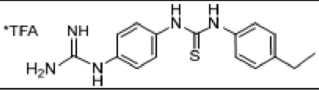
**MM287**	**10**	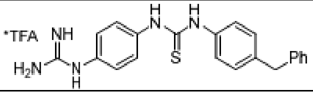

**MM288**	**11**	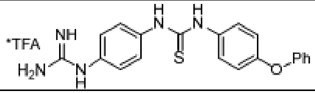
**MM540**	**12**	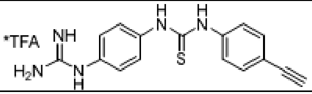
**MM303**	**13**	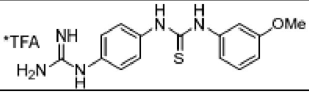
**MM289**	**14**	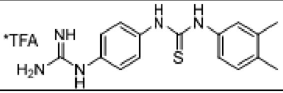
**MM290**	**15**	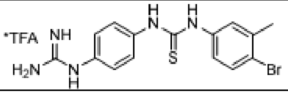
**MM294**	**16**	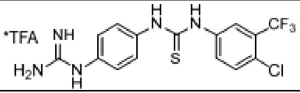
**MM285**	**17**	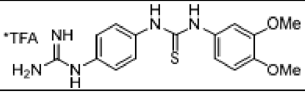
**MM301**	**18**	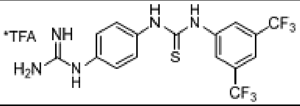
**MM297**	**19**	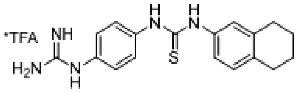
**MM552**	**20**	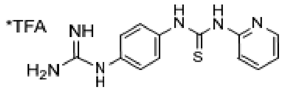
**MM293**	**21**	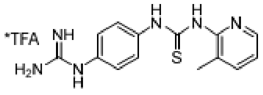
**MM292**	**22**	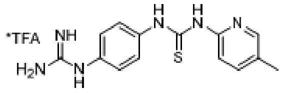
**MM291**	**23**	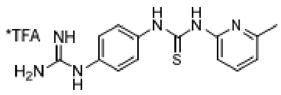
**MM302**	**24**	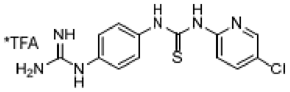
**MM299**	**25**	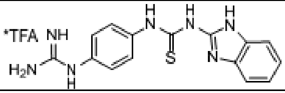
**MM536**	**26**	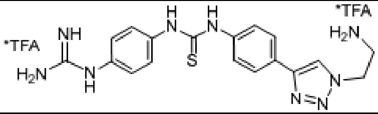

Luciferase assays

Vero76 cell infection with pseudotypes

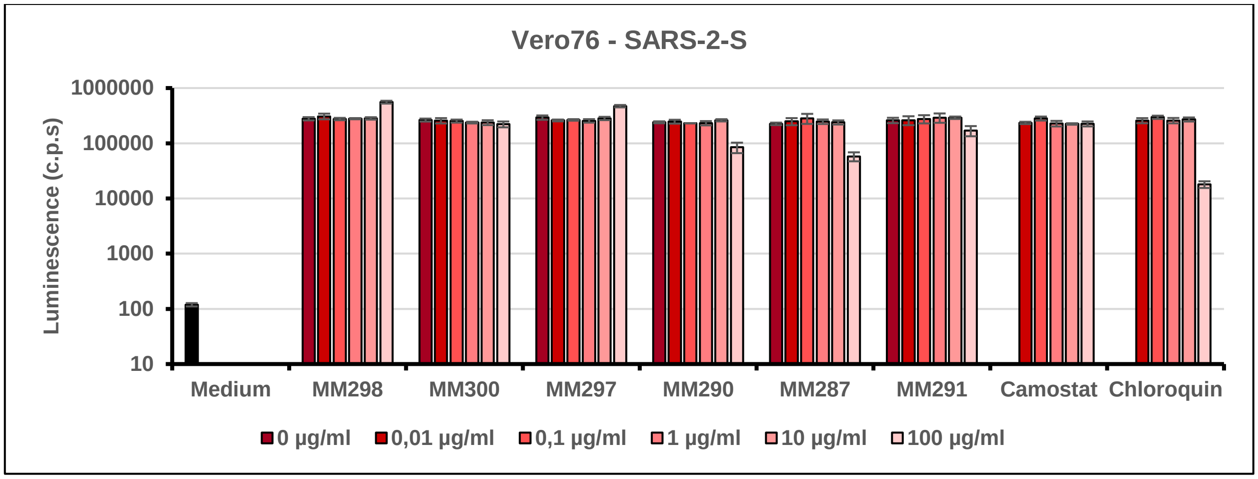

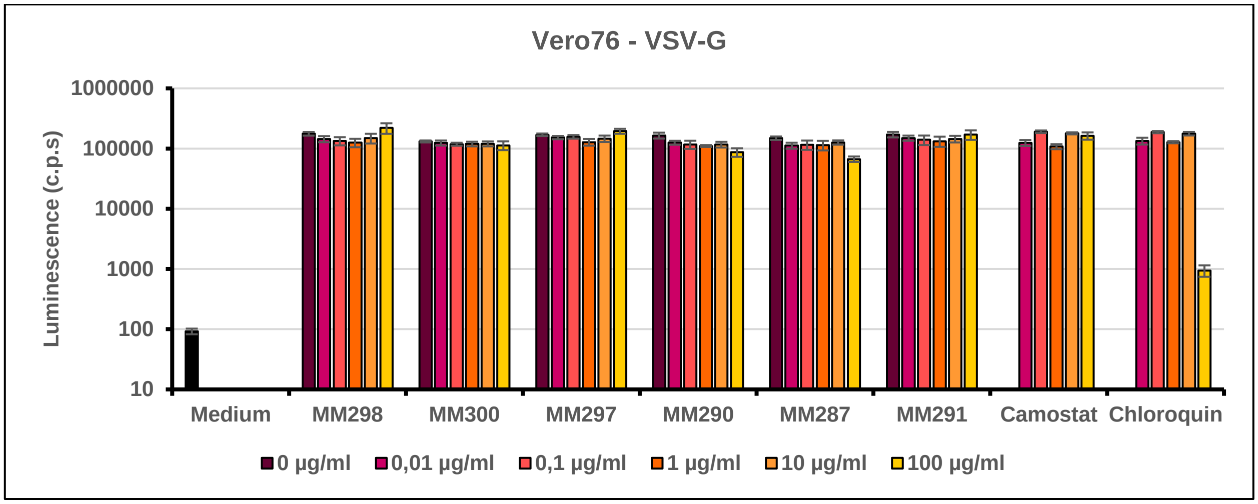

Figure S5. Vero76 cells seeded in 96-well plates were incubated with the indicated compounds at the indicated final concentrations and inoculated with VSV pseudoparticles bearing SARS-CoV-2 S protein (top panel) or VSV-G (bottom panel) or control inoculated with medium. Luciferase activity in cell lysates was determined at 16-18 h post infection. The results of a single experiment carried out with technical quadruplicates is shown. Error bars indicate SD.

Calu-3 cell infection with pseudotypes

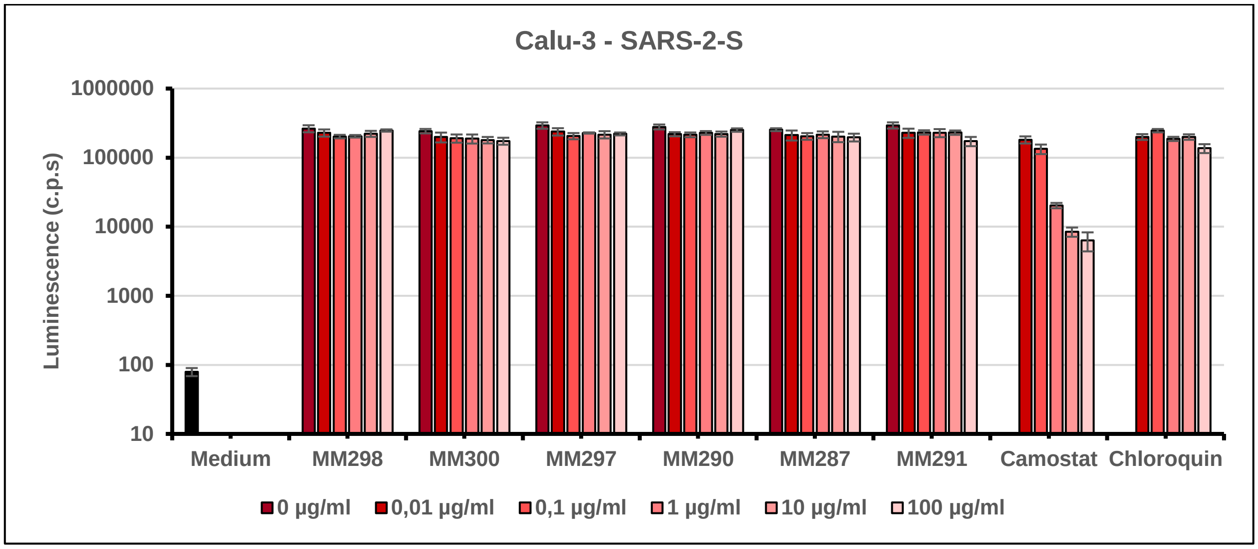

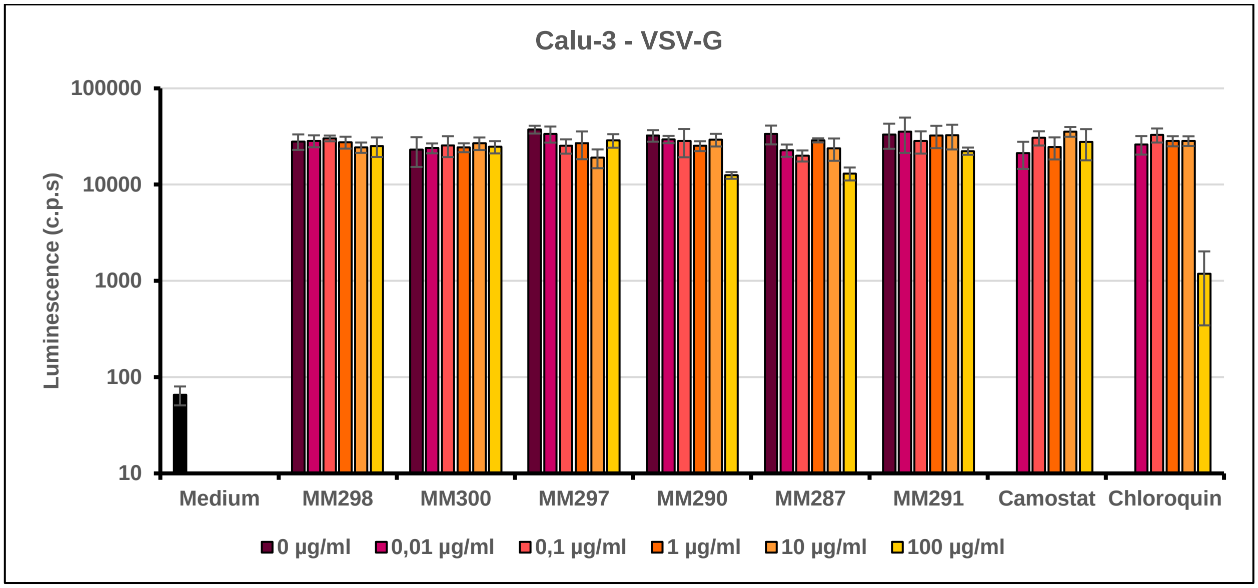

Figure S6. Calu-3 cells seeded in 96-well plates were incubated with the indicated compounds at the indicated final concentrations and inoculated with VSV pseudoparticles bearing SARS-CoV-2 S protein (top panel) or VSV-G (bottom panel) or control inoculated with medium. Luciferase activity in cell lysates was determined at 16-18 h post infection. The results of a single experiment carried out with technical quadruplicates is shown. Error bars indicate SD.

Vero76 cell infection with pseudotypes

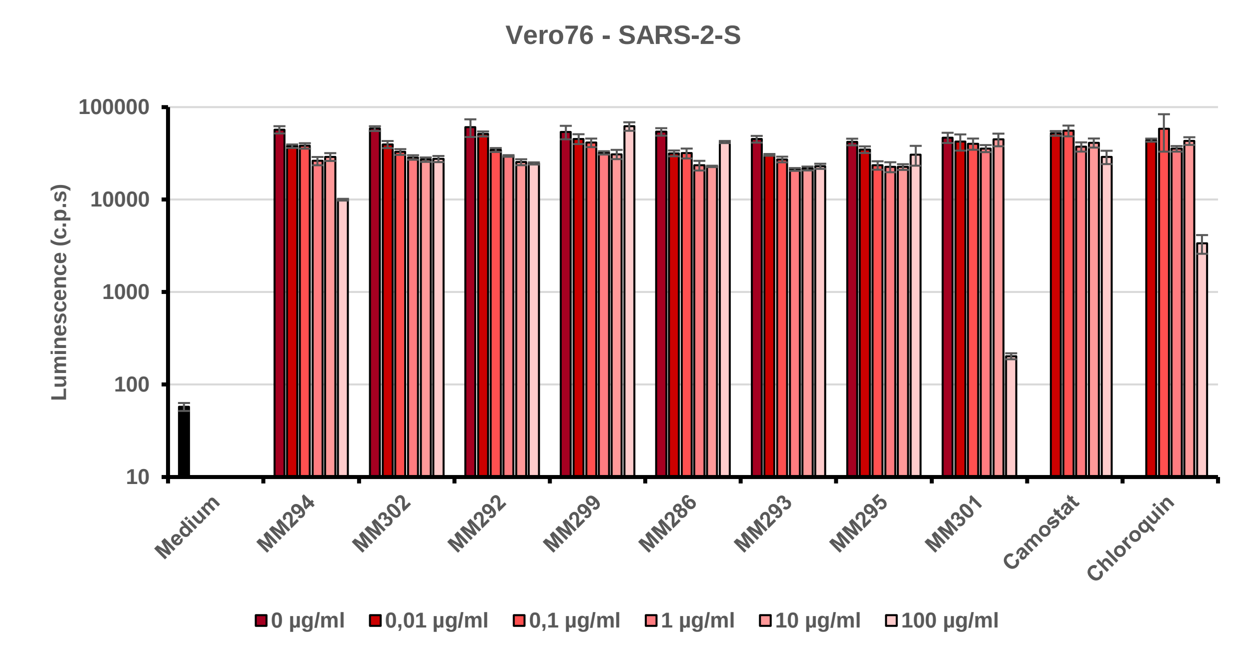

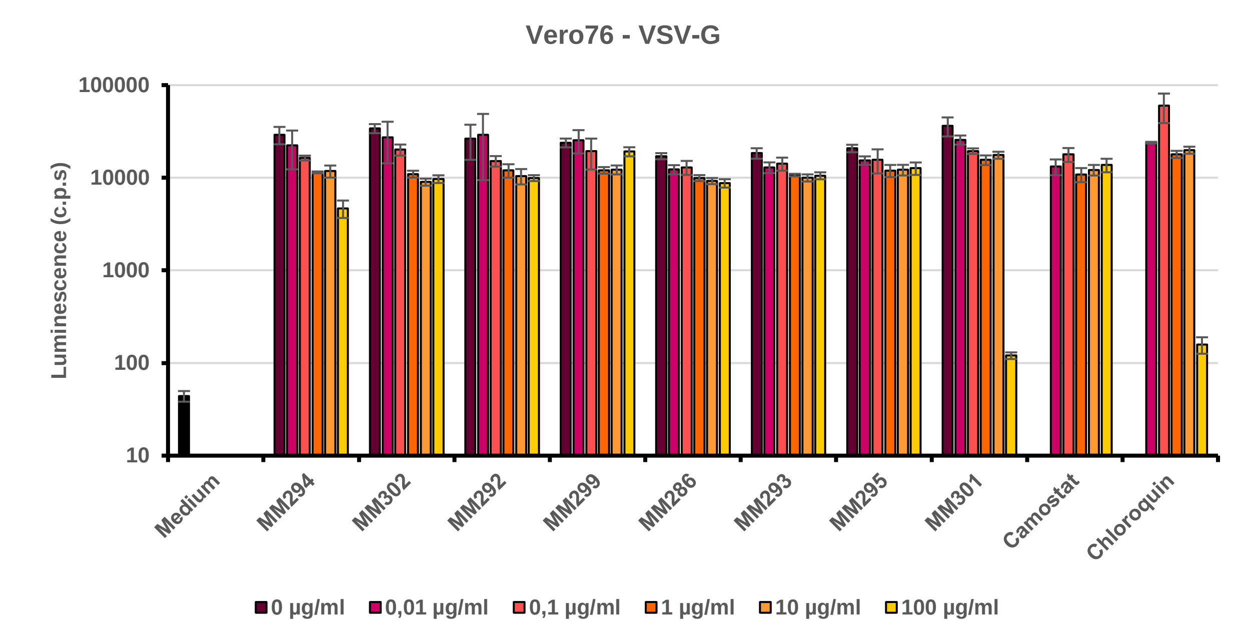

Figure S7. Vero76 cells seeded in 96-well plates were incubated with the indicated compounds at the indicated final concentrations and inoculated with VSV pseudoparticles bearing SARS-CoV-2 S protein (top panel) or VSV-G (bottom panel) or control inoculated with medium. Luciferase activity in cell lysates was determined at 16-18 h post infection. The results of a single experiment carried out with technical quadruplicates is shown. Error bars indicate SD.

Calu-3 cell infection with pseudotypes

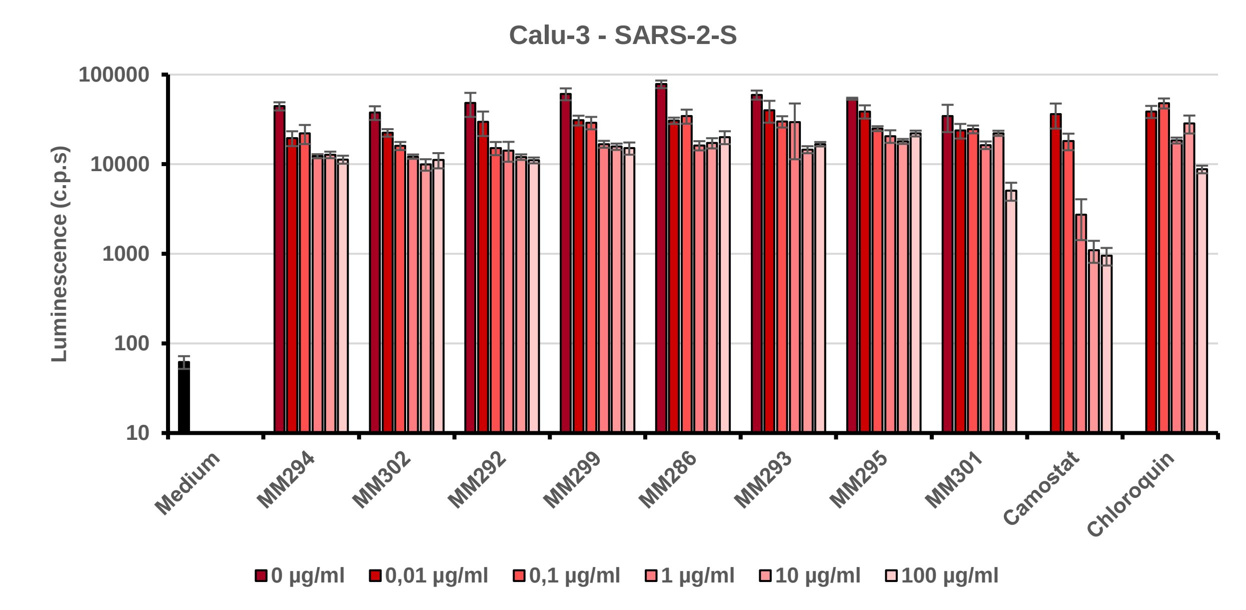

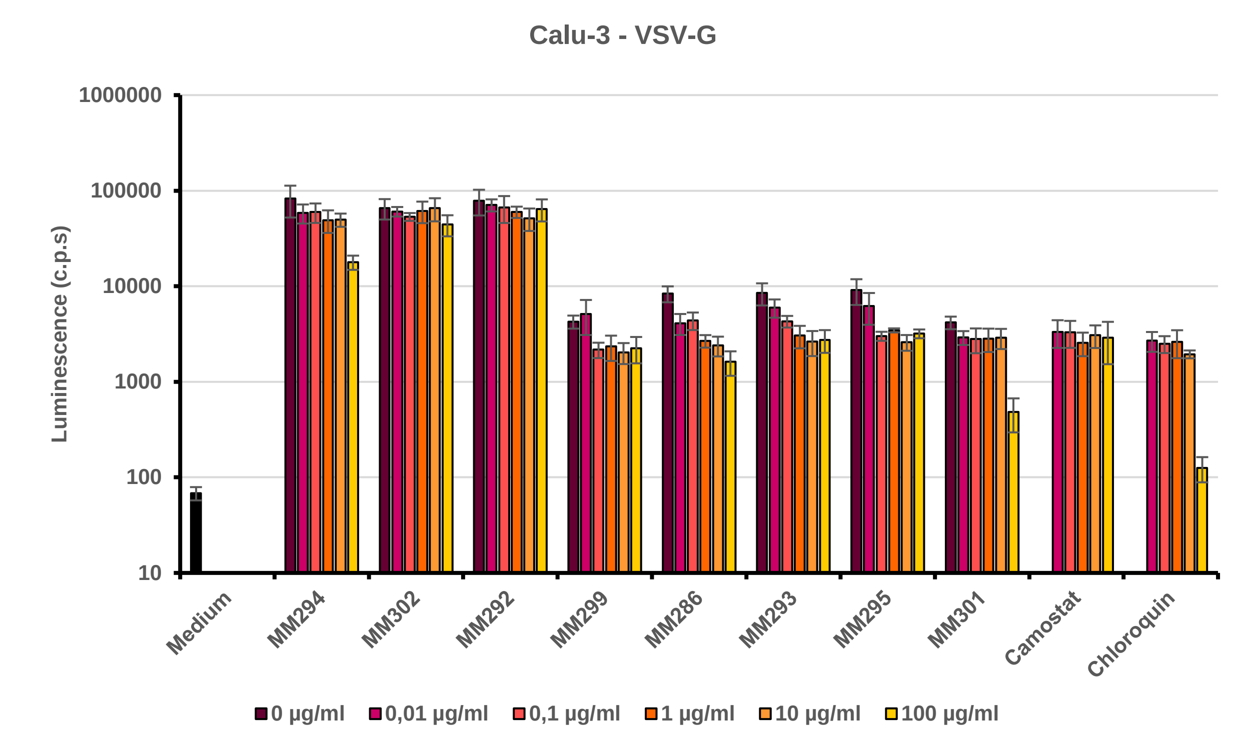

Figure S8. Calu-3 cells seeded in 96-well plates were incubated with the indicated compounds at the indicated final concentrations and inoculated with VSV pseudoparticles bearing SARS-CoV-2 S protein (top panel) or VSV-G (bottom panel) or control inoculated with medium. Luciferase activity in cell lysates was determined at 16-18 h post infection. The results of a single experiment carried out with technical quadruplicates is shown. Error bars indicate SD.

Vero76 cell infection with pseudotypes

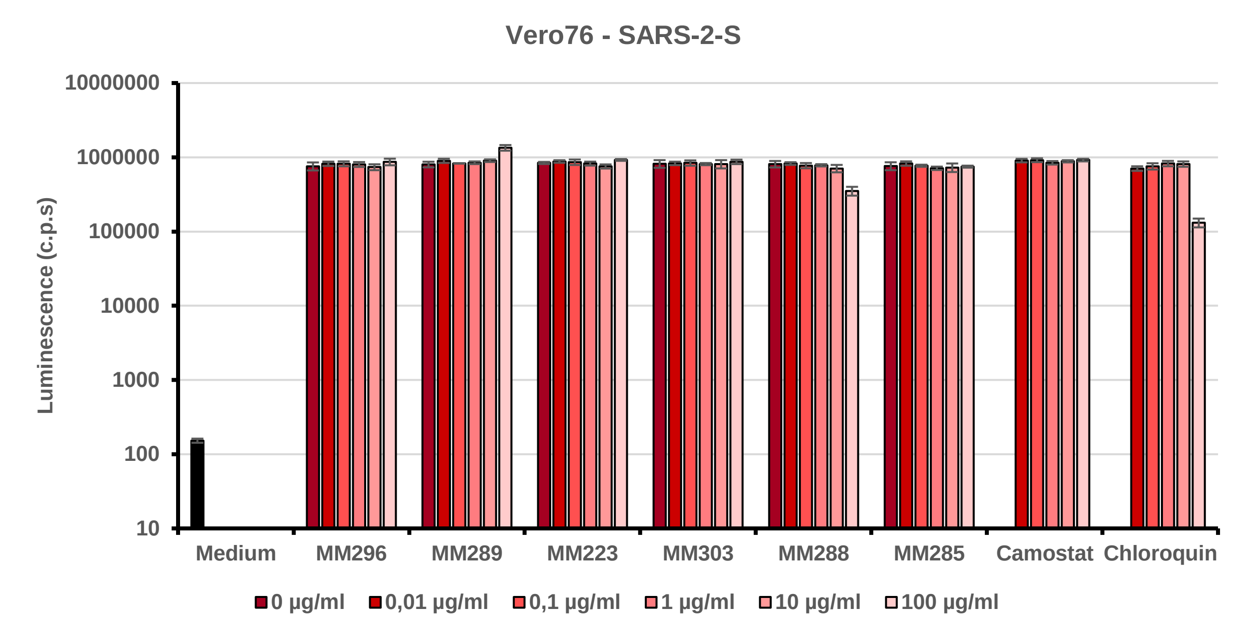

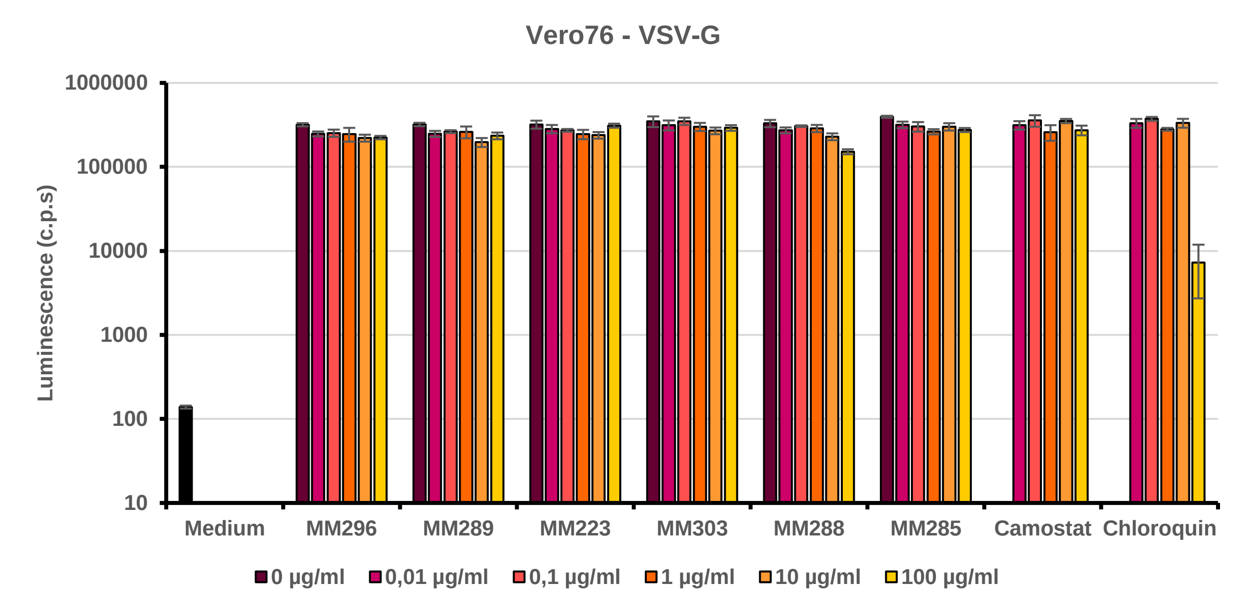

Figure S9. Vero76 cells seeded in 96-well plates were incubated with the indicated compounds at the indicated final concentrations and inoculated with VSV pseudoparticles bearing SARS-CoV-2 S protein (top panel) or VSV-G (bottom panel) or control inoculated with medium. Luciferase activity in cell lysates was determined at 16-18 h post infection. The results of a single experiment carried out with technical quadruplicates is shown. Error bars indicate SD.

Calu-3 cell infection with pseudotypes

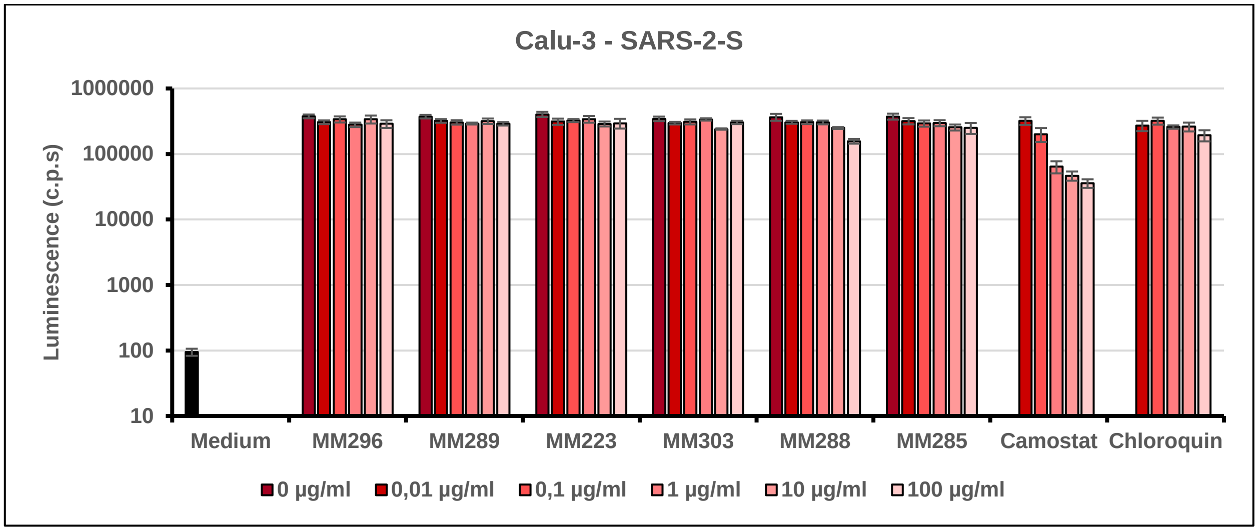

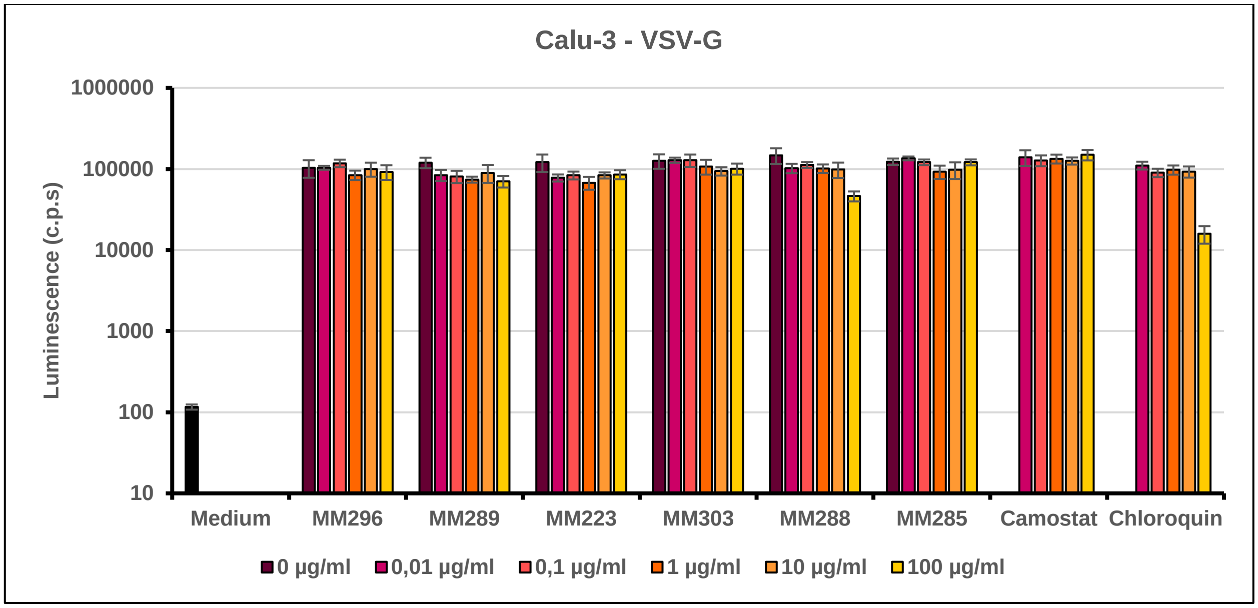

Figure S10. Calu-3 cells seeded in 96-well plates were incubated with the indicated compounds at the indicated final concentrations and inoculated with VSV pseudoparticles bearing SARS-CoV-2 S protein (top panel) or VSV-G (bottom panel) or control inoculated with medium. Luciferase activity in cell lysates was determined at 16-18 h post infection. The results of a single experiment carried out with technical quadruplicates is shown. Error bars indicate SD.

Vero76 cell infection with pseudotypes (ignore results for MM541 and MM551)

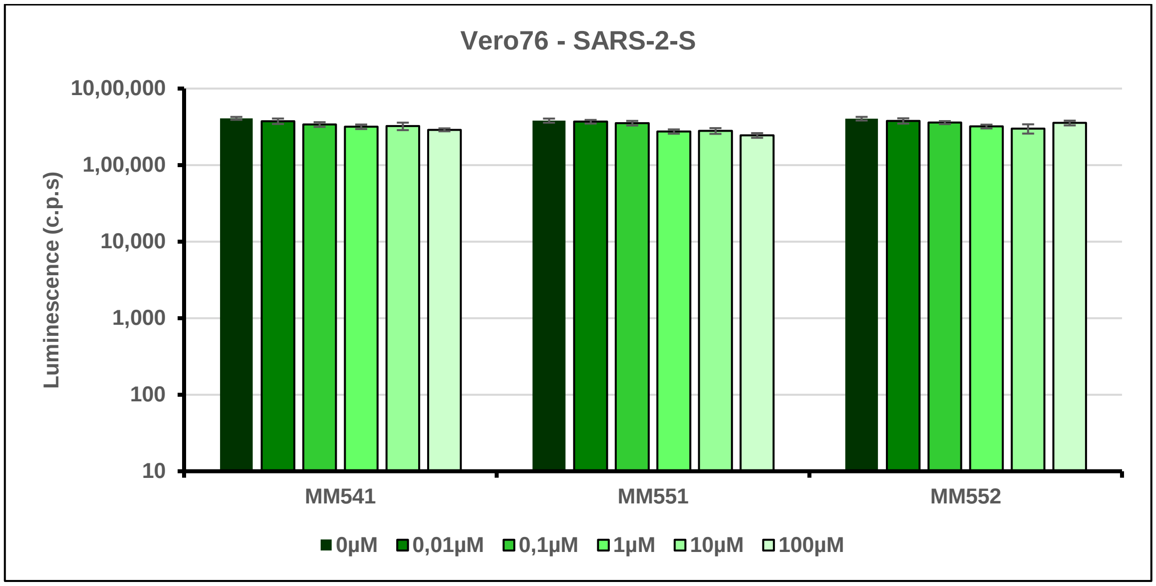

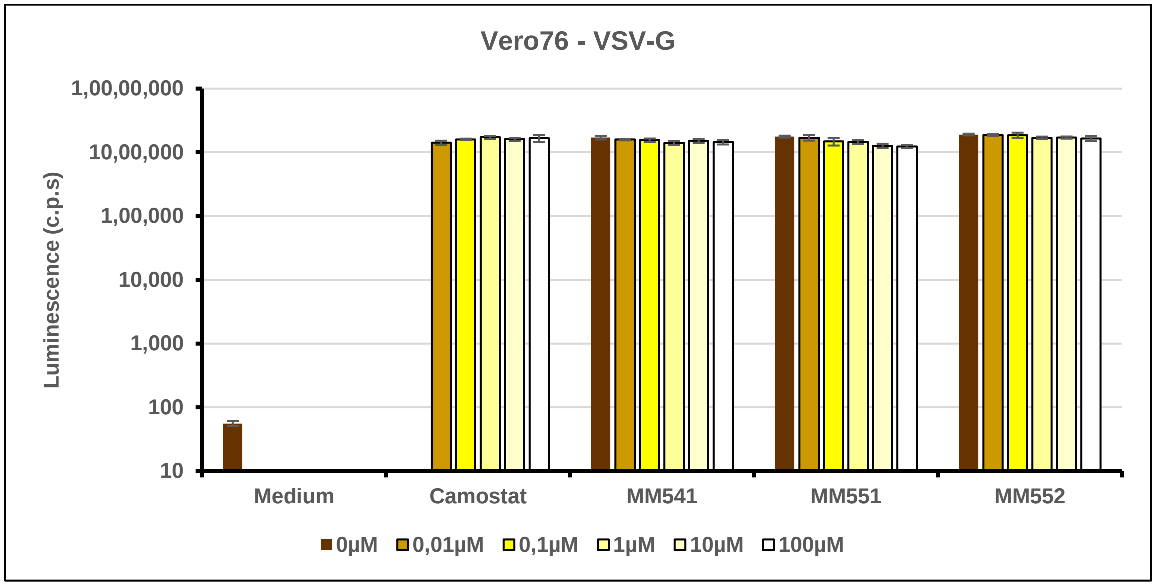

Figure S11. Vero76 cells seeded in 96-well plates were incubated with the indicated compounds at the indicated final concentrations and inoculated with VSV pseudoparticles bearing SARS-CoV-2 S protein (top panel) or VSV-G (bottom panel) or control inoculated with medium. Luciferase activity in cell lysates was determined at 16-18 h post infection. The results of a single experiment carried out with technical quadruplicates is shown. Error bars indicate SD.

Calu-3 cell infection with pseudotypes (ignore results for MM541 and MM551)

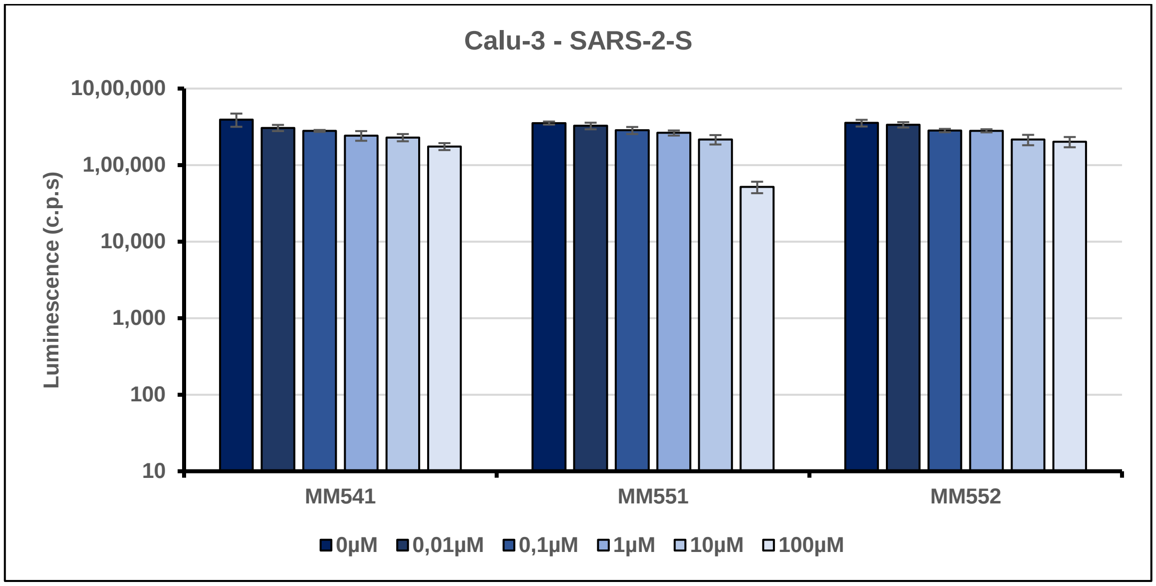

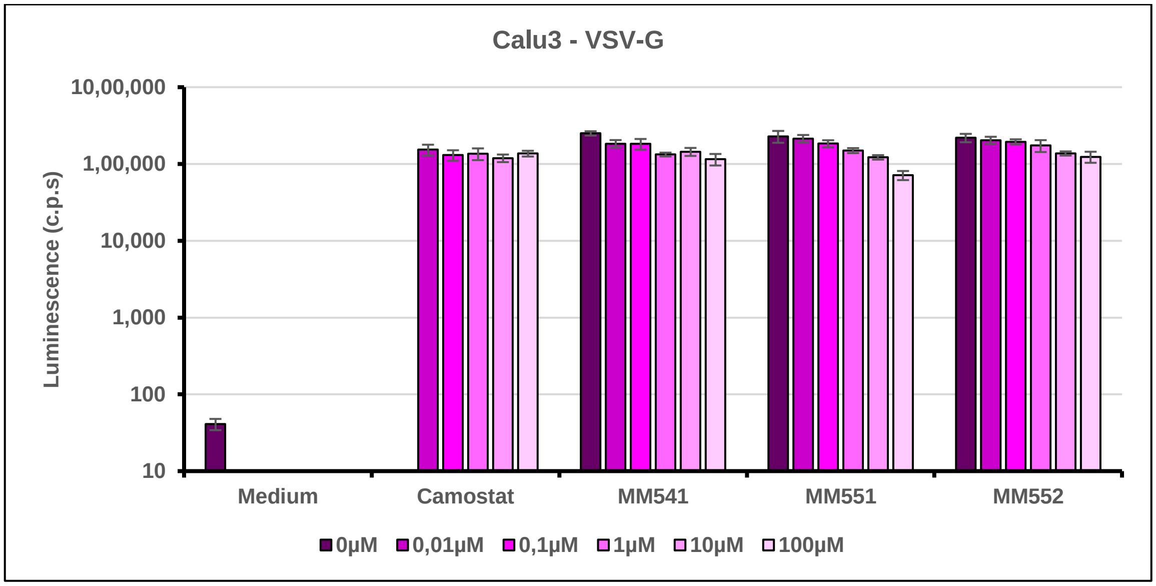

Figure S12. Calu-3 cells seeded in 96-well plates were incubated with the indicated compounds at the indicated final concentrations and inoculated with VSV pseudoparticles bearing SARS-CoV-2 S protein (top panel) or VSV-G (bottom panel) or control inoculated with medium. Luciferase activity in cell lysates was determined at 16-18 h post infection. The results of a single experiment carried out with technical quadruplicates is shown. Error bars indicate SD.

Vero76 cell infection with pseudotypes (ignore results for MM539, MM357, MM542, MM543 & MM544)

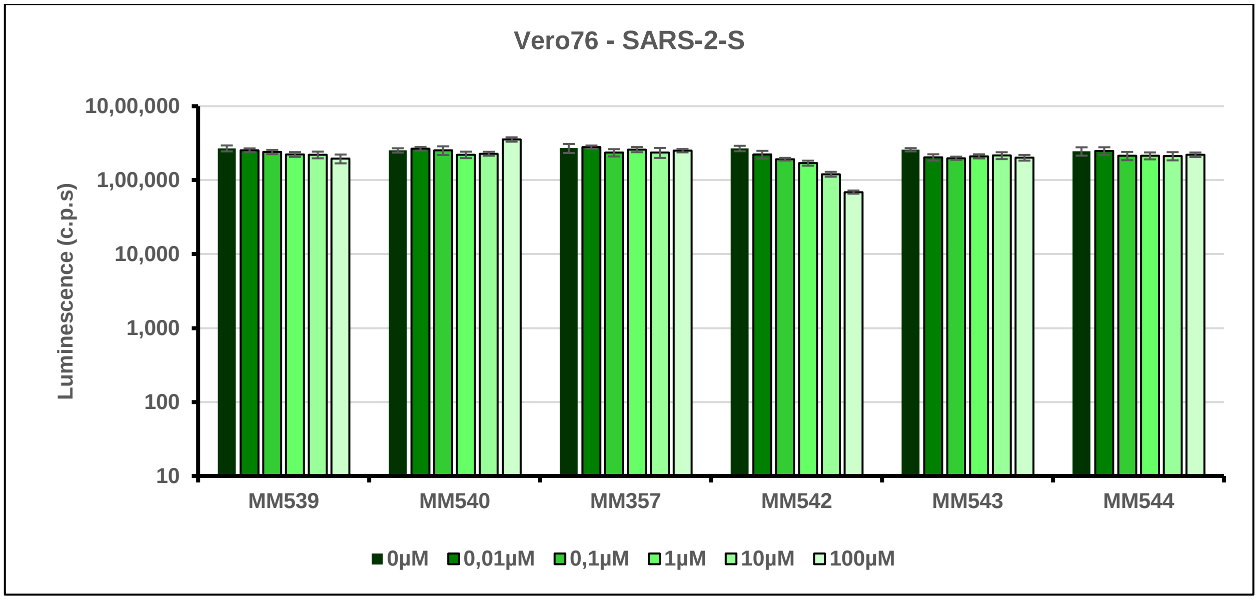

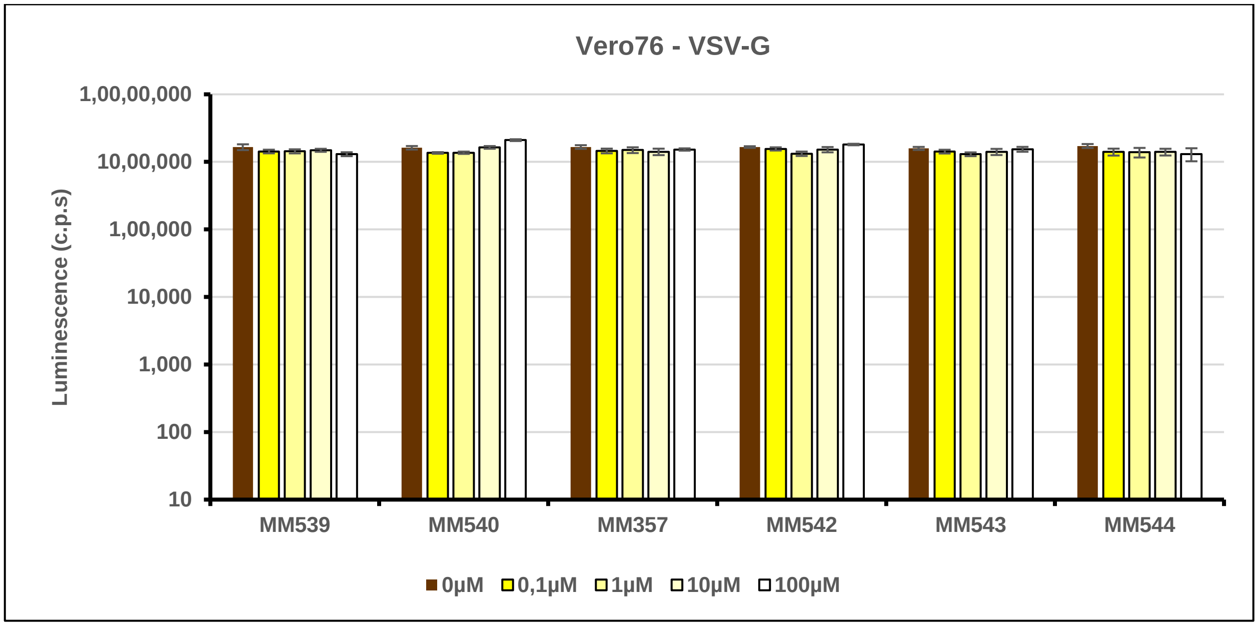

Figure S13. Vero76 cells seeded in 96-well plates were incubated with the indicated compounds at the indicated final concentrations and inoculated with VSV pseudoparticles bearing SARS-CoV-2 S protein (top panel) or VSV-G (bottom panel). Luciferase activity in cell lysates was determined at 16-18 h post infection. The results of a single experiment carried out with technical quadruplicates is shown. Error bars indicate SD.

Calu-3 cell infection with pseudotypes (ignore results for MM539, MM357, MM542, MM543 & MM544)

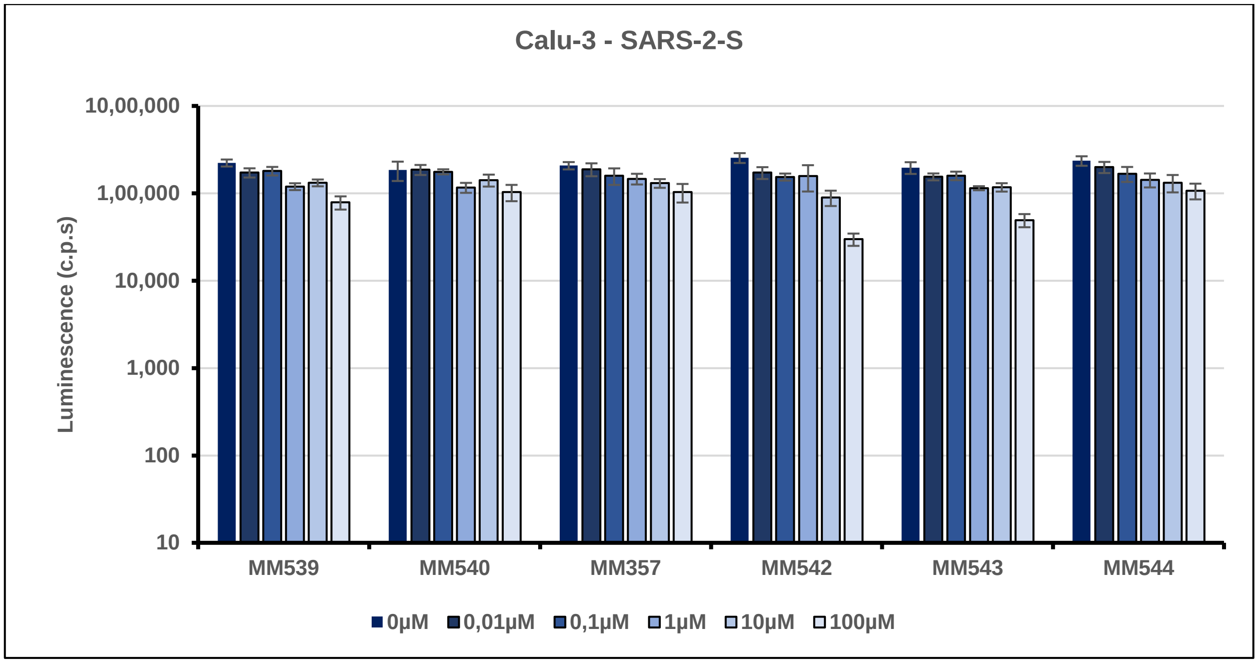

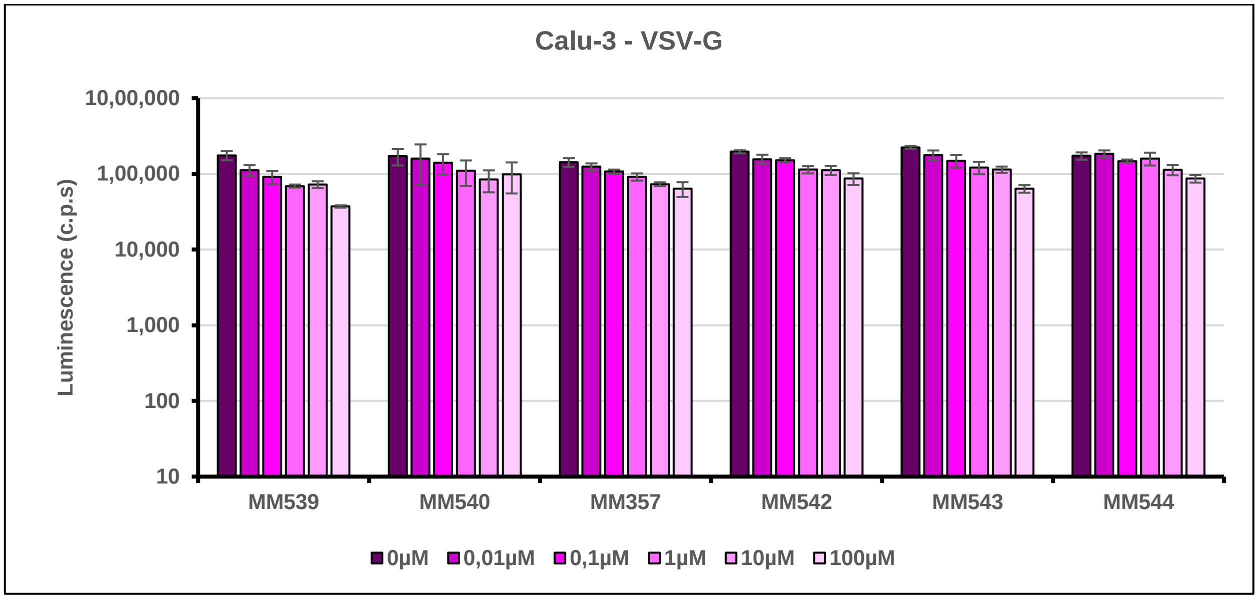

Figure S14. Calu-3 cells seeded in 96-well plates were incubated with the indicated compounds at the indicated final concentrations and inoculated with VSV pseudoparticles bearing SARS-CoV-2 S protein (top panel) or VSV-G (bottom panel). Luciferase activity in cell lysates was determined at 16-18 h post infection. The results of a single experiment carried out with technical quadruplicates is shown. Error bars indicate SD.

Vero76 cell infection with pseudotypes (ignore results for MM534, MM537, & MM538)

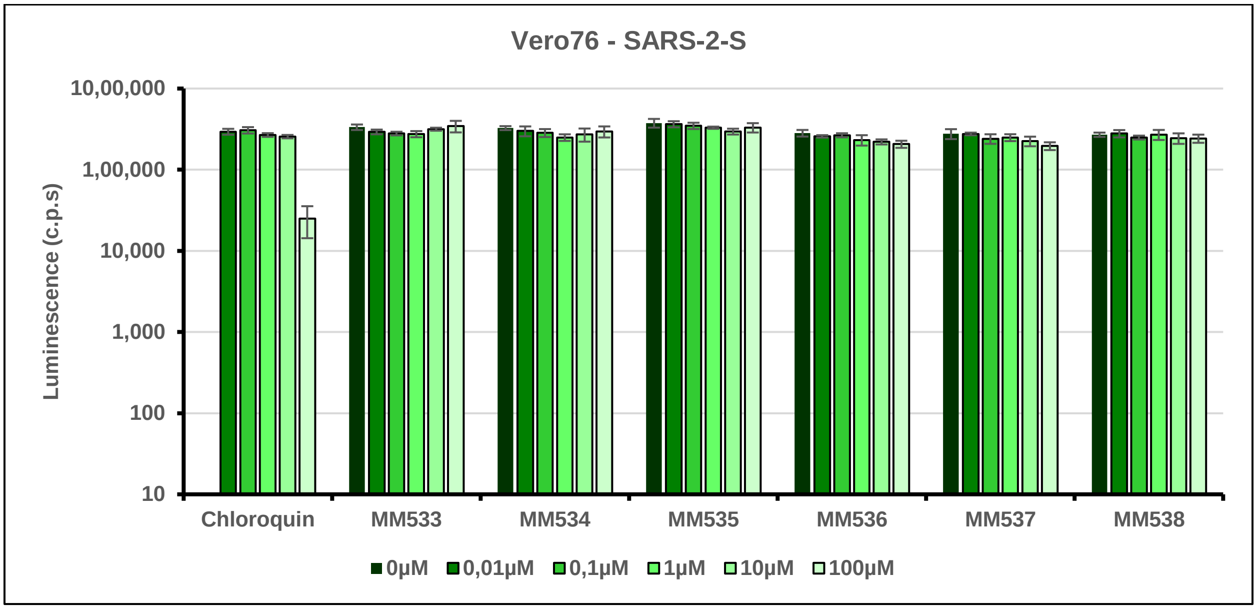

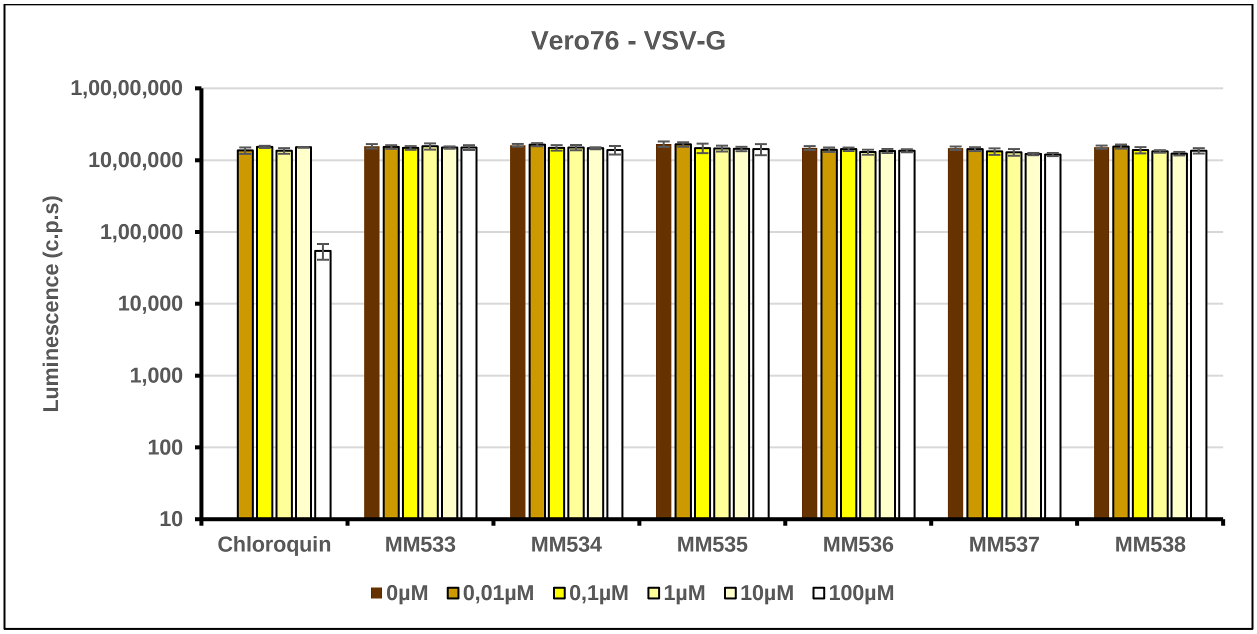

Figure S15. Vero76 cells seeded in 96-well plates were incubated with the indicated compounds at the indicated final concentrations and inoculated with VSV pseudoparticles bearing SARS-CoV-2 S protein (top panel) or VSV-G (bottom panel). Luciferase activity in cell lysates was determined at 16-18 h post infection. The results of a single experiment carried out with technical quadruplicates is shown. Error bars indicate SD.

Calu-3 cell infection with pseudotypes (ignore results for MM534, MM537, & MM538)

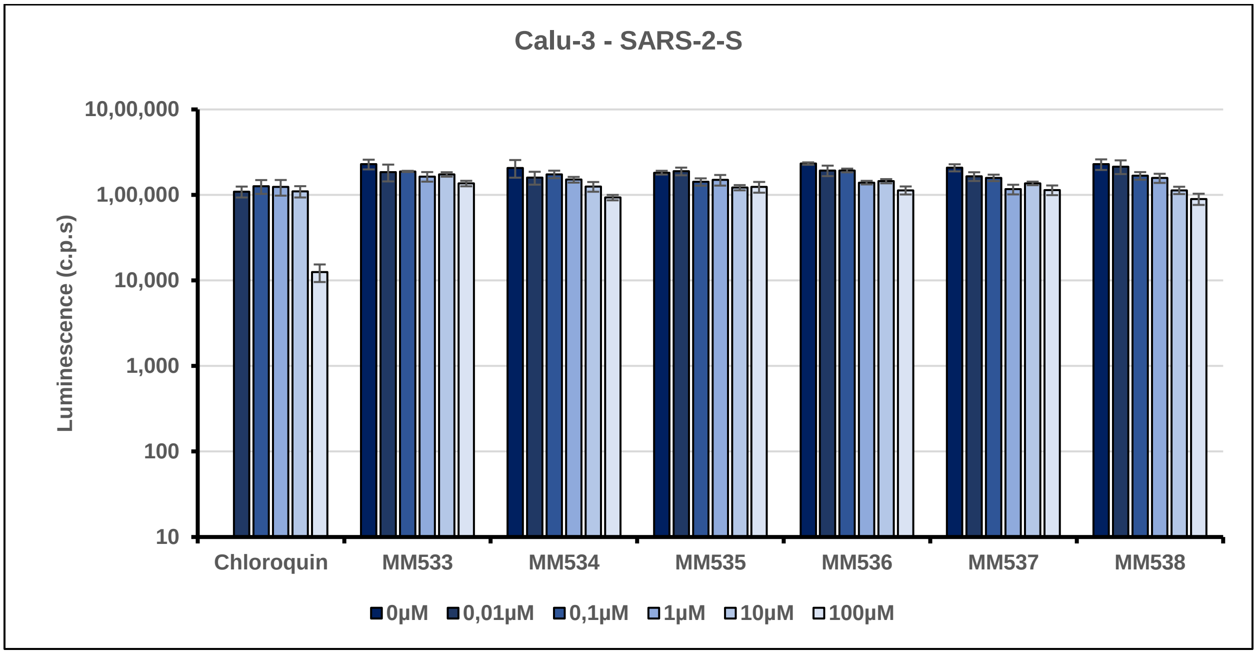

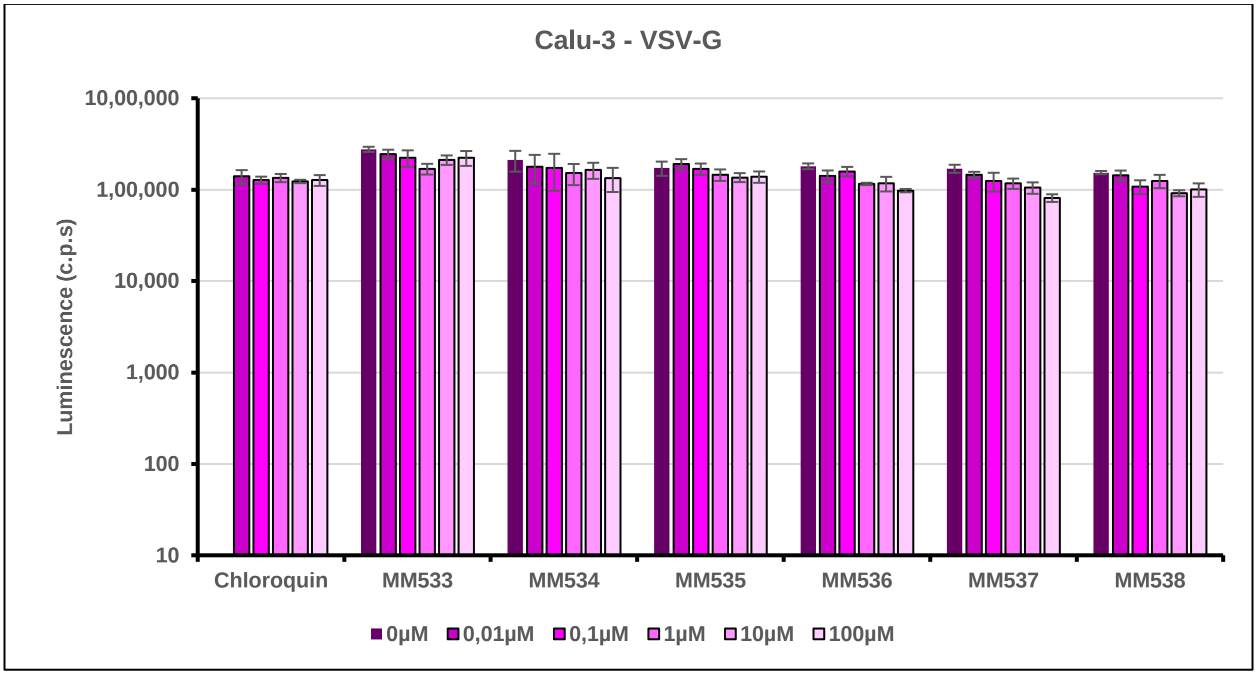

Figure S16. Calu-3 cells seeded in 96-well plates were incubated with the indicated compounds at the indicated final concentrations and inoculated with VSV pseudoparticles bearing SARS-CoV-2 S protein (top panel) or VSV-G (bottom panel). Luciferase activity in cell lysates was determined at 16-18 h post infection. The results of a single experiment carried out with technical quadruplicates is shown. Error bars indicate SD.

6. ^{1}H, and ^{13}C NMR spectra

Compound **27**

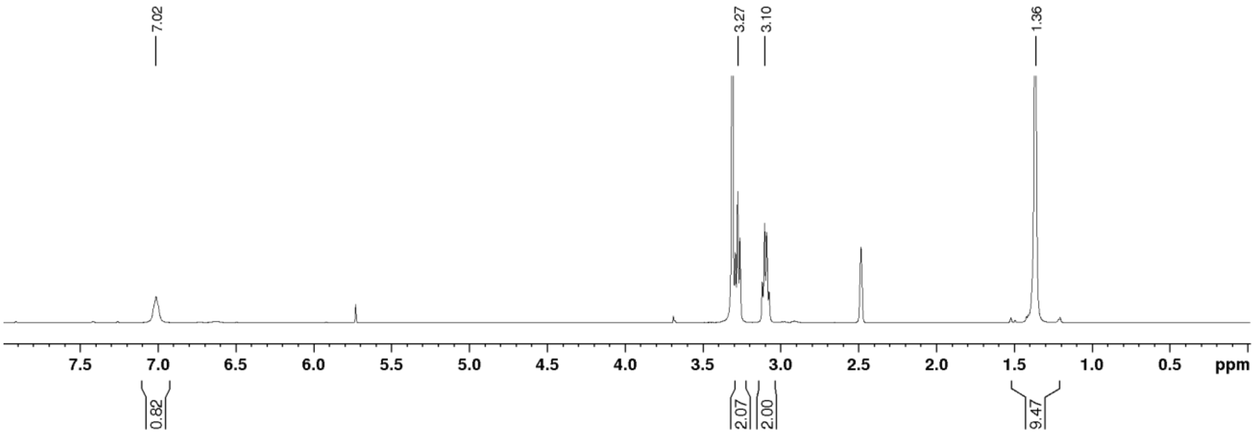

Compound **Boc26A**

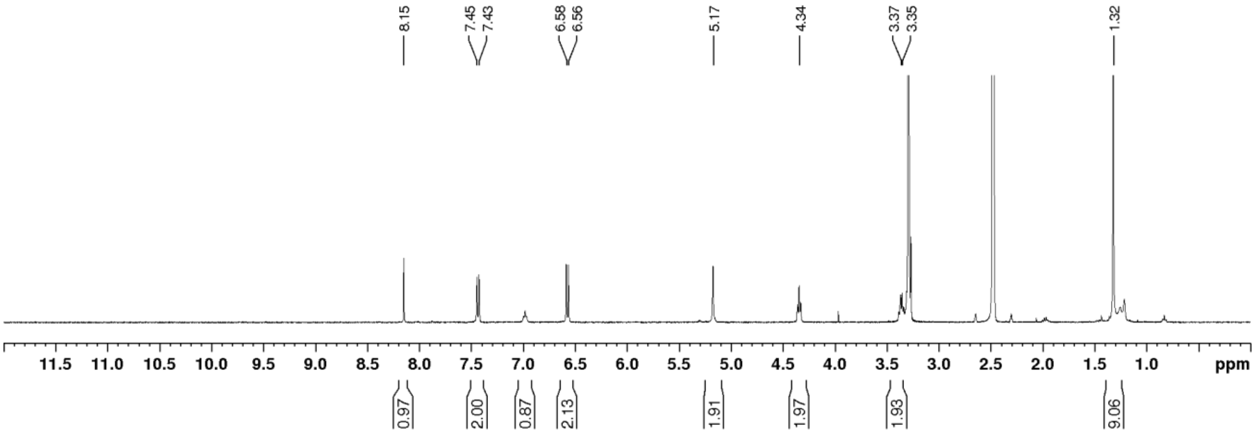

Compound **1**

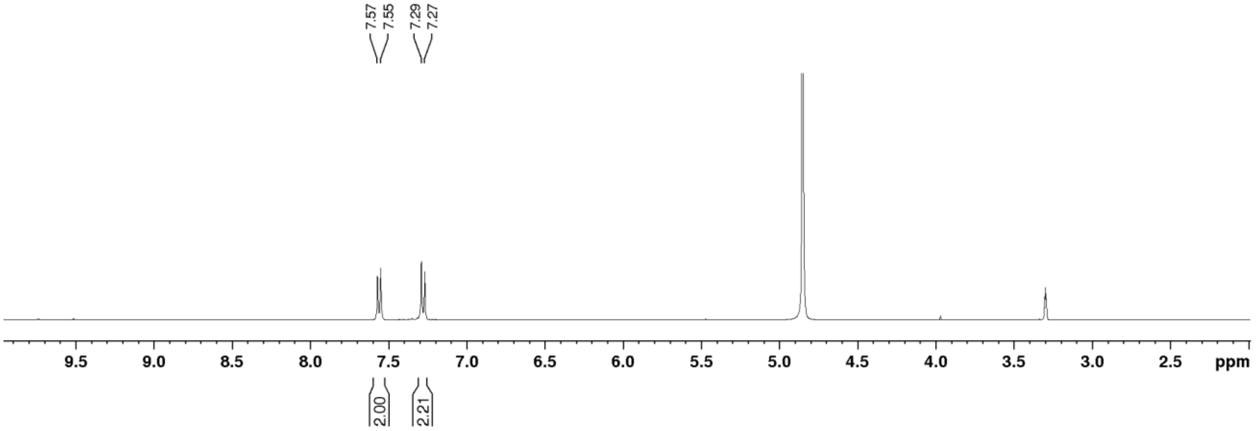

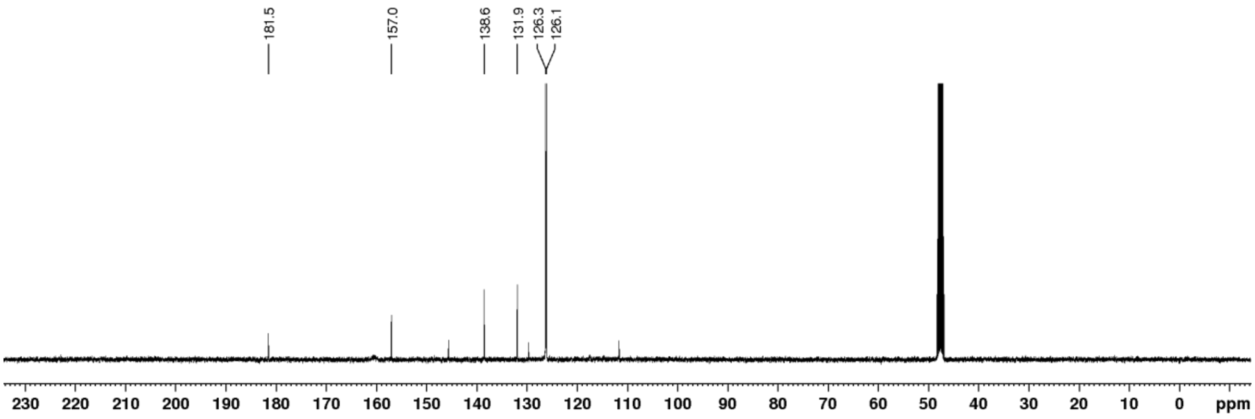
181.5
157.0
138.6
131.9
126.3
126.1
230 220 210 200 190 180 170 160 150 140 130 120 110 100 90 80 70 60 50 40 30 20 10 0 ppm

Compound **2**

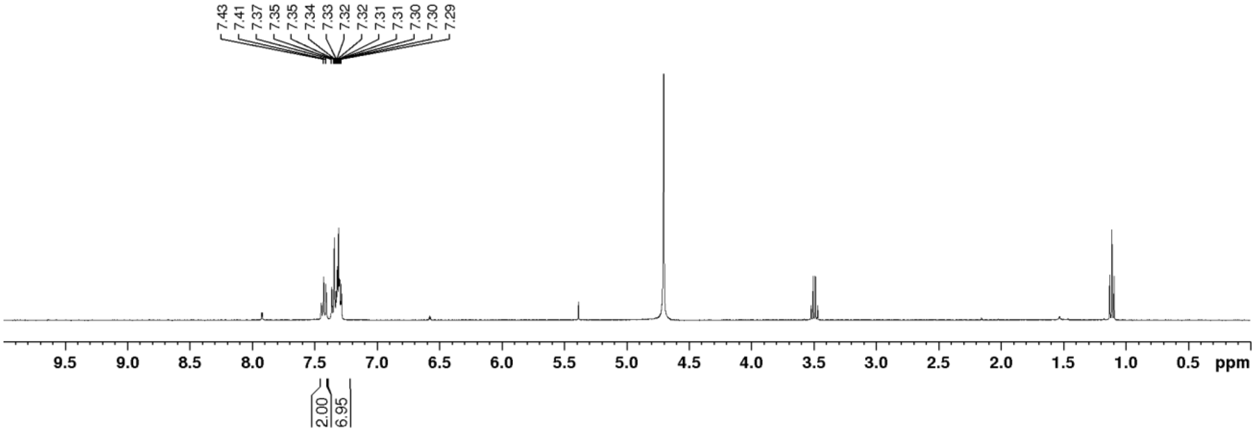
7.43
7.41
7.37
7.35
7.35
7.34
7.33
7.32
7.32
7.31
7.31
7.30
7.30
7.29
9.5 9.0 8.5 8.0 7.5 7.0 6.5 6.0 5.5 5.0 4.5 4.0 3.5 3.0 2.5 2.0 1.5 1.0 0.5 ppm
2.00
6.95

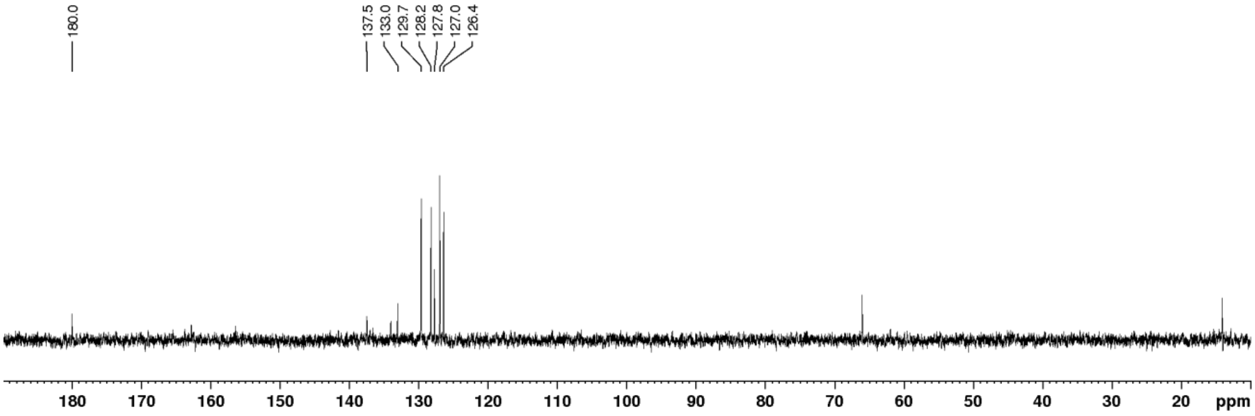
180.0
137.5
133.0
129.7
128.2
127.8
127.0
126.4
180 170 160 150 140 130 120 110 100 90 80 70 60 50 40 30 20 ppm

Compound **3**

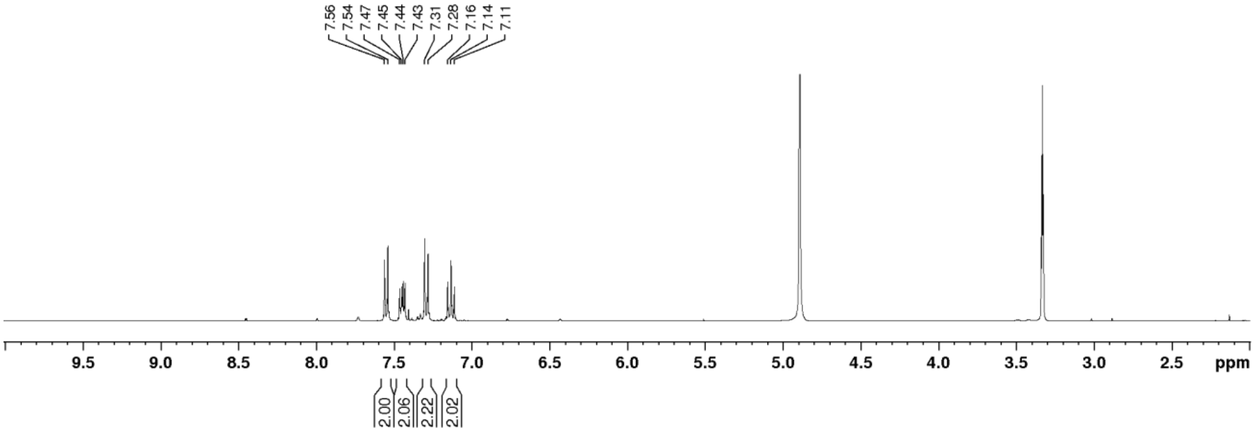

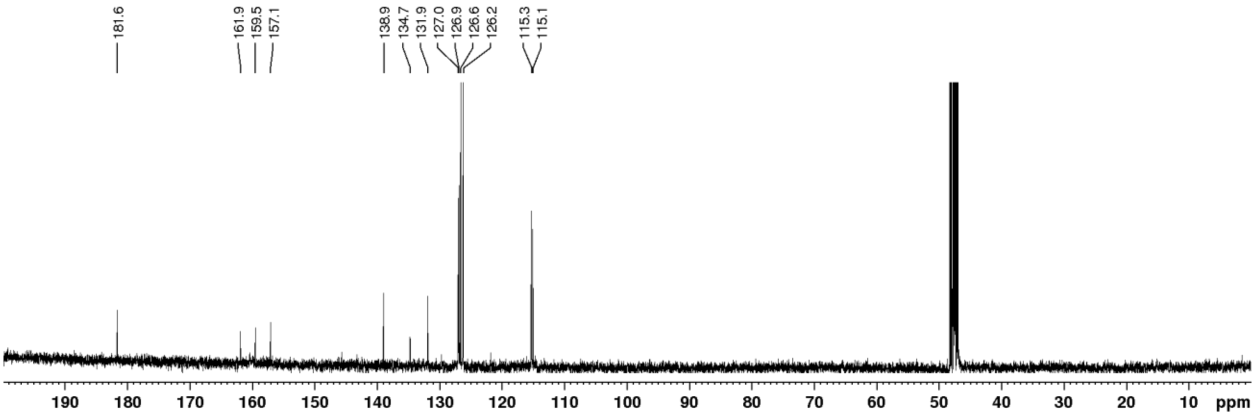

Compound **4**

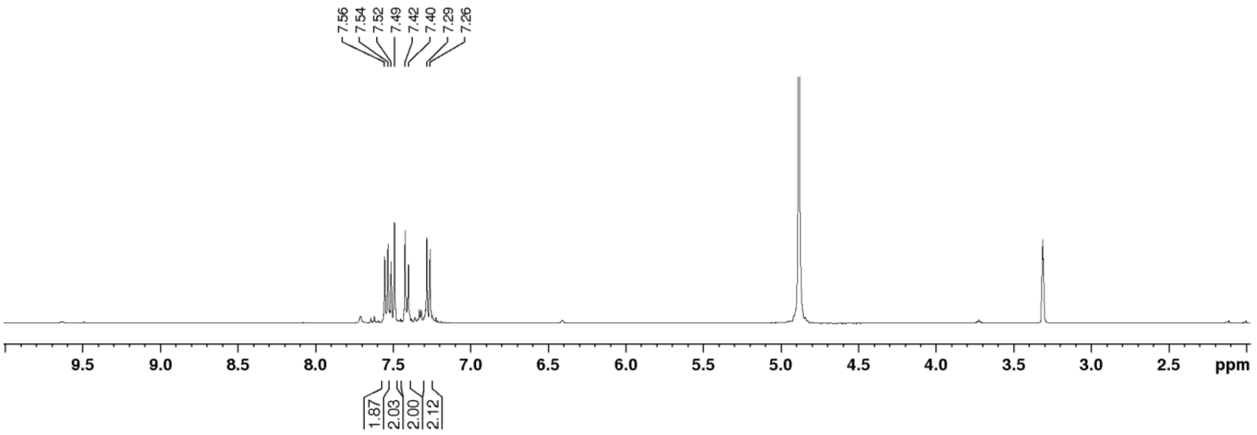

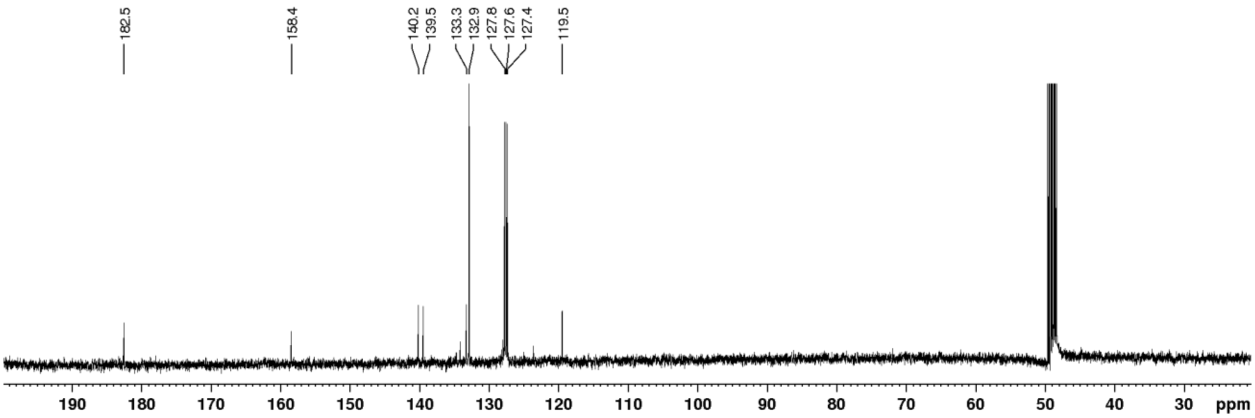
182.5
158.4
140.2
139.5
133.3
132.9
127.8
127.6
127.4
119.5
190 180 170 160 150 140 130 120 110 100 90 80 70 60 50 40 30 ppm

Compound **5**

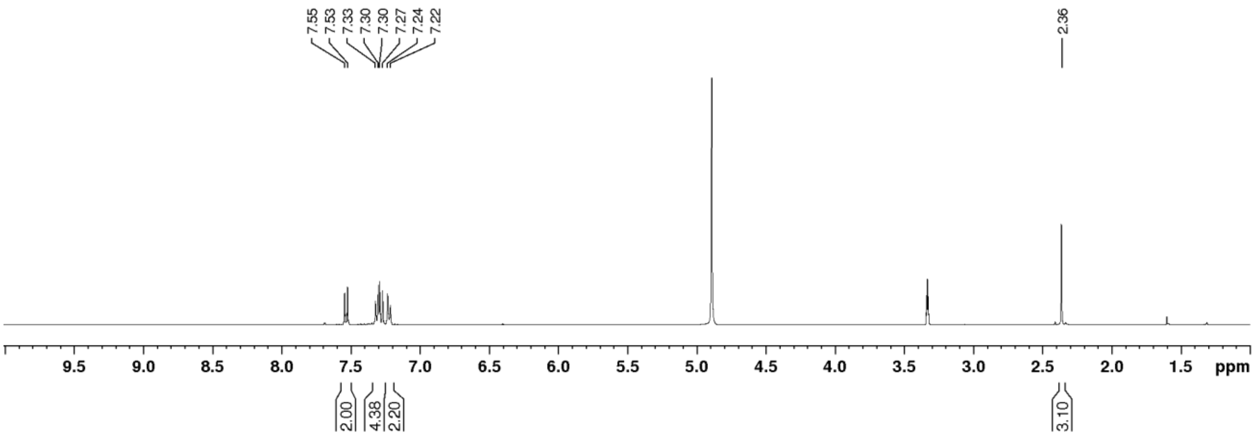
7.55
7.53
7.33
7.30
7.30
7.27
7.24
7.22
2.36
9.5 9.0 8.5 8.0 7.5 7.0 6.5 6.0 5.5 5.0 4.5 4.0 3.5 3.0 2.5 2.0 1.5 ppm
2.00
4.38
2.20
3.10

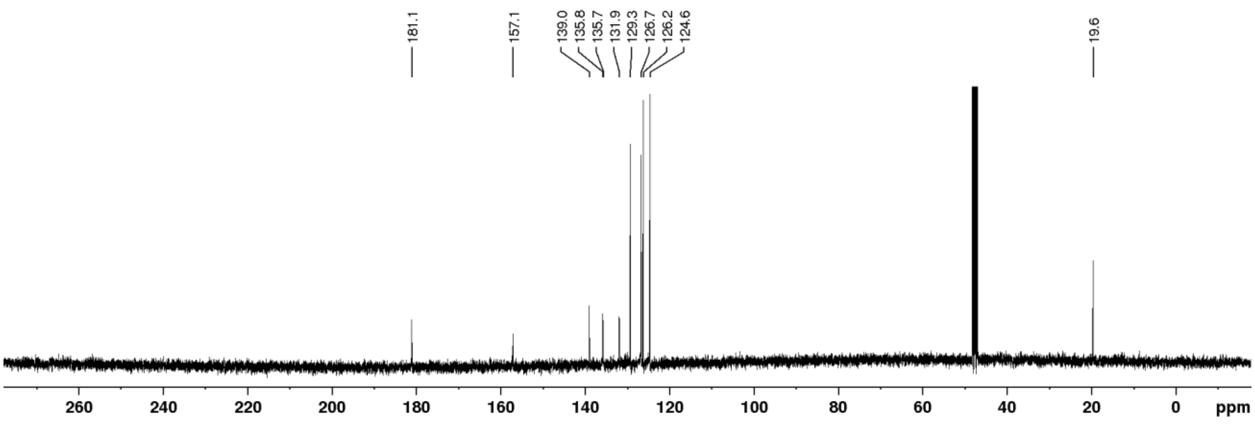
181.1
157.1
139.0
135.8
135.7
131.9
129.3
126.7
126.2
124.6
19.6
260 240 220 200 180 160 140 120 100 80 60 40 20 0 ppm

Compound **6**

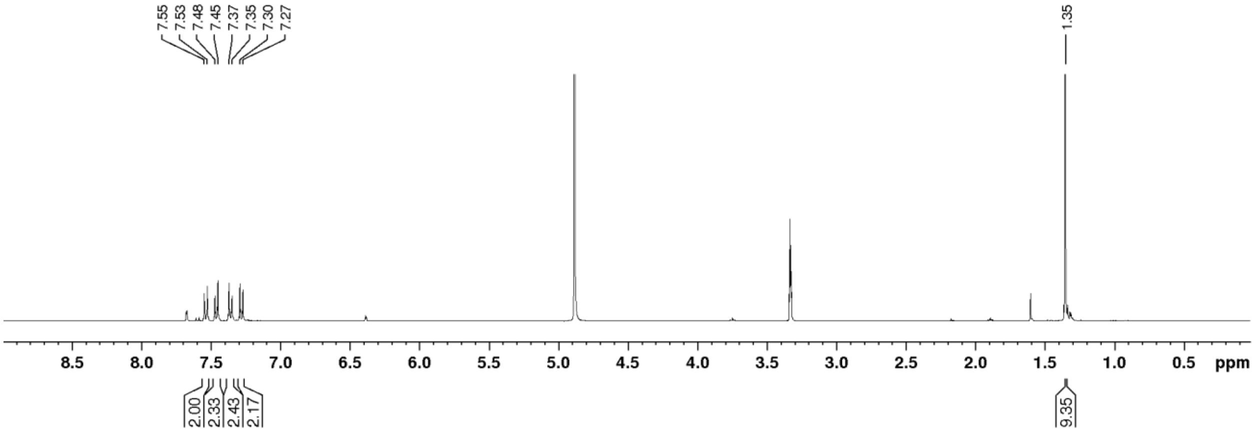

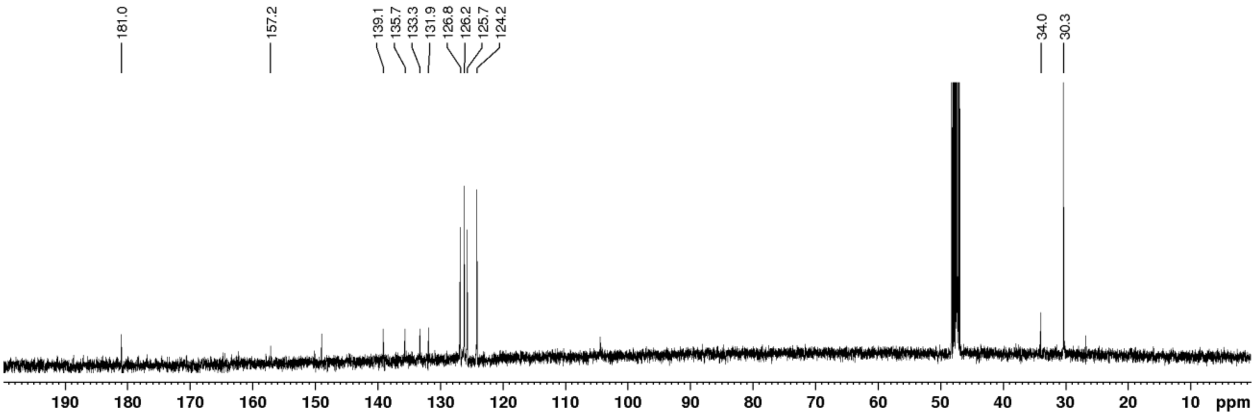

Compound **7**

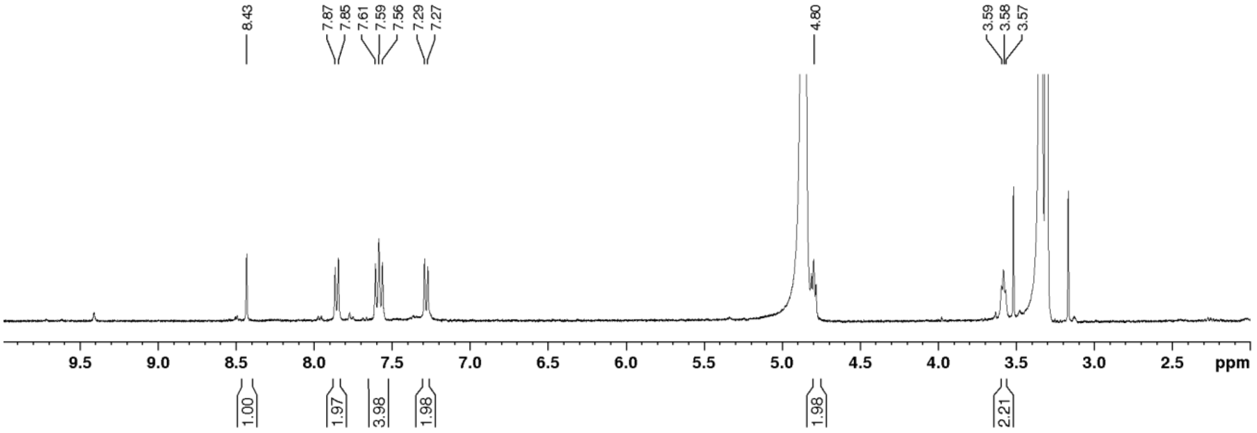

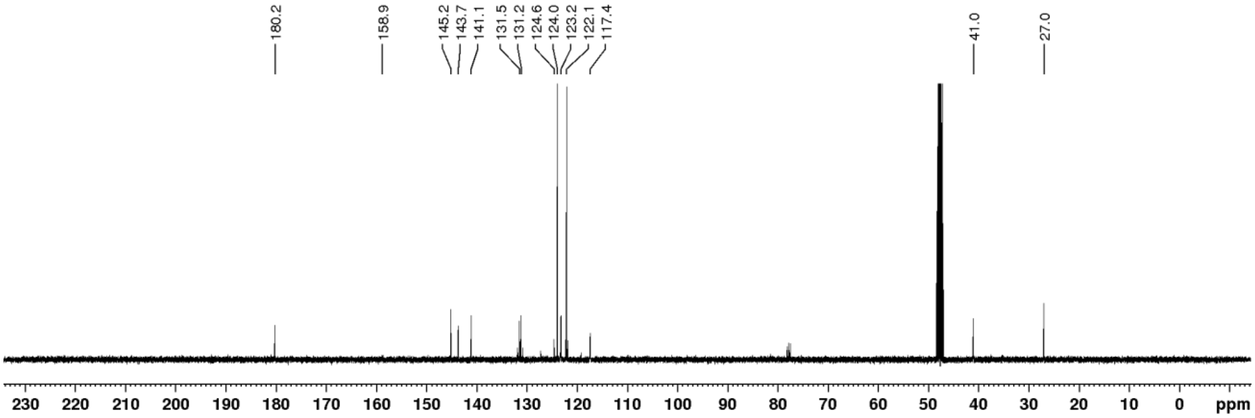
180.2
158.9
145.2
143.7
141.1
131.5
131.2
124.6
124.0
123.2
122.1
117.4
41.0
27.0
230 220 210 200 190 180 170 160 150 140 130 120 110 100 90 80 70 60 50 40 30 20 10 0 ppm

Compound **8**

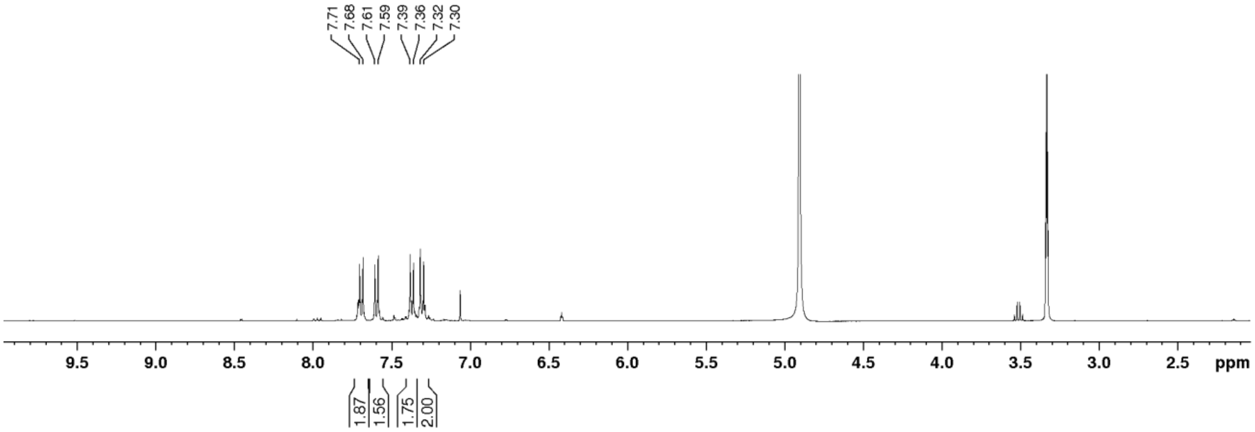
7.71
7.68
7.61
7.59
7.39
7.36
7.32
7.30
9.5 9.0 8.5 8.0 7.5 7.0 6.5 6.0 5.5 5.0 4.5 4.0 3.5 3.0 2.5 ppm
1.87
1.56
1.75
2.00

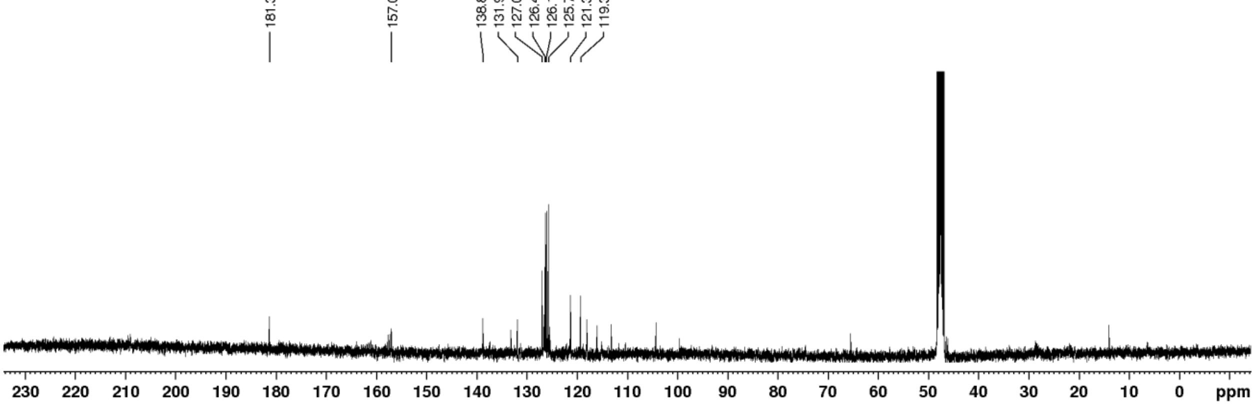
181.3
157.0
138.8
131.9
127.0
126.4
126.1
125.7
121.3
119.3
230 220 210 200 190 180 170 160 150 140 130 120 110 100 90 80 70 60 50 40 30 20 10 0 ppm

Compound **9**

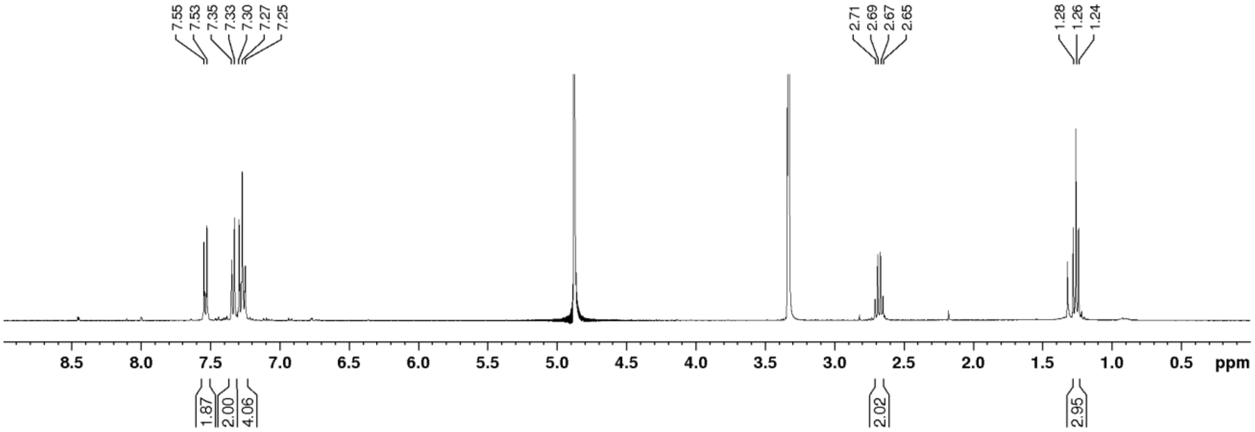

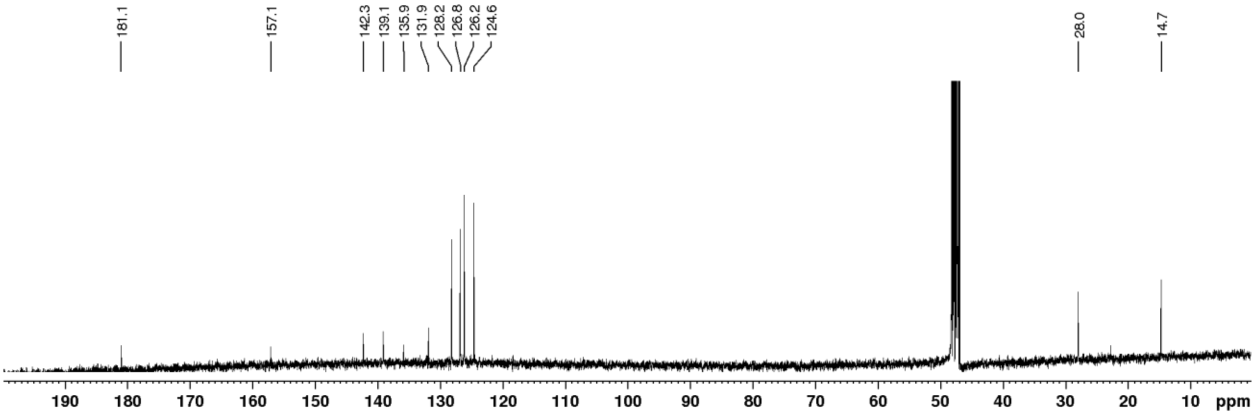

Compound **10**

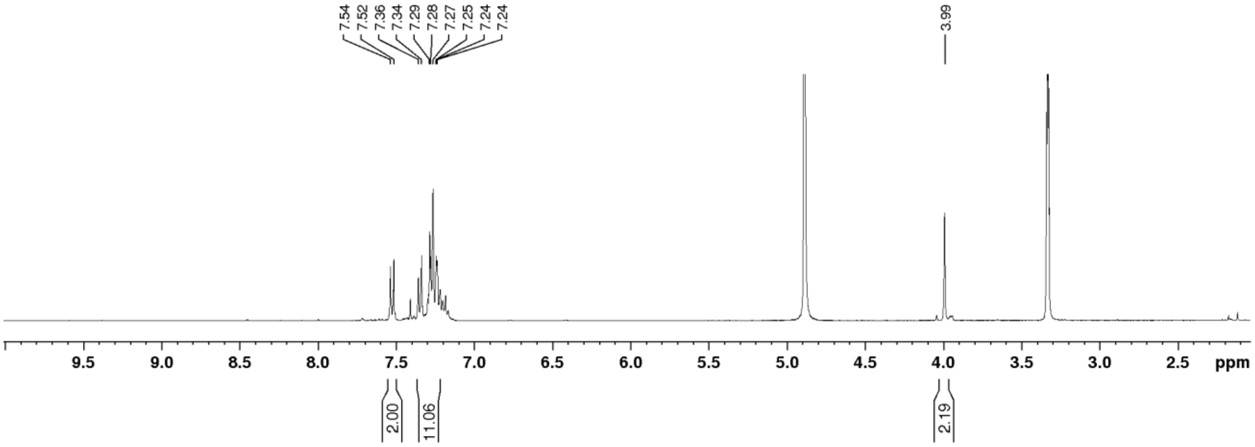

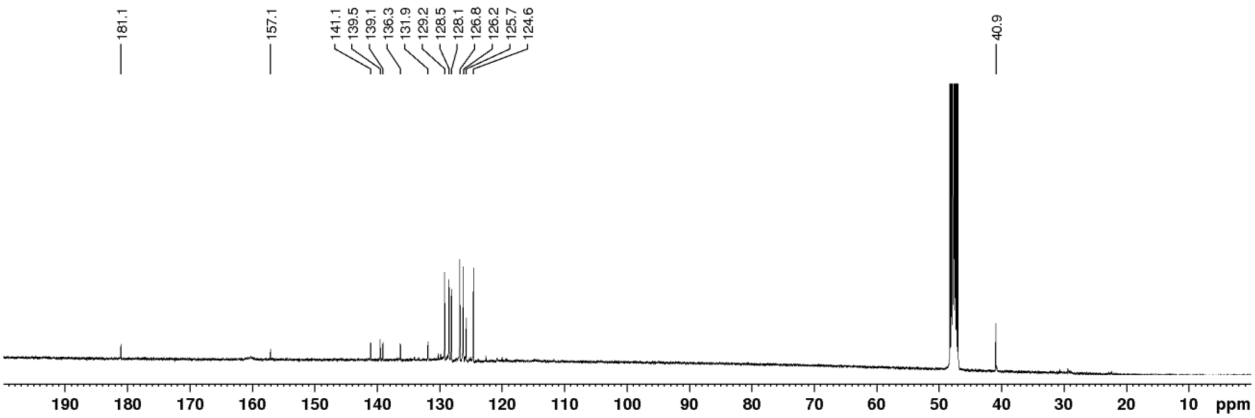
181.1
157.1
141.1
139.5
139.1
136.3
131.9
129.2
128.5
128.1
126.8
126.2
125.7
124.6
40.9
190 180 170 160 150 140 130 120 110 100 90 80 70 60 50 40 30 20 10 ppm

Compound **11**

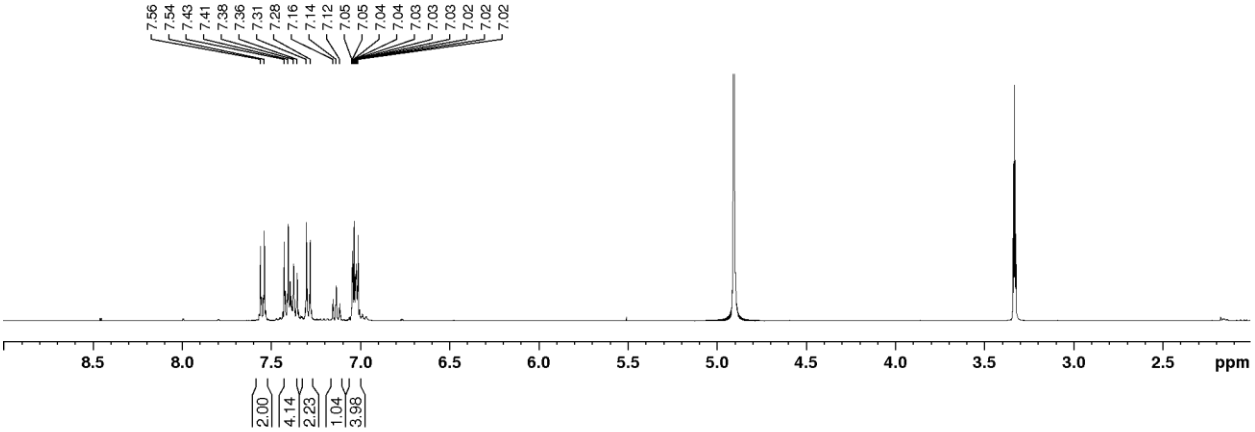
7.56
7.54
7.43
7.41
7.38
7.36
7.31
7.28
7.16
7.14
7.12
7.05
7.05
7.04
7.04
7.03
7.03
7.02
7.02
7.02
8.5 8.0 7.5 7.0 6.5 6.0 5.5 5.0 4.5 4.0 3.5 3.0 2.5 ppm
2.00
4.14
2.23
1.04
3.98

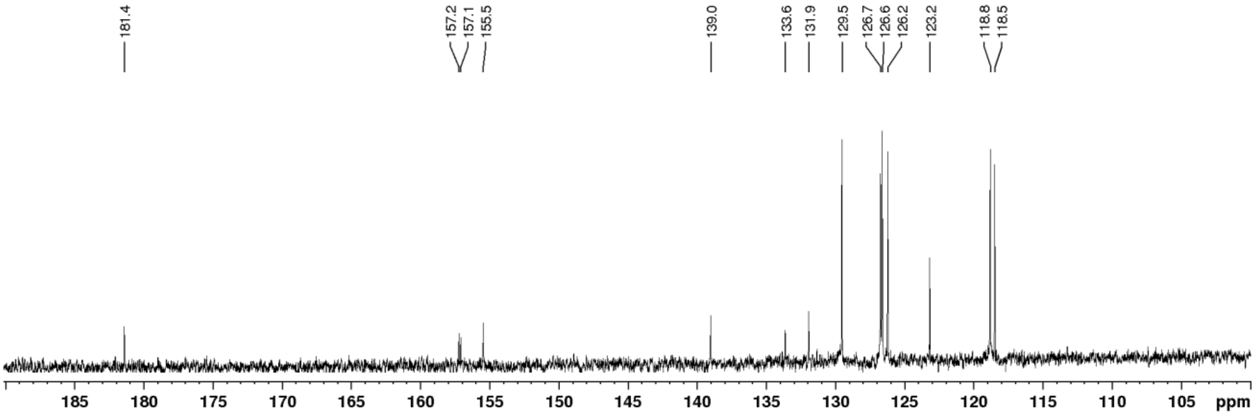
181.4
157.2
157.1
155.5
139.0
133.6
131.9
129.5
126.7
126.6
126.2
123.2
118.8
118.5
185 180 175 170 165 160 155 150 145 140 135 130 125 120 115 110 105 ppm

Compound **12**

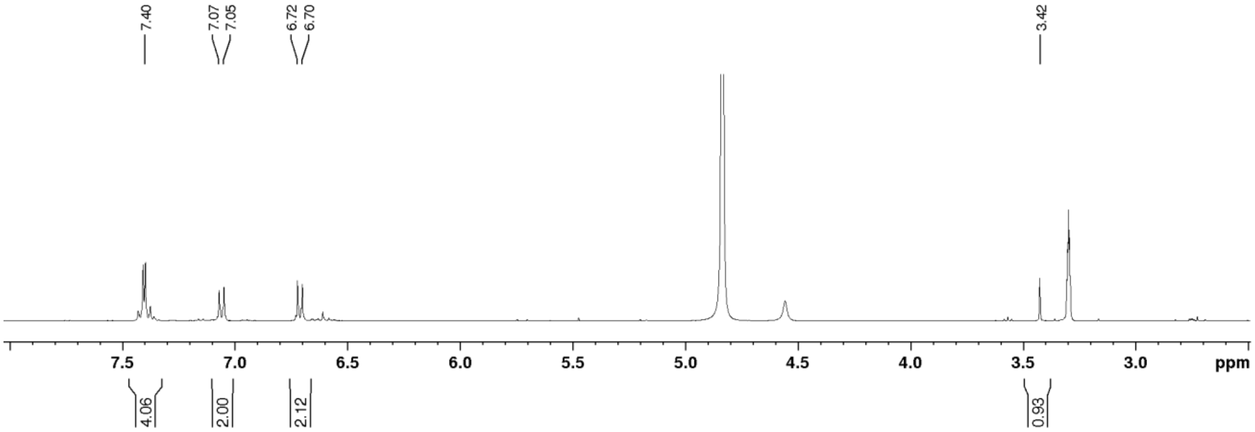

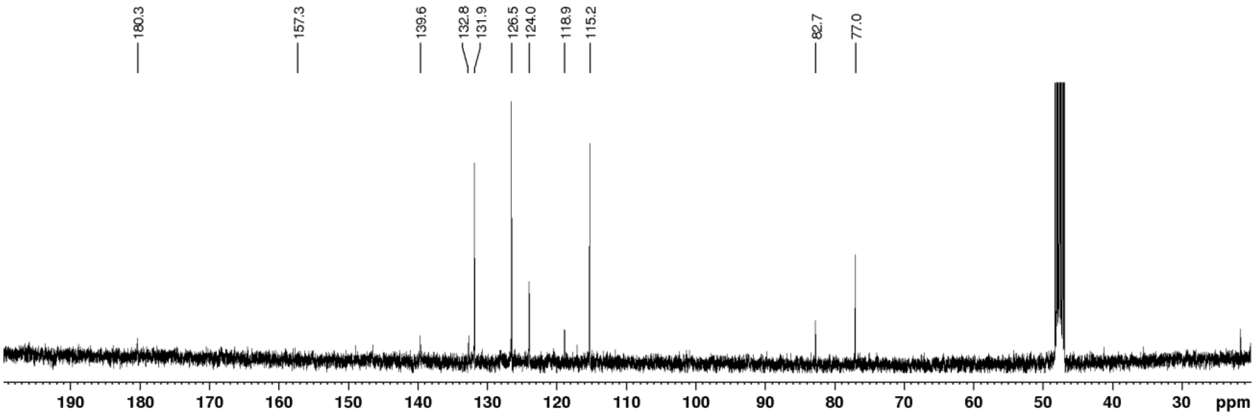

Compound **13**

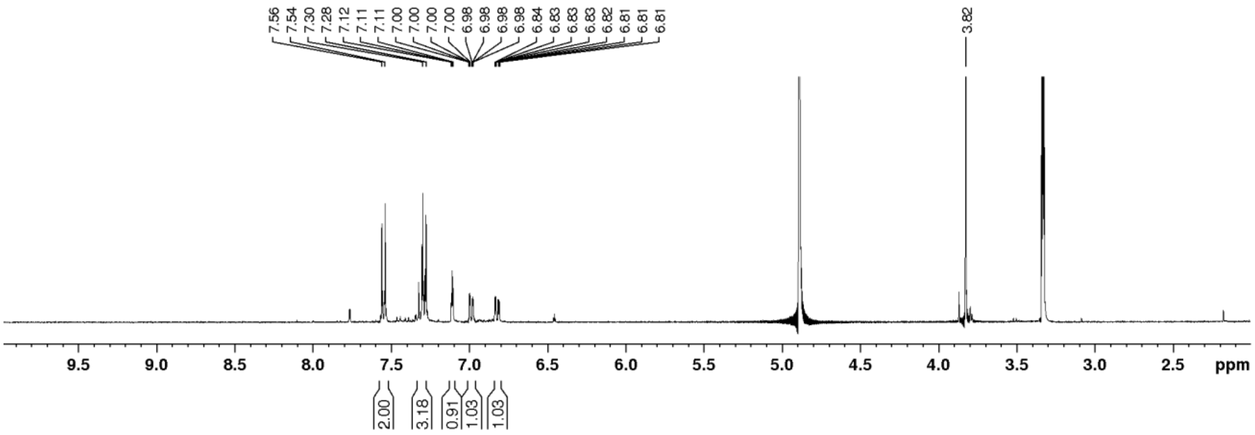

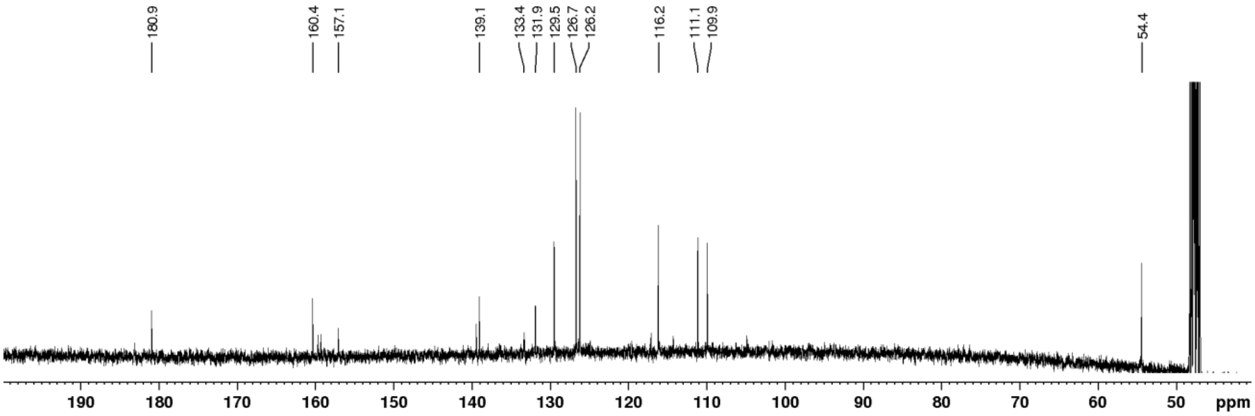
180.9
160.4
157.1
139.1
133.4
131.9
129.5
126.7
126.2
116.2
111.1
109.9
54.4
190 180 170 160 150 140 130 120 110 100 90 80 70 60 50 ppm

Compound **14**

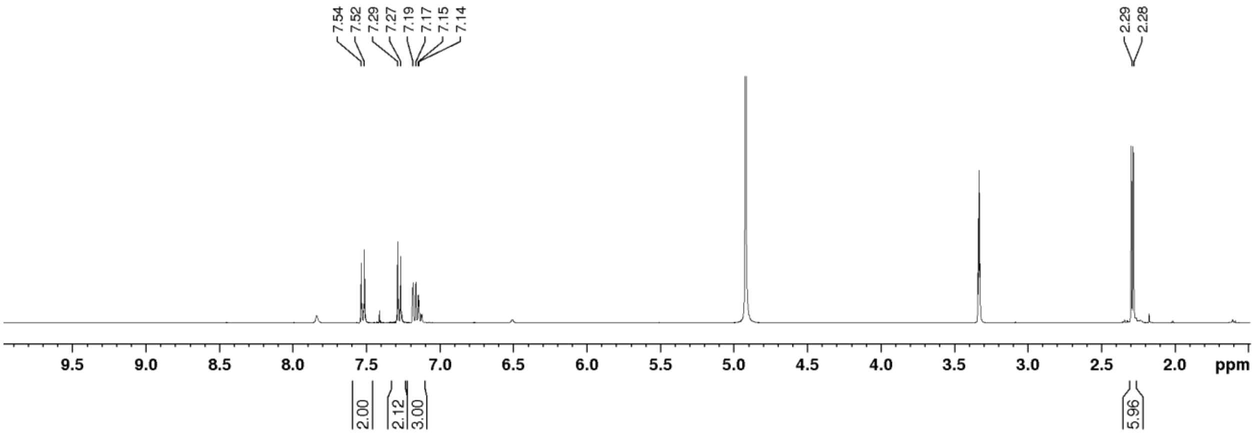
7.54
7.52
7.29
7.27
7.19
7.17
7.15
7.14
2.29
2.28
9.5 9.0 8.5 8.0 7.5 7.0 6.5 6.0 5.5 5.0 4.5 4.0 3.5 3.0 2.5 2.0 ppm
2.00
2.12
3.00
5.96

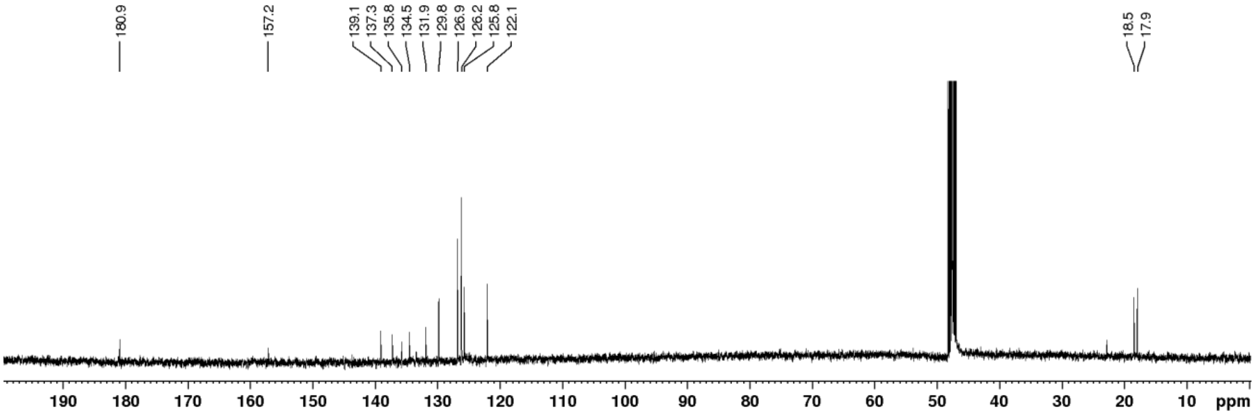
180.9
157.2
139.1
137.3
135.8
134.5
131.9
129.8
126.9
126.2
125.8
122.1
18.5
17.9
190 180 170 160 150 140 130 120 110 100 90 80 70 60 50 40 30 20 10 ppm

Compound **15**

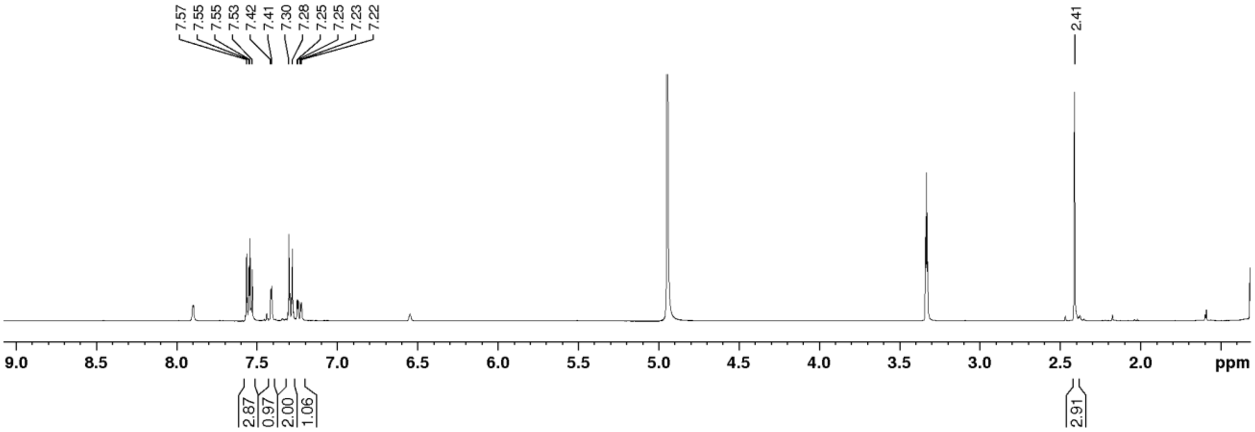

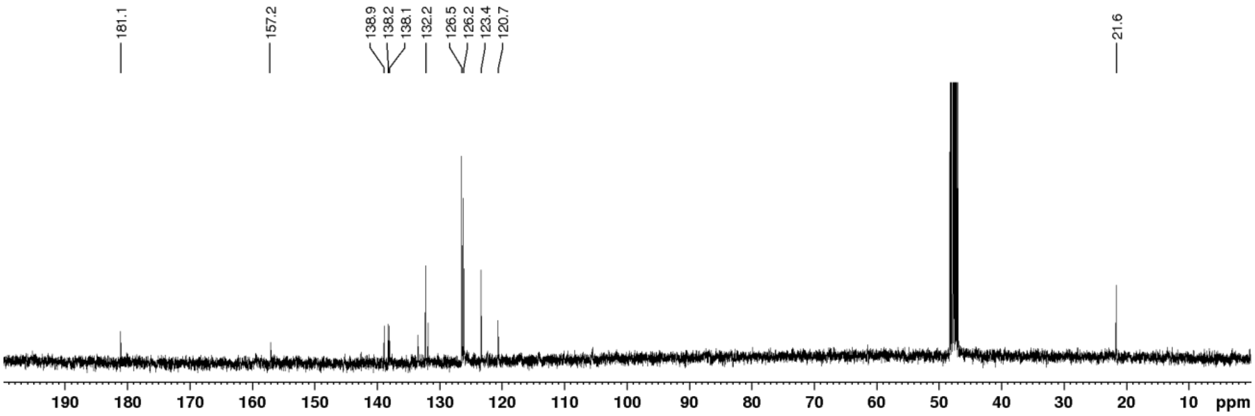

Compound **16**

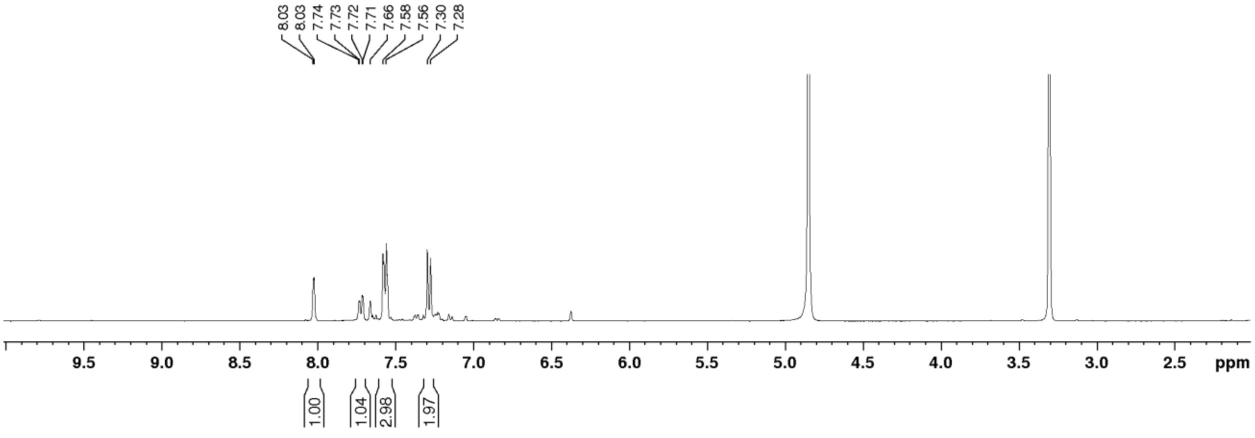

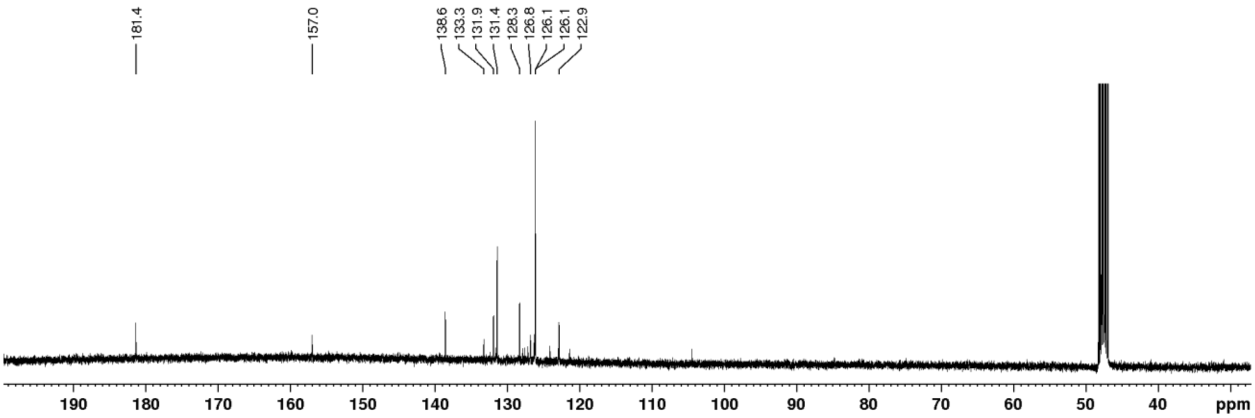
181.4
157.0
138.6
133.3
131.9
131.4
128.3
126.8
126.1
126.1
122.9
190 180 170 160 150 140 130 120 110 100 90 80 70 60 50 40 ppm

Compound **17**

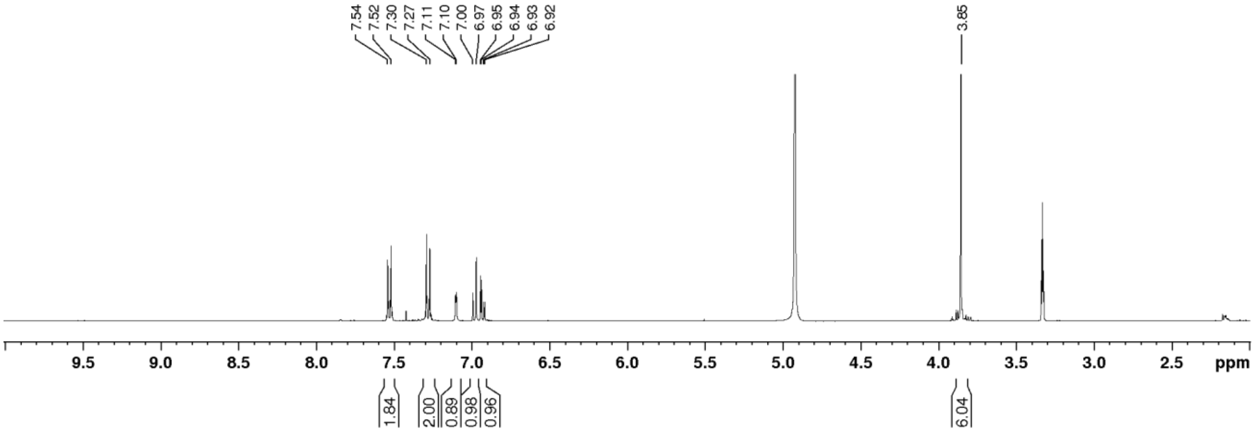
7.54
7.52
7.30
7.27
7.11
7.10
7.00
6.97
6.95
6.94
6.93
6.92
3.85
9.5 9.0 8.5 8.0 7.5 7.0 6.5 6.0 5.5 5.0 4.5 4.0 3.5 3.0 2.5 ppm
1.84
2.00
0.89
0.98
0.96
6.04

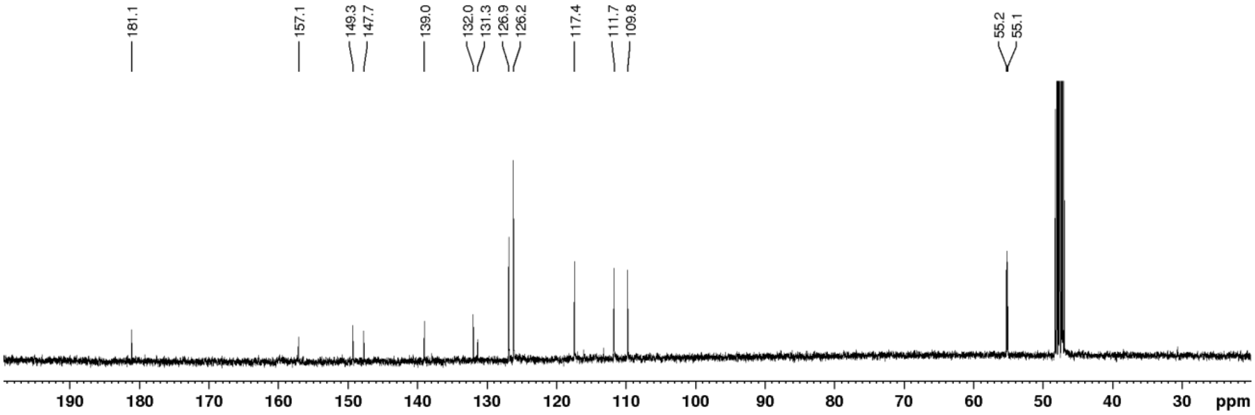
181.1
157.1
149.3
147.7
139.0
132.0
131.3
126.9
126.2
117.4
111.7
109.8
55.2
55.1
190 180 170 160 150 140 130 120 110 100 90 80 70 60 50 40 30 ppm

Compound **18**

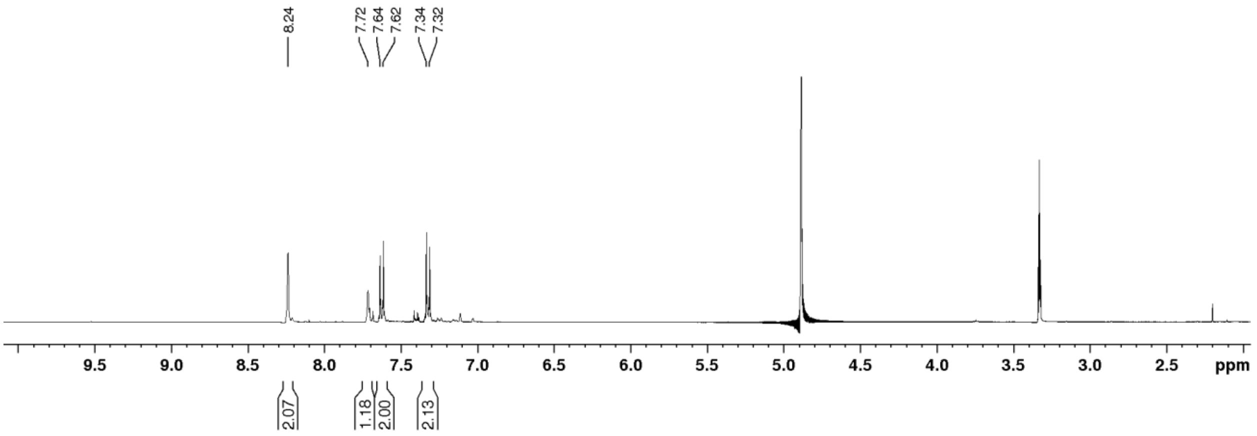

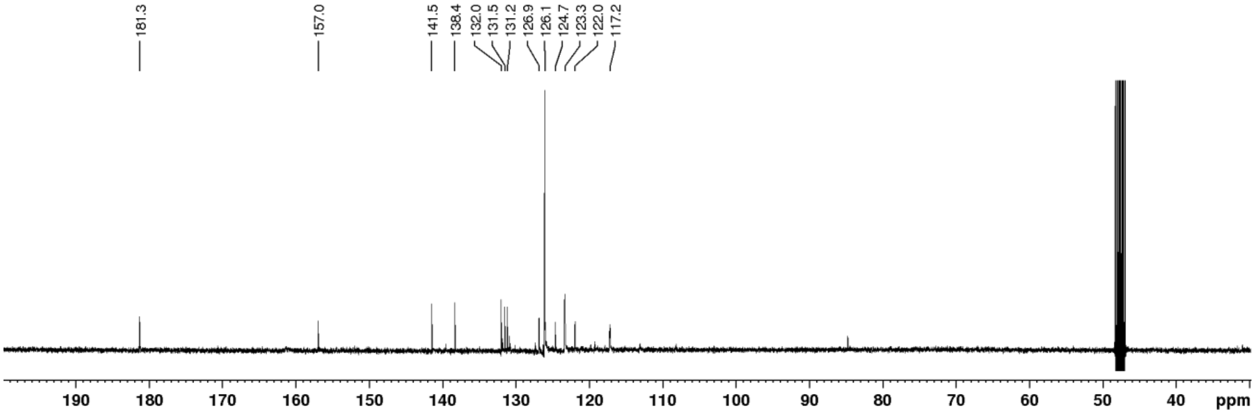

Compound **19**

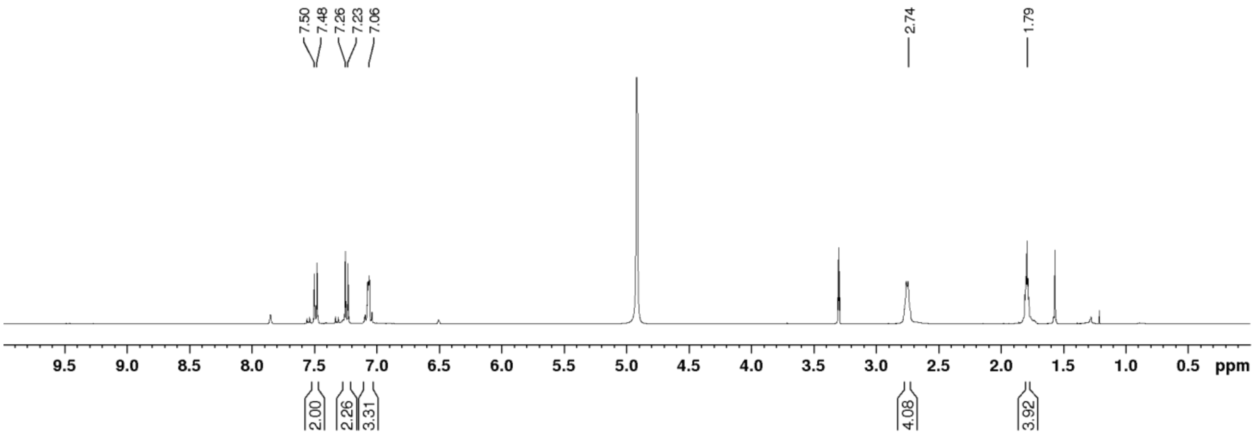

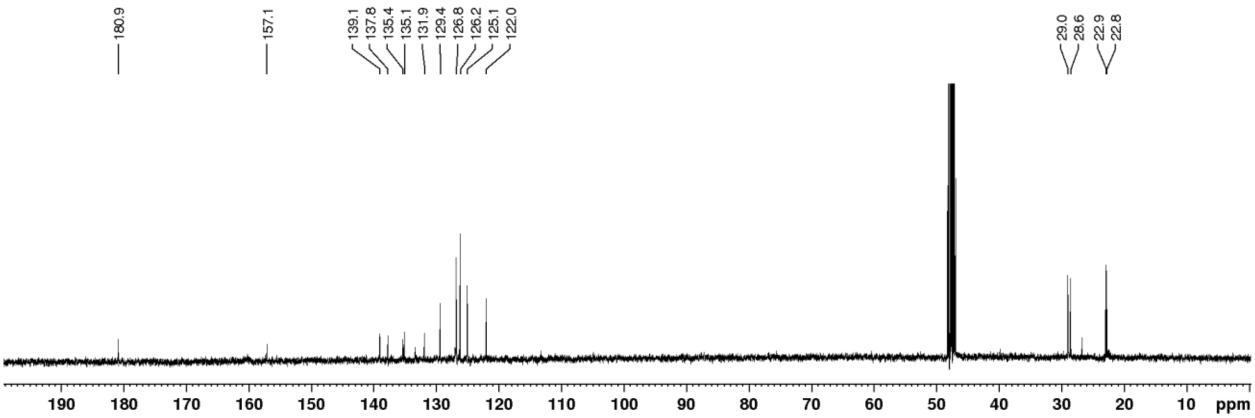
180.9
157.1
139.1
137.8
135.4
135.1
131.9
129.4
126.8
126.2
125.1
122.0
29.0
28.6
22.9
22.8
190 180 170 160 150 140 130 120 110 100 90 80 70 60 50 40 30 20 10 ppm

Compound **20**

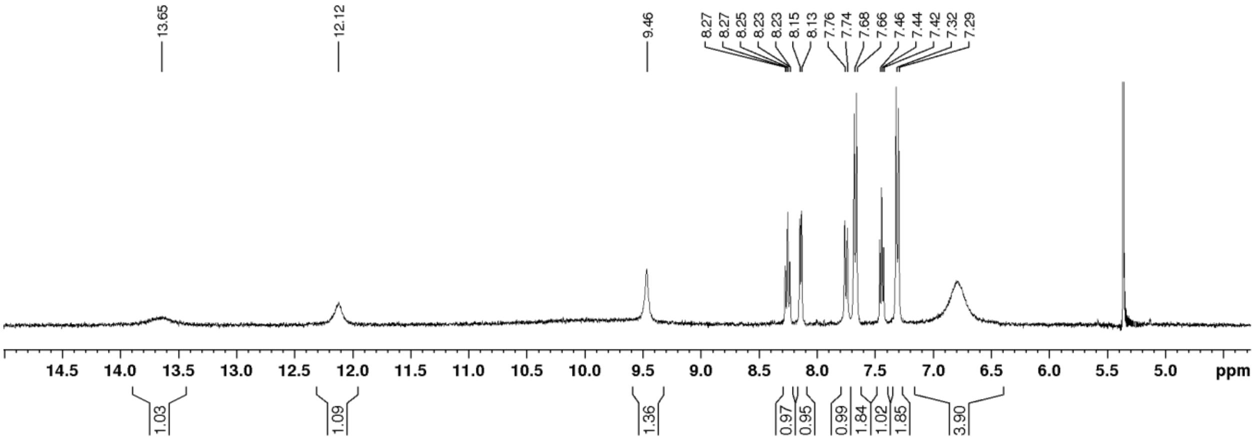
13.65
12.12
9.46
8.27
8.27
8.25
8.23
8.23
8.15
8.13
7.76
7.74
7.68
7.66
7.46
7.44
7.42
7.32
7.29
14.5 14.0 13.5 13.0 12.5 12.0 11.5 11.0 10.5 10.0 9.5 9.0 8.5 8.0 7.5 7.0 6.5 6.0 5.5 5.0 ppm
1.03
1.09
1.36
0.97
0.95
0.99
1.84
1.02
1.85
3.90

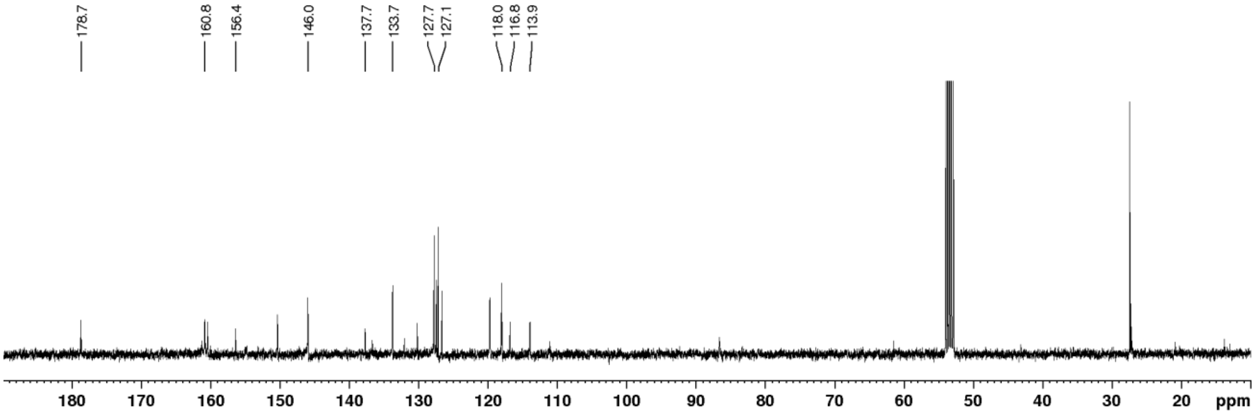
178.7
160.8
156.4
146.0
137.7
133.7
127.7
127.1
118.0
116.8
113.9
180 170 160 150 140 130 120 110 100 90 80 70 60 50 40 30 20 ppm

Compound **21**

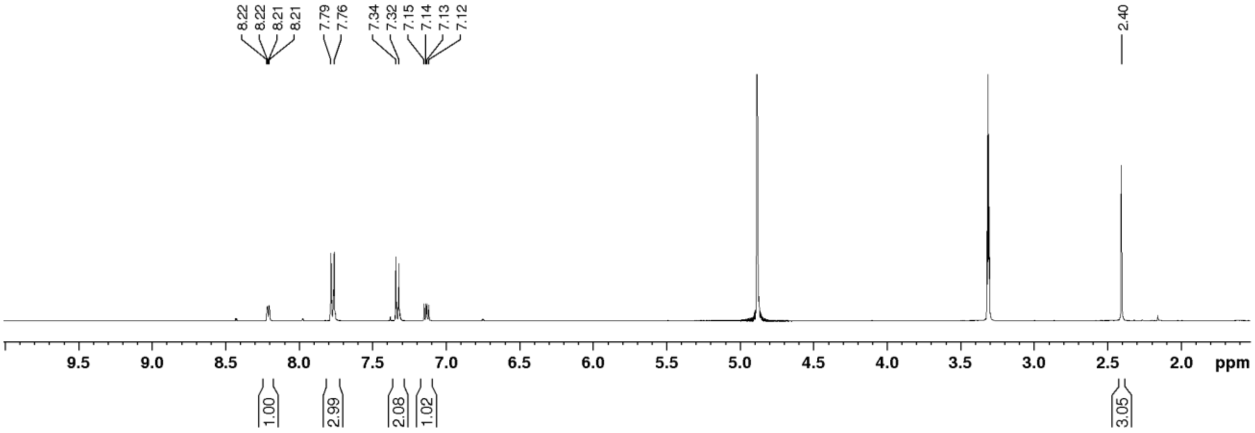

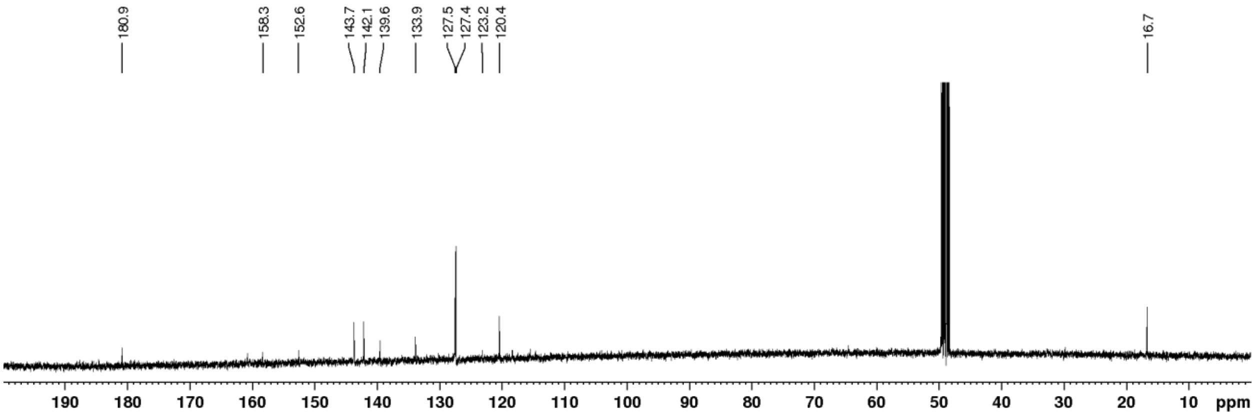

Compound **22**

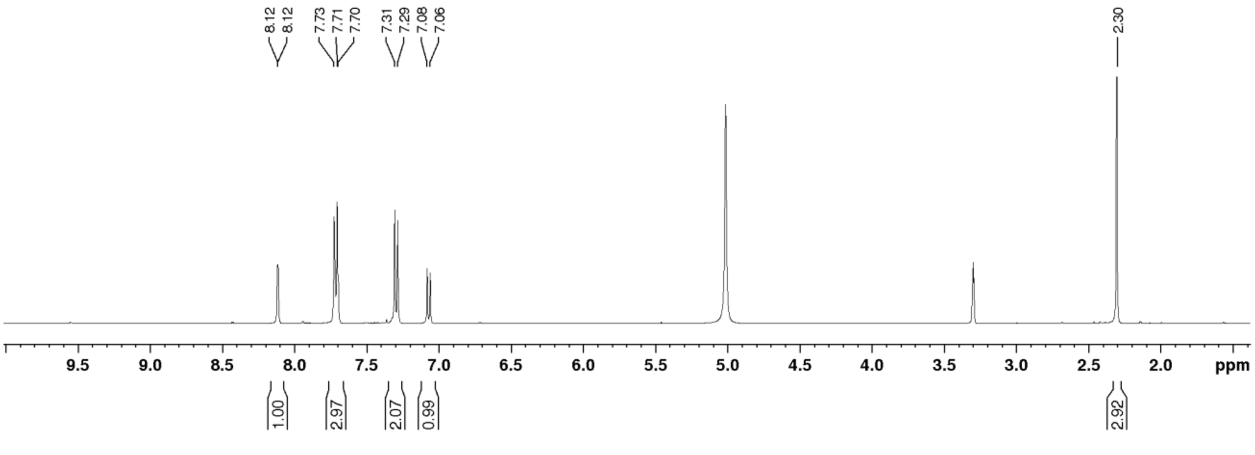

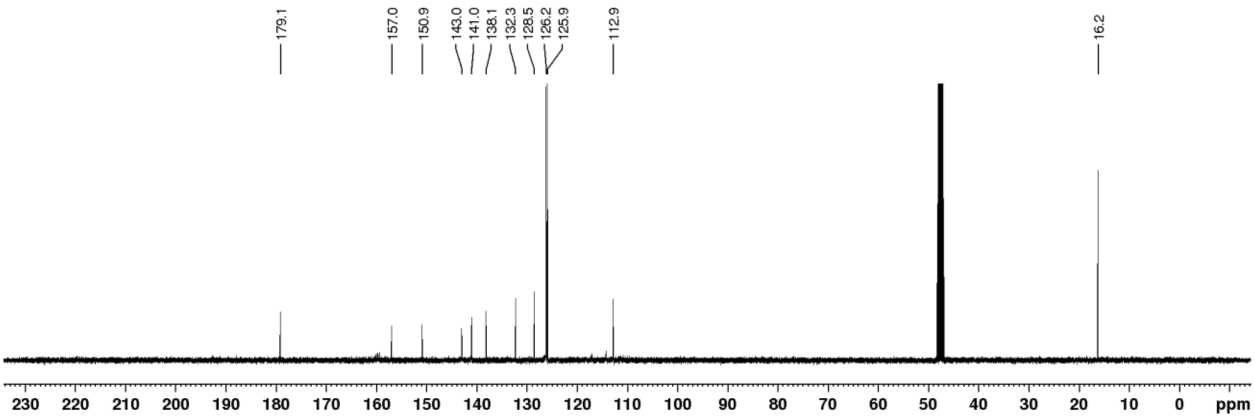
179.1 157.0 150.9 143.0 141.0 138.1 132.3 128.5 126.2 125.9 112.9 16.2
230 220 210 200 190 180 170 160 150 140 130 120 110 100 90 80 70 60 50 40 30 20 10 0 ppm

Compound **23**

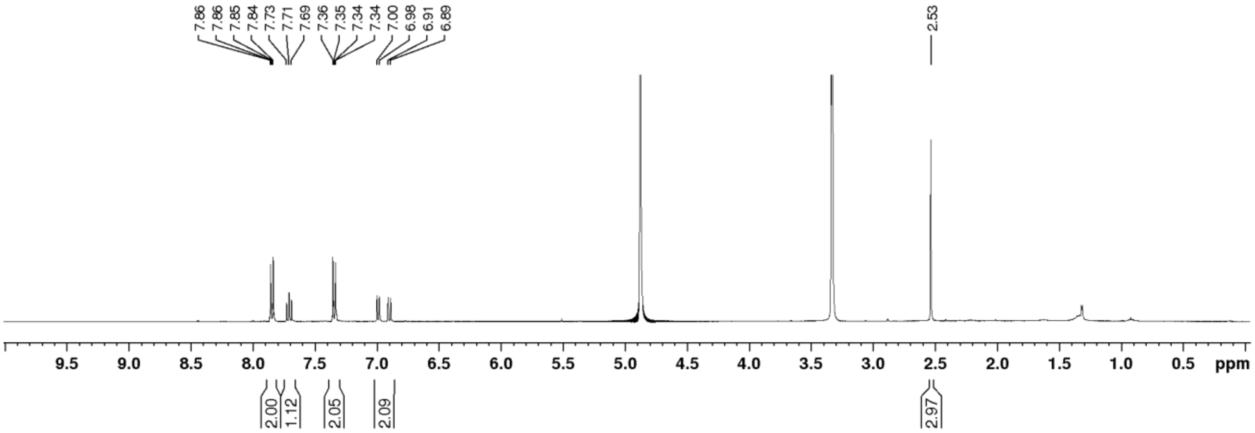
7.86 7.86 7.85 7.84 7.73 7.71 7.69 7.36 7.35 7.34 7.34 7.00 6.98 6.91 6.89
2.53
9.5 9.0 8.5 8.0 7.5 7.0 6.5 6.0 5.5 5.0 4.5 4.0 3.5 3.0 2.5 2.0 1.5 1.0 0.5 ppm
2.00 1.12 2.05 2.09 2.97

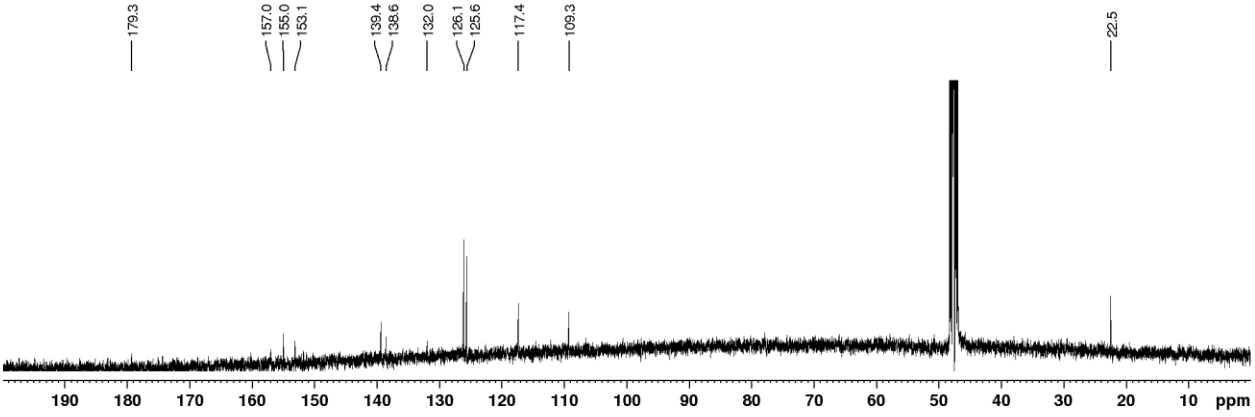
179.3 157.0 155.0 153.1 139.4 138.6 132.0 126.1 125.6 117.4 109.3 22.5
190 180 170 160 150 140 130 120 110 100 90 80 70 60 50 40 30 20 10 ppm

Compound **24**

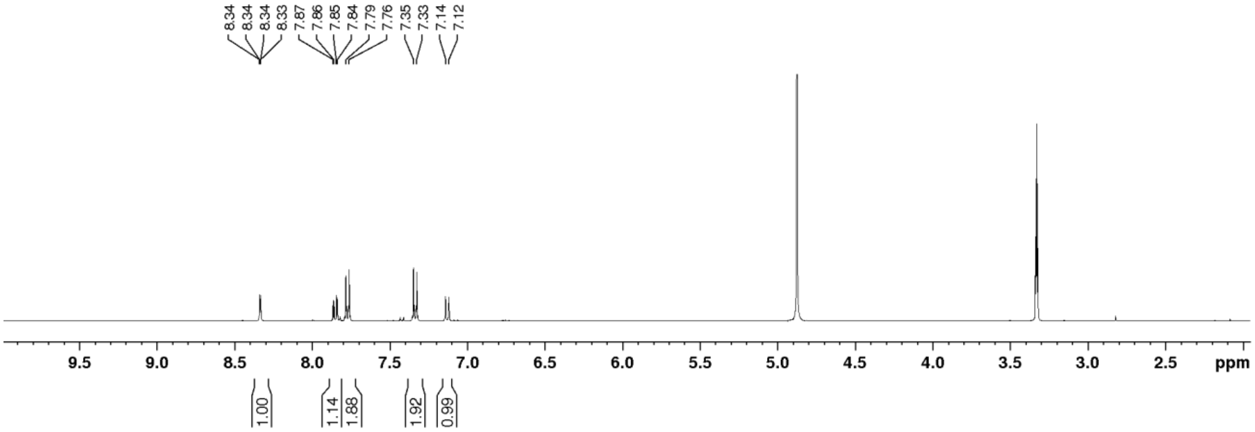

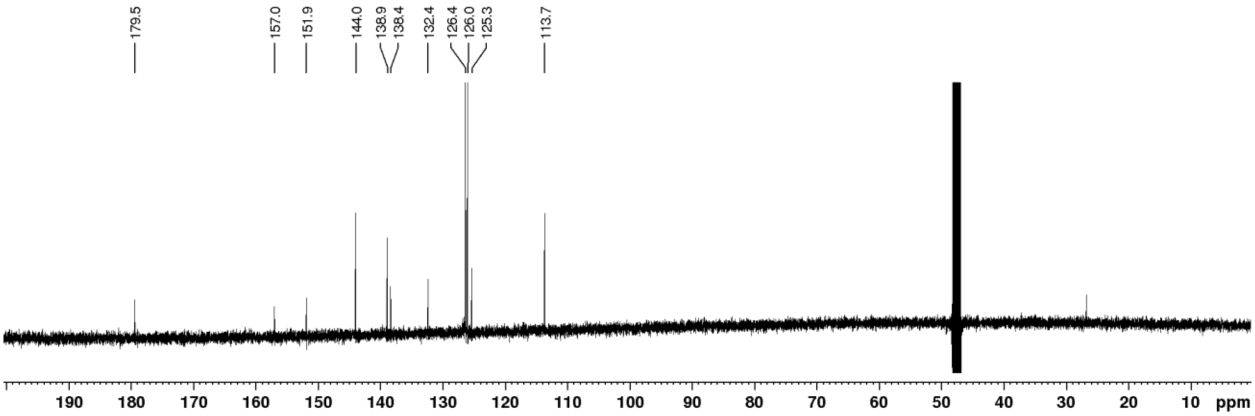

Compound **25**

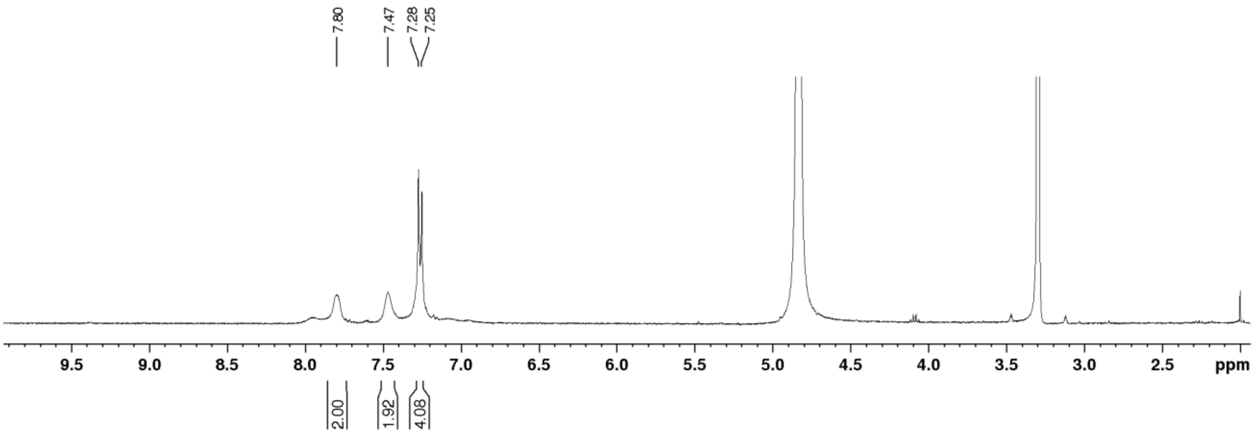

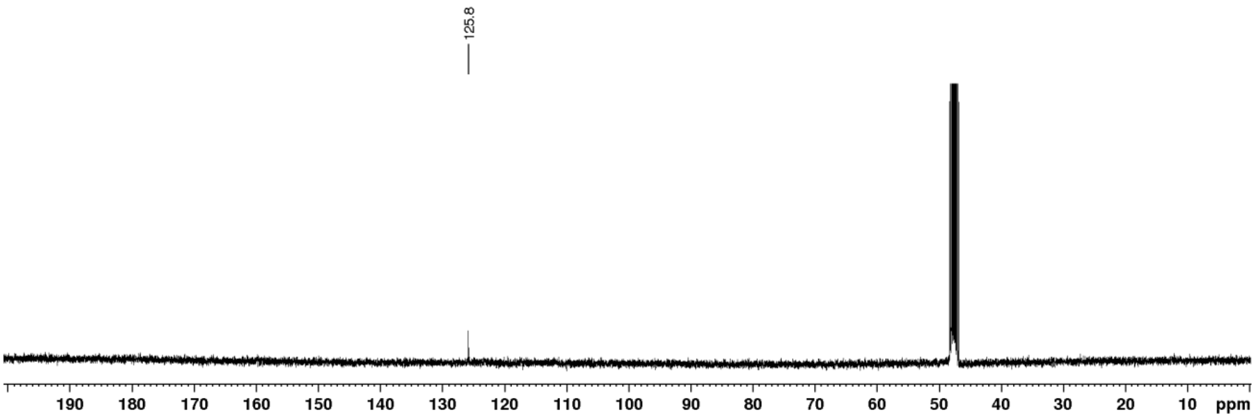
125.8
190
180
170
160
150
140
130
120
110
100
90
80
70
60
50
40
30
20
10
ppm

7. SwissADME results

Compound 1

Molecule 1

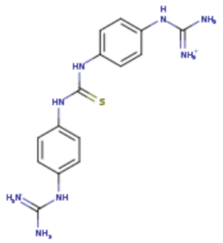

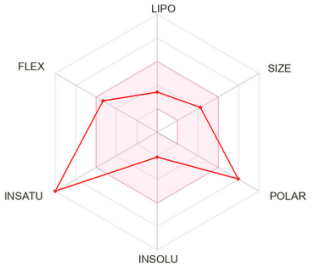

SMILES S=C(Nc1ccc(cc1)NC(=[NH2+])N)Nc1ccc(cc1)NC(=[NH2+])N

Physicochemical Properties	
Formula	C15H20N8S++
Molecular weight	344.44 g/mol
Num. heavy atoms	24
Num. arom. heavy atoms	12
Fraction Csp3	0.00
Num. rotatable bonds	8
Num. H-bond acceptors	0
Num. H-bond donors	8
Molar Refractivity	104.91
TPSA	183.43 Å²

Lipophilicity	
Log $P_{o/w}$ (iLOGP)	1.10
Log $P_{o/w}$ (XLOGP3)	0.47
Log $P_{o/w}$ (WLOGP)	-2.30
Log $P_{o/w}$ (MLOGP)	1.39
Log $P_{o/w}$ (SILICOS-IT)	0.38
Consensus Log $P_{o/w}$	0.21

Water Solubility	
Log *S* (ESOL)	-2.11
Solubility	2.65e+00 mg/ml ; 7.70e-03 mol/l
Class	Soluble
Log *S* (Ali)	-3.89
Solubility	4.43e-02 mg/ml ; 1.29e-04 mol/l
Class	Soluble
Log *S* (SILICOS-IT)	-4.71
Solubility	6.75e-03 mg/ml ; 1.96e-05 mol/l
Class	Moderately soluble

Pharmacokinetics	
GI absorption	Low
BBB permeant	No
P-gp substrate	No
CYP1A2 inhibitor	No
CYP2C19 inhibitor	No
CYP2C9 inhibitor	No
CYP2D6 inhibitor	No
CYP3A4 inhibitor	No
Log K_p (skin permeation)	-8.07 cm/s

Druglikeness	
Lipinski	Yes; 1 violation: NHorOH>5
Ghose	No; 1 violation: WLOGP<-0.4
Veber	No; 1 violation: TPSA>140
Egan	No; 1 violation: TPSA>131.6
Muegge	No; 2 violations: TPSA>150, H-don>5
Bioavailability Score	0.55

Medicinal Chemistry	
PAINS	0 alert
Brenk	3 alerts: imine_1, imine_2, thiocarbonyl_group
Leadlikeness	No; 1 violation: Rotors>7
Synthetic accessibility	2.68

Compound 2

Molecule 1

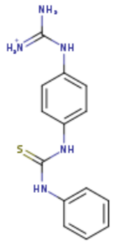

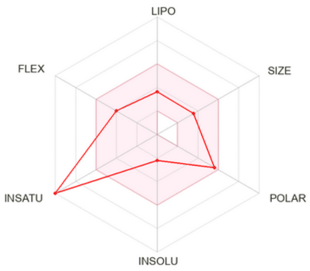

SMILES S=C(Nc1ccccc1)Nc1ccc(cc1)NC(=[NH2+])N

Physicochemical Properties	
Formula	C14H16N5S+
Molecular weight	286.38 g/mol
Num. heavy atoms	20
Num. arom. heavy atoms	12
Fraction Csp3	0.00
Num. rotatable bonds	6
Num. H-bond acceptors	0
Num. H-bond donors	5
Molar Refractivity	88.64
TPSA	119.79 Å²

Lipophilicity	
Log $P_{o/w}$ (iLOGP)	1.54
Log $P_{o/w}$ (XLOGP3)	0.86
Log $P_{o/w}$ (WLOGP)	0.41
Log $P_{o/w}$ (MLOGP)	2.12
Log $P_{o/w}$ (SILICOS-IT)	1.78
Consensus Log $P_{o/w}$	1.34

Water Solubility	
Log *S* (ESOL)	-2.21
Solubility	1.78e+00 mg/ml ; 6.23e-03 mol/l
Class	Soluble
Log *S* (Ali)	-2.96
Solubility	3.15e-01 mg/ml ; 1.10e-03 mol/l
Class	Soluble
Log *S* (SILICOS-IT)	-4.95
Solubility	3.21e-03 mg/ml ; 1.12e-05 mol/l
Class	Moderately soluble

Pharmacokinetics	
GI absorption	High
BBB permeant	No
P-gp substrate	No
CYP1A2 inhibitor	No
CYP2C19 inhibitor	No
CYP2C9 inhibitor	No
CYP2D6 inhibitor	No
CYP3A4 inhibitor	No
Log K_p (skin permeation)	-7.44 cm/s

Druglikeness	
Lipinski	Yes; 0 violation
Ghose	Yes
Veber	Yes
Egan	Yes
Muegge	Yes
Bioavailability Score	0.55

Medicinal Chemistry	
PAINS	0 alert
Brenk	3 alerts: imine_1, imine_2, thiocarbonyl_group
Leadlikeness	Yes
Synthetic accessibility	2.39

Compound 3

Molecule 1

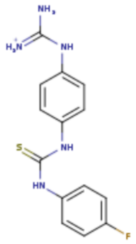

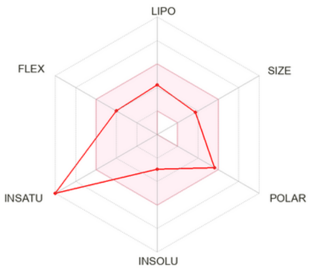

SMILES S=C(Nc1ccc(cc1)F)Nc1ccc(cc1)NC(=[NH2+])N

Physicochemical Properties	
Formula	C14H15FN5S+
Molecular weight	304.37 g/mol
Num. heavy atoms	21
Num. arom. heavy atoms	12
Fraction Csp3	0.00
Num. rotatable bonds	6
Num. H-bond acceptors	1
Num. H-bond donors	5
Molar Refractivity	88.60
TPSA	119.79 Å²

Lipophilicity	
Log $P_{o/w}$ (iLOGP)	1.58
Log $P_{o/w}$ (XLOGP3)	1.88
Log $P_{o/w}$ (WLOGP)	0.97
Log $P_{o/w}$ (MLOGP)	2.52
Log $P_{o/w}$ (SILICOS-IT)	2.21
Consensus Log $P_{o/w}$	1.83

Water Solubility	
Log *S* (ESOL)	-2.94
Solubility	3.51e-01 mg/ml ; 1.15e-03 mol/l
Class	Soluble
Log *S* (Ali)	-4.02
Solubility	2.92e-02 mg/ml ; 9.60e-05 mol/l
Class	Moderately soluble
Log *S* (SILICOS-IT)	-5.22
Solubility	1.82e-03 mg/ml ; 5.98e-06 mol/l
Class	Moderately soluble

Pharmacokinetics	
GI absorption	High
BBB permeant	No
P-gp substrate	No
CYP1A2 inhibitor	No
CYP2C19 inhibitor	No
CYP2C9 inhibitor	No
CYP2D6 inhibitor	No
CYP3A4 inhibitor	No
Log K_p (skin permeation)	-6.82 cm/s

Druglikeness	
Lipinski	Yes; 0 violation
Ghose	Yes
Veber	Yes
Egan	Yes
Muegge	Yes
Bioavailability Score	0.55

Medicinal Chemistry	
PAINS	0 alert
Brenk	3 alerts: imine_1, imine_2, thiocarbonyl_group
Leadlikeness	Yes
Synthetic accessibility	2.38

Compound 4

Molecule 1

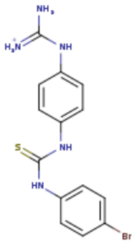

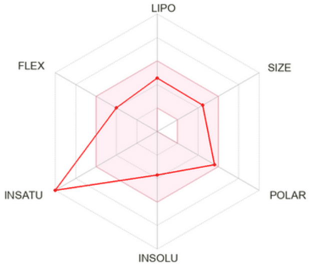

SMILES S=C(Nc1ccc(cc1)Br)Nc1ccc(cc1)NC(=[NH2+])N

Physicochemical Properties	
Formula	C14H15BrN5S+
Molecular weight	365.27 g/mol
Num. heavy atoms	21
Num. arom. heavy atoms	12
Fraction Csp3	0.00
Num. rotatable bonds	6
Num. H-bond acceptors	0
Num. H-bond donors	5
Molar Refractivity	96.34
TPSA	119.79 Å²

Lipophilicity	
Log $P_{o/w}$ (iLOGP)	1.86
Log $P_{o/w}$ (XLOGP3)	2.47
Log $P_{o/w}$ (WLOGP)	1.17
Log $P_{o/w}$ (MLOGP)	2.77
Log $P_{o/w}$ (SILICOS-IT)	2.47
Consensus Log $P_{o/w}$	2.15

Water Solubility	
Log *S* (ESOL)	-3.69
Solubility	7.50e-02 mg/ml ; 2.05e-04 mol/l
Class	Soluble
Log *S* (Ali)	-4.63
Solubility	8.56e-03 mg/ml ; 2.34e-05 mol/l
Class	Moderately soluble
Log *S* (SILICOS-IT)	-5.76
Solubility	6.38e-04 mg/ml ; 1.75e-06 mol/l
Class	Moderately soluble

Pharmacokinetics	
GI absorption	High
BBB permeant	No
P-gp substrate	No
CYP1A2 inhibitor	No
CYP2C19 inhibitor	No
CYP2C9 inhibitor	Yes
CYP2D6 inhibitor	No
CYP3A4 inhibitor	Yes
Log K_p (skin permeation)	-6.77 cm/s

Druglikeness	
Lipinski	Yes; 0 violation
Ghose	Yes
Veber	Yes
Egan	Yes
Muegge	Yes
Bioavailability Score	0.55

Medicinal Chemistry	
PAINS	0 alert
Brenk	3 alerts: imine_1, imine_2, thiocarbonyl_group
Leadlikeness	No; 1 violation: MW>350
Synthetic accessibility	2.57

Compound 5

Molecule 1

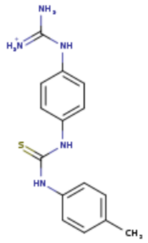

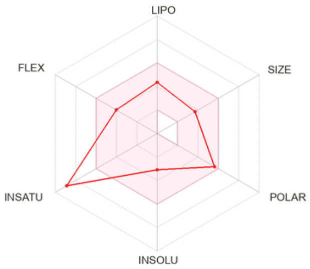

SMILES S=C(Nc1ccc(cc1)C)Nc1ccc(cc1)NC(=[NH2+])N

Physicochemical Properties	
Formula	C15H18N5S+
Molecular weight	300.40 g/mol
Num. heavy atoms	21
Num. arom. heavy atoms	12
Fraction Csp3	0.07
Num. rotatable bonds	6
Num. H-bond acceptors	0
Num. H-bond donors	5
Molar Refractivity	93.60
TPSA	119.79 Å²

Lipophilicity	
Log $P_{o/w}$ (iLOGP)	1.85
Log $P_{o/w}$ (XLOGP3)	2.14
Log $P_{o/w}$ (WLOGP)	0.72
Log $P_{o/w}$ (MLOGP)	2.38
Log $P_{o/w}$ (SILICOS-IT)	2.29
Consensus Log $P_{o/w}$	1.88

Water Solubility	
Log *S* (ESOL)	-3.08
Solubility	2.51e-01 mg/ml ; 8.36e-04 mol/l
Class	Soluble
Log *S* (Ali)	-4.29
Solubility	1.55e-02 mg/ml ; 5.16e-05 mol/l
Class	Moderately soluble
Log *S* (SILICOS-IT)	-5.33
Solubility	1.40e-03 mg/ml ; 4.65e-06 mol/l
Class	Moderately soluble

Pharmacokinetics	
GI absorption	High
BBB permeant	No
P-gp substrate	No
CYP1A2 inhibitor	No
CYP2C19 inhibitor	No
CYP2C9 inhibitor	No
CYP2D6 inhibitor	No
CYP3A4 inhibitor	No
Log K_p (skin permeation)	-6.61 cm/s

Druglikeness	
Lipinski	Yes; 0 violation
Ghose	Yes
Veber	Yes
Egan	Yes
Muegge	Yes
Bioavailability Score	0.55

Medicinal Chemistry	
PAINS	0 alert
Brenk	3 alerts: imine_1, imine_2, thiocarbonyl_group
Leadlikeness	Yes
Synthetic accessibility	2.29

Compound 6

Molecule 1

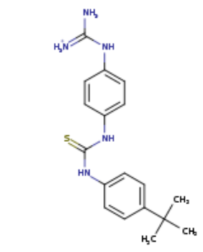

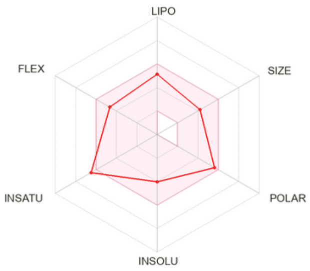

SMILES S=C(Nc1ccc(cc1)C(C)(C)C)Nc1ccc(cc1)NC(=[NH2+])N

Physicochemical Properties	
Formula	C18H24N5S+
Molecular weight	342.48 g/mol
Num. heavy atoms	24
Num. arom. heavy atoms	12
Fraction Csp3	0.22
Num. rotatable bonds	7
Num. H-bond acceptors	0
Num. H-bond donors	5
Molar Refractivity	107.91
TPSA	119.79 Å²

Lipophilicity	
Log $P_{o/w}$ (iLOGP)	2.59
Log $P_{o/w}$ (XLOGP3)	3.45
Log $P_{o/w}$ (WLOGP)	1.71
Log $P_{o/w}$ (MLOGP)	3.11
Log $P_{o/w}$ (SILICOS-IT)	3.14
Consensus Log $P_{o/w}$	2.80

Water Solubility	
Log S (ESOL)	-4.04
Solubility	3.09e-02 mg/ml ; 9.02e-05 mol/l
Class	Moderately soluble
Log S (Ali)	-5.65
Solubility	7.72e-04 mg/ml ; 2.25e-06 mol/l
Class	Moderately soluble
Log S (SILICOS-IT)	-6.13
Solubility	2.53e-04 mg/ml ; 7.38e-07 mol/l
Class	Poorly soluble

Pharmacokinetics	
GI absorption	High
BBB permeant	No
P-gp substrate	No
CYP1A2 inhibitor	No
CYP2C19 inhibitor	Yes
CYP2C9 inhibitor	Yes
CYP2D6 inhibitor	Yes
CYP3A4 inhibitor	Yes
Log K_p (skin permeation)	-5.94 cm/s

Druglikeness	
Lipinski	Yes; 0 violation
Ghose	Yes
Veber	Yes
Egan	Yes
Muegge	Yes
Bioavailability Score	0.55

Medicinal Chemistry	
PAINS	0 alert
Brenk	3 alerts: imine_1, imine_2, thiocarbonyl_group
Leadlikeness	Yes
Synthetic accessibility	2.57

Compound 7

Molecule 1

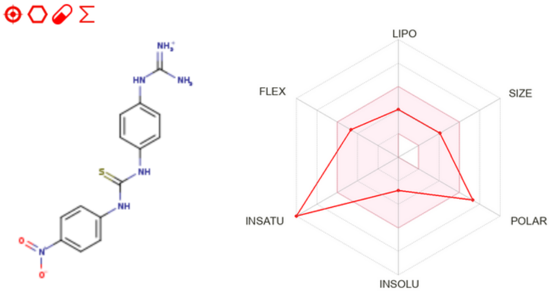

SMILES S=C(Nc1ccc(cc1)[N+](=O)[O-])Nc1ccc(cc1)NC(=[NH2+])N

Physicochemical Properties	
Formula	C14H15N6O2S+
Molecular weight	331.37 g/mol
Num. heavy atoms	23
Num. arom. heavy atoms	12
Fraction Csp3	0.00
Num. rotatable bonds	7
Num. H-bond acceptors	2
Num. H-bond donors	5
Molar Refractivity	97.46
TPSA	165.61 Å²

Lipophilicity	
Log $P_{o/w}$ (iLOGP)	1.18
Log $P_{o/w}$ (XLOGP3)	1.61
Log $P_{o/w}$ (WLOGP)	0.32
Log $P_{o/w}$ (MLOGP)	1.20
Log $P_{o/w}$ (SILICOS-IT)	-0.34
Consensus Log $P_{o/w}$	0.79

Water Solubility	
Log *S* (ESOL)	-2.83
Solubility	4.87e-01 mg/ml ; 1.47e-03 mol/l
Class	Soluble
Log *S* (Ali)	-4.70
Solubility	6.62e-03 mg/ml ; 2.00e-05 mol/l
Class	Moderately soluble
Log *S* (SILICOS-IT)	-4.31
Solubility	1.63e-02 mg/ml ; 4.91e-05 mol/l
Class	Moderately soluble

Pharmacokinetics	
GI absorption	Low
BBB permeant	No
P-gp substrate	No
CYP1A2 inhibitor	No
CYP2C19 inhibitor	No
CYP2C9 inhibitor	No
CYP2D6 inhibitor	No
CYP3A4 inhibitor	No
Log K_p (skin permeation)	-7.18 cm/s

Druglikeness	
Lipinski	Yes; 0 violation
Ghose	Yes
Veber	No; 1 violation: TPSA>140
Egan	No; 1 violation: TPSA>131.6
Muegge	No; 1 violation: TPSA>150
Bioavailability Score	0.55

Medicinal Chemistry	
PAINS	0 alert
Brenk	5 alerts: imine_1, imine_2, nitro_group, oxygen-nitrogen_single_bond, thiocarbonyl_group
Leadlikeness	Yes
Synthetic accessibility	2.72

Compound 8

Molecule 1

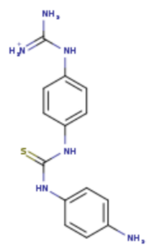

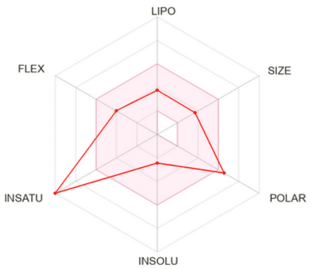

SMILES S=C(Nc1ccc(cc1)N)Nc1ccc(cc1)NC(=[NH2+])N

Physicochemical Properties	
Formula	C14H17N6S+
Molecular weight	301.39 g/mol
Num. heavy atoms	21
Num. arom. heavy atoms	12
Fraction Csp3	0.00
Num. rotatable bonds	6
Num. H-bond acceptors	0
Num. H-bond donors	6
Molar Refractivity	93.04
TPSA	145.81 Å²

Lipophilicity	
Log $P_{o/w}$ (iLOGP)	1.25
Log $P_{o/w}$ (XLOGP3)	1.10
Log $P_{o/w}$ (WLOGP)	-0.00
Log $P_{o/w}$ (MLOGP)	1.58
Log $P_{o/w}$ (SILICOS-IT)	1.07
Consensus Log $P_{o/w}$	1.00

Water Solubility	
Log *S* (ESOL)	-2.43
Solubility	1.12e+00 mg/ml ; 3.73e-03 mol/l
Class	Soluble
Log *S* (Ali)	-3.75
Solubility	5.30e-02 mg/ml ; 1.76e-04 mol/l
Class	Soluble
Log *S* (SILICOS-IT)	-4.59
Solubility	7.82e-03 mg/ml ; 2.59e-05 mol/l
Class	Moderately soluble

Pharmacokinetics	
GI absorption	Low
BBB permeant	No
P-gp substrate	No
CYP1A2 inhibitor	No
CYP2C19 inhibitor	No
CYP2C9 inhibitor	No
CYP2D6 inhibitor	No
CYP3A4 inhibitor	No
Log K_p (skin permeation)	-7.36 cm/s

Druglikeness	
Lipinski	Yes; 1 violation: NHorOH>5
Ghose	Yes
Veber	No; 1 violation: TPSA>140
Egan	No; 1 violation: TPSA>131.6
Muegge	No; 1 violation: H-don>5
Bioavailability Score	0.55

Medicinal Chemistry	
PAINS	1 alert: anil_no_alk
Brenk	4 alerts: aniline, imine_1, imine_2, thiocarbonyl_group
Leadlikeness	Yes
Synthetic accessibility	2.46

Compound 9

Molecule 1

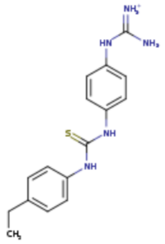

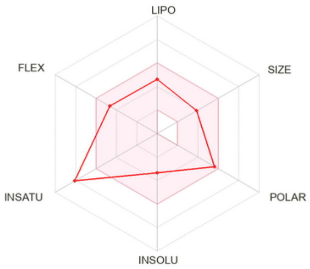

SMILES CCc1ccc(cc1)NC(=S)Nc1ccc(cc1)NC(=[NH2+])N

Physicochemical Properties	
Formula	C16H20N5S+
Molecular weight	314.43 g/mol
Num. heavy atoms	22
Num. arom. heavy atoms	12
Fraction Csp3	0.12
Num. rotatable bonds	7
Num. H-bond acceptors	0
Num. H-bond donors	5
Molar Refractivity	98.41
TPSA	119.79 Å²

Lipophilicity	
Log $P_{o/w}$ (iLOGP)	2.39
Log $P_{o/w}$ (XLOGP3)	2.57
Log $P_{o/w}$ (WLOGP)	0.97
Log $P_{o/w}$ (MLOGP)	2.63
Log $P_{o/w}$ (SILICOS-IT)	2.68
Consensus Log $P_{o/w}$	2.25

Water Solubility	
Log *S* (ESOL)	-3.35
Solubility	1.40e-01 mg/ml ; 4.46e-04 mol/l
Class	Soluble
Log *S* (Ali)	-4.73
Solubility	5.81e-03 mg/ml ; 1.85e-05 mol/l
Class	Moderately soluble
Log *S* (SILICOS-IT)	-5.73
Solubility	5.84e-04 mg/ml ; 1.86e-06 mol/l
Class	Moderately soluble

Pharmacokinetics	
GI absorption	High
BBB permeant	No
P-gp substrate	No
CYP1A2 inhibitor	No
CYP2C19 inhibitor	No
CYP2C9 inhibitor	No
CYP2D6 inhibitor	Yes
CYP3A4 inhibitor	No
Log K_p (skin permeation)	-6.39 cm/s

Druglikeness	
Lipinski	Yes; 0 violation
Ghose	Yes
Veber	Yes
Egan	Yes
Muegge	Yes
Bioavailability Score	0.55

Medicinal Chemistry	
PAINS	0 alert
Brenk	3 alerts: imine_1, imine_2, thiocarbonyl_group
Leadlikeness	Yes
Synthetic accessibility	2.35

Compound 10

Molecule 1

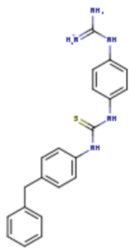

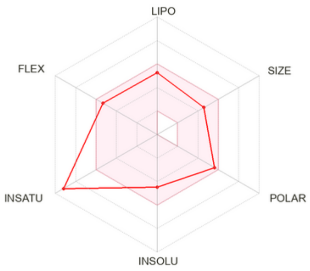

SMILES S=C(Nc1ccc(cc1)NC(=[NH2+])N)Nc1ccc(cc1)Cc1ccccc1

Physicochemical Properties	
Formula	C21H22N5S+
Molecular weight	376.50 g/mol
Num. heavy atoms	27
Num. arom. heavy atoms	18
Fraction Csp3	0.05
Num. rotatable bonds	8
Num. H-bond acceptors	0
Num. H-bond donors	5
Molar Refractivity	118.09
TPSA	119.79 Å²

Lipophilicity	
Log $P_{o/w}$ (iLOGP)	2.61
Log $P_{o/w}$ (XLOGP3)	3.70
Log $P_{o/w}$ (WLOGP)	2.00
Log $P_{o/w}$ (MLOGP)	3.57
Log $P_{o/w}$ (SILICOS-IT)	3.79
Consensus Log $P_{o/w}$	3.13

Water Solubility	
Log *S* (ESOL)	-4.47
Solubility	1.27e-02 mg/ml ; 3.38e-05 mol/l
Class	Moderately soluble
Log *S* (Ali)	-5.91
Solubility	4.67e-04 mg/ml ; 1.24e-06 mol/l
Class	Moderately soluble
Log *S* (SILICOS-IT)	-7.82
Solubility	5.71e-06 mg/ml ; 1.52e-08 mol/l
Class	Poorly soluble

Pharmacokinetics	
GI absorption	High
BBB permeant	No
P-gp substrate	No
CYP1A2 inhibitor	No
CYP2C19 inhibitor	Yes
CYP2C9 inhibitor	Yes
CYP2D6 inhibitor	No
CYP3A4 inhibitor	Yes
Log K_p (skin permeation)	-5.97 cm/s

Druglikeness	
Lipinski	Yes; 0 violation
Ghose	Yes
Veber	Yes
Egan	Yes
Muegge	Yes
Bioavailability Score	0.55

Medicinal Chemistry	
PAINS	0 alert
Brenk	3 alerts: imine_1, imine_2, thiocarbonyl_group
Leadlikeness	No; 3 violations: MW>350, Rotors>7, XLOGP3>3.5
Synthetic accessibility	2.75

Compound 11

Molecule 1

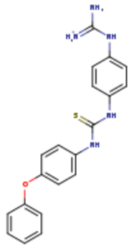

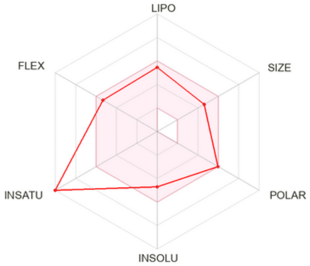

SMILES S=C(Nc1ccc(cc1)NC(=[NH2+])N)Nc1ccc(cc1)Oc1ccccc1

Physicochemical Properties	
Formula	C20H20N5OS+
Molecular weight	378.47 g/mol
Num. heavy atoms	27
Num. arom. heavy atoms	18
Fraction Csp3	0.00
Num. rotatable bonds	8
Num. H-bond acceptors	1
Num. H-bond donors	5
Molar Refractivity	115.15
TPSA	129.02 Å²

Lipophilicity	
Log $P_{o/w}$ (iLOGP)	2.63
Log $P_{o/w}$ (XLOGP3)	4.05
Log $P_{o/w}$ (WLOGP)	2.20
Log $P_{o/w}$ (MLOGP)	3.07
Log $P_{o/w}$ (SILICOS-IT)	2.94
Consensus Log $P_{o/w}$	2.98

Water Solubility	
Log *S* (ESOL)	-4.70
Solubility	7.49e-03 mg/ml ; 1.98e-05 mol/l
Class	Moderately soluble
Log *S* (Ali)	-6.46
Solubility	1.30e-04 mg/ml ; 3.44e-07 mol/l
Class	Poorly soluble
Log *S* (SILICOS-IT)	-7.15
Solubility	2.65e-05 mg/ml ; 7.01e-08 mol/l
Class	Poorly soluble

Pharmacokinetics	
GI absorption	High
BBB permeant	No
P-gp substrate	No
CYP1A2 inhibitor	No
CYP2C19 inhibitor	No
CYP2C9 inhibitor	Yes
CYP2D6 inhibitor	No
CYP3A4 inhibitor	No
Log K_p (skin permeation)	-5.73 cm/s

Druglikeness	
Lipinski	Yes; 0 violation
Ghose	Yes
Veber	Yes
Egan	Yes
Muegge	Yes
Bioavailability Score	0.55

Medicinal Chemistry	
PAINS	0 alert
Brenk	3 alerts: imine_1, imine_2, thiocarbonyl_group
Leadlikeness	No; 3 violations: MW>350, Rotors>7, XLOGP3>3.5
Synthetic accessibility	2.83

Compound 12

Molecule 1

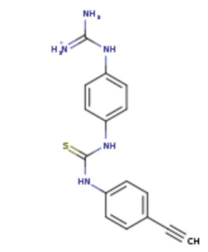

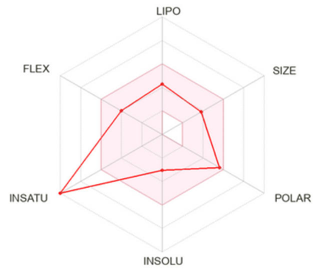

SMILES C#Cc1ccc(cc1)NC(=S)Nc1ccc(cc1)NC(=[NH2+])N

Physicochemical Properties	
Formula	C16H16N5S+
Molecular weight	310.40 g/mol
Num. heavy atoms	22
Num. arom. heavy atoms	12
Fraction Csp3	0.00
Num. rotatable bonds	6
Num. H-bond acceptors	0
Num. H-bond donors	5
Molar Refractivity	96.57
TPSA	119.79 Å²

Lipophilicity	
Log $P_{o/w}$ (iLOGP)	1.85
Log $P_{o/w}$ (XLOGP3)	2.02
Log $P_{o/w}$ (WLOGP)	0.47
Log $P_{o/w}$ (MLOGP)	2.55
Log $P_{o/w}$ (SILICOS-IT)	2.51
Consensus Log $P_{o/w}$	1.88

Water Solubility	
Log S (ESOL)	-3.04
Solubility	2.80e-01 mg/ml ; 9.02e-04 mol/l
Class	Soluble
Log S (Ali)	-4.16
Solubility	2.13e-02 mg/ml ; 6.87e-05 mol/l
Class	Moderately soluble
Log S (SILICOS-IT)	-5.02
Solubility	2.94e-03 mg/ml ; 9.48e-06 mol/l
Class	Moderately soluble

Pharmacokinetics	
GI absorption	High
BBB permeant	No
P-gp substrate	No
CYP1A2 inhibitor	No
CYP2C19 inhibitor	No
CYP2C9 inhibitor	No
CYP2D6 inhibitor	No
CYP3A4 inhibitor	No
Log K_p (skin permeation)	-6.76 cm/s

Druglikeness	
Lipinski	Yes; 0 violation
Ghose	Yes
Veber	Yes
Egan	Yes
Muegge	Yes
Bioavailability Score	0.55

Medicinal Chemistry	
PAINS	0 alert
Brenk	4 alerts: imine_1, imine_2, thiocarbonyl_group, triple_bond
Leadlikeness	Yes
Synthetic accessibility	2.59

Compound 13

Molecule 1

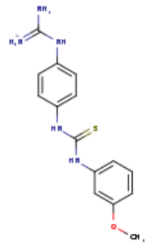

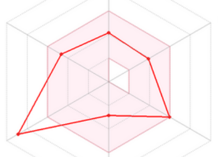

SMILES COc1cccc(c1)NC(=S)Nc1ccc(cc1)NC(=[NH2+])N

Physicochemical Properties	
Formula	C15H18N5OS+
Molecular weight	316.40 g/mol
Num. heavy atoms	22
Num. arom. heavy atoms	12
Fraction Csp3	0.07
Num. rotatable bonds	7
Num. H-bond acceptors	1
Num. H-bond donors	5
Molar Refractivity	95.13
TPSA	129.02 Å²

Lipophilicity	
Log $P_{o/w}$ (iLOGP)	2.15
Log $P_{o/w}$ (XLOGP3)	1.75
Log $P_{o/w}$ (WLOGP)	0.42
Log $P_{o/w}$ (MLOGP)	1.83
Log $P_{o/w}$ (SILICOS-IT)	1.84
Consensus Log $P_{o/w}$	1.60

Water Solubility	
Log *S* (ESOL)	-2.85
Solubility	4.51e-01 mg/ml ; 1.43e-03 mol/l
Class	Soluble
Log *S* (Ali)	-4.08
Solubility	2.65e-02 mg/ml ; 8.38e-05 mol/l
Class	Moderately soluble
Log *S* (SILICOS-IT)	-5.07
Solubility	2.72e-03 mg/ml ; 8.61e-06 mol/l
Class	Moderately soluble

Pharmacokinetics	
GI absorption	High
BBB permeant	No
P-gp substrate	No
CYP1A2 inhibitor	No
CYP2C19 inhibitor	No
CYP2C9 inhibitor	No
CYP2D6 inhibitor	No
CYP3A4 inhibitor	No
Log K_p (skin permeation)	-6.99 cm/s

Druglikeness	
Lipinski	Yes; 0 violation
Ghose	Yes
Veber	Yes
Egan	Yes
Muegge	Yes
Bioavailability Score	0.55

Medicinal Chemistry	
PAINS	0 alert
Brenk	3 alerts: imine_1, imine_2, thiocarbonyl_group
Leadlikeness	Yes
Synthetic accessibility	2.60

Compound 14

Molecule 1

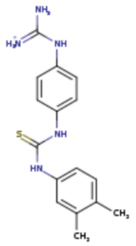

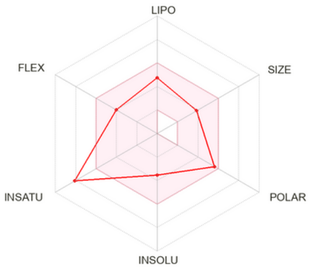

SMILES S=C(Nc1ccc(c(c1)C)C)Nc1ccc(cc1)NC(=[NH2+])N

Physicochemical Properties	
Formula	C16H20N5S+
Molecular weight	314.43 g/mol
Num. heavy atoms	22
Num. arom. heavy atoms	12
Fraction Csp3	0.12
Num. rotatable bonds	6
Num. H-bond acceptors	0
Num. H-bond donors	5
Molar Refractivity	98.57
TPSA	119.79 Å²

Lipophilicity	
Log $P_{o/w}$ (iLOGP)	1.98
Log $P_{o/w}$ (XLOGP3)	2.78
Log $P_{o/w}$ (WLOGP)	1.03
Log $P_{o/w}$ (MLOGP)	2.63
Log $P_{o/w}$ (SILICOS-IT)	2.81
Consensus Log $P_{o/w}$	2.24

Water Solubility	
Log *S* (ESOL)	-3.55
Solubility	8.89e-02 mg/ml ; 2.83e-04 mol/l
Class	Soluble
Log *S* (Ali)	-4.95
Solubility	3.52e-03 mg/ml ; 1.12e-05 mol/l
Class	Moderately soluble
Log *S* (SILICOS-IT)	-5.71
Solubility	6.09e-04 mg/ml ; 1.94e-06 mol/l
Class	Moderately soluble

Pharmacokinetics	
GI absorption	High
BBB permeant	No
P-gp substrate	No
CYP1A2 inhibitor	No
CYP2C19 inhibitor	No
CYP2C9 inhibitor	No
CYP2D6 inhibitor	Yes
CYP3A4 inhibitor	Yes
Log K_p (skin permeation)	-6.24 cm/s

Druglikeness	
Lipinski	Yes; 0 violation
Ghose	Yes
Veber	Yes
Egan	Yes
Muegge	Yes
Bioavailability Score	0.55

Medicinal Chemistry	
PAINS	0 alert
Brenk	3 alerts: imine_1, imine_2, thiocarbonyl_group
Leadlikeness	Yes
Synthetic accessibility	2.49

Compound 15

Molecule 1

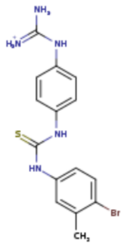

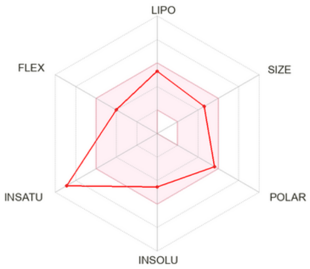

SMILES S=C(Nc1ccc(c(c1)C)Br)Nc1ccc(cc1)NC(=[NH2+])N

Physicochemical Properties	
Formula	C15H17BrN5S+
Molecular weight	379.30 g/mol
Num. heavy atoms	22
Num. arom. heavy atoms	12
Fraction Csp3	0.07
Num. rotatable bonds	6
Num. H-bond acceptors	0
Num. H-bond donors	5
Molar Refractivity	101.30
TPSA	119.79 Å²

Lipophilicity	
Log $P_{o/w}$ (iLOGP)	2.03
Log $P_{o/w}$ (XLOGP3)	3.74
Log $P_{o/w}$ (WLOGP)	1.48
Log $P_{o/w}$ (MLOGP)	3.02
Log $P_{o/w}$ (SILICOS-IT)	2.98
Consensus Log $P_{o/w}$	2.65

Water Solubility	
Log *S* (ESOL)	-4.56
Solubility	1.06e-02 mg/ml ; 2.78e-05 mol/l
Class	Moderately soluble
Log *S* (Ali)	-5.95
Solubility	4.28e-04 mg/ml ; 1.13e-06 mol/l
Class	Moderately soluble
Log *S* (SILICOS-IT)	-6.13
Solubility	2.78e-04 mg/ml ; 7.33e-07 mol/l
Class	Poorly soluble

Pharmacokinetics	
GI absorption	High
BBB permeant	No
P-gp substrate	No
CYP1A2 inhibitor	No
CYP2C19 inhibitor	No
CYP2C9 inhibitor	Yes
CYP2D6 inhibitor	No
CYP3A4 inhibitor	Yes
Log K_p (skin permeation)	-5.96 cm/s

Druglikeness	
Lipinski	Yes; 0 violation
Ghose	Yes
Veber	Yes
Egan	Yes
Muegge	Yes
Bioavailability Score	0.55

Medicinal Chemistry	
PAINS	0 alert
Brenk	3 alerts: imine_1, imine_2, thiocarbonyl_group
Leadlikeness	No; 2 violations: MW>350, XLOGP3>3.5
Synthetic accessibility	2.54

Compound 16

Molecule 1

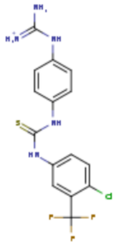

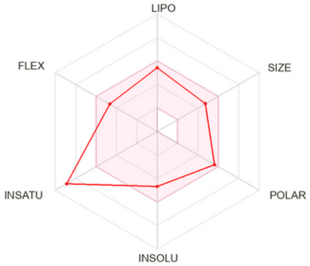

SMILES S=C(Nc1ccc(c(c1)C(F)(F)F)Cl)Nc1ccc(cc1)NC(=[NH2+])N

Physicochemical Properties	
Formula	C15H14ClF3N5S+
Molecular weight	388.82 g/mol
Num. heavy atoms	25
Num. arom. heavy atoms	12
Fraction Csp3	0.07
Num. rotatable bonds	7
Num. H-bond acceptors	3
Num. H-bond donors	5
Molar Refractivity	98.65
TPSA	119.79 Å²

Lipophilicity	
Log $P_{o/w}$ (iLOGP)	1.76
Log $P_{o/w}$ (XLOGP3)	4.01
Log $P_{o/w}$ (WLOGP)	3.23
Log $P_{o/w}$ (MLOGP)	3.26
Log $P_{o/w}$ (SILICOS-IT)	3.54
Consensus Log $P_{o/w}$	3.16

Water Solubility	
Log *S* (ESOL)	-4.67
Solubility	8.31e-03 mg/ml ; 2.14e-05 mol/l
Class	Moderately soluble
Log *S* (Ali)	-6.23
Solubility	2.30e-04 mg/ml ; 5.92e-07 mol/l
Class	Poorly soluble
Log *S* (SILICOS-IT)	-6.39
Solubility	1.58e-04 mg/ml ; 4.06e-07 mol/l
Class	Poorly soluble

Pharmacokinetics	
GI absorption	High
BBB permeant	No
P-gp substrate	No
CYP1A2 inhibitor	No
CYP2C19 inhibitor	No
CYP2C9 inhibitor	Yes
CYP2D6 inhibitor	No
CYP3A4 inhibitor	Yes
Log K_p (skin permeation)	-5.82 cm/s

Druglikeness	
Lipinski	Yes; 0 violation
Ghose	Yes
Veber	Yes
Egan	Yes
Muegge	Yes
Bioavailability Score	0.55

Medicinal Chemistry	
PAINS	0 alert
Brenk	3 alerts: imine_1, imine_2, thiocarbonyl_group
Leadlikeness	No; 2 violations: MW>350, XLOGP3>3.5
Synthetic accessibility	2.61

Compound 17

Molecule 1

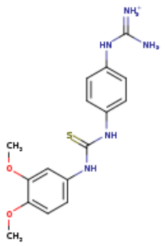

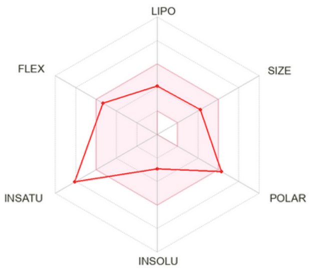

SMILES COc1cc(ccc1OC)NC(=S)Nc1ccc(cc1)NC(=[NH2+])N

Physicochemical Properties	
Formula	C16H20N5O2S+
Molecular weight	346.43 g/mol
Num. heavy atoms	24
Num. arom. heavy atoms	12
Fraction Csp3	0.12
Num. rotatable bonds	8
Num. H-bond acceptors	2
Num. H-bond donors	5
Molar Refractivity	101.62
TPSA	138.25 Å²

Lipophilicity	
Log $P_{o/w}$ (iLOGP)	2.12
Log $P_{o/w}$ (XLOGP3)	1.72
Log $P_{o/w}$ (WLOGP)	0.43
Log $P_{o/w}$ (MLOGP)	1.55
Log $P_{o/w}$ (SILICOS-IT)	1.91
Consensus Log $P_{o/w}$	1.55

Water Solubility	
Log *S* (ESOL)	-2.91
Solubility	4.23e-01 mg/ml ; 1.22e-03 mol/l
Class	Soluble
Log *S* (Ali)	-4.24
Solubility	2.00e-02 mg/ml ; 5.76e-05 mol/l
Class	Moderately soluble
Log *S* (SILICOS-IT)	-5.17
Solubility	2.32e-03 mg/ml ; 6.68e-06 mol/l
Class	Moderately soluble

Pharmacokinetics	
GI absorption	High
BBB permeant	No
P-gp substrate	No
CYP1A2 inhibitor	No
CYP2C19 inhibitor	No
CYP2C9 inhibitor	No
CYP2D6 inhibitor	No
CYP3A4 inhibitor	No
Log K_p (skin permeation)	-7.19 cm/s

Druglikeness	
Lipinski	Yes; 0 violation
Ghose	Yes
Veber	Yes
Egan	No; 1 violation: TPSA>131.6
Muegge	Yes
Bioavailability Score	0.55

Medicinal Chemistry	
PAINS	0 alert
Brenk	3 alerts: imine_1, imine_2, thiocarbonyl_group
Leadlikeness	No; 1 violation: Rotors>7
Synthetic accessibility	2.82

Compound 18

Molecule 1

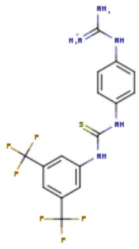

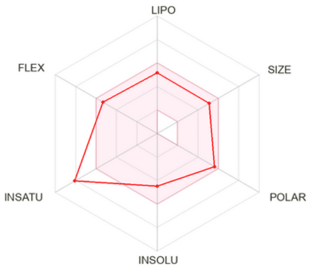

SMILES S=C(Nc1cc(cc(c1)C(F)(F)F)C(F)(F)F)Nc1ccc(cc1)NC(=[NH2+])N

Physicochemical Properties	
Formula	C16H14F6N5S+
Molecular weight	422.37 g/mol
Num. heavy atoms	28
Num. arom. heavy atoms	12
Fraction Csp3	0.12
Num. rotatable bonds	8
Num. H-bond acceptors	6
Num. H-bond donors	5
Molar Refractivity	98.64
TPSA	119.79 Å²

Lipophilicity	
Log $P_{o/w}$ (iLOGP)	1.85
Log $P_{o/w}$ (XLOGP3)	3.55
Log $P_{o/w}$ (WLOGP)	4.75
Log $P_{o/w}$ (MLOGP)	3.61
Log $P_{o/w}$ (SILICOS-IT)	4.01
Consensus Log $P_{o/w}$	3.56

Water Solubility	
Log *S* (ESOL)	-4.48
Solubility	1.38e-02 mg/ml ; 3.28e-05 mol/l
Class	Moderately soluble
Log *S* (Ali)	-5.75
Solubility	7.50e-04 mg/ml ; 1.78e-06 mol/l
Class	Moderately soluble
Log *S* (SILICOS-IT)	-6.63
Solubility	9.86e-05 mg/ml ; 2.33e-07 mol/l
Class	Poorly soluble

Pharmacokinetics	
GI absorption	Low
BBB permeant	No
P-gp substrate	No
CYP1A2 inhibitor	No
CYP2C19 inhibitor	No
CYP2C9 inhibitor	No
CYP2D6 inhibitor	No
CYP3A4 inhibitor	No
Log K_p (skin permeation)	-6.36 cm/s

Druglikeness	
Lipinski	Yes; 0 violation
Ghose	Yes
Veber	Yes
Egan	Yes
Muegge	Yes
Bioavailability Score	0.55

Medicinal Chemistry	
PAINS	0 alert
Brenk	3 alerts: imine_1, imine_2, thiocarbonyl_group
Leadlikeness	No; 3 violations: MW>350, Rotors>7, XLOGP3>3.5
Synthetic accessibility	2.77

Compound 19

Molecule 1

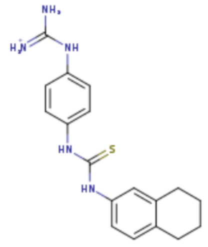

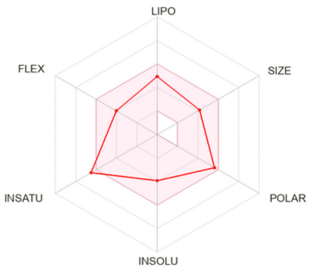

SMILES S=C(Nc1ccc2c(c1)CCCC2)Nc1ccc(cc1)NC(=[NH2+])N

Physicochemical Properties	
Formula	C18H22N5S+
Molecular weight	340.47 g/mol
Num. heavy atoms	24
Num. arom. heavy atoms	12
Fraction Csp3	0.22
Num. rotatable bonds	6
Num. H-bond acceptors	0
Num. H-bond donors	5
Molar Refractivity	106.07
TPSA	119.79 Å²

Lipophilicity	
Log $P_{o/w}$ (iLOGP)	2.26
Log $P_{o/w}$ (XLOGP3)	3.16
Log $P_{o/w}$ (WLOGP)	1.29
Log $P_{o/w}$ (MLOGP)	2.72
Log $P_{o/w}$ (SILICOS-IT)	3.19
Consensus Log $P_{o/w}$	2.52

Water Solubility	
Log *S* (ESOL)	-3.92
Solubility	4.13e-02 mg/ml ; 1.21e-04 mol/l
Class	Soluble
Log *S* (Ali)	-5.35
Solubility	1.54e-03 mg/ml ; 4.51e-06 mol/l
Class	Moderately soluble
Log *S* (SILICOS-IT)	-6.03
Solubility	3.16e-04 mg/ml ; 9.29e-07 mol/l
Class	Poorly soluble

Pharmacokinetics	
GI absorption	High
BBB permeant	No
P-gp substrate	Yes
CYP1A2 inhibitor	Yes
CYP2C19 inhibitor	Yes
CYP2C9 inhibitor	No
CYP2D6 inhibitor	Yes
CYP3A4 inhibitor	Yes
Log K_p (skin permeation)	-6.13 cm/s

Druglikeness	
Lipinski	Yes; 0 violation
Ghose	Yes
Veber	Yes
Egan	Yes
Muegge	Yes
Bioavailability Score	0.55

Medicinal Chemistry	
PAINS	0 alert
Brenk	3 alerts: imine_1, imine_2, thiocarbonyl_group
Leadlikeness	Yes
Synthetic accessibility	2.76

Compound 20

Molecule 1

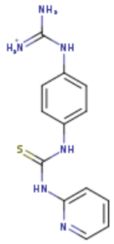

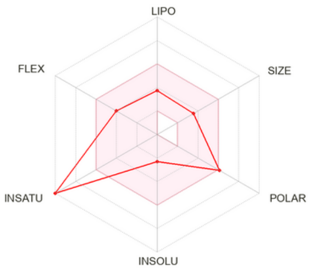

SMILES S=C(Nc1ccccn1)Nc1ccc(cc1)NC(=[NH2+])N

Physicochemical Properties	
Formula	C13H15N6S+
Molecular weight	287.36 g/mol
Num. heavy atoms	20
Num. arom. heavy atoms	12
Fraction Csp3	0.00
Num. rotatable bonds	6
Num. H-bond acceptors	1
Num. H-bond donors	5
Molar Refractivity	86.43
TPSA	132.68 Å²

Lipophilicity	
Log $P_{o/w}$ (iLOGP)	1.38
Log $P_{o/w}$ (XLOGP3)	1.04
Log $P_{o/w}$ (WLOGP)	-0.20
Log $P_{o/w}$ (MLOGP)	1.06
Log $P_{o/w}$ (SILICOS-IT)	1.25
Consensus Log $P_{o/w}$	0.91

Water Solubility	
Log *S* (ESOL)	-2.32
Solubility	1.36e+00 mg/ml ; 4.73e-03 mol/l
Class	Soluble
Log *S* (Ali)	-3.42
Solubility	1.10e-01 mg/ml ; 3.83e-04 mol/l
Class	Soluble
Log *S* (SILICOS-IT)	-4.58
Solubility	7.58e-03 mg/ml ; 2.64e-05 mol/l
Class	Moderately soluble

Pharmacokinetics	
GI absorption	High
BBB permeant	No
P-gp substrate	No
CYP1A2 inhibitor	No
CYP2C19 inhibitor	No
CYP2C9 inhibitor	No
CYP2D6 inhibitor	No
CYP3A4 inhibitor	No
Log K_p (skin permeation)	-7.31 cm/s

Druglikeness	
Lipinski	Yes; 0 violation
Ghose	Yes
Veber	Yes
Egan	No; 1 violation: TPSA>131.6
Muegge	Yes
Bioavailability Score	0.55

Medicinal Chemistry	
PAINS	0 alert
Brenk	3 alerts: imine_1, imine_2, thiocarbonyl_group
Leadlikeness	Yes
Synthetic accessibility	2.68

Compound 21

Molecule 1

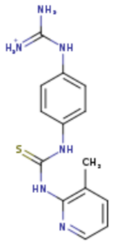

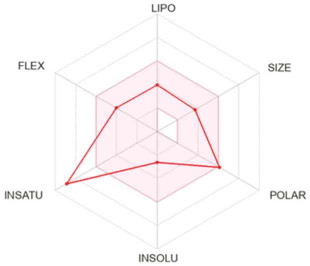

SMILES S=C(Nc1ncccc1C)Nc1ccc(cc1)NC(=[NH2+])N

Physicochemical Properties	
Formula	C14H17N6S+
Molecular weight	301.39 g/mol
Num. heavy atoms	21
Num. arom. heavy atoms	12
Fraction Csp3	0.07
Num. rotatable bonds	6
Num. H-bond acceptors	1
Num. H-bond donors	5
Molar Refractivity	91.40
TPSA	132.68 Å²

Lipophilicity	
Log $P_{o/w}$ (iLOGP)	1.77
Log $P_{o/w}$ (XLOGP3)	1.41
Log $P_{o/w}$ (WLOGP)	0.11
Log $P_{o/w}$ (MLOGP)	1.32
Log $P_{o/w}$ (SILICOS-IT)	1.75
Consensus Log $P_{o/w}$	1.27

Water Solubility	
Log *S* (ESOL)	-2.62
Solubility	7.17e-01 mg/ml ; 2.38e-03 mol/l
Class	Soluble
Log *S* (Ali)	-3.80
Solubility	4.77e-02 mg/ml ; 1.58e-04 mol/l
Class	Soluble
Log *S* (SILICOS-IT)	-4.96
Solubility	3.30e-03 mg/ml ; 1.10e-05 mol/l
Class	Moderately soluble

Pharmacokinetics	
GI absorption	High
BBB permeant	No
P-gp substrate	Yes
CYP1A2 inhibitor	Yes
CYP2C19 inhibitor	No
CYP2C9 inhibitor	No
CYP2D6 inhibitor	No
CYP3A4 inhibitor	No
Log K_p (skin permeation)	-7.14 cm/s

Druglikeness	
Lipinski	Yes; 0 violation
Ghose	Yes
Veber	Yes
Egan	No; 1 violation: TPSA>131.6
Muegge	Yes
Bioavailability Score	0.55

Medicinal Chemistry	
PAINS	0 alert
Brenk	3 alerts: imine_1, imine_2, thiocarbonyl_group
Leadlikeness	Yes
Synthetic accessibility	2.69

Compound 22

Molecule 1

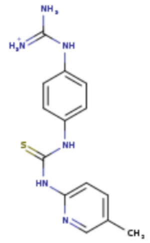

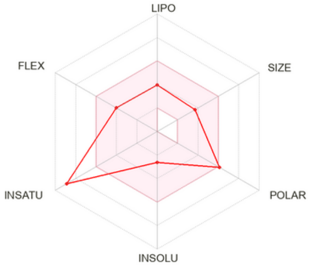

SMILES S=C(Nc1ccc(cn1)C)Nc1ccc(cc1)NC(=[NH2+])N

Physicochemical Properties	
Formula	C14H17N6S+
Molecular weight	301.39 g/mol
Num. heavy atoms	21
Num. arom. heavy atoms	12
Fraction Csp3	0.07
Num. rotatable bonds	6
Num. H-bond acceptors	1
Num. H-bond donors	5
Molar Refractivity	91.40
TPSA	132.68 Å²

Lipophilicity	
Log $P_{o/w}$ (iLOGP)	1.55
Log $P_{o/w}$ (XLOGP3)	1.41
Log $P_{o/w}$ (WLOGP)	0.11
Log $P_{o/w}$ (MLOGP)	1.32
Log $P_{o/w}$ (SILICOS-IT)	1.75
Consensus Log $P_{o/w}$	1.23

Water Solubility	
Log *S* (ESOL)	-2.62
Solubility	7.17e-01 mg/ml ; 2.38e-03 mol/l
Class	Soluble
Log *S* (Ali)	-3.80
Solubility	4.77e-02 mg/ml ; 1.58e-04 mol/l
Class	Soluble
Log *S* (SILICOS-IT)	-4.96
Solubility	3.30e-03 mg/ml ; 1.10e-05 mol/l
Class	Moderately soluble

Pharmacokinetics	
GI absorption	High
BBB permeant	No
P-gp substrate	Yes
CYP1A2 inhibitor	Yes
CYP2C19 inhibitor	No
CYP2C9 inhibitor	No
CYP2D6 inhibitor	No
CYP3A4 inhibitor	No
Log K_p (skin permeation)	-7.14 cm/s

Druglikeness	
Lipinski	Yes; 0 violation
Ghose	Yes
Veber	Yes
Egan	No; 1 violation: TPSA>131.6
Muegge	Yes
Bioavailability Score	0.55

Medicinal Chemistry	
PAINS	0 alert
Brenk	3 alerts: imine_1, imine_2, thiocarbonyl_group
Leadlikeness	Yes
Synthetic accessibility	2.69

Compound 23

Molecule 1

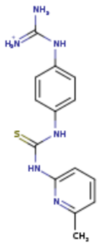

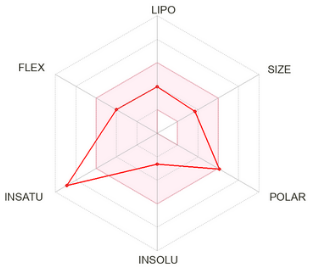

SMILES S=C(Nc1cccc(n1)C)Nc1ccc(cc1)NC(=[NH2+])N

Physicochemical Properties	
Formula	C14H17N6S+
Molecular weight	301.39 g/mol
Num. heavy atoms	21
Num. arom. heavy atoms	12
Fraction Csp3	0.07
Num. rotatable bonds	6
Num. H-bond acceptors	1
Num. H-bond donors	5
Molar Refractivity	91.40
TPSA	132.68 Å²

Lipophilicity	
Log $P_{o/w}$ (iLOGP)	1.56
Log $P_{o/w}$ (XLOGP3)	1.44
Log $P_{o/w}$ (WLOGP)	0.11
Log $P_{o/w}$ (MLOGP)	1.32
Log $P_{o/w}$ (SILICOS-IT)	1.75
Consensus Log $P_{o/w}$	1.24

Water Solubility	
Log *S* (ESOL)	-2.64
Solubility	6.86e-01 mg/ml ; 2.28e-03 mol/l
Class	Soluble
Log *S* (Ali)	-3.83
Solubility	4.44e-02 mg/ml ; 1.47e-04 mol/l
Class	Soluble
Log *S* (SILICOS-IT)	-4.96
Solubility	3.30e-03 mg/ml ; 1.10e-05 mol/l
Class	Moderately soluble

Pharmacokinetics	
GI absorption	High
BBB permeant	No
P-gp substrate	Yes
CYP1A2 inhibitor	Yes
CYP2C19 inhibitor	No
CYP2C9 inhibitor	No
CYP2D6 inhibitor	No
CYP3A4 inhibitor	No
Log K_p (skin permeation)	-7.12 cm/s

Druglikeness	
Lipinski	Yes; 0 violation
Ghose	Yes
Veber	Yes
Egan	No; 1 violation: TPSA>131.6
Muegge	Yes
Bioavailability Score	0.55

Medicinal Chemistry	
PAINS	0 alert
Brenk	3 alerts: imine_1, imine_2, thiocarbonyl_group
Leadlikeness	Yes
Synthetic accessibility	2.75

Compound 24

Molecule 1

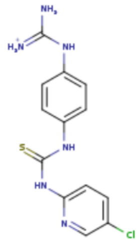

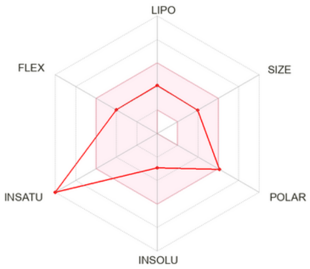

SMILES S=C(Nc1ccc(cn1)Cl)Nc1ccc(cc1)NC(=[NH2+])N

Physicochemical Properties	
Formula	C13H14ClN6S+
Molecular weight	321.81 g/mol
Num. heavy atoms	21
Num. arom. heavy atoms	12
Fraction Csp3	0.00
Num. rotatable bonds	6
Num. H-bond acceptors	1
Num. H-bond donors	5
Molar Refractivity	91.44
TPSA	132.68 Å²

Lipophilicity	
Log $P_{o/w}$ (iLOGP)	1.45
Log $P_{o/w}$ (XLOGP3)	1.67
Log $P_{o/w}$ (WLOGP)	0.46
Log $P_{o/w}$ (MLOGP)	1.59
Log $P_{o/w}$ (SILICOS-IT)	1.90
Consensus Log $P_{o/w}$	1.41

Water Solubility	
Log *S* (ESOL)	-2.91
Solubility	3.92e-01 mg/ml ; 1.22e-03 mol/l
Class	Soluble
Log *S* (Ali)	-4.07
Solubility	2.74e-02 mg/ml ; 8.50e-05 mol/l
Class	Moderately soluble
Log *S* (SILICOS-IT)	-5.18
Solubility	2.14e-03 mg/ml ; 6.64e-06 mol/l
Class	Moderately soluble

Pharmacokinetics	
GI absorption	High
BBB permeant	No
P-gp substrate	No
CYP1A2 inhibitor	Yes
CYP2C19 inhibitor	No
CYP2C9 inhibitor	No
CYP2D6 inhibitor	No
CYP3A4 inhibitor	No
Log K_p (skin permeation)	-7.08 cm/s

Druglikeness	
Lipinski	Yes; 0 violation
Ghose	Yes
Veber	Yes
Egan	No; 1 violation: TPSA>131.6
Muegge	Yes
Bioavailability Score	0.55

Medicinal Chemistry	
PAINS	0 alert
Brenk	3 alerts: imine_1, imine_2, thiocarbonyl_group
Leadlikeness	Yes
Synthetic accessibility	2.70

Compound 25

Molecule 1

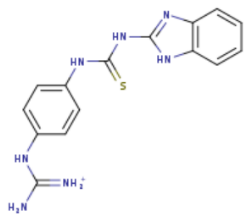

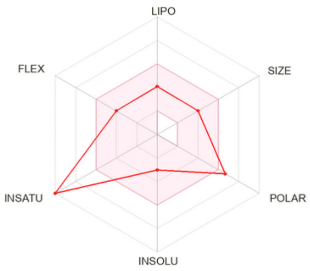

SMILES S=C(Nc1nc2c([nH]1)cccc2)Nc1ccc(cc1)NC(=[NH2+])N

Physicochemical Properties	
Formula	C15H16N7S+
Molecular weight	326.40 g/mol
Num. heavy atoms	23
Num. arom. heavy atoms	15
Fraction Csp3	0.00
Num. rotatable bonds	6
Num. H-bond acceptors	1
Num. H-bond donors	6
Molar Refractivity	98.29
TPSA	148.47 Å²

Lipophilicity	
Log $P_{o/w}$ (iLOGP)	1.27
Log $P_{o/w}$ (XLOGP3)	1.68
Log $P_{o/w}$ (WLOGP)	0.29
Log $P_{o/w}$ (MLOGP)	1.57
Log $P_{o/w}$ (SILICOS-IT)	1.81
Consensus Log $P_{o/w}$	1.32

Water Solubility	
Log *S* (ESOL)	-3.01
Solubility	3.20e-01 mg/ml ; 9.80e-04 mol/l
Class	Soluble
Log *S* (Ali)	-4.41
Solubility	1.26e-02 mg/ml ; 3.87e-05 mol/l
Class	Moderately soluble
Log *S* (SILICOS-IT)	-5.45
Solubility	1.17e-03 mg/ml ; 3.58e-06 mol/l
Class	Moderately soluble

Pharmacokinetics	
GI absorption	Low
BBB permeant	No
P-gp substrate	Yes
CYP1A2 inhibitor	Yes
CYP2C19 inhibitor	No
CYP2C9 inhibitor	No
CYP2D6 inhibitor	No
CYP3A4 inhibitor	No
Log K_p (skin permeation)	-7.10 cm/s

Druglikeness	
Lipinski	Yes; 1 violation: NHorOH>5
Ghose	Yes
Veber	No; 1 violation: TPSA>140
Egan	No; 1 violation: TPSA>131.6
Muegge	No; 1 violation: H-don>5
Bioavailability Score	0.55

Medicinal Chemistry	
PAINS	0 alert
Brenk	3 alerts: imine_1, imine_2, thiocarbonyl_group
Leadlikeness	Yes
Synthetic accessibility	2.76

References

1. Natesh Singh, Etienne Decroly, Abdel-Majid Khatib, Bruno O. Villoutreix, Structure-based drug repositioning over the human TMPRSS2 protease domain: search for chemical probes able to repress SARS-CoV-2 Spike protein cleavages, European Journal of Pharmaceutical Sciences, Volume 153, 2020, 105495,

2. Andrew Waterhouse, Martino Bertoni, Stefan Bienert, Gabriel Studer, Gerardo Tauriello, Rafal Gumienny, Florian T Heer, Tjaart A P de Beer, Christine Rempfer, Lorenza Bordoli, Rosalba Lepore, Torsten Schwede, SWISS-MODEL: homology modelling of protein structures and complexes, *Nucleic Acids Research*, Volume 46, Issue W1, 2 July 2018, Pages W296–W303

3. Gaussian16, Revision C.01, M. J. Frisch, G. W. Trucks, H. B. Schlegel, G. E. Scuseria, M. A. Robb, J. R. Cheeseman, G. Scalmani, V. Barone, G. A. Petersson, H. Nakatsuji, X. Li, M. Caricato, A. V. Marenich, J. Bloino, B. G. Janesko, R. Gomperts, B. Mennucci, H. P. Hratchian, J. V. Ortiz, A. F. Izmaylov, J. L. Sonnenberg, D. Williams-Young, F. Ding, F. Lipparini, F. Egidi, J. Goings, B. Peng, A. Petrone, T. Henderson,

D. Ranasinghe, V. G. Zakrzewski, J. Gao, N. Rega, G. Zheng, W. Liang, M. Hada, M. Ehara, K. Toyota, R. Fukuda, J. Hasegawa, M. Ishida, T. Nakajima, Y. Honda, O. Kitao, H. Nakai, T. Vreven, K. Throssell, J. A. Montgomery, Jr., J. E. Peralta, F. Ogliaro, M. J. Bearpark, J. J. Heyd, E. N. Brothers, K. N. Kudin, V. N. Staroverov, T. A. Keith, R. Kobayashi, J. Normand, K. Raghavachari, A. P. Rendell, J. C. Burant, S. S. Iyengar, J. Tomasi, M. Cossi, J. M. Millam, M. Klene, C. Adamo, R. Cammi, J. W. Ochterski, R. L. Martin, K. Morokuma, O. Farkas, J. B. Foresman, and D. J. Fox, Gaussian, Inc., Wallingford CT, 2016.

4. AutoDock Vina 1.2.0: New Docking Methods, Expanded Force Field, and Python Bindings. Eberhardt, Jerome, Santos-Martins, Diogo, Tillack, Andreas F., Forli, Stefano. J. Chem. Inf. Model. 2021, 61, 3891-3898

5. Ryne C. Johnston, Kun Yao, Zachary Kaplan, Monica Chelliah, Karl Leswing, Sean Seekins, Shawn Watts, David Calkins, Jackson Chief Elk, Steven V. Jerome, Matthew P. Repasky, and John C. Shelley. Epik: p*K*a and Protonation State Prediction through Machine Learning. *Journal of Chemical Theory and Computation* **2023** *19* (8), 2380-2388

6. Madhavi Sastry, G., Adzhigirey, M., Day, T. *et al.* Protein and ligand preparation: parameters, protocols, and influence on virtual screening enrichments. *J Comput Aided Mol Des* **27**, 221–234 (2013).

7. Richard A. Friesner, Jay L. Banks, Robert B. Murphy, Thomas A. Halgren, Jasna J. Klicic, Daniel T. Mainz, Matthew P. Repasky, Eric H. Knoll, Mee Shelley, Jason K. Perry, David E. Shaw, Perry Francis, and Peter S. Shenkin. Glide: A New Approach for Rapid, Accurate Docking and Scoring. 1. Method and Assessment of Docking Accuracy**.** *Journal of Medicinal Chemistry* **2004** *47* (7), 1739-1749

8. Richard A. Friesner, Robert B. Murphy, Matthew P. Repasky, Leah L. Frye, Jeremy R. Greenwood, Thomas A. Halgren, Paul C. Sanschagrin, and Daniel T. Mainz. Extra Precision Glide: Docking and Scoring Incorporating a Model of Hydrophobic Enclosure for Protein−Ligand Complexes**.** *Journal of Medicinal Chemistry* **2006** *49* (21), 6177-6196

9. Woody Sherman, Tyler Day, Matthew P. Jacobson, Richard A. Friesner, and Ramy Farid. Novel Procedure for Modeling Ligand/Receptor Induced Fit Effects**.** *Journal of Medicinal Chemistry* **2006** *49* (2), 534-553

10. E. D. Goddard-Borger, R. V. Stick, "An Efficient, Inexpensive, and Shelf-Stable Diazotransfer Reagent: Imidazole-1-sulfonyl Azide Hydrochloride" *Org. Lett.* **2007**, *9*, 3797-3800.

11. M. Minneci, M. Misevicius, I. Rozas, "Green Synthesis of Nitroaryl Thioureas: Towards an Improved Preparation of Guanidinium DNA Binders" *Bioorg. Med. Chem. Lett*. **2023**, *90*,129346.

12. (a) S. K. Hamilton, D. E. Wilkinson, G. S. Hamilton, Y.-Q. Wu, "Microwave-Assisted Synthesis of *N,N'*-Diaryl Cyanoguanidines" *Org. Lett*. **2005**, *7*, 2429-2431. (b) Zheng Li, Zhi-Yuan Wang, Yan-Long Zhao, Yu-Lin Xing, Wei Zhu, "An Environmentally Benign Method for the Synthesis of Symmetrical *N,N '*-Disubstituted Thioureas in a Water Medium" *Phosphorus, Sulfur, and Silicon*, **2005**, *180*, 2745–2750.

13. F. Rodriguez, I. Rozas, M. Kaiser, R. Brun, B. Nguyen, W.D. Wilson, R.N. Garcia, C. Dardonville, "New bis(2-aminoimidazoline) and bisguanidine DNA minor groove binders with potent *in vivo* antitrypanosomal and antiplasmodial activity" *J. Med. Chem*. **2008**, *51*, 909-923.
